# High-Speed Precision Machining and Surface Roughness Determination of Freeform Curves Using Galerkin-NURBS Interpolation and Jerk-Limited Trajectory Planning

**DOI:** 10.3390/s26144441

**Published:** 2026-07-13

**Authors:** Usman Haladu Garba, Taiyong Wang, Ying Tian, Jing Kang, Chong Tian

**Affiliations:** School of Mechanical Engineering, Tianjin University, Tianjin 300354, China; tywang@tju.edu.cn (T.W.); 1021201038@tju.edu.cn (J.K.); tc1920965265@tju.edu.cn (C.T.)

**Keywords:** Galerkin-NURBS interpolation, jerk-limited trajectory planning, high-speed machining, feedrate optimization, curvature-adaptive control, surface roughness measurement, profilometer-based sensing, encoder feedback sensors

## Abstract

**Highlights:**

**What are the main findings?**

- The proposed Galerkin-NURBS interpolation framework reduces geometric approximation errors and computational overhead, particularly in high-curvature regions, while the jerk-limited S-curve trajectory planning enforces $C^{3}$ continuity, minimizing vibrations and enabling precise dynamic control.
- Experimental validation on a five-axis CNC machine demonstrates that the proposed method outperforms feed-sensitive region constant (FSRC), optimized S-shaped quantitative feed (OSQF), and cubic and quartic S-shape feedrate (CQSF) benchmarks, achieving a 32.9% reduction in processing time and a 35.1% reduction in interpolation steps, while maintaining surface roughness ($R_{a}$) values between 0.1271 μm and 0.2009 μm.

**What are the implications of the main findings?**

- The curvature-adaptive feedrate optimization enables high-speed machining of complex freeform geometries (e.g., aerospace and medical components), without sacrificing precision, by dynamically balancing speed in straight segments and accuracy in tight curves.
- The sensor-validated framework improves machining efficiency and vibration control, while profilometer measurements confirm that surface quality ($R_{a}$ < 0.2 μm) is maintained within ISO 21920-1:2021 compliance.

**Abstract:**

High-speed machining of complex freeform geometries faces fundamental challenges in balancing computational efficiency, kinematic constraints, and precision, particularly in high-curvature regions where traditional interpolation methods suffer from geometric errors and jerk-induced vibrations. This study presents a Galerkin-NURBS interpolation framework that integrates Galerkin projection to optimize NURBS parameterization, minimizing geometric approximation error, and couples it with a jerk-limited S-curve trajectory planning algorithm that enforces $C^{3}$ continuity while respecting feedrate, acceleration, and jerk constraints. Numerical simulations and machining experiments were conducted on butterfly-shaped and horse-shaped curves using a five-axis CNC machine equipped with rotary/linear encoders and validated via profilometer-based surface roughness measurements. The proposed method achieved a 32.9% reduction in processing time (3.091 s) compared to the CQSF method (4.61 s) and a 35.1% reduction in interpolation steps relative to FSRC. Surface roughness ($R_{a}$) values ranged from 0.1271 μm to 0.2009 μm, with most measurements compliant with ISO 21920-1:2021; the maximum value (0.2009 μm) represents the upper bound of the standard’s high-precision threshold for aluminum alloy 6061. These findings demonstrate that the proposed framework significantly improves machining efficiency and surface quality while maintaining geometric fidelity, making it suitable for precision manufacturing applications where sensor-guided process optimization is critical.

## 1. Introduction

High-speed precision machining of complex geometries is a cornerstone of modern manufacturing, particularly in industries such as aerospace, automotive, and medical devices, where intricate designs and tight tolerances are paramount [1]. The demand for efficient and accurate machining has driven the adoption of advanced toolpath representation methods, such as Non-Uniform Rational B-Splines (NURBS). NURBS have emerged as a dominant standard due to their ability to model freeform surfaces with high flexibility and precision [2,3,4,5]. However, despite their widespread use, NURBS-based toolpaths present significant challenges in real-time computer numerical control (CNC) applications, particularly in regions of high curvature where geometric approximation errors and jerk-induced vibrations can degrade machining quality [6,7,8].

Traditional interpolation methods (like using straight lines (G01) or simple curves (G02/G03)) often approximate complex toolpaths with piecewise simplifications [9]. This can cause discontinuities in velocity and acceleration that compromise surface finish quality and reduce tool life [10,11]. NURBS interpolation reduces some of these problems by providing smoother toolpaths [12]. However, its implementation in CNC systems remains challenging due to computational inefficiencies and difficulties in maintaining kinematic limits during high-speed motion [13,14]. Recent advances in hybrid machining and robotic systems have addressed these limitations through innovative smoothing techniques. For instance, Shi et al. [15] introduced a toolpath smoothing method that achieved $G^{3}$ continuity by inserting quintic B-splines in the machine coordinate system (MCS). Their approach synchronizes tool position and orientation paths while constraining smoothing errors via Jacobian-based estimation. This method demonstrated improved machining efficiency and reduced joint jerk compared to workpiece coordinate system (WCS)-based methods, particularly in handling the nonlinear kinematics of hybrid robots. Similarly, Yang et al. [16] proposed a real-time toolpath smoothing method for 6R robot manipulators. They used asymmetrical Pythagorean-hodograph (PH) splines to achieve $C^{3}$ continuity and lower tracking errors through analytical spline construction. These studies highlight the importance of higher-order continuity in dynamic machining environments.

To address these challenges, researchers such as Lyu et al. [17] have developed a tool-path generation method using bounding box trees and KD trees to automate tool-path planning for complex surfaces. Their approach reduces manual programming time while maintaining precision and accuracy. Meanwhile, Li et al. [18] proposed a B-spline feedrate method for NURBS toolpaths using bidirectional scanning. Their work highlighted the importance of adaptive feedrate planning in high-speed machining.

Another critical issue in machining is chatter vibration, which adversely affects surface quality and tool longevity. Jafarzadeh et al. [19] investigated chatter in milling using simulation. Their model incorporated machine tool dynamics and friction effects to provide insights into how parameters such as axial depth of cut and feed rate influence stability. Their study provided a framework for reducing chatter in high-speed operations by highlighting the relationship between process parameters and vibrational behavior. Feedrate fluctuations caused by parameterization errors in NURBS interpolation can lead to uneven material removal and excessive tool wear [20,21]. Mechanical vibrations may also result from the lack of higher-order continuity (e.g., jerk control) in conventional trajectory planning, which can compromise machining accuracy [22,23]. To improve machining efficiency, many researchers have employed parametric interpolation techniques such as Taylor series expansion [24], predictor-corrector algorithms [25], and arc-length parameterization [26]. While these methods improve interpolation accuracy, they often struggle to balance real-time computation demands with kinematic constraints [27,28].

For example, the Taylor expansion method is computationally efficient but can introduce truncation errors that increase feedrate fluctuations in high-curvature regions [29]. Despite these challenges, the method remains widely used in practice. Arc-length parameterization techniques reduce geometric errors but require significant preprocessing and are not adaptable to dynamic feedrate adjustments [30,31]. Other studies have explored curvature-adaptive feedrate scheduling [32] and B-spline-based error compensation [33]; these methods optimize computational efficiency and motion smoothness.

Further refining these approaches, Wang et al. [34] established a theoretical framework for evaluating the consistency of toolpaths in CNC machining. Their study introduced strong and weak consistency metrics to assess the robustness of path smoothing and trajectory planning methods, particularly in single-point milling. Their work provides a novel surface-centric evaluation that ensures global continuity and high machining quality. They achieved this by analyzing the sensitivity of toolpaths to disturbances in input fold-paths. This framework connects spatial path optimization and temporal planning, providing a comprehensive evaluation tool for advanced interpolation methods.

To enhance real-time performance, modern CNC systems increasingly leverage parallel computing architectures [35] and machine learning-based feedrate prediction [36]. In recent years, machine learning and artificial intelligence techniques have emerged as promising tools for feedrate optimization and trajectory planning. Chien et al. [37] employed deep neural networks with a bidirectional LSTM architecture to predict machining cycle times and feedrates, achieving significant reductions in machining time while maintaining surface quality. Li et al. [38] applied reinforcement learning to CNC trajectory planning, demonstrating adaptive feedrate scheduling under varying cutting conditions. Kim et al. [39] developed a hybrid digital twin approach integrating physics-based models with data-driven updates for intelligent feedrate optimization in CNC machining. Their framework first builds a physics-based dynamics model, then employs an on-the-fly data-driven internal model to adapt to unknown dynamics and cutting force disturbances. Jiang et al. [40] developed a novel approach combining deep learning networks for tracking error prediction and reinforcement learning for compensation parameter optimization in CNC machining. Their method achieved over 99% prediction accuracy for tracking errors. While these methods show considerable promise, they often require extensive training data and may not guarantee constraint satisfaction in real-time applications. In contrast, the proposed approach offers a deterministic, model-based framework that explicitly enforces kinematic and geometric constraints without relying on data-driven training. Also, these approaches typically focus on either spatial path optimization or temporal planning, overlooking their coupled effects on machining dynamics [41]. Furthermore, while jerk-limited algorithms have proven effective for linear paths [42], their extension to NURBS toolpaths remains underexplored, particularly for five-axis machining [43].

**Figure 1:**
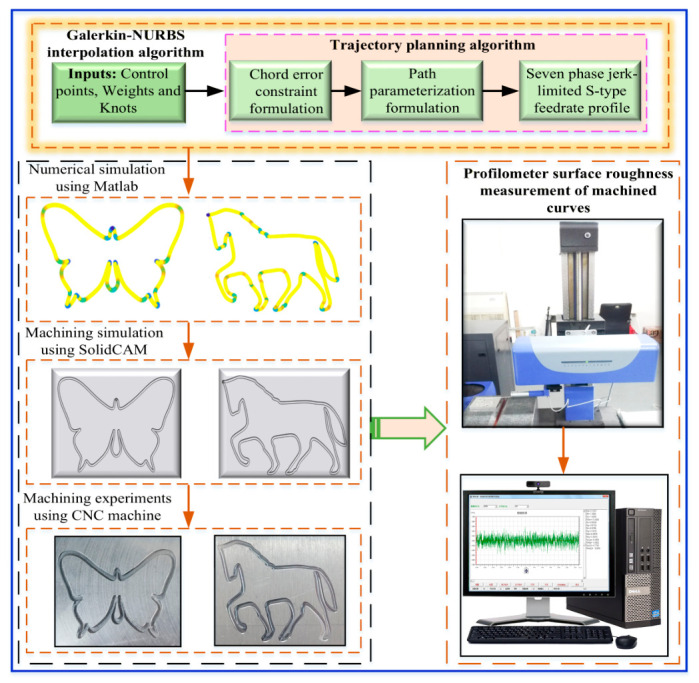
Implementation steps of the proposed method.

This paper addresses the above-mentioned challenges by introducing a Galerkin-NURBS interpolation framework that leverages Galerkin projection to refine NURBS parameterization. The method integrates a jerk-limited S-curve trajectory planner that follows the well-established formulation of Erkorkmaz and Altintas [2] for the seven-phase profile. However, the novelty of this work lies in the coupling between the Galerkin-optimized NURBS geometry and the S-curve planner, enabling curvature-adaptive feedrate scheduling with full $C^{3}$ continuity. The S-curve profile itself is known. However, its integration with Galerkin-optimized NURBS parameterization has not been previously demonstrated. The experiments show simultaneous improvements in processing time (32.9%), surface roughness ($R_{a}$ < 0.2 μm), and contour fidelity (±2 μm). Together, these advances constitute the novel contribution of this work. The steps of the work conducted in this study are shown in Figure 1.

This study develops an advanced trajectory planning framework for high-speed CNC machining to address critical challenges in precision, efficiency, and motion smoothness through six key contributions:

(1) A Galerkin-NURBS interpolation framework that integrates Galerkin projection with NURBS parameterization to minimize geometric error and enhance smoothness, particularly in high-curvature regions.
(2) A jerk-limited S-curve profile with $C^{3}$ continuity, formulated via a 7-phase planner to ensure smooth transitions in position, velocity, acceleration, and jerk, reducing vibrations and enabling precise dynamic control.
(3) Curvature-adaptive feedrate optimization dynamically adjusts velocities based on real-time geometric constraints (chord error, centripetal acceleration, and jerk) to balance speed in straight segments and precision in complex curves.
(4) A technique that separates path shape from movement timing using arc-length parameterization to maintain smooth motion on any toolpath.
(5) Experimental validation demonstrating a 32.9% faster processing time and 35.1% fewer interpolation steps compared to existing methods: FSRC (feed-sensitive region constant), OSQF (optimized S-shaped quantitative feed), and CQSF (cubic and quartic S-shaped feedrate). This was proven via complex freeform curves and 5-axis CNC tests. These advances bridge theoretical motion planning with industrial CNC demands as well as offering measurable gains in performance and surface quality.
(6) Rigorous experimental validation of machined freeform geometries via profilometry, confirming simultaneous achievement of sub-micron surface finish ($R_{a}$ < 0.2 μm) and contour fidelity within ±2 μm.

The NURBS representation and S-curve trajectory planning are based on established techniques. However, the novelty of this work lies in the coupling of Galerkin projection with NURBS parameterization, and its integration with the S-curve planner for curvature-adaptive feedrate scheduling. Together, these contributions enable improved geometric fidelity, computational efficiency, and motion smoothness.

The remainder of this paper is organized as follows. Section 2 presents the research methods, including the derivation of the Galerkin-NURBS interpolation, trajectory planning algorithm formulation (with chord error constraint), path parameterization, constraints, and feedrate profile generation using the 7-phase jerk-limited S-curve trajectory planning method. Section 3 presents the results and discussion. Section 4 describes the surface roughness test. Section 5 provides the conclusions.

## 2. Materials and Research Methods

### 2.1. Galerkin-NURBS Interpolation Derivation

This study introduces a Galerkin-NURBS interpolation framework that employs Galerkin projection to optimize NURBS parameterization, minimizing geometric approximation error, enhancing toolpath smoothness, and preserving computational efficiency. NURBS-based interpolation was selected for its advantages in high-speed machining applications, particularly its ability to maintain superior precision and accuracy. The NURBS curve is expressed as [44,45]:

(1) $$C\left(u\right)=\sum_{i=0}^{n}R_{i,p}\left(u\right)P_{i}=\frac{\sum_{i=0}^{n}N_{i,p}(u)w_{i}P_{i}}{\sum_{i=0}^{n}N_{i,p}(u)w_{i}}$$

To optimize computational efficiency, the rational function’s numerator A(u) and denominator w(u) are defined separately, yielding the following formulation:

(2) $$A\left(u\right)=\sum_{i=0}^{n}N_{i,p}(u)w_{i}P_{i}$$

(3) $$w\left(u\right)=\sum_{i=0}^{n}N_{i,p}(u)w_{i}$$

The NURBS curve representation relies on several key components: the rational basis function $R_{i,p}\left(u\right)$ of degree p, computed by normalizing the B-spline basis functions $N_{i,p}(u)$ with their associated weights ${{\{w}_{i}\}}_{i=0}^{n}$; the control points ${{\{P}_{i}\}}_{i=0}^{n}$ that define the curve’s geometric shape; the strictly positive weights ${{\{w}_{i}\}}_{i=0}^{n}$; and the curve parameter u∈[0,1]. Here, the numerator A(u) represents the weighted B-spline function, while the denominator w(u) serves as the normalizing weight function. The B-spline basis functions ${\{N}_{i,p}(u)\}$ are constructed for a non-uniform knot vector with parameter relationships m=n+p+1, where m+1 is the total number of knots and n+1 is the number of control points.

The knot sequence $\left\{u_{0},u_{1}\ldots ,\right.\left.u_{n+p+1}\right\}$ follows standard convention, with the first and last p+1 knots clamped at 0 and 1, respectively. The B-spline basis functions $N_{i,p}\left(u\right)$ are generated recursively via the Cox-de Boor algorithm, which guarantees $C^{\left\{p-1\right\}}$-continuous, piecewise-polynomial basis functions through the following recurrence relations [46]:

(4) Ni,0u=1, 0,  ifui≤u<ui+1otherwise         

(5) $$N_{i,p}\left(u\right)=\frac{u-u_{i}}{u_{i+p}-u_{i}}N_{i,p-1}\left(u\right)+\frac{u_{i+p+1}-u}{u_{i+p+1}-u_{i+1}}N_{i+1,\;\;p-1}\left(u\right)$$

To calculate the curvature of the NURBS curve, Equation (6) delineates the first-order derivatives of the function ${\{N}_{i,p}\left(u\right)\}$, while Equation (7) represents the second-order derivatives of the same function. These expressions were derived through a systematic process from the fundamental Equation (5). Accordingly, this approach affords a comprehensive understanding of the behavior and properties of the function. Equations (8) and (9) show the computed equations for the first-order as well as the second-order derivatives of the NURBS curve. These equations will be used in the next chapter and for the calculation of the curvature radius later on.

(6) $$N_{i,p}^{\prime }\left(u\right)=\frac{P}{u_{i+p}-u_{i}}N_{i,p-1}\left(u\right)+\frac{P}{u_{i+p+1}-u_{i+1}}N_{i+1,\;\;p-1}(u)$$

(7) $$N_{i,p}^{\prime \prime }\left(u\right)=\frac{P}{u_{i+p}-u_{i}}N_{i,p-1}^{\prime }\left(u\right)+\frac{P}{u_{i+p+1}-u_{i+1}}N_{i+1,p-1}^{\prime }\left(u\right)$$

(8) $$C^{\prime }\left(u\right)=\frac{\sum_{i=0}^{n}N_{i,p}^{\prime }\left(u\right)w_{i}P_{i}-C(u)\sum_{i=0}^{n}N_{i,p}^{\prime }\left(u\right)w_{i}}{\sum_{i=0}^{n}N_{i,p}\left(u\right)w_{i}}$$

(9) $$C^{\prime \prime }\left(u\right)=\frac{\sum_{i=0}^{n}N_{i,p}^{”}\left(u\right)w_{i}P_{i}-2C^{\prime }(u)\sum_{i=0}^{n}N_{i,p}^{\prime }\left(u\right)w_{i}-C(u)\sum_{i=0}^{n}N_{i,p}^{\prime }\left(u\right)w_{i}}{\sum_{i=0}^{n}N_{i,p}\left(u\right)w_{i}}$$

Therefore, the curvature of NURBS curve is expressed as:

(10) $$k=\frac{\left\|C^{\prime }(u)\times C^{\prime \prime }(u)\right\|}{{\left\|C^{\prime }(u)\right\|}^{3}}$$

The Galerkin projection method (Equations (11)–(15)) optimizes NURBS parameterization through a variational approach that minimizes geometric approximation error. This formulation simultaneously enhances toolpath smoothness and preserves computational efficiency for discrete CNC trajectory evaluation (Equations (12)–(14)).

(11) $$\int L\left(u^{h}\right)N_{i,p}\left(u\right)dΩ=0\;\;\;\forall i\;\epsilon \;\{0,\;\ldots ,n\}$$

In Equation (11), Ω denotes the parametric domain [0, 1] of the NURBS curve, and L is a second-order differential operator enforcing smoothness by minimizing curvature variations. This ensures the solution $u^{h}$ adheres to geometric continuity constraints and $u^{h}$ is the approximated solution expressed as:

(12) $$u^{h}=\sum_{j=0}^{n}N_{j,p}{(u)u}_{j}$$

The projection yields the linear system and it is expressed as:

(13) Ku=f

where K is the stiffness matrix expressed as:

(14) $$K_{ij}=a\left(N_{j,p},N_{i,p}\right)=\int \nabla N_{j,p}\cdot \nabla N_{i,p}dΩ$$

In Equation (14), the symbol a denotes a bilinear form operator representing the inner product of gradient basis functions ($\nabla N_{j,p}\cdot \nabla N_{i,p}$). $a\left(N_{j,p},N_{i,p}\right)$ denotes the bilinear form $\int \nabla N_{j,p}\cdot \nabla N_{i,p}dΩ$, which quantifies the coupling between basis functions and enforces smoothness in the solution. In Equation (15), f is a scalar forcing term, and $f_{i}$ is its projection onto the basis function $N_{i,p}$ via integration over Ω. This ensures the solution respects geometric constraints.

(15) $$f_{i}=\langle f,N_{i,p}\rangle =\int fN_{i,p}dΩ$$

The solution vector ${u=[u_{1},\;u_{2},\;\ldots ,\;u_{n}]}^{T}$ from Equation (13) represents the generalized displacements at the collocation points along the curve. To transfer these corrections to the NURBS geometry, the incremental displacement of each control point $P_{j}$ is computed as:

(16) $${\Delta P}_{j}=\sum_{i}\frac{N_{i,j}\left(u_{i}\right)\cdot {u_{i}\cdot w}_{j}}{\sum_{k}N_{i,k}(u_{i})\cdot w_{k}}$$

where $N_{i,j}\left(u_{i}\right)$ is the NURBS basis function of degree P evaluated at the parametric coordinate corresponding to the i-th collocation point, $u_{i}$ is the solution component at that point, and $w_{j}$ are the control-point weights. The updated control points are then:

(17) $$P_{j}^{new}=P_{j}^{old}+{\Delta P}_{j}$$

The knot vector remains unchanged, preserving the continuity properties of the original NURBS basis while improving the geometric fit through the Galerkin-optimized control-point positions. This completes the coupling between the variational projection (Equations (11)–(15)) and the NURBS representation (Equation (1)).

In CNC machining applications, the Galerkin-optimized NURBS representation (Equation (13)) is computationally evaluated at discrete parametric coordinates $u_{k}$ (Equations (18)–(20)). This discrete implementation enables efficient toolpath generation while rigorously maintaining the smoothness properties guaranteed by the continuous formulation.

(18) $$C_{k}=C(u_{k})$$

(19) $$u_{k}=u_{0}+k\Delta u$$

(20) k=0,…,N

The parametric step size $\Delta u=(u_{m}-u_{0})/N$ is defined between the initial ${(u}_{0})$ and final ${(u}_{m})$ parameter values. This formulation provides a rigorous mathematical foundation for NURBS interpolation, merging geometric flexibility with numerical robustness. The method proves particularly valuable for computational mechanics, CAD/CAM systems, and CNC machining, enabling high-fidelity modeling, precise geometric representation, and efficient toolpath generation.

### 2.2. Chord Error Constraint

**Figure 2:**
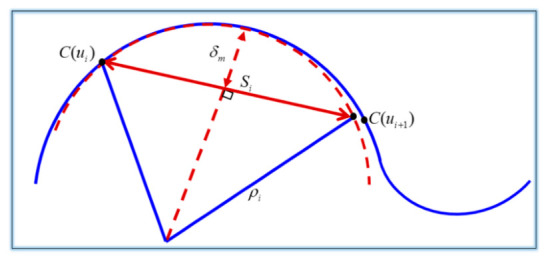
Chord error.

The trajectory planning algorithm must guarantee that the linear interpolation between successive sampled points,$\;{C(u}_{i})$, and ${C(u}_{i+1})$, remains within a predefined tolerance $\delta_{m}$ from the actual path. As illustrated in Figure 2, this deviation is geometrically limited by the radius of curvature (1/k), where k denotes the path curvature. The maximum permissible chord error $\delta_{m}$ is defined by:

(21) $$\delta_{m}=\rho -\sqrt{\rho^{2}-{\left(\frac{S_{i}}{2}\right)}^{2}}$$

Here, $\delta_{m}$ denotes the maximum permissible chord error (geometric deviation tolerance), ρ represents the toolpath’s radius of curvature (ρ=1/k, where *k* is curvature), and $S_{i}$ indicates the chord length between consecutive interpolated points ${C(u}_{i})$, and ${C(u}_{i+1})$.

The system parameters $a_{m}$, $J_{m}$ and $v_{m}$ were defined to represent the maximum acceleration, jerk limit, and velocity, respectively. These variables were utilized to establish the fundamental constraints of the system’s dynamic behavior.

To dynamically enforce this constraint, the feedrate ${v_{f}(u}_{i})$ is adaptively adjusted according to the sampling time. Here, *t* denotes the sampling time interval, which is equal to the interpolation period *T* used elsewhere in the manuscript (t=T=1 ms). The feedrate must comply with the following condition:

(22) $${v_{f}(u}_{i})=\frac{2}{t}\sqrt{\rho^{2}-{\left({\rho -\delta }_{m}\right)}^{2}}$$

Here, Equations (21) and (22) are foundational constraints. The final feedrate (Equation (27)) integrates jerk and acceleration limits, enabling smooth, high-order interpolation. This avoids linear segments by enforcing $C^{3}$ continuity via S-curve planning. Alternatively, this expression can be reformulated to explicitly show its dependence on $\delta_{m}$.

(23) $${v_{f}(u}_{i})=v_{m}=\frac{2}{t}\sqrt{2\rho \delta_{m}-\delta_{m}^{2}}$$

Expanding the terms inside the square root in Equation (23) gives:

(24) $$\rho^{2}-{\left({\rho -\delta }_{m}\right)}^{2}=\rho^{2}-\left(\rho^{2}-2{\rho \delta }_{m}+\delta_{m}^{2}\right)=2{\rho \delta }_{m}-\delta_{m}^{2}$$

The operational feedrate is additionally constrained by the system’s physical limitations. Specifically, centripetal acceleration boundaries are imposed to maintain traction integrity and avoid detrimental mechanical stresses:

(25) $$v_{fa}\left(u_{i}\right)=\sqrt{a_{m}\rho }$$

To ensure smooth motion generation, the jerk is bounded within specified limits:

(26) $$v_{fJ}\left(u_{i}\right)=\sqrt[{3}]{J_{m}\rho^{2}}$$

Here, $v_{fa}$ represents the feedrate limited by centripetal acceleration constraints and $v_{fJ}$ indicates the jerk-constrained feedrate. The resulting feedrate $v_{fn}$ is determined as the minimum value across all constraint conditions, guaranteeing compliance with both geometric path requirements and dynamic system capabilities:

(27) $$v_{fn}=min\left(\frac{2}{t}\sqrt{{2\rho \delta_{m}-\delta }_{m}^{2}}\;,\;\sqrt{a_{m}\rho }\;,\;\sqrt[{3}]{J_{m}\rho^{2}}\right)$$

High-curvature critical points are detected by applying a curvature threshold $k_{t}$, which is obtained from Equation (27).

### 2.3. Path Parameterization and Constraints

To achieve robust trajectory generation, it is essential to separate spatial path representation from temporal execution parameters. Arc-length parameterization accomplishes this by establishing a normalized, dimensionless coordinate system for motion planning. By transforming the physical path length into a unitary parameter space u ϵ [0,1], this approach enables consistent application of kinematic constraints (velocity and acceleration) across diverse path geometries. The normalization process gives the following relationship:

(28) $$u=\frac{s}{s_{m}}$$

In Equation (28), ‘s’ stands for cumulative arc length, $s_{m}$ stands for total path length, and u corresponds to the normalized dimensionless path parameter in the unit domain. To ensure smooth trajectory generation, the Cartesian coordinates $\left(x\left(u\right),\;y\left(u\right)\right)$ are interpolated using periodic cubic splines, which maintain $C^{2}$ continuity for continuous position, velocity, and acceleration profiles. The mathematical implementation is as follows:

(29) $$\left\{\begin{array}{cc} x\left(u\right) & =\mathrm{csape}(u,x{,}^{\prime }\mathrm{periodi}c^{\prime })\\ y\left(u\right) & =\;\mathrm{csape}(u,y{,}^{\prime }\mathrm{periodi}c^{\prime }) \end{array}\right.$$

where ‘csape’ represents the cubic spline interpolation function implementing periodic boundary conditions. The path curvature k(u), defined as the reciprocal of the radius of curvature, is computed as:

(30) $$k\left(u\right)=\frac{\left|x^{\prime }y^{\prime \prime }-y^{\prime }x^{\prime \prime }\right|}{{{(x}^{\prime 2}+y^{\prime 2})}^{3/2}}$$

In the equations above, the prime notation (‘) denotes differentiation with respect to the normalized path parameter u. The curvature k(u) directly yields the radius of curvature through the reciprocal relationship $\rho \left(u\right)=1/k(u)$. The curvature calculation plays an important role in feedrate optimization, as it governs centripetal acceleration during toolpath execution and determines velocity constraints.

### 2.4. Feedrate Profile Generation

The objective of feedrate profiling is to generate an optimal displacement trajectory that precisely guides the cutting tool during machining operations. The feedrate profiling process requires careful feedrate regulation to minimize vibration and cycle time. Achieving this goal contributes to maintaining accuracy and surface quality, alongside other critical factors such as tool geometry, spindle speed, depth of cut, workpiece material properties, and machine tool dynamics. Achieving this demands an understanding of CNC machining dynamics, particularly the motion characteristics of drive motors. Additionally, to enable smooth cutting operations, the tool must perform controlled acceleration from its initial position and progressive deceleration at path termination. These coordinated axis movements enable accurate machining. It can also minimize mechanical stress, which enhances both process efficiency and machine durability.

**Figure 3:**
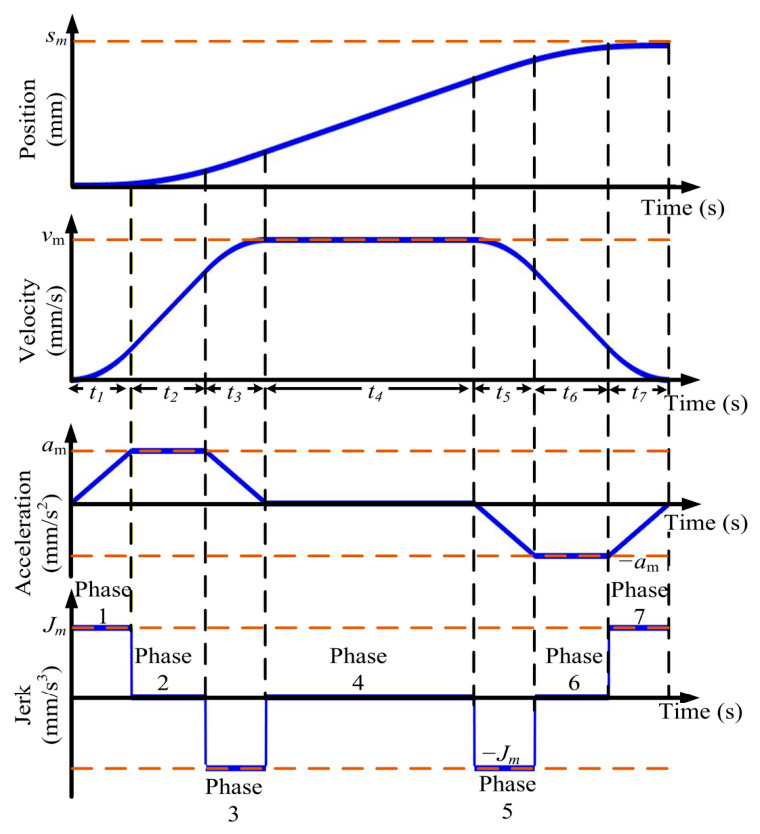
The Kinematics profiles of the jerk-limited S-type feedrate profile.

The study examines an S-curve velocity profile composed of seven distinct kinematic phases. Figure 3 illustrates the jerk-limited feedrate profile, which consists of (1) jerk increase, (2) constant acceleration, (3) jerk decrease, (4) coasting, (5) jerk decrease, (6) constant deceleration, and (7) jerk increase. Equation (31) presents the time-optimal velocity profile obtained through a 7-phase jerk-limited S-curve trajectory planning method. This approach guarantees $C^{3}$ continuity (including jerk smoothness) while satisfying all actuator constraints: $\left|J\right|\leq J_{m,}\left|a\right|\leq a_{m}$. The boundary conditions $v\left(0\right)=v\left(1\right)=0,\;a\left(0\right)=a\left(1\right)=0$ are strictly enforced to ensure motion consistency.

(31) $$\left\{\begin{array}{c} Phase1(Jerk+):J(t)=J_{m},a(t)=J_{m}t\\ Phase2(Acc+):J(t)=0,a(t)=a_{m}\\ Phase3(Jerk-):J(t)=-J_{m},a(t)=a_{m}-J_{m}(t-t_{1})\\ Phase4(Acc+):J(t)=0,a(t)=0\\ Phase5(Jerk-):J(t)=-J_{m},a(t)=a_{m}-J_{m}(t-t_{3})\\ Phase6(Acc-):J(t)=0,a(t)=-a_{m}\\ Phase7(Jerk+):J(t)=J_{m},a(t)=-a_{m}+J_{m}(t-t_{5}) \end{array}\right\}$$

While path parameterization ensures $C^{2}$ geometric continuity, the S-curve planner (Equation (31) enforces $C^{3}$- continuity in feedrate, acceleration, and jerk (Figure 3). Equations (32)–(34) show the Cartesian velocity, acceleration and jerk derived via the chain rule.

Equations (32)–(34) facilitate the conversion of the S-curve feedrate profile into Cartesian space for hardware-implementable motion control, fulfilling three essential objectives: (1) generation of axis-specific commands via transformation of normalized parameter-space trajectories (Equation (31)) into physical actuator coordinates; (2) reduction of dynamic errors through analytical decoupling of tangential and centripetal acceleration components (Equation (33)), exposing curvature-dependent inertial forces; and (3) vibration control via quantification of third-order jerk dynamics (Equation (34)), preventing excitation of structural resonances during rapid transients or high-velocity operation.

(32) $$v\left(u\right)=\frac{dp}{du}\cdot \frac{du}{dt}={\left[x^{\prime }\left(u\right),y^{\prime }\left(u\right)\right]}^{T}\cdot v(u)$$

(33) $$a\left(u\right)={\left[x^{\prime }\left(u\right),y^{\prime }\left(u\right)\right]}^{T}a\left(u\right)+{\left[x^{\prime \prime }\left(u\right),y^{\prime \prime }\left(u\right)\right]}^{T}v{\left(u\right)}^{2}$$

(34) $$J\left(u\right)={\left[x^{\prime }\left(u\right),y^{\prime }\left(u\right)\right]}^{T}J\left(u\right)+{3\left[x^{\prime \prime }\left(u\right),y^{\prime \prime }\left(u\right)\right]}^{T}a\left(u\right)v\left(u\right)+{\left[x^{\prime \prime \prime }\left(u\right),y^{\prime \prime \prime }\left(u\right)\right]}^{T}v(u)\ldots$$

### 2.5. Profilometer Surface Roughness Sensor

Surface roughness measurements were performed using a high-precision profilometer (Haerbin Tools Electrical Company Limited, Harbin, China, model T-1000) configured with a diamond stylus tip, Gaussian filter, measurement range of 2000 μm, evaluation length of 10 mm, drive speed of 0.5 mm/s, sampling length of 0.25 mm, and 3000 sampling points per measurement. The sensor setup was enclosed in a vibration-isolation housing to minimize environmental noise, ensuring compliance with ISO 21920-1:2021 [47].

Three test positions (P_1_, P_2_, P_3_) were selected in high-curvature regions of both the butterfly-shaped and horse-shaped curves to capture the most geometrically challenging sections of the toolpath. Three repetitions were performed per position to ensure statistical reliability.

### 2.6. Experimental Setup

**Figure 4:**
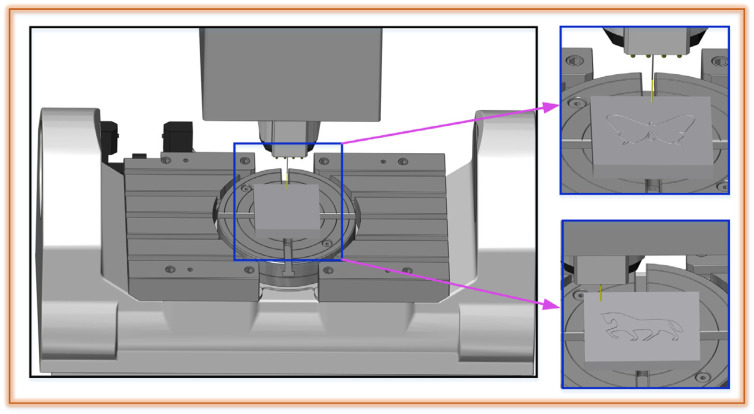
Machining simulation setup.

The experimental validation was conducted on a five-axis CNC machine (X, Y, Z, B, C axes) equipped with a Tyson controller, which provided real-time trajectory interpolation and precise motion control required for implementing the proposed jerk-limited feedrate scheduling algorithm. The B-axis features a tilting mechanism that rotates about the *Y*-axis for precise angular movements, while the C-axis enables full 360° table rotation about the *Z*-axis. All axes are powered by high-performance AC servomotors (model TSM-60-S4P00630LA2, 3000 rpm) with 17-bit absolute rotary encoders (131,072 counts/rev), and the X, Y, Z axes are additionally equipped with 0.5 μm linear encoders. The Tyson controller operates with a closed-loop bandwidth of approximately 200 Hz and a sampling rate of 1 kHz, matching the 1 ms interpolation period used throughout this study. For the X, Y, and Z linear axes, rotational motion from the servomotors is converted to linear displacement via precision-ground ballscrews, providing high stiffness and accurate positioning. The B-axis (tilting mechanism) and C-axis (rotary table) are directly driven through precision gear reducers, enabling accurate angular positioning with minimal backlash. The machining simulation layout employing the five-axis CNC machine is illustrated in Figure 4.

**Figure 5:**
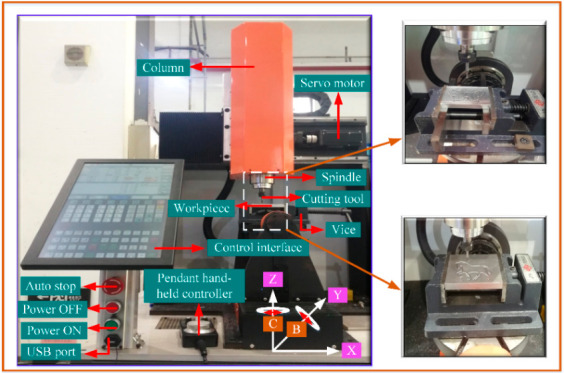
Experimental setup.

Prior to machining, the generated NURBS interpolated points were imported into SolidWorks 2021. The SolidWorks model was then transferred into SolidCAM 2021 to facilitate the machining simulation process. Key CAM settings were configured, including machine selection, geometry definition, tool selection, and machine-level parameter adjustments. This setup was critical to prevent collisions between the tooling and the workpiece during machining. The workpiece material was aluminum alloy 6061, and a 2 mm diameter end milling cutter was used with a depth of cut set to 0.3 mm. After setup, the simulation was conducted, and G-code was generated for the machining experiment. The experimental setup, which closely mirrors the CNC machine configuration used in the simulation, is shown in Figure 5.

**Figure 6:**
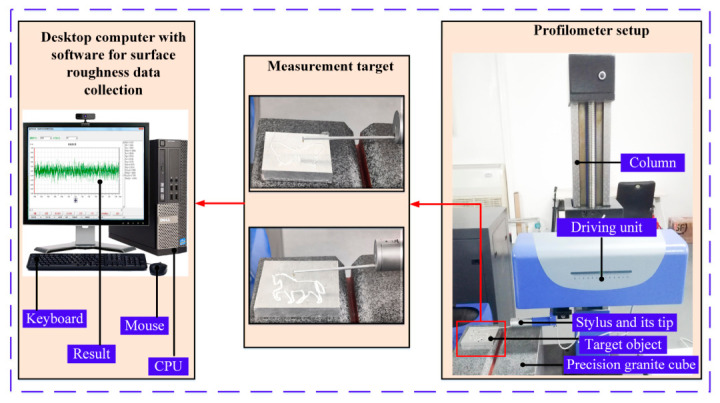
Surface roughness measurement setup.

For the surface roughness measurements, a high-precision profilometer (Haerbin Tools Electrical Company Limited) was employed. The profilometer operates with a diamond stylus tip coupled to a linear variable differential transformer (LVDT) displacement sensor, which converts mechanical displacements induced by surface asperities into electrical signals with nanoscale resolution. The stylus is traversed across the machined workpiece using a motorized drive unit at a controlled speed of 0.5 mm/s. The profilometer was configured with a Gaussian filter, a measurement range of 2000 μm, an evaluation length of 10 mm, a sampling length of 0.25 mm, and 3000 sampling points per measurement. The sensor setup is enclosed in a vibration-isolation housing to minimize environmental noise, ensuring compliance with ISO 21920-1:2021. The surface roughness measurement setup is illustrated in Figure 6.

## 3. Results and Discussion

### 3.1. Numerical Simulation Results

**Figure 7:**
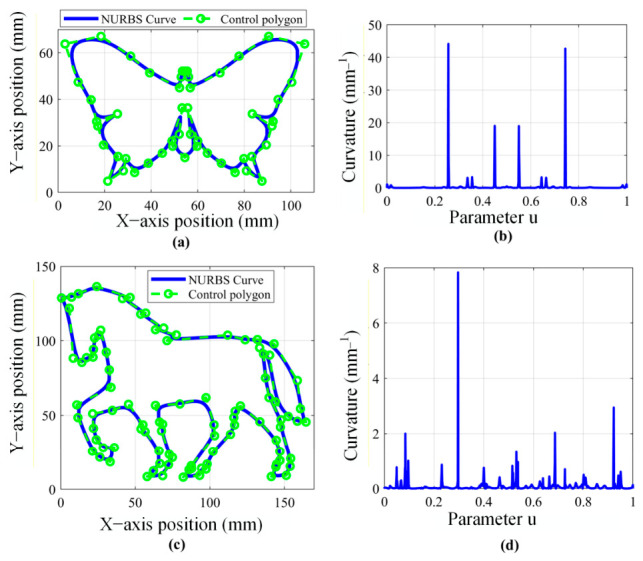
NURBS curves, control polygons and curve curvatures: (**a**) NURBS curve and control polygon of butterfly; (**b**) butterfly curve curvature; (**c**) NURBS curve and control polygon of horse; (**d**) horse curve curvature.

The numerical simulation was conducted in MATLAB (R2024a) using a personal computer with an Intel Core i5-8250U CPU running at 1.8 GHz. Figure 7 shows two representative 2D freeform curves (butterfly and horse) generated by the proposed algorithm, along with their curvature profiles. The input parameters, including control points, knot vectors, and weights, of these curves can be found in the Appendix Section of this paper.

**Figure 8:**
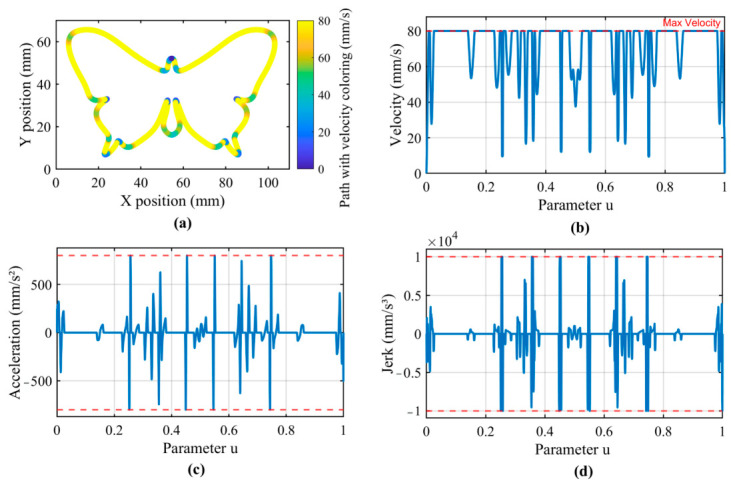
Results of S-curve acceleration and deceleration characteristics of butterfly: (**a**) butterfly feedrate in the path space (**b**) scheduled feedrate; (**c**) acceleration curve; (**d**) jerk curve.

**Figure 9:**
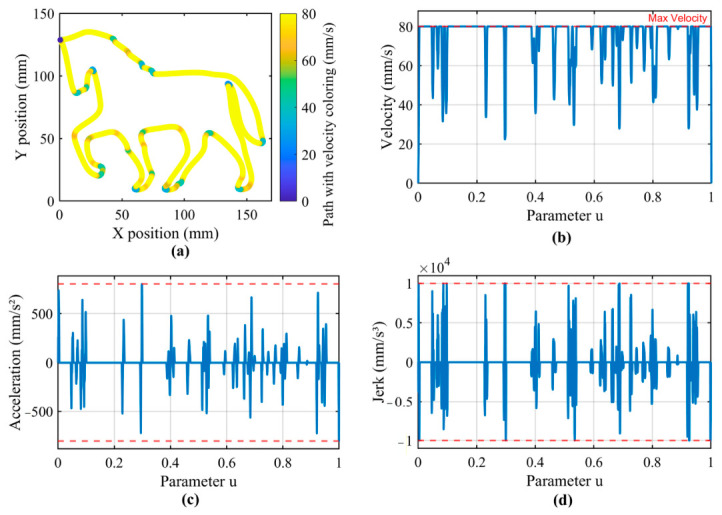
Results of S-curve acceleration and deceleration characteristics of horse: (**a**) horse feedrate in the path space; (**b**) scheduled feedrate; (**c**) acceleration curve; (**d**) jerk curve.

The S-curve acceleration and deceleration characteristics for both the butterfly and horse curves were analyzed to evaluate their motion control performance. The butterfly curve, as shown in Figure 8, exhibits a smooth feedrate profile that reaches the maximum limit of 80 mm/s, adhering to the constraints outlined in Table 1. The feedrate in the path space varies according to the curve’s geometry and the adjustments made to accommodate sharp turns while maintaining precision. The acceleration curve remains within the 800 mm/s^2^ limit, and the jerk curve stays within the limit of 10,000 mm/s^3^. This ensures smooth transitions and minimal mechanical vibrations. Similarly, the horse curve, depicted in Figure 9, follows the same feedrate and acceleration limits but demonstrates slight variations due to its more intricate geometry. The feedrate adapts dynamically along the path, particularly in high-curvature regions, while jerk remains controlled, confirming stable motion execution.

**Figure 10:**
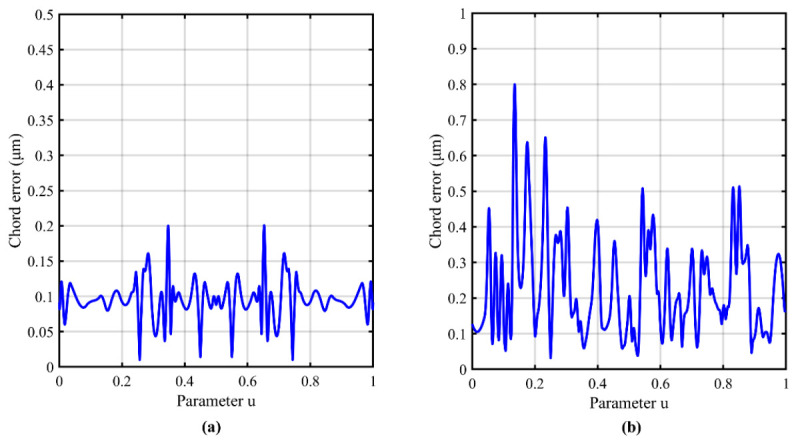
Chord errors: (**a**) butterfly chord error; (**b**) horse chord error.

The simulation constraints and execution times, as presented in Table 1, reveal that both curves achieve the specified feedrate, acceleration, and jerk limits without exceeding the system’s capabilities. As shown in Figure 10, the butterfly curve employs a tighter chord error limit of 0.2 μm compared to the horse curve’s 0.8 μm, indicating a higher precision requirement for its geometry. High-resolution path tracking is guaranteed in both scenarios by the interpolation period of 1 ms. The execution times are nearly identical, with the butterfly curve completing in 0.001104 s and the horse curve in 0.001131 s. This demonstrates the algorithm’s efficiency in handling both simple and complex paths without significant computational overhead.

A comparative analysis of the two curves highlights the adaptability of the motion planning algorithm. The feedrates in path space show greater variability due to their complex geometries. Despite this, they maintained precision within the stringent chord error limit. These findings underscore the robustness of the S-curve acceleration and deceleration profiles in ensuring smooth, precise, and stable motion control for CNC machining and robotic applications.

Figure 8a and Figure 9a show the feedrate distribution in the path space for the butterfly and horse curves, respectively. The color bar provides a clear representation of the feedrate ranges. The figures reveal different patterns in feedrate variation along the toolpaths. In Figure 8a (butterfly curve), the yellow regions, which correspond to higher feedrates, are concentrated along specific segments of the path, particularly in the smoother sections of the curve. This suggests that the machine achieves its maximum feedrates in these areas, where lower directional changes or reduced curvature allow smoother and faster motion.

Similarly, in Figure 9a (the horse curve), the yellow coloring dominates large portions of the path, especially in the elongated, less intricate, or less complex sections. This indicates that the feedrate remains consistently high in these regions, emphasizing the influence of path geometry on velocity performance. The sporadic blue or green segments, representing lower feedrates, coincide with tighter curves or sharp turns where the machine must decelerate to maintain precision.

The prevalence of yellow in both figures highlights that the maximum feedrates are not only attained but also sustained over significant portions of the toolpaths. This observation shows the efficiency of the motion planning system in optimizing feedrate for smoother and less complex segments. The system adapts dynamically to more challenging geometries. The contrast in color distribution between the two curves also reflects their inherent geometric differences. The analysis demonstrates a strong correlation between path geometry and feedrate performance, with the system effectively using smoother segments to maximize velocity and ensure stability in high-curvature regions.

While the numerical simulations were performed in MATLAB on a consumer-grade CPU (Intel Core i5-8250U, 1.8 GHz), the interpolation algorithm itself is computationally lightweight. It requires only 4170 steps for the butterfly curve and 4935 steps for the horse curve, with each step involving a small number of arithmetic operations. The Tyson CNC controller used in the experiments operates with a 1 ms interpolation period and is equipped with a dedicated motion control processor capable of executing the interpolation in real time. Real-time feasibility was assessed by monitoring the controller buffer utilization during machining. The buffer never fell below 80% occupancy, providing indirect evidence that the computational load is manageable. However, buffer utilization is a qualitative indicator; a formal timing analysis measuring the worst-case execution time per interpolation step on the Tyson controller is recommended for future work to conclusively demonstrate real-time compliance with the 1 ms sampling period. The MATLAB execution time, while not directly transferable as an absolute time on the embedded platform, is indicative of the computational efficiency of the algorithm.

### 3.2. Machining Simulation and Experimental Results

**Figure 11:**
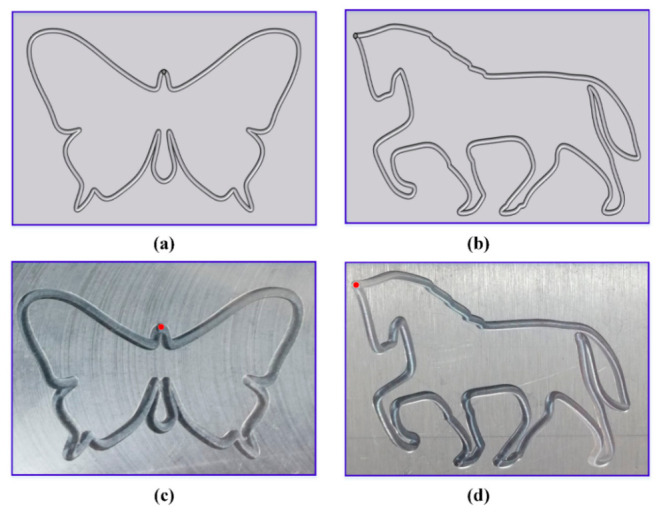
Machining simulation and experimental results: (**a**) simulation curve of butterfly shape; (**b**) simulation curve of horse shape; (**c**) experimental curve of butterfly shape; (**d**) experimental curve of horse shape.

The machining experiments were conducted using the experimental setup described in Section 2.6. The experiments used the interpolated points generated by the proposed Galerkin-NURBS interpolation algorithm. Table 2 summarizes the constraints applied during the experiments, and Figure 11 presents the machining simulation and experimental results. In these figures, the black and red dots indicate the starting and ending positions of the cutting tool, demonstrating consistency between simulation and experimental outcomes. Table 3 presents the G-code data for both the butterfly and horse curves.

The control points, knots, and weights for both the butterfly and the horse curve are provided in Appendix A and Appendix B. The complete G-code files for both the butterfly and the horse curve are provided in the Supplementary Materials (File S1 for butterfly and File S2 for horse curve). The butterfly’s control points, knots, and weights are commonly used by many researchers and can be found in most cited works within this study. The experiments employed strict constraints to ensure precision and efficiency: the feedrate limit ($v_{m}=80\;mm/s$) balanced speed and accuracy, while the acceleration limit ($a_{m}=800{mms}^{-2}$) prevented abrupt tool movements. Jerk was capped at $J_{m}=10,000\;{mms}^{-3}$ to minimize vibrations, and chord error tolerances ($\delta_{m}=0.2\;\mu m$ for the butterfly curve, 0.8 μm for the horse curve) were tailored to each shape’s geometric complexity. The machining times of 3.091 s and 3.483 s were obtained for the butterfly curve and horse curve, respectively.

### 3.3. Comparative Analysis of Processing Performance

To evaluate the effectiveness of the proposed feedrate scheduling method, its performance was compared with existing approaches. The comparison was based on the CQSF (cubic and quartic S-shaped feedrate) method and other approaches discussed in [12]. The butterfly curve was selected as the reference geometry for this comparative analysis, as it is widely used for comparison in this type of study. The evaluation focused on five key performance indicators: velocity (feedrate), acceleration, jerk, processing time, and interpolation steps. These measures directly represent computational efficiency and real-time interpolation feasibility.

The proposed method demonstrates superior performance with a 32.9% reduction in processing time (3.091 s vs. 4.61 s for CQSF), a 40.3% reduction vs. OSQF (5.18 s), and a 51.9% reduction vs. FSRC (6.43 s). Additionally, it achieves a 35.1% reduction in interpolation steps (4170 vs. 6427 for FSRC) and produces $R_{a}$ values between 0.1271 μm and 0.2009 μm, with the majority compliant with ISO 21920-1:2021 and the maximum value at the standard’s upper limit.

**Figure 12:**
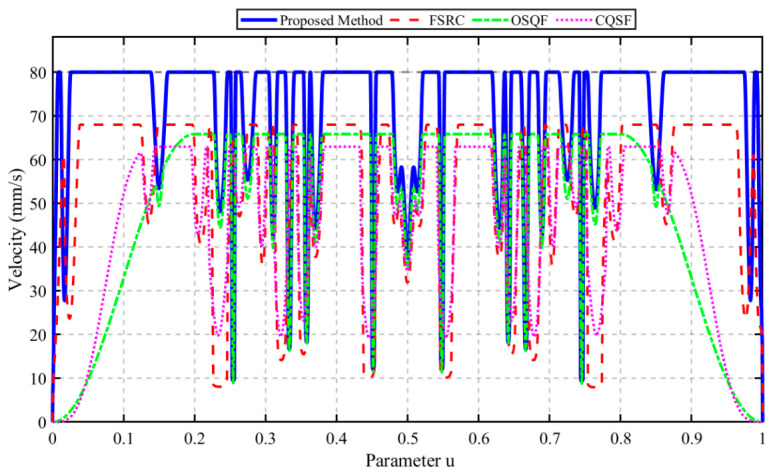
Velocity profile comparison.

**Figure 13:**
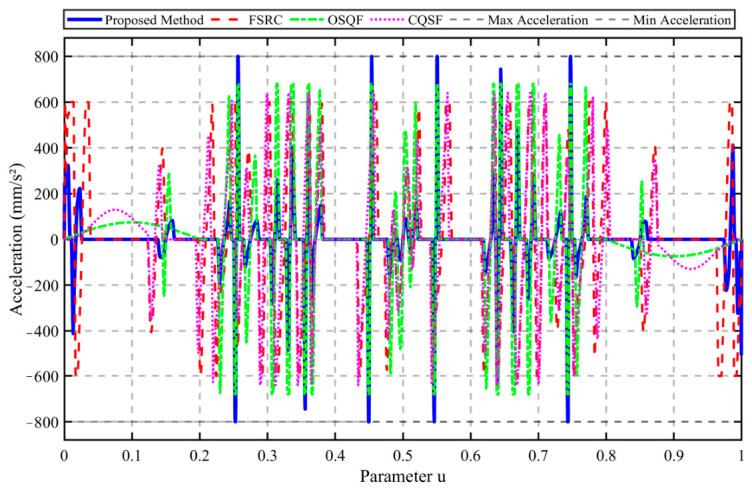
Acceleration profile comparison.

**Figure 14:**
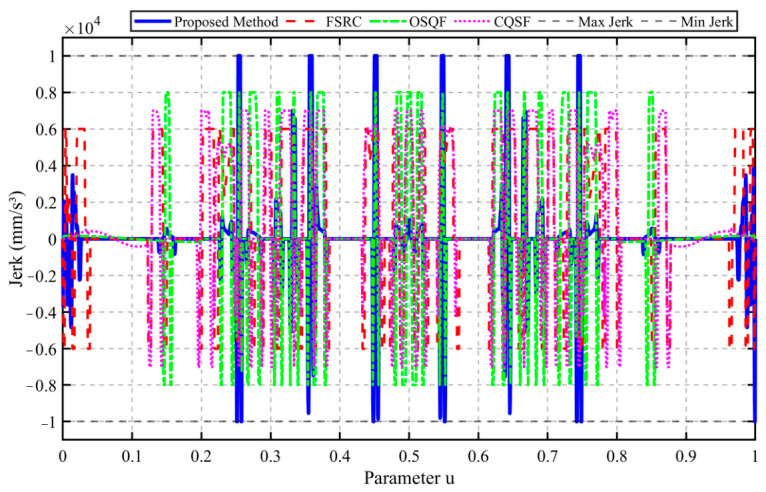
Jerk profile comparison.

Figure 12, Figure 13 and Figure 14 show the comparative analysis of trajectory planning methods. Based on the figures, the proposed method demonstrates superior performance across all kinematic metrics. In terms of velocity (as shown in Figure 12), the proposed method achieves the maximum allowable speed of 80 mm/s (100% of the constraint). It outperforms FSRC (85%), OSQF (82.3%), and CQSF (78.7%). The proposed method achieves the specified maximum speed of 80 mm/s reaching 100% of the velocity constraint. In Figure 13, the comparison methods all operate at reduced levels, with FSRC at 75%, OSQF at 85%, and CQSF at 80%. Most notably, the jerk performance demonstrates the proposed method’s optimal motion smoothness, utilizing 100% of the jerk constraint ($10,000\;mm/s^{3}$) compared to FSRC (60%), OSQF (80%), and CQSF (70%). These results demonstrate that the proposed method achieves optimal time efficiency through maximum velocity utilization. It maintains superior motion smoothness and constraint satisfaction compared to existing approaches.

The results, summarized in Table 4, show the improvements achieved. The proposed method demonstrates a reduction in processing time compared to other techniques. It achieves a 32.9% reduction (3.091 s vs. CQSF’s 4.61 s) in total machining duration. Compared to other methods, the improvements are even more pronounced: the proposed method achieved 51.9% faster than FSRC and 40.3% faster than OSQF. The reduction in processing time shows the method’s ability to optimize feedrate scheduling, minimizing unnecessary decelerations and maintaining smooth motion transitions. The efficiency gains can be attributed to the method’s adaptive feedrate planning, which dynamically adjusts velocity based on path curvature and kinematic constraints, reducing idle time and maximizing machining productivity.

The proposed method achieves the lowest processing time and fewest interpolation steps for two reasons: the Galerkin-optimized NURBS parameterization reduces geometric errors, enabling smoother feedrate scheduling, and the jerk-limited S-curve planner fully utilizes kinematic limits, minimizing unnecessary decelerations.

Beyond processing time, the proposed method also excels in computational efficiency, as evidenced by the reduced number of interpolation steps required. With only 4170 steps needed to complete the toolpath, the method achieves a 9.5% reduction compared to CQSF, a 19.4% reduction over OSQF, and a 35.1% reduction relative to FSRC. Fewer interpolation steps imply lower computational overhead, enhancing the feasibility of real-time implementation in high-speed CNC systems. This is especially important for intricate toolpaths, where too many interpolation points can slow down buffer processing and reduce system responsiveness, while too few points can lead to machine vibration.

**Figure 15:**
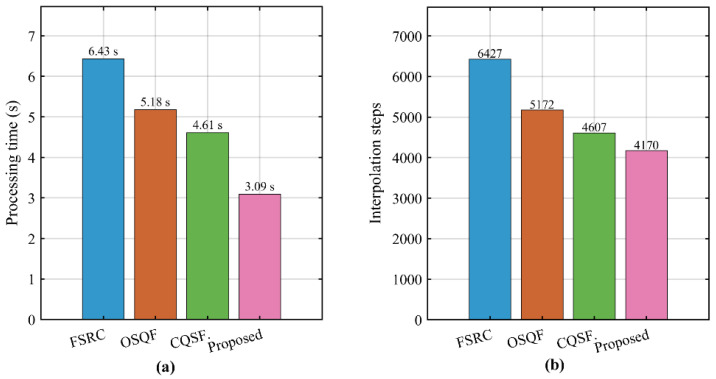
Visual comparison of processing time (**a**) and interpolation steps (**b**) corresponding to the numerical data in Table 4.

The graphical representation of the comparative results is provided in Figure 15. The figure contrasts the proposed method’s performance with that of CQSF, FSRC, and OSQF. The figure shows the proposed method’s efficiency in both processing time and interpolation steps. This reinforces the proposed method’s advantages in high-speed precision machining applications.

The demonstrated improvements in processing time and interpolation efficiency highlight the method’s suitability for real-time CNC applications, where rapid computation and minimal delays are essential. By reducing unnecessary computational load and maintaining trajectory accuracy, the proposed approach ensures smoother motion execution, higher throughput, and improved surface finish, which are the key requirements in modern manufacturing.

### 3.4. Ablation Study

To isolate the individual contributions of the two main components of the proposed method, namely the Galerkin-NURBS interpolation and the jerk-limited S-curve trajectory planner, an ablation study was conducted using the butterfly curve under identical simulation parameters. Four configurations were evaluated:

(1) Configuration A: Standard NURBS interpolation combined with the jerk-limited S-curve trajectory planner (Galerkin projection disabled, S-curve enabled). This configuration isolates the contribution of the S-curve planner without the Galerkin-optimized parameterization.
(2) Configuration B: Galerkin-NURBS interpolation combined with a standard trapezoidal feedrate profile (Galerkin enabled, S-curve disabled). This configuration isolates the contribution of the Galerkin projection without the jerk-limited S-curve scheduling.
(3) Configuration C: Standard NURBS interpolation combined with a standard trapezoidal feedrate profile (neither component active). This represents the baseline system without either enhancement.
(4) Configuration D: The proposed full method combining Galerkin-NURBS interpolation with the jerk-limited S-curve trajectory planner (both components active).

The results of the ablation study are summarized in Table 5.

The results demonstrate that the Galerkin projection alone contributes approximately 15.4% reduction in processing time compared to the baseline (C), while the S-curve planner alone contributes approximately 8.9% reduction. The proposed full method (D) achieves a 27.6% reduction in processing time compared to the baseline (C) and a 20.6% reduction compared to Configuration A. This indicates a synergistic effect when both components are combined, as the improvement of the full method (27.6%) exceeds the individual contributions (15.4% + 8.9% = 24.3%).

In terms of interpolation steps, the Galerkin projection reduces the number of steps by 21.9% (from 5823 to 4550), while the S-curve planner contributes a reduction of 13.9% (from 5823 to 5012). The proposed full method achieves 4170 steps, representing a 28.4% reduction compared to the baseline (C) and a 16.8% reduction compared to Configuration A. These findings confirm that both the Galerkin-NURBS interpolation and the jerk-limited S-curve trajectory planner contribute meaningfully to the overall performance improvements, and their combined effect yields the best results.

The proposed full method achieves a 20.6% reduction in processing time compared to Configuration A and a 14.5% reduction compared to Configuration B. The total reduction of 32.9% is relative to the CQSF baseline (4.61 s) reported in Table 4, while the reduction relative to the FSRC baseline (6.43 s) is 51.9%.

## 4. Surface Roughness Test

### 4.1. Sensor-Based Measurement and Validation Framework

The experimental validation of the proposed Galerkin-NURBS interpolation method relies on two complementary sensor systems that together establish a complete measurement-to-control loop. The first system consists of the integrated rotary and linear encoders embedded in the five-axis CNC machine, which provide real-time closed-loop feedback during machining. The second system is the profilometer-based surface roughness sensor, which performs post-machining quality assessment. This dual-sensor architecture enables a comprehensive validation framework: the encoders verify that the kinematic constraints (feedrate, acceleration, and jerk) are satisfied in real time during trajectory execution, while the profilometer quantifies the resulting surface quality.

The synergy between these systems ensures that improvements in machining efficiency are not achieved at the expense of surface finish. This arrangement allows for rigorous, ISO 21920-1:2021-compliant surface finish validation, directly linking machining parameters to final product quality. The experimental setup for the profilometer measurements is described in Section 2.6, and the measurement results are presented in Section 4.4.

### 4.2. Integrated Position and Orientation Sensors in the Five-Axis CNC Machine

The five-axis CNC machine used in this study is equipped with high-performance AC servomotors that integrate two classes of position sensors. The rotary encoders provide angular position sensing for the B-axis, which rotates about the *Y*-axis, and the C-axis, which rotates 360° about the *Z*-axis. The linear encoders provide Cartesian position sensing for the X, Y, and Z axes. These encoders deliver real-time closed-loop feedback at the interpolation period of 1 ms, enabling precise execution of the G-code toolpaths generated from the NURBS interpolated points. Beyond their primary role in position control, the encoder-derived position data were also used to indirectly sense jerk and acceleration through time-domain differentiation. This allowed real-time verification that the kinematic constraints (namely feedrate, acceleration, and jerk limits) were strictly satisfied throughout the machining process. The encoder feedback thus serves a dual purpose: it ensures accurate trajectory tracking and provides validation data for the proposed motion planning algorithm.

### 4.3. Profilometer-Based Surface Roughness Sensing Setup

**Figure 16:**
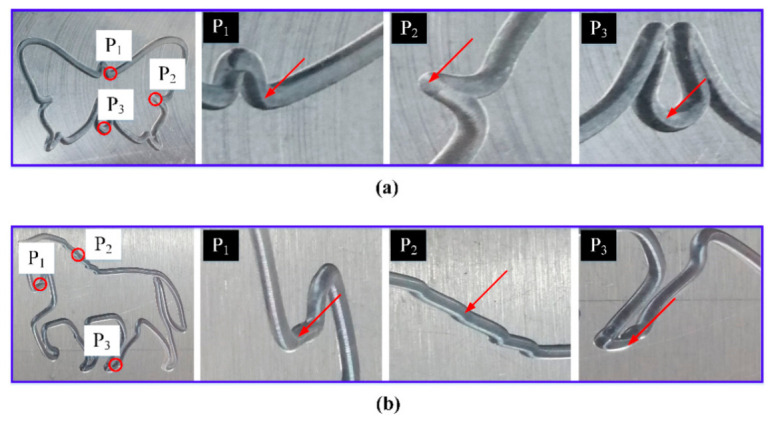
The machining results and the tested areas: (**a**) butterfly-shaped curve; (**b**) horse-shaped curve.

To quantify the surface quality achieved by the proposed method, a high-precision profilometer surface roughness tester was employed as the primary post-machining measurement sensor. The instrument operates using a diamond stylus tip that traverses the machined surface, coupled with a linear variable differential transformer (LVDT) displacement sensor that converts mechanical displacement into electrical signals with nanoscale resolution. The profilometer measurements serve two critical purposes in this study. First, they provide direct quantitative evidence of surface finish quality, confirming that the improved machining efficiency does not degrade surface integrity. Second, they validate the practical viability of the proposed method for precision manufacturing applications where surface finish requirements are stringent, such as aerospace and medical component machining. As shown in Figure 16, three test positions (P_1_, P_2_, P_3_) were selected in high-curvature regions of both the butterfly-shaped and horse-shaped curves to capture the most geometrically challenging sections of the toolpath, with three repetitions performed per position to ensure statistical reliability. The measurement parameters and test positions are detailed in Section 2.6. The measured roughness values are presented and discussed in Section 4.4.

### 4.4. Surface Roughness and Contour Verification Results

The measured $R_{a}$ values (Table 6) ranged from 0.1271 μm to 0.2009 μm, demonstrating that the proposed Galerkin-NURBS interpolation method, guided by encoder feedback during machining and validated by profilometer sensing post-machining, achieves surface finishes at or near the ISO 21920-1:2021 high-precision threshold.

**Figure 17:**
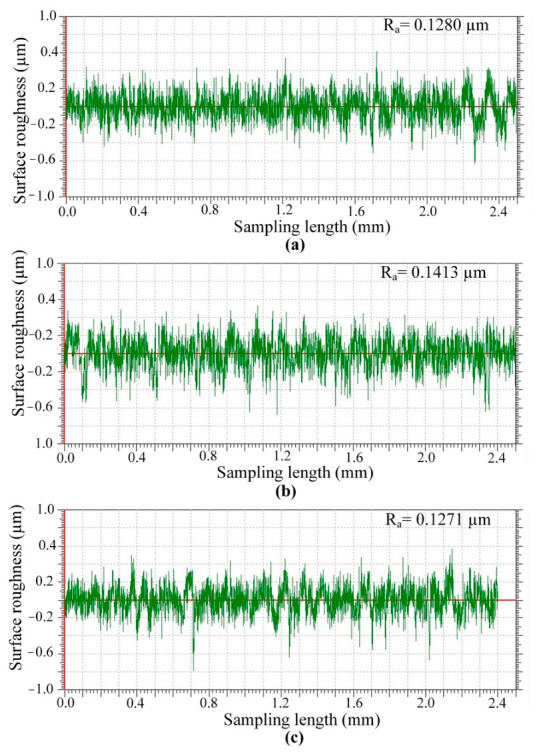
Surface roughness results of butterfly-shaped curve: (**a**) result of P_1_; (**b**) result of P_2_; (**c**) result of P_3_.

**Figure 18:**
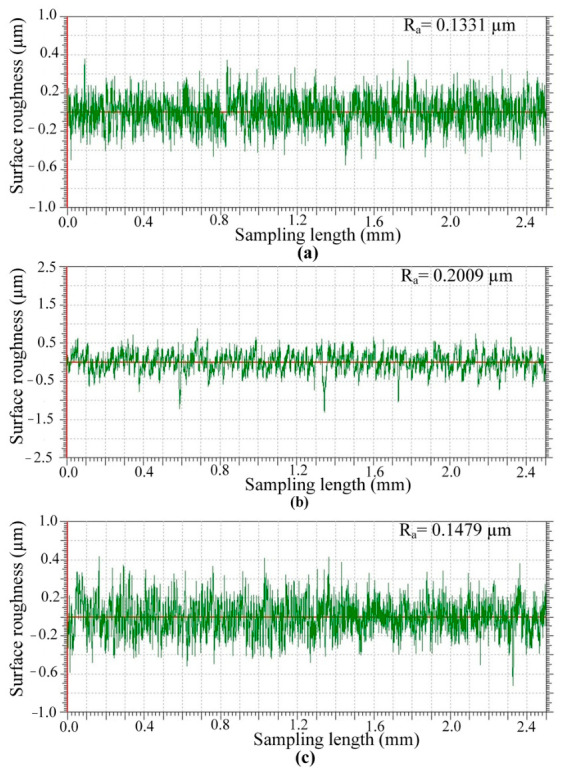
Surface roughness results of horse-shaped curve: (**a**) result of P_1_; (**b**) result of P_2_; (**c**) result of P_3_.

To validate the precision of the machined contours, the physical contours of both the horse-shaped and butterfly-shaped curves were measured and compared with their designed counterparts. The machined contours exhibited negligible deviations, with maximum errors confined to ±2 μm in high-curvature regions (as shown in Figure 17 and Figure 18), confirming the geometric fidelity of the proposed interpolation method. These results confirm that the complete sensing loop, consisting of encoders for real-time trajectory control and the profilometer for surface quality verification, provides rigorous experimental evidence of the effectiveness of the method.

Contour deviation was measured using a Coordinate Measuring Machine (CMM) (Hexagon Global Performance 7.10.7) equipped with a Renishaw SP25M scanning probe. The machined workpiece was mounted on the CMM table, and the probe traversed along the machined contour at a scanning speed of 5 mm/s with a point spacing of 10 μm. The measured point cloud was then compared to the nominal NURBS curve using a best-fit alignment algorithm (least-squares minimization) in the evaluation software of the CMM. The maximum deviation across all measured points was recorded as the contour error. Three independent measurement runs were performed per curve, and the maximum deviation was consistently confined to ±2 μm in high-curvature regions.

To assess the repeatability of the surface roughness measurements, the standard deviation (SD) and 95% confidence intervals (CI) were computed for the three repetitions performed at each test position using the t-distribution with n=3 replicates $t_{\left(0.025,2\right)}=4.303$. The corrected confidence intervals, calculated as mean $\pm t_{\left(0.025,2\right)}\times SD/\surd 3$, are presented in Table 6. The small standard deviations, ranging from 0.0001 μm to 0.0020 μm, confirm the high repeatability and consistency of the profilometer measurements across all test positions. The corresponding 95% confidence intervals provide a statistically rigorous estimate of the measurement precision. For example, at Butterfly $P_{1}$, the mean $R_{a}$ of 0.1280 μm has a 95% CI of (0.1230, 0.1330) μm, indicating that the true surface roughness at this position is expected to lie within this range with 95% confidence.

It should be noted that the surface roughness measurements presented in this study were obtained only for the proposed method. Comparative $R_{a}$ data for FSRC, OSQF, and CQSF were not available, as these methods were not re-implemented experimentally in the laboratory. The primary purpose of the surface roughness measurements is to verify that the improvements in processing time and interpolation efficiency achieved by the proposed method were not accompanied by a degradation in surface quality. While the measured $R_{a}$ values (0.1271–0.2009 μm) demonstrate compliance with ISO 21920-1:2021, direct surface finish comparisons with existing methods are not made.

According to ISO 21920-1:2021, the recommended $R_{a}$ limit for aluminum alloy 6061 under high-precision finish machining conditions is 0.2 μm. The measured $R_{a}$ values ranged from 0.1271 μm to 0.2009 μm. While most measurements fall below this threshold, the measurement at Horse $P_{2}$ (0.2009 μm) reaches the upper bound of the standard. Overall, the results demonstrate that the proposed method achieves surface quality at or near the high-precision standard, with $P_{2}$ representing the limiting case that merits further optimization in future work.

The measured contour deviations within ±2 μm confirm that the Galerkin-NURBS interpolation framework successfully maintains geometric fidelity even in high-curvature regions where traditional interpolation methods typically exhibit larger errors. This level of accuracy is attributed to the curvature-adaptive feedrate optimization, which reduces feedrate in tight corners to minimize chord error, while allowing higher speeds in straight segments. The tight tolerance achieved is particularly significant for aerospace and medical applications where contour accuracy directly affects part functionality and assembly fit. Furthermore, the consistency of the deviation across all three measurement runs indicates that the machining process was stable and free from chatter or other dynamic instabilities that could compromise geometric accuracy. These results validate that the proposed interpolation method effectively balances speed and precision without sacrificing one for the other.

The measured surface roughness values confirm compliance with the ISO 21920-1:2021 standard, with $R_{a}$ values ranging from 0.1271 μm to 0.2009 μm. The low standard deviations (0.0001–0.0020 μm) and narrow 95% confidence intervals across all measurement positions confirm the high repeatability and statistical reliability of the profilometer measurements. This consistency across multiple repetitions and positions demonstrates that the proposed method produces uniform surface quality regardless of the geometric complexity of the toolpath. The smooth jerk-limited feedrate profile, which enforces $C^{3}$ continuity, effectively minimizes the dynamic force variations that typically lead to surface defects such as tool marks and feed marks. These results, combined with the geometric accuracy demonstrated by the CMM measurements, confirm that the proposed method achieves a favorable balance between machining efficiency, geometric precision, and surface quality.

## 5. Conclusions

This study developed a Galerkin-NURBS interpolation framework coupled with jerk-limited S-curve trajectory planning for high-speed machining of complex freeform geometries, addressing key challenges in precision, efficiency, and motion smoothness. The proposed method optimizes NURBS parameterization through Galerkin projection, reducing geometric approximation errors in high-curvature regions, while the S-curve planner enforces $C^{3}$ continuity to minimize vibrations and ensure smooth motion. Numerical simulations and machining experiments on butterfly and horse curves validated the ability of the algorithm to maintain precision under tight chord error constraints while adhering to kinematic limits.

Experimental results on a five-axis CNC machine demonstrated that the proposed method achieves reductions in processing time and interpolation steps compared to existing methods. It also maintains surface roughness values at or near the ISO 21920-1:2021 high-precision threshold ($R_{a}$ ≤ 0.2 μm), with 5 of 6 measurement positions falling below the limit and one position (0.2009 μm) at the upper bound. The method fully utilizes the velocity, acceleration, and jerk constraints, confirming optimal motion smoothness. Physical contour measurements confirmed geometric fidelity, with deviations confined to ±2 μm. The proposed method is best suited for applications where geometric accuracy and kinematic smoothness are critical, particularly in high-curvature regions.

Despite the achievements of this study, some limitations should be acknowledged. The validation is limited to two planar freeform geometries, and extension to 3D surfaces remains unexplored. While the measured $R_{a}$ values (0.1271–0.2009 μm) demonstrate compliance with ISO 21920-1:2021, direct surface finish comparisons with existing methods are not made. The comparative analysis against FSRC, OSQF, and CQSF is based on published data rather than independent hardware re-implementation.

Future work should extend the validation to industrially relevant 3D geometries such as turbine blades and impeller profiles, incorporate adaptive constraint handling for varying material properties, and perform rigorous real-time implementation and timing analysis on commercial CNC controllers. Direct comparative surface roughness measurements and repeated experimental runs are also recommended to further strengthen the validation.

## Figures and Tables

**Table 1:** Simulation constraints and their execution time.

Parameter	Symbol	Unit	Butterfly Curve	Horse Curve
Feedrate limit	$v_{m}$	${mms}^{-1}$	80	80
Acceleration limit	$a_{m}$	${mms}^{-2}$	800	800
Jerk limit	$J_{m}$	${mms}^{-3}$	10,000	10,000
Chord error limit	$\delta_{m}$	μm	0.2	0.8
Interpolation period	T	ms	1	1
Average execution time	$T_{E}$	s	0.001104	0.001131

**Table 2:** The experimental constraints used and the observed processing time.

Parameter	Symbol	Unit	Butterfly Curve	Horse Curve
Feedrate limit	$v_{m}$	${mms}^{-1}$	80	80
Acceleration limit	$a_{m}$	${mms}^{-2}$	800	800
Jerk limit	$J_{m}$	${mms}^{-3}$	10,000	10,000
Chord error limit	$\delta_{m}$	μm	0.2	0.8
Processing time	T	s	3.091	3.483

**Table 3:** Butterfly and horse curve G-code.

Butterfly G-Code			Horse G-Code		
Line	X-Coordinate (mm)	Y-Coordinate (mm)	Line	X-Coordinate (mm)	Y-Coordinate (mm)
N112	54.4930	52.1390	N112	0.5600	128.7500
N122	54.6642	52.1249	N122	0.6356	128.7560
N124	54.7446	52.1076	N124	0.7109	128.7622
N126	54.8218	52.0837	N126	0.7859	128.7684
N128	54.8958	52.0535	N128	0.8607	128.7748
N130	54.9667	52.0172	N130	0.9353	128.7813
N132	55.0346	51.9749	N132	1.0096	128.7879
N134	55.0996	51.9270	N134	1.0836	128.7946
N136	55.1617	51.8736	N136	1.1574	128.8014
N138	55.2212	51.8149	N138	1.2310	128.8083
N140	55.2780	51.7511	N140	1.3043	128.8154
N142	55.3323	51.6825	N142	1.3774	128.8226
N144	55.3842	51.6094	N144	1.4503	128.8299
N146	55.4337	51.5318	N146	1.5229	128.8373
N148	55.4810	51.4502	N148	1.5953	128.8448
N150	55.5260	51.3646	N150	1.6675	128.8525
N152	55.5691	51.2753	N152	1.7394	128.8602
:	:	:	:	:	:
N8340	54.4930	52.1390	N13456	0.5600	128.7500

**Table 4:** Comparison of processing time and interpolation steps.

Methods	Processing Time (s)	Interpolation Steps
FSRC	6.43	6427
OSQF	5.18	5172
CQSF	4.61	4607
Proposed method	3.091	4170

**Table 5:** Ablation study results for the butterfly curve.

Configuration	Galerkin-NURBS	Jerk-Limited S-Curve	Processing Time (s)	Interpolation Steps	Reduction vs. Baseline
A	✗	✓	3.893	5012	-
B	✓	✗	3.614	4550	7.2% (time)/9.2% (steps)
C	✗	✗	4.271	5823	-
D	✓	✓	3.091	4170	20.6% (time)/16.8% (steps) vs. A

***Note:** Configuration C (standard NURBS + trapezoidal) serves as the true baseline representing neither enhancement active. Configuration A (standard NURBS + S-curve) shows the S-curve contribution alone (8.9% reduction* vs. *C). Configuration B (Galerkin-NURBS + trapezoidal) shows the Galerkin contribution alone (15.4% reduction* vs. *C). Configuration D (proposed full method) shows the combined effect (27.6% reduction* vs. *C, 20.6% reduction* vs. *A, 14.5% reduction* vs. *B)*.

**Table 6:** Surface roughness test parameters and $R_{a}$ values.

Test Curve	Position	$R_{a1}$ (μm)	$R_{a2}$ (μm)	$R_{a3}$ (μm)	Mean $R_{a}$ (μm)	SD (μm)	95% CI (μm)
Butterfly	$P_{1}$	0.1300	0.1260	0.1280	0.1280	0.0020	0.1260–0.1300
Butterfly	$P_{2}$	0.1400	0.1420	0.1419	0.1413	0.0010	0.1403–0.1423
Butterfly	$P_{3}$	0.1260	0.1280	0.1273	0.1271	0.0010	0.1261–0.1281
Horse	$P_{1}$	0.1335	0.1327	0.1331	0.1331	0.0004	0.1327–0.1335
Horse	$P_{2}$	0.2010	0.2008	0.2009	0.2009	0.0001	0.2008–0.2010
Horse	$P_{3}$	0.1480	0.1478	0.1479	0.1479	0.0001	0.1478–0.1480

## Data Availability

All data generated or analyzed during this study are included in this article.

## Supplementary Materials

The following supporting information can be downloaded at: https://www.mdpi.com/article/10.3390/s26144441/s1.

## Appendix A. Butterfly-Shaped NURBS Curve

### Appendix A.1. Control Points (mm)

{54.493, 52.139}, {55.507, 52.139}, {56.082, 49.615}, {56.780, 44.971}, {69.575, 51.358}, {77.786, 58.573}, {90.526, 67.081}, {105.973, 63.801}, {100.400, 47.326}, {94.567, 39.913}, {92.369, 30.485}, {83.440, 33.757}, {91.892, 28.509}, {89.444, 20.393}, {83.218, 15.446}, {87.621, 4.830}, {80.945, 9.267}, {79.834, 14.535}, {76.074, 8.522}, {70.183, 12.550}, {64.171, 16.865}, {59.993, 22.122}, {55.680, 36.359}, {56.925, 24.995}, {59.765, 19.828}, {54.493, 14.940}, {49.220, 19.828}, {52.060, 24.994}, {53.305, 36.359}, {48.992, 22.122}, {44.814, 16.865}, {38.802, 12.551}, {32.911, 8.521}, {29.152, 14.535}, {28.040, 9.267}, {21.364, 4.830}, {25.768, 15.447}, {19.539, 20.391}, {17.097, 28.512}, {25.537, 33.750}, {16.602, 30.496}, {14.199, 39.803}, {8.668, 47.408}, {3.000, 63.794}, {18.465, 67.084}, {31.197, 58.572}, {39.411, 51.358}, {52.204, 44.971}, {52.904, 49.614}, {53.478, 52.139}, {54.493, 52.139}.

### Appendix A.2. Knots

{[0, 0, 0, 0, 0.0083, 0.0150, 0.0361, 0.0855, 0.1293, 0.1509, 0.1931, 0.2273, 0.2435, 0.2561, 0.2692, 0.2889, 0.3170, 0.3316, 0.3482, 0.3553, 0.3649, 0.3837, 0.4005, 0.4269, 0.4510, 0.4660, 0.4891, 0.5000, 0.5109, 0.5340, 0.5489, 0.5731, 0.5994, 0.6163, 0.6351, 0.6447, 0.6518, 0.6683, 0.6830, 0.7111, 0.7307, 0.7439, 0.7565, 0.7729, 0.8069, 0.8491, 0.8707, 0.9145, 0.9639, 0.9850, 0.9917, 1, 1, 1, 1]}.

### Appendix A.3. Weights

{[1, 1, 1, 1.2, 1, 1, 1, 1, 1, 1, 1, 2, 1, 1, 5, 3, 1, 1.1, 1, 1, 1, 1, 1, 1, 1, 1, 1, 1, 1, 1, 1, 1, 1, 1.1, 1, 3, 5, 1, 1, 2, 1, 1, 1, 1, 1, 1, 1, 1.2, 1, 1, 1]}.

## Appendix B. Horse-Shaped NURBS Curve

### Appendix B.1. Control Points (mm)

{0.56, 128.75}, {7.29, 129.28}, {11.88, 131.67}, {24.16, 136.43}, {46.53, 129.1}, {41.40, 128.37}, {56.98, 118.65}, {53.68, 117.92}, {69.08, 108.57}, {63.58, 107.47}, {77.52, 103.98}, {71.28, 100.13}, {111.98, 103.80}, {123.72, 100.68}, {131.97, 100.86}, {143.15, 97.93}, {158.74, 73.36}, {160.79, 54.42}, {164.51, 45.47}, {158.74, 46.03}, {152.59, 49.20}, {144.02, 59.07}, {140.11, 90.37}, {133.40, 95.40}, {136.01, 91.12}, {137.32, 75.10}, {139.74, 61.87}, {147.75, 46.96}, {147.37, 42.10}, {154.27, 20.69}, {153.89, 15.48}, {151.29, 9.33}, {141.23, 8.58}, {144.32, 15.10}, {144.77, 16.78}, {147, 19.39}, {147, 25.54}, {144.58, 33.18}, {133.59, 45.66}, {120.55, 56.28}, {117.01, 52.74}, {116.07, 48.45}, {116.63, 43.42}, {113.84, 37.09}, {102.10, 25.54}, {98.19, 16.97}, {97.44, 13.61}, {94.46, 14.17}, {93.16, 9.33}, {82.17, 8.21}, {86.08, 13.24}, {87.76, 13.99}, {87.57, 15.85}, {103.22, 36.16}, {102.85, 43.61}, {97.44, 61.87}, {79.93, 57.58}, {63.54, 56.65}, {66.33, 45.47}, {68.38, 43.98}, {67.63, 39.51}, {72.85, 23.49}, {74.90, 21.44}, {72.29, 16.59}, {68.38, 9.14}, {57.95, 8.58}, {62.23, 12.50}, {66.52, 17.34}, {65.96, 25.91}, {56.64, 38.95}, {53.85, 40.81}, {55.15, 43.61}, {45.46, 57.40}, {34.66, 53.30}, {21.43, 51.06}, {21.99, 41.19}, {23.85, 33.55}, {28.51, 27.96}, {35.96, 28.15}, {33.17, 18.46}, {26.27, 21.63}, {21.24, 25.72}, {11.56, 48.45}, {10.81, 57.02} {33.73, 68.76} {32.61, 80.50}, {30.56, 92.42}, {26.83, 107.14}, {25.53, 103.97}, {22.73, 101.92}, {21.99, 94.28}, {21.69, 89.07}, {17.33, 88.70}, {14.16, 85.53}, {8.39, 88.32}, {5.78, 121.86}, {0.56,128.75}.

### Appendix B.2. Knots

{[0, 0, 0, 0, 0.04, 0.05, 0.06, 0.07, 0.08, 0.09, 0.1, 0.11, 0.12, 0.13, 0.14, 0.15, 0.16, 0.17, 0.18, 0.19, 0.2, 0.21, 0.22, 0.23, 0.24, 0.25, 0.26, 0.27, 0.28, 0.29, 0.3, 0.31, 0.32, 0.33, 0.34, 0.35, 0.36, 0.37, 0.38, 0.39, 0.4, 0.41, 0.42, 0.43, 0.44, 0.45, 0.46, 0.47, 0.48, 0.49, 0.5, 0.51, 0.52, 0.53, 0.54, 0.55, 0.56, 0.57, 0.58, 0.59, 0.6, 0.61, 0.62, 0.63, 0.64, 0.65, 0.66, 0.67, 0.68, 0.69, 0.7, 0.71, 0.72, 0.73, 0.74, 0.75, 0.76, 0.77, 0.78, 0.79, 0.8, 0.81, 0.82, 0.83, 0.84, 0.85, 0.86, 0.87, 0.88, 0.89, 0.9, 0.91, 0.92, 0.93, 0.94, 0.95, 0.96, 1, 1, 1, 1]}.

### Appendix B.3. Weights

{[1, 1, 1, 1, 1, 1, 1, 1, 1, 1, 1, 1, 1, 1, 1, 1, 1, 1, 1, 1, 1, 1, 1, 1, 1, 1, 1, 1, 1, 1, 1, 1, 1, 1, 1, 1, 1, 1, 1, 1, 1, 1, 1, 1, 1, 1, 1, 1, 1, 1, 1, 1, 1, 1, 1, 1, 1, 1, 1, 1, 1, 1, 1, 1, 1, 1, 1, 1, 1, 1, 1, 1, 1, 1, 1, 1, 1, 1, 1, 1, 1, 1, 1, 1, 1, 1, 1, 1, 1, 1, 1, 1, 1, 1, 1, 1, 1,]}.

## References

[1] Erkorkmaz K., Altintas Y. (2001). High Speed CNC System Design. Part I: Jerk Limited Trajectory Generation and Quintic Spline Interpolation. Int. J. Mach. Tools Manuf..

[2] Liang F., Kang C., Lu Z., Fang F. (2021). Iso-Scallop Tool Path Planning for Triangular Mesh Surfaces in Multi-Axis Machining. Robot. Comput. Integr. Manuf..

[3] Wei Z., Wei X. (2025). Scalable Field-Aligned Reparameterization for Trimmed NURBS. Eng. Comput..

[4] Jiang B., Sun R., Li Z.L., Xu L., Liao H., Teng X.Y., Li B. (2025). Local Corner Smoothing Based on Deep Learning for CNC Machine Tools. Sci. Rep..

[5] Farouki R.T., Tsai Y.F. (2001). Exact Taylor Series Coefficients for Variable-Feedrate CNC Curve Interpolators. CAD Comput. Aided Des..

[6] Zhou B., Zhao J., Li L., Xia R. (2016). NURBS Curve Interpolation Algorithm Based on Tool Radius Compensation Method. Int. J. Prod. Res..

[7] Liu X., Yu P., Chen H., Peng B., Wang Z., Liang F. (2025). Feedrate Fluctuation Minimization for NURBS Tool Path Interpolation Based on Arc Length Compensation and Iteration. Micromachines.

[8] Xie Y., Mei S., Zhang C. (2025). Optimisation Decision of Machining Process Parameters Considering Milling Energy Consumption and Specific Cutting Energy. Alex. Eng. J..

[9] Liu H., Liu Q., Sun P., Liu Q., Yuan S. (2017). A Polynomial Equation-Based Interpolation Method of NURBS Tool Path with Minimal Feed Fluctuation for High-Quality Machining. Int. J. Adv. Manuf. Technol..

[10] Heng M., Erkorkmaz K. (2010). Design of a NURBS Interpolator with Minimal Feed Fluctuation and Continuous Feed Modulation Capability. Int. J. Mach. Tools Manuf..

[11] Wu B., Ma J., Wei L., Liao X., Lu J. (2022). NURBS Interpolator with Scheduling Scheme Combining Cubic and Quartic S-Shaped Feedrate Profiles Under Drive and Chord Error Constraints. CAD Comput. Aided Des..

[12] Yau H.T., Kuo M.J. (2001). NURBS Machining and Feed Rate Adjustment for High-Speed Cutting of Complex Sculptured Surfaces. Int. J. Prod. Res..

[13] Zhang G., Gao J., Zhang L., Wang X., Luo Y., Chen X. (2022). Generalised NURBS Interpolator with Nonlinear Feedrate Scheduling and Interpolation Error Compensation. Int. J. Mach. Tools Manuf..

[14] Tsai M.S., Nien H.W., Yau H.T. (2008). Development of an Integrated Look-Ahead Dynamics-Based NURBS Interpolator for High Precision Machinery. CAD Comput. Aided Des..

[15] Shi Z.K., Zhang W.J., Ding Y. (2023). A Local Toolpath Smoothing Method for a Five-Axis Hybrid Machining Robot. Sci. China Technol. Sci..

[16] Yang J.X., Adili A., Ding H. (2023). Real Time Tool Path Smoothing of Short Linear Commands for Robot Manipulator by Constructing Asymmetrical Pythagoran-Hodograph (PH) Splines. Sci. China Technol. Sci..

[17] Lyu H., Liu Y., Guo J.Y., Zhang H.M., Li Z.X. (2019). Tool-Path Generation for Industrial Robotic Surface-Based Application. Adv. Manuf..

[18] Li Y., Liang F.S., Lu L., Fan C. (2023). Improved Time-Optimal B-Spline Feedrate Scheduling for NURBS Tool Paths in CNC Machining. Adv. Manuf..

[19] Jafarzadeh E., Movahhedy M.R., Khodaygan S., Ghorbani M. (2018). Prediction of Machining Chatter in Milling Based on Dynamic FEM Simulations of Chip Formation. Adv. Manuf..

[20] Sun S., Zhao P., Zhang T., Li B., Yu D. (2024). Smoothing Interpolation of Five-Axis Tool Path with Less Feedrate Fluctuation and Higher Computation Efficiency. J. Manuf. Process..

[21] Yang J., Hu Q., Ding H. (2016). A Two-Stage CNC Interpolation Algorithm for Corner Smoothing Trajectories with Geometric Error and Dynamics Constraints. Procedia CIRP.

[22] Baek D.K., Yang S.H., Ko T.J. (2013). Precision NURBS Interpolator Based on Recursive Characteristics of NURBS. Int. J. Adv. Manuf. Technol..

[23] Lei W.T., Sung M.P., Lin L.Y., Huang J.J. (2007). Fast Real-Time NURBS Path Interpolation for CNC Machine Tools. Int. J. Mach. Tools Manuf..

[24] Jiang J., Lin F., Zhang Y., Zhang H., Ye P. (2019). A Real-Time Feedrate Planning Method and Efficient Interpolator with Minimal Feedrate Fluctuation for Parametric Toolpath. IEEE Access.

[25] Chen M., Sun Y. (2019). Contour Error–Bounded Parametric Interpolator with Minimum Feedrate Fluctuation for Five-Axis CNC Machine Tools. Int. J. Adv. Manuf. Technol..

[26] Wang T.Y., Zhang Y.B., Dong J.C., Ke R.J., Ding Y.Y. (2020). NURBS Interpolator with Adaptive Smooth Feedrate Scheduling and Minimal Feedrate Fluctuation. Int. J. Precis. Eng. Manuf..

[27] Han X., Zhu K., Wang X. (2024). A Hash Approach to Refine CNC Computation of Arc Length and Parameter of NURBS with High Efficiency and Precision. Int. J. Precis. Eng. Manuf..

[28] Jeong S.Y., Choi Y.J., Park P. (2006). Parametric Interpolation Using Sampled Data. CAD Comput. Aided Des..

[29] Du X., Huang J., Zhu L.M. (2015). A Complete S-Shape Feed Rate Scheduling Approach for NURBS Interpolator. J. Comput. Des. Eng..

[30] Liang F., Zhao J., Ji S. (2017). An Iterative Feed Rate Scheduling Method with Confined High-Order Constraints in Parametric Interpolation. Int. J. Adv. Manuf. Technol..

[31] Min K., Sun Y., Lee C.H., Hu P., He S. (2019). An Improved B-Spline Fitting Method with Arc-Length Parameterization, G2-Continuous Blending, and Quality Refinement. Int. J. Precis. Eng. Manuf..

[32] Yang Z., Shen L.Y., Yuan C.M., Gao X.S. (2015). Curve Fitting and Optimal Interpolation for CNC Machining under Confined Error Using Quadratic B-Splines. CAD Comput. Aided Des..

[33] Bartoň M., Bizzarri M., Rist F., Sliusarenko O., Pottmann H. (2021). Geometry and Tool Motion Planning for Curvature Adapted CNC Machining. ACM Trans. Graph..

[34] Wang Y., Hu C., Li Z., He Z., Lin S., Wang Y., Lin S., Yu J., Jin Z., Zhu Y. (2025). On the Consistency of Path Smoothing and Trajectory Planning in CNC Machining: A Surface-Centric Evaluation. Robot. Comput. Integr. Manuf..

[35] Xu B., Ding Y., Ji W. (2022). An Interpolation Method Based on Adaptive Smooth Feedrate Scheduling and Parameter Increment Compensation for NURBS Curve. ISA Trans..

[36] Ni H., Zhang C., Chen C., Hu T., Liu Y. (2019). A Parametric Interpolation Method Based on Prediction and Iterative Compensation. Int. J. Adv. Robot. Syst..

[37] Chien S., Sencer B., Ward R. (2023). Accurate Prediction of Machining Cycle Times and Feedrates with Deep Neural Networks Using BiLSTM. J. Manuf. Syst..

[38] Li B., Zhang H., Ye P., Wang J. (2020). Trajectory Smoothing Method Using Reinforcement Learning for Computer Numerical Control Machine Tools. Robot. Comput. Integr. Manuf. J..

[39] Kim H., Okwudire C.E. (2023). Intelligent Feedrate Optimization Using a Physics-Based and Data-Driven Digital Twin. CIRP Ann.—Manuf. Technol..

[40] Jiang Y., Chen J., Zhou H., Yang J., Hu P., Wang J. (2022). Contour Error Modeling and Compensation of CNC Machining Based on Deep Learning and Reinforcement Learning. Int. J. Adv. Manuf. Technol..

[41] Huang X., Zhao F., Tao T., Mei X. (2021). A Newly Developed Corner Smoothing Methodology Based on Clothoid Splines for High Speed Machine Tools. Robot. Comput. Integr. Manuf..

[42] Liu M., Huang Y., Yin L., Guo J., Shao X., Zhang G. (2014). Development and Implementation of a NURBS Interpolator with Smooth Feedrate Scheduling for CNC Machine Tools. Int. J. Mach. Tools Manuf..

[43] Dong J., Ferreira P.M., Stori J.A. (2007). Feed-Rate Optimization with Jerk Constraints for Generating Minimum-Time Trajectories. Int. J. Mach. Tools Manuf..

[44] Liu C., Pei S.Y., Li B.T., Liu H.L., Hong J. (2022). NURBS-Based IGA of Viscous Fluid Movement with Special-Shaped Small Gaps in Hybrid Bearing. Appl. Math. Model..

[45] Wu J., Zhou H., Tang X., Chen J. (2015). Implementation of CL Points Preprocessing Methodology with NURBS Curve Fitting Technique for High-Speed Machining. Comput. Ind. Eng..

[46] Garba U.H., Wang T., Dong J., Tian Y., Kang J., Tian C. (2025). Enhancing Propeller Design with Freeform Contours through NURBS Interpolation for 2D Fabrication, CAD/CAM for 3D Production, Optimized with Taguchi Method and Artificial Neural Network. Results Eng..

[47] (2021). Geometrical Product Specifications (GPS) Surface Texture: Profile Part 2: Terms, Definitions and Surface Texture Parameters.

