# Supplementary material for: High-Speed Precision Machining and Surface Roughness Determination of Freeform Curves Using Galerkin-NURBS Interpolation and Jerk-Limited Trajectory Planning
Source: Sensors (Basel). 2026 Jul 13;26(14):4441. doi: 10.3390/s26144441 (PMC13417550; doi:10.3390/s26144441)
Supplement: Supplementary file 1 [file sensors-26-04441-s001.zip › sensors-4388769-Supplementary S1.pdf]

```
N110	(Butterfly-contour)
N112	G00	G54	G90	X54.4930	Y52.1390
N114	G43	H1	Z136.
N116	Z41.
N118	Z18.
N120	G01
N122	X54.6642	Y52.1249
N124	X54.7446	Y52.1076
N126	X54.8218	Y52.0837
N128	X54.8958	Y52.0535
N130	X54.9667	Y52.0172
N132	X55.0346	Y51.9749
N134	X55.0996	Y51.9270
N136	X55.1617	Y51.8736
N138	X55.2212	Y51.8149
N140	X55.2780	Y51.7511
N142	X55.3323	Y51.6825
N144	X55.3842	Y51.6094
N146	X55.4337	Y51.5318
N148	X55.4810	Y51.4502
N150	X55.5260	Y51.3646
N152	X55.5691	Y51.2753
N154	X55.6101	Y51.1826
N156	X55.6492	Y51.0867
N158	X55.6866	Y50.9878
N160	X55.7222	Y50.8862
N162	X55.7562	Y50.7822
N164	X55.7887	Y50.6758
N166	X55.8197	Y50.5675
```

N168	X55.8494	Y50.4575
N170	X55.8779	Y50.3460
N172	X55.9051	Y50.2333
N174	X55.9313	Y50.1195
N176	X55.9566	Y50.0051
N178	X55.9809	Y49.8902
N180	X56.0044	Y49.7750
N182	X56.0272	Y49.6599
N184	X56.0494	Y49.5451
N186	X56.0710	Y49.4309
N188	X56.0922	Y49.3174
N190	X56.1130	Y49.2049
N192	X56.1335	Y49.0935
N194	X56.1537	Y48.9833
N196	X56.1738	Y48.8745
N198	X56.1938	Y48.7670
N200	X56.2138	Y48.6612
N202	X56.2338	Y48.5569
N204	X56.2539	Y48.4545
N206	X56.2742	Y48.3538
N208	X56.2948	Y48.2551
N210	X56.3158	Y48.1584
N212	X56.3371	Y48.0638
N214	X56.3589	Y47.9713
N216	X56.3812	Y47.8811
N218	X56.4042	Y47.7932
N220	X56.4278	Y47.7076
N222	X56.4522	Y47.6244
N224	X56.4774	Y47.5437

N226	X56.5035	Y47.4655
N228	X56.5306	Y47.3899
N230	X56.5587	Y47.3169
N232	X56.5879	Y47.2465
N234	X56.6182	Y47.1789
N236	X56.6498	Y47.1140
N238	X56.6827	Y47.0518
N240	X56.7171	Y46.9925
N242	X56.7528	Y46.9361
N244	X56.7901	Y46.8825
N246	X56.8288	Y46.8317
N248	X56.8690	Y46.7837
N250	X56.9107	Y46.7383
N252	X56.9537	Y46.6955
N254	X56.9982	Y46.6553
N256	X57.0440	Y46.6177
N258	X57.0912	Y46.5824
N260	X57.1397	Y46.5496
N262	X57.1895	Y46.5191
N264	X57.2406	Y46.4909
N266	X57.2930	Y46.4649
N268	X57.3467	Y46.4412
N270	X57.4016	Y46.4197
N272	X57.4577	Y46.4002
N274	X57.5151	Y46.3829
N276	X57.5736	Y46.3676
N278	X57.6334	Y46.3543
N280	X57.6942	Y46.3429
N282	X57.7563	Y46.3335

N284	X57.8195	Y46.3260
N286	X57.8838	Y46.3203
N288	X57.9492	Y46.3165
N290	X58.0157	Y46.3145
N292	X58.0833	Y46.3142
N294	X58.1519	Y46.3156
N296	X58.2216	Y46.3188
N298	X58.2924	Y46.3236
N300	X58.3641	Y46.3300
N302	X58.4369	Y46.3381
N304	X58.5107	Y46.3477
N306	X58.5855	Y46.3589
N308	X58.6612	Y46.3716
N310	X58.7379	Y46.3857
N312	X58.8155	Y46.4014
N314	X58.8941	Y46.4185
N316	X58.9735	Y46.4370
N318	X59.0539	Y46.4569
N320	X59.1352	Y46.4781
N322	X59.2173	Y46.5007
N324	X59.3003	Y46.5246
N326	X59.3841	Y46.5498
N328	X59.4688	Y46.5762
N330	X59.5543	Y46.6039
N332	X59.6406	Y46.6328
N334	X59.7276	Y46.6628
N336	X59.8154	Y46.6941
N338	X59.9040	Y46.7264
N340	X59.9933	Y46.7599

N342	X60.0833	Y46.7945
N344	X60.1741	Y46.8301
N346	X60.2655	Y46.8667
N348	X60.3575	Y46.9044
N350	X60.4502	Y46.9431
N352	X60.5436	Y46.9827
N354	X60.6375	Y47.0232
N356	X60.7321	Y47.0647
N358	X60.8272	Y47.1071
N360	X60.9228	Y47.1503
N362	X61.0190	Y47.1943
N364	X61.1157	Y47.2392
N366	X61.2128	Y47.2848
N368	X61.3105	Y47.3312
N370	X61.4085	Y47.3784
N372	X61.5070	Y47.4262
N374	X61.6059	Y47.4747
N376	X61.7051	Y47.5239
N378	X61.8047	Y47.5737
N380	X61.9046	Y47.6241
N382	X62.0048	Y47.6750
N384	X62.1052	Y47.7265
N386	X62.2059	Y47.7785
N388	X62.3068	Y47.8309
N390	X62.4079	Y47.8839
N392	X62.5091	Y47.9372
N394	X62.6104	Y47.9909
N396	X62.7118	Y48.0450
N398	X62.8133	Y48.0994

N400	X62.9148	Y48.1540
N402	X63.0163	Y48.2090
N404	X63.1178	Y48.2642
N406	X63.2192	Y48.3195
N408	X63.3205	Y48.3751
N410	X63.4216	Y48.4307
N412	X63.5226	Y48.4864
N414	X63.6233	Y48.5422
N416	X63.7238	Y48.5981
N418	X63.8240	Y48.6539
N420	X63.9240	Y48.7097
N422	X64.0236	Y48.7655
N424	X64.1230	Y48.8213
N426	X64.2221	Y48.8771
N428	X64.3210	Y48.9329
N430	X64.4195	Y48.9887
N432	X64.5178	Y49.0445
N434	X64.6158	Y49.1003
N436	X64.7135	Y49.1561
N438	X64.8109	Y49.2119
N440	X64.9081	Y49.2676
N442	X65.0049	Y49.3234
N444	X65.1015	Y49.3791
N446	X65.1978	Y49.4349
N448	X65.2938	Y49.4906
N450	X65.3895	Y49.5463
N452	X65.4849	Y49.6019
N454	X65.5800	Y49.6576
N456	X65.6749	Y49.7132

N458	X65.7694	Y49.7688
N460	X65.8636	Y49.8244
N462	X65.9576	Y49.8800
N464	X66.0513	Y49.9356
N466	X66.1446	Y49.9911
N468	X66.2377	Y50.0466
N470	X66.3305	Y50.1021
N472	X66.4229	Y50.1576
N474	X66.5151	Y50.2130
N476	X66.6070	Y50.2684
N478	X66.6986	Y50.3238
N480	X66.7899	Y50.3791
N482	X66.8809	Y50.4344
N484	X66.9716	Y50.4897
N486	X67.0620	Y50.5449
N488	X67.1521	Y50.6002
N490	X67.2419	Y50.6553
N492	X67.3315	Y50.7105
N494	X67.4207	Y50.7656
N496	X67.5096	Y50.8207
N498	X67.5982	Y50.8757
N500	X67.6866	Y50.9307
N502	X67.7746	
N504	X67.8623	Y51.0406
N506	X67.9498	Y51.0955
N508	X68.0369	Y51.1504
N510	X68.1238	Y51.2052
N512	X68.2104	Y51.2599
N514	X68.2966	Y51.3147

N516	X68.3826	Y51.3693
N518	X68.4683	Y51.4240
N520	X68.5537	Y51.4786
N522	X68.6388	Y51.5331
N524	X68.7236	Y51.5876
N526	X68.8081	Y51.6421
N528	X68.8924	Y51.6965
N530	X68.9763	Y51.7509
N532	X69.0600	Y51.8052
N534	X69.1434	Y51.8595
N536	X69.2264	Y51.9137
N538	X69.3092	Y51.9679
N540	X69.3918	Y52.0220
N542	X69.4740	Y52.0761
N544	X69.5560	Y52.1301
N546	X69.6377	Y52.1841
N548	X69.7191	Y52.2381
N550	X69.8002	Y52.2919
N552	X69.8810	Y52.3458
N554	X69.9616	Y52.3995
N556	X70.0419	Y52.4533
N558	X70.1219	Y52.5070
N560	X70.2017	Y52.5606
N562	X70.2812	Y52.6142
N564	X70.3604	Y52.6677
N566	X70.4393	Y52.7212
N568	X70.5180	Y52.7746
N570	X70.5965	Y52.8279
N572	X70.6746	Y52.8812

N574	X70.7525	Y52.9345
N576	X70.8302	Y52.9877
N578	X70.9075	Y53.0408
N580	X70.9847	Y53.0939
N582	X71.0616	Y53.1470
N584	X71.1382	Y53.2000
N586	X71.2146	Y53.2529
N588	X71.2907	Y53.3058
N590	X71.3666	Y53.3586
N592	X71.4422	Y53.4114
N594	X71.5176	Y53.4641
N596	X71.5928	Y53.5168
N598	X71.6677	Y53.5694
N600	X71.7424	Y53.6219
N602	X71.8168	Y53.6744
N604	X71.8910	Y53.7269
N606	X71.9650	Y53.7793
N608	X72.0388	Y53.8316
N610	X72.1123	Y53.8839
N612	X72.1856	Y53.9361
N614	X72.2587	Y53.9883
N616	X72.3316	Y54.0404
N618	X72.4043	Y54.0925
N620	X72.4767	Y54.1445
N622	X72.5489	Y54.1965
N624	X72.6210	Y54.2484
N626	X72.6928	Y54.3003
N628	X72.7644	Y54.3521
N630	X72.8358	Y54.4038

N632	X72.9070	Y54.4555
N634	X72.9781	Y54.5072
N636	X73.0489	Y54.5588
N638	X73.1195	Y54.6104
N640	X73.1900	Y54.6619
N642	X73.2602	Y54.7133
N644	X73.3303	Y54.7647
N646	X73.4002	Y54.8161
N648	X73.4700	Y54.8674
N650	X73.5395	Y54.9187
N652	X73.6089	Y54.9699
N654	X73.6781	Y55.0210
N656	X73.7471	Y55.0722
N658	X73.8160	Y55.1232
N660	X73.8848	Y55.1743
N662	X73.9533	Y55.2252
N664	X74.0217	Y55.2762
N666	X74.0900	Y55.3271
N668	X74.1581	Y55.3779
N670	X74.2261	Y55.4287
N672	X74.2939	Y55.4795
N674	X74.3616	Y55.5302
N676	X74.4292	Y55.5809
N678	X74.4966	Y55.6315
N680	X74.5639	Y55.6821
N682	X74.6310	Y55.7327
N684	X74.6981	Y55.7832
N686	X74.7650	Y55.8337
N688	X74.8318	Y55.8841

N690	X74.8985	Y55.9346
N692	X74.9651	Y55.9849
N694	X75.0316	Y56.0353
N696	X75.0980	Y56.0856
N698	X75.1642	Y56.1359
N700	X75.2304	Y56.1861
N702	X75.2965	Y56.2363
N704	X75.3625	Y56.2865
N706	X75.4284	Y56.3366
N708	X75.4943	Y56.3867
N710	X75.5600	Y56.4368
N712	X75.6257	Y56.4869
N714	X75.6913	Y56.5369
N716	X75.7568	Y56.5869
N718	X75.8223	Y56.6369
N720	X75.8877	Y56.6869
N722	X75.9531	Y56.7368
N724	X76.0184	Y56.7867
N726	X76.0836	Y56.8366
N728	X76.1488	Y56.8865
N730	X76.2140	Y56.9364
N732	X76.2791	Y56.9862
N734	X76.3442	Y57.0360
N736	X76.4093	Y57.0858
N738	X76.4743	Y57.1356
N740	X76.5393	Y57.1854
N742	X76.6043	Y57.2351
N744	X76.6693	Y57.2849
N746	X76.7342	Y57.3346

N748	X76.7992	Y57.3844
N750	X76.8641	Y57.4341
N752	X76.9290	Y57.4838
N754	X76.9940	Y57.5335
N756	X77.0589	Y57.5832
N758	X77.1239	Y57.6329
N760	X77.1889	Y57.6826
N762	X77.2539	Y57.7323
N764	X77.3189	Y57.7820
N766	X77.3839	Y57.8317
N768	X77.4490	Y57.8814
N770	X77.5141	Y57.9311
N772	X77.5793	Y57.9808
N774	X77.6445	Y58.0305
N776	X77.7097	Y58.0802
N778	X77.7750	Y58.1299
N780	X77.8403	Y58.1797
N782	X77.9057	Y58.2294
N784	X77.9712	Y58.2792
N786	X78.0368	Y58.3290
N788	X78.1024	Y58.3787
N790	X78.1681	Y58.4286
N792	X78.2338	Y58.4784
N794	X78.2997	Y58.5282
N796	X78.3656	Y58.5781
N798	X78.4316	Y58.6280
N800	X78.4978	Y58.6779
N802	X78.5640	Y58.7278
N804	X78.6303	Y58.7778

N806	X78.6968	Y58.8278
N808	X78.7634	Y58.8778
N810	X78.8300	Y58.9279
N812	X78.8968	Y58.9780
N814	X78.9638	Y59.0281
N816	X79.0308	Y59.0782
N818	X79.0980	Y59.1284
N820	X79.1654	Y59.1787
N822	X79.2329	Y59.2289
N824	X79.3005	Y59.2792
N826	X79.3683	Y59.3296
N828	X79.4362	Y59.3800
N830	X79.5043	Y59.4304
N832	X79.5726	Y59.4809
N834	X79.6409	Y59.5313
N836	X79.7095	Y59.5818
N838	X79.7782	Y59.6323
N840	X79.8470	Y59.6828
N842	X79.9160	Y59.7334
N844	X79.9851	Y59.7839
N846	X80.0544	Y59.8344
N848	X80.1238	Y59.8850
N850	X80.1934	Y59.9355
N852	X80.2632	Y59.9861
N854	X80.3331	Y60.0366
N856	X80.4031	Y60.0871
N858	X80.4733	Y60.1376
N860	X80.5436	Y60.1881
N862	X80.6141	Y60.2385

N864	X80.6848	Y60.2889
N866	X80.7556	Y60.3393
N868	X80.8266	Y60.3897
N870	X80.8977	Y60.4400
N872	X80.9689	Y60.4903
N874	X81.0403	Y60.5405
N876	X81.1119	Y60.5907
N878	X81.1836	Y60.6408
N880	X81.2555	Y60.6909
N882	X81.3275	Y60.7410
N884	X81.3997	Y60.7909
N886	X81.4720	Y60.8408
N888	X81.5445	Y60.8907
N890	X81.6171	Y60.9404
N892	X81.6899	Y60.9901
N894	X81.7629	Y61.0397
N896	X81.8360	Y61.0893
N898	X81.9093	Y61.1387
N900	X81.9827	Y61.1881
N902	X82.0562	Y61.2374
N904	X82.1300	Y61.2865
N906	X82.2038	Y61.3356
N908	X82.2779	Y61.3846
N910	X82.3521	Y61.4335
N912	X82.4264	Y61.4822
N914	X82.5009	Y61.5309
N916	X82.5756	Y61.5794
N918	X82.6504	Y61.6278
N920	X82.7253	Y61.6761

N922	X82.8005	Y61.7243
N924	X82.8757	Y61.7724
N926	X82.9512	Y61.8203
N928	X83.0268	Y61.8681
N930	X83.1025	Y61.9157
N932	X83.1784	Y61.9632
N934	X83.2545	Y62.0106
N936	X83.3307	Y62.0578
N938	X83.4071	Y62.1048
N940	X83.4837	Y62.1517
N942	X83.5604	Y62.1985
N944	X83.6372	Y62.2451
N946	X83.7142	Y62.2915
N948	X83.7914	Y62.3377
N950	X83.8687	Y62.3838
N952	X83.9462	Y62.4297
N954	X84.0239	Y62.4754
N956	X84.1017	Y62.5209
N958	X84.1797	Y62.5663
N960	X84.2578	Y62.6115
N962	X84.3361	Y62.6564
N964	X84.4145	Y62.7012
N966	X84.4931	Y62.7458
N968	X84.5719	Y62.7901
N970	X84.6508	Y62.8343
N972	X84.7299	Y62.8783
N974	X84.8092	Y62.9220
N976	X84.8886	Y62.9655
N978	X84.9681	Y63.0089

N980	X85.0479	Y63.0519
N982	X85.1278	Y63.0948
N984	X85.2078	Y63.1374
N986	X85.2880	Y63.1798
N988	X85.3684	Y63.2220
N990	X85.4490	Y63.2639
N992	X85.5297	Y63.3056
N994	X85.6105	Y63.3470
N996	X85.6915	Y63.3882
N998	X85.7727	Y63.4291
N1000	X85.8541	Y63.4698
N1002	X85.9356	Y63.5102
N1004	X86.0173	Y63.5504
N1006	X86.0991	Y63.5903
N1008	X86.1811	Y63.6299
N1010	X86.2633	Y63.6692
N1012	X86.3456	Y63.7083
N1014	X86.4281	Y63.7471
N1016	X86.5107	Y63.7856
N1018	X86.5936	Y63.8238
N1020	X86.6765	Y63.8617
N1022	X86.7597	Y63.8993
N1024	X86.8430	Y63.9366
N1026	X86.9265	Y63.9737
N1028	X87.0101	Y64.0104
N1030	X87.0939	Y64.0468
N1032	X87.1779	Y64.0829
N1034	X87.2620	Y64.1187
N1036	X87.3463	Y64.1542

N1038	X87.4308	Y64.1894
N1040	X87.5154	Y64.2242
N1042	X87.6002	Y64.2587
N1044	X87.6852	Y64.2929
N1046	X87.7703	Y64.3267
N1048	X87.8556	Y64.3602
N1050	X87.9411	Y64.3934
N1052	X88.0267	Y64.4262
N1054	X88.1125	Y64.4586
N1056	X88.1985	Y64.4908
N1058	X88.2846	Y64.5225
N1060	X88.3709	Y64.5539
N1062	X88.4573	Y64.5850
N1064	X88.5440	Y64.6156
N1066	X88.6308	Y64.6460
N1068	X88.7177	Y64.6759
N1070	X88.8049	Y64.7055
N1072	X88.8922	Y64.7346
N1074	X88.9796	Y64.7634
N1076	X89.0673	Y64.7919
N1078	X89.1551	Y64.8199
N1080	X89.2431	Y64.8475
N1082	X89.3312	Y64.8748
N1084	X89.4195	Y64.9016
N1086	X89.5080	Y64.9281
N1088	X89.5967	Y64.9541
N1090	X89.6855	Y64.9797
N1092	X89.7745	Y65.0049
N1094	X89.8636	Y65.0298

N1096	X89.9530	Y65.0541
N1098	X90.0425	Y65.0781
N1100	X90.1321	Y65.1016
N1102	X90.2220	Y65.1247
N1104	X90.3120	Y65.1474
N1106	X90.4022	Y65.1696
N1108	X90.4925	Y65.1914
N1110	X90.5831	Y65.2128
N1112	X90.6737	Y65.2337
N1114	X90.7646	Y65.2542
N1116	X90.8557	Y65.2742
N1118	X90.9469	Y65.2937
N1120	X91.0382	Y65.3128
N1122	X91.1298	Y65.3315
N1124	X91.2215	Y65.3496
N1126	X91.3134	Y65.3673
N1128	X91.4055	Y65.3845
N1130	X91.4977	Y65.4013
N1132	X91.5902	Y65.4176
N1134	X91.6827	Y65.4333
N1136	X91.7755	Y65.4486
N1138	X91.8684	Y65.4634
N1140	X91.9615	Y65.4778
N1142	X92.0548	Y65.4916
N1144	X92.1483	Y65.5049
N1146	X92.2419	Y65.5177
N1148	X92.3357	Y65.5300
N1150	X92.4297	Y65.5418
N1152	X92.5238	Y65.5531

N1154	X92.6182	Y65.5639
N1156	X92.7127	Y65.5741
N1158	X92.8073	Y65.5838
N1160	X92.9022	Y65.5930
N1162	X92.9972	Y65.6017
N1164	X93.0924	Y65.6098
N1166	X93.1878	Y65.6174
N1168	X93.2833	Y65.6245
N1170	X93.3791	Y65.6310
N1172	X93.4750	Y65.6370
N1174	X93.5710	Y65.6424
N1176	X93.6673	Y65.6472
N1178	X93.7637	Y65.6515
N1180	X93.8603	Y65.6553
N1182	X93.9571	Y65.6585
N1184	X94.0541	Y65.6611
N1186	X94.1512	Y65.6631
N1188	X94.2484	Y65.6646
N1190	X94.3458	Y65.6655
N1192	X94.4433	Y65.6658
N1194	X94.5410	Y65.6655
N1196	X94.6387	Y65.6647
N1198	X94.7364	Y65.6633
N1200	X94.8343	Y65.6612
N1202	X94.9321	Y65.6587
N1204	X95.0300	Y65.6555
N1206	X95.1280	Y65.6517
N1208	X95.2259	Y65.6473
N1210	X95.3238	Y65.6423

N1212	X95.4217	Y65.6368
N1214	X95.5195	Y65.6306
N1216	X95.6173	Y65.6238
N1218	X95.7150	Y65.6165
N1220	X95.8126	Y65.6085
N1222	X95.9101	Y65.5999
N1224	X96.0075	Y65.5907
N1226	X96.1048	Y65.5809
N1228	X96.2019	Y65.5704
N1230	X96.2989	Y65.5594
N1232	X96.3957	Y65.5477
N1234	X96.4923	Y65.5354
N1236	X96.5887	Y65.5225
N1238	X96.6849	Y65.5089
N1240	X96.7809	Y65.4948
N1242	X96.8766	Y65.4799
N1244	X96.9720	Y65.4645
N1246	X97.0672	Y65.4484
N1248	X97.1621	Y65.4317
N1250	X97.2567	Y65.4143
N1252	X97.3509	Y65.3963
N1254	X97.4448	Y65.3777
N1256	X97.5384	Y65.3584
N1258	X97.6316	Y65.3384
N1260	X97.7245	Y65.3178
N1262	X97.8169	Y65.2966
N1264	X97.9090	Y65.2747
N1266	X98.0006	Y65.2521
N1268	X98.0918	Y65.2289

N1270	X98.1825	Y65.2050
N1272	X98.2728	Y65.1804
N1274	X98.3626	Y65.1552
N1276	X98.4519	Y65.1293
N1278	X98.5407	Y65.1027
N1280	X98.6289	Y65.0755
N1282	X98.7167	Y65.0476
N1284	X98.8039	Y65.0190
N1286	X98.8905	Y64.9897
N1288	X98.9765	Y64.9597
N1290	X99.0620	Y64.9291
N1292	X99.1468	Y64.8978
N1294	X99.2310	Y64.8657
N1296	X99.3146	Y64.8330
N1298	X99.3975	Y64.7996
N1300	X99.4797	Y64.7655
N1302	X99.5613	Y64.7307
N1304	X99.6422	Y64.6952
N1306	X99.7223	Y64.6590
N1308	X99.8018	Y64.6221
N1310	X99.8804	Y64.5845
N1312	X99.9584	Y64.5461
N1314	X100.0356	Y64.5071
N1316	X100.1119	Y64.4673
N1318	X100.1875	Y64.4268
N1320	X100.2623	Y64.3856
N1322	X100.3362	Y64.3437
N1324	X100.4094	Y64.3011
N1326	X100.4816	Y64.2577

N1328	X100.5530	Y64.2136
N1330	X100.6235	Y64.1688
N1332	X100.6931	Y64.1233
N1334	X100.7617	Y64.0770
N1336	X100.8295	Y64.0300
N1338	X100.8963	Y63.9822
N1340	X100.9621	Y63.9337
N1342	X101.0270	Y63.8844
N1344	X101.0909	Y63.8345
N1346	X101.1538	Y63.7837
N1348	X101.2157	Y63.7322
N1350	X101.2765	Y63.6800
N1352	X101.3363	Y63.6270
N1354	X101.3950	Y63.5732
N1356	X101.4527	Y63.5187
N1358	X101.5093	Y63.4635
N1360	X101.5647	Y63.4074
N1362	X101.6191	Y63.3506
N1364	X101.6723	Y63.2931
N1366	X101.7245	Y63.2348
N1368	X101.7755	Y63.1757
N1370	X101.8255	Y63.1159
N1372	X101.8744	Y63.0554
N1374	X101.9222	Y62.9942
N1376	X101.9690	Y62.9322
N1378	X102.0147	Y62.8696
N1380	X102.0593	Y62.8062
N1382	X102.1029	Y62.7421
N1384	X102.1454	Y62.6774

N1386	X102.1869	Y62.6120
N1388	X102.2274	Y62.5459
N1390	X102.2668	Y62.4791
N1392	X102.3052	Y62.4117
N1394	X102.3426	Y62.3436
N1396	X102.3790	Y62.2748
N1398	X102.4145	Y62.2055
N1400	X102.4489	Y62.1354
N1402	X102.4823	Y62.0648
N1404	X102.5147	Y61.9935
N1406	X102.5462	Y61.9217
N1408	X102.5767	Y61.8492
N1410	X102.6062	Y61.7761
N1412	X102.6348	Y61.7024
N1414	X102.6625	Y61.6282
N1416	X102.6892	Y61.5533
N1418	X102.7149	Y61.4779
N1420	X102.7397	Y61.4019
N1422	X102.7636	Y61.3254
N1424	X102.7866	Y61.2483
N1426	X102.8087	Y61.1706
N1428	X102.8298	Y61.0924
N1430	X102.8501	Y61.0137
N1432	X102.8695	Y60.9344
N1434	X102.8879	Y60.8547
N1436	X102.9055	Y60.7744
N1438	X102.9223	Y60.6936
N1440	X102.9381	Y60.6123
N1442	X102.9531	Y60.5305

N1444	X102.9673	Y60.4483
N1446	X102.9805	Y60.3655
N1448	X102.9930	Y60.2823
N1450	X103.0046	Y60.1986
N1452	X103.0154	Y60.1145
N1454	X103.0253	Y60.0299
N1456	X103.0345	Y59.9449
N1458	X103.0428	Y59.8594
N1460	X103.0503	Y59.7735
N1462	X103.0570	Y59.6872
N1464	X103.0630	Y59.6004
N1466	X103.0681	Y59.5132
N1468	X103.0724	Y59.4257
N1470	X103.0760	Y59.3377
N1472	X103.0788	Y59.2493
N1474	X103.0809	Y59.1606
N1476	X103.0822	Y59.0715
N1478	X103.0827	Y58.9820
N1480	X103.0825	Y58.8921
N1482	X103.0816	Y58.8019
N1484	X103.0799	Y58.7114
N1486	X103.0775	Y58.6205
N1488	X103.0744	Y58.5292
N1490	X103.0706	Y58.4376
N1492	X103.0661	Y58.3457
N1494	X103.0609	Y58.2535
N1496	X103.0549	Y58.1610
N1498	X103.0483	Y58.0682
N1500	X103.0411	Y57.9750

N1502	X103.0331	Y57.8816
N1504	X103.0245	Y57.7879
N1506	X103.0152	Y57.6940
N1508	X103.0052	Y57.5997
N1510	X102.9946	Y57.5052
N1512	X102.9834	Y57.4104
N1514	X102.9715	Y57.3154
N1516	X102.9590	Y57.2202
N1518	X102.9459	Y57.1247
N1520	X102.9321	Y57.0290
N1522	X102.9178	Y56.9330
N1524	X102.9028	Y56.8369
N1526	X102.8872	Y56.7405
N1528	X102.8711	Y56.6439
N1530	X102.8543	Y56.5472
N1532	X102.8370	Y56.4502
N1534	X102.8191	Y56.3531
N1536	X102.8006	Y56.2558
N1538	X102.7816	Y56.1583
N1540	X102.7620	Y56.0607
N1542	X102.7419	Y55.9629
N1544	X102.7212	Y55.8649
N1546	X102.7000	Y55.7669
N1548	X102.6782	Y55.6686
N1550	X102.6559	Y55.5703
N1552	X102.6331	Y55.4718
N1554	X102.6098	Y55.3732
N1556	X102.5860	Y55.2746
N1558	X102.5617	Y55.1758

N1560	X102.5369	Y55.0769
N1562	X102.5116	Y54.9779
N1564	X102.4859	Y54.8789
N1566	X102.4596	Y54.7798
N1568	X102.4329	Y54.6806
N1570	X102.4057	Y54.5813
N1572	X102.3781	Y54.4820
N1574	X102.3500	Y54.3827
N1576	X102.3215	Y54.2833
N1578	X102.2925	Y54.1839
N1580	X102.2631	Y54.0844
N1582	X102.2333	Y53.9850
N1584	X102.2031	Y53.8855
N1586	X102.1725	Y53.7860
N1588	X102.1414	Y53.6865
N1590	X102.1100	Y53.5870
N1592	X102.0781	Y53.4876
N1594	X102.0459	Y53.3881
N1596	X102.0133	Y53.2887
N1598	X101.9803	Y53.1894
N1600	X101.9469	Y53.0900
N1602	X101.9132	Y52.9907
N1604	X101.8791	Y52.8915
N1606	X101.8447	Y52.7923
N1608	X101.8100	Y52.6932
N1610	X101.7749	Y52.5942
N1612	X101.7394	Y52.4953
N1614	X101.7037	Y52.3964
N1616	X101.6676	Y52.2976

N1618	X101.6312	Y52.1990
N1620	X101.5945	Y52.1004
N1622	X101.5575	Y52.0020
N1624	X101.5203	Y51.9037
N1626	X101.4827	Y51.8055
N1628	X101.4448	Y51.7075
N1630	X101.4067	Y51.6096
N1632	X101.3683	Y51.5118
N1634	X101.3297	Y51.4142
N1636	X101.2908	Y51.3168
N1638	X101.2516	Y51.2195
N1640	X101.2122	Y51.1224
N1642	X101.1726	Y51.0255
N1644	X101.1327	Y50.9288
N1646	X101.0926	Y50.8323
N1648	X101.0523	Y50.7360
N1650	X101.0118	Y50.6399
N1652	X100.9710	Y50.5440
N1654	X100.9301	Y50.4483
N1656	X100.8890	Y50.3529
N1658	X100.8477	Y50.2577
N1660	X100.8062	Y50.1627
N1662	X100.7645	Y50.0681
N1664	X100.7227	Y49.9736
N1666	X100.6807	Y49.8794
N1668	X100.6386	Y49.7855
N1670	X100.5963	Y49.6919
N1672	X100.5538	Y49.5986
N1674	X100.5112	Y49.5055

N1676	X100.4685	Y49.4128
N1678	X100.4257	Y49.3203
N1680	X100.3828	Y49.2282
N1682	X100.3397	Y49.1364
N1684	X100.2965	Y49.0449
N1686	X100.2533	Y48.9537
N1688	X100.2099	Y48.8629
N1690	X100.1665	Y48.7724
N1692	X100.1229	Y48.6823
N1694	X100.0793	Y48.5925
N1696	X100.0357	Y48.5031
N1698	X99.9919	Y48.4141
N1700	X99.9481	Y48.3255
N1702	X99.9043	Y48.2372
N1704	X99.8604	Y48.1493
N1706	X99.8165	Y48.0619
N1708	X99.7726	Y47.9748
N1710	X99.7286	Y47.8882
N1712	X99.6846	Y47.8019
N1714	X99.6406	Y47.7160
N1716	X99.5965	Y47.6306
N1718	X99.5525	Y47.5455
N1720	X99.5084	Y47.4607
N1722	X99.4643	Y47.3764
N1724	X99.4202	Y47.2924
N1726	X99.3760	Y47.2087
N1728	X99.3319	Y47.1254
N1730	X99.2877	Y47.0425
N1732	X99.2436	Y46.9598

N1734	X99.1994	Y46.8775
N1736	X99.1552	Y46.7955
N1738	X99.1110	Y46.7139
N1740	X99.0668	Y46.6325
N1742	X99.0226	Y46.5515
N1744	X98.9784	Y46.4707
N1746	X98.9343	Y46.3902
N1748	X98.8901	Y46.3101
N1750	X98.8459	Y46.2301
N1752	X98.8017	Y46.1505
N1754	X98.7575	Y46.0711
N1756	X98.7134	Y45.9920
N1758	X98.6692	Y45.9132
N1760	X98.6251	Y45.8345
N1762	X98.5810	Y45.7561
N1764	X98.5369	Y45.6780
N1766	X98.4928	Y45.6001
N1768	X98.4487	Y45.5224
N1770	X98.4047	Y45.4449
N1772	X98.3607	Y45.3676
N1774	X98.3167	Y45.2905
N1776	X98.2727	Y45.2136
N1778	X98.2287	Y45.1369
N1780	X98.1848	Y45.0604
N1782	X98.1409	Y44.9840
N1784	X98.0971	Y44.9078
N1786	X98.0533	Y44.8318
N1788	X98.0095	Y44.7560
N1790	X97.9657	Y44.6802

N1792	X97.9220	Y44.6047
N1794	X97.8784	Y44.5292
N1796	X97.8347	Y44.4539
N1798	X97.7911	Y44.3787
N1800	X97.7476	Y44.3037
N1802	X97.7041	Y44.2287
N1804	X97.6607	Y44.1539
N1806	X97.6173	Y44.0791
N1808	X97.5739	Y44.0044
N1810	X97.5306	Y43.9299
N1812	X97.4874	Y43.8554
N1814	X97.4442	Y43.7809
N1816	X97.4011	Y43.7066
N1818	X97.3580	Y43.6323
N1820	X97.3150	Y43.5580
N1822	X97.2721	Y43.4838
N1824	X97.2292	Y43.4097
N1826	X97.1864	Y43.3355
N1828	X97.1437	Y43.2614
N1830	X97.1010	Y43.1874
N1832	X97.0584	Y43.1133
N1834	X97.0159	Y43.0392
N1836	X96.9734	Y42.9652
N1838	X96.9311	Y42.8911
N1840	X96.8888	Y42.8171
N1842	X96.8465	Y42.7430
N1844	X96.8044	Y42.6689
N1846	X96.7623	Y42.5947
N1848	X96.7204	Y42.5205

N1850	X96.6785	Y42.4463
N1852	X96.6367	Y42.3721
N1854	X96.5949	Y42.2977
N1856	X96.5533	Y42.2234
N1858	X96.5118	Y42.1489
N1860	X96.4703	Y42.0744
N1862	X96.4290	Y41.9998
N1864	X96.3877	Y41.9251
N1866	X96.3466	Y41.8503
N1868	X96.3055	Y41.7754
N1870	X96.2646	Y41.7004
N1872	X96.2237	Y41.6253
N1874	X96.1830	Y41.5501
N1876	X96.1424	Y41.4747
N1878	X96.1018	Y41.3992
N1880	X96.0614	Y41.3236
N1882	X96.0211	Y41.2478
N1884	X95.9809	Y41.1719
N1886	X95.9408	Y41.0959
N1888	X95.9008	Y41.0196
N1890	X95.8610	Y40.9432
N1892	X95.8213	Y40.8666
N1894	X95.7816	Y40.7898
N1896	X95.7422	Y40.7129
N1898	X95.7028	Y40.6357
N1900	X95.6636	Y40.5584
N1902	X95.6244	Y40.4808
N1904	X95.5855	Y40.4030
N1906	X95.5466	Y40.3250

N1908	X95.5079	Y40.2468
N1910	X95.4693	Y40.1683
N1912	X95.4308	Y40.0896
N1914	X95.3925	Y40.0107
N1916	X95.3543	Y39.9315
N1918	X95.3163	Y39.8520
N1920	X95.2784	Y39.7723
N1922	X95.2407	Y39.6923
N1924	X95.2030	Y39.6120
N1926	X95.1656	Y39.5314
N1928	X95.1283	Y39.4505
N1930	X95.0911	Y39.3694
N1932	X95.0541	Y39.2879
N1934	X95.0172	Y39.2062
N1936	X94.9805	Y39.1241
N1938	X94.9440	Y39.0416
N1940	X94.9076	Y38.9589
N1942	X94.8713	Y38.8758
N1944	X94.8353	Y38.7924
N1946	X94.7993	Y38.7086
N1948	X94.7636	Y38.6245
N1950	X94.7280	Y38.5400
N1952	X94.6926	Y38.4552
N1954	X94.6574	Y38.3699
N1956	X94.6223	Y38.2843
N1958	X94.5874	Y38.1983
N1960	X94.5526	Y38.1119
N1962	X94.5181	Y38.0251
N1964	X94.4837	Y37.9379

N1966	X94.4495	Y37.8503
N1968	X94.4155	Y37.7623
N1970	X94.3816	Y37.6738
N1972	X94.3480	Y37.5849
N1974	X94.3145	Y37.4956
N1976	X94.2812	Y37.4058
N1978	X94.2481	Y37.3156
N1980	X94.2152	Y37.2249
N1982	X94.1825	Y37.1338
N1984	X94.1500	Y37.0421
N1986	X94.1177	Y36.9500
N1988	X94.0855	Y36.8575
N1990	X94.0536	Y36.7644
N1992	X94.0218	Y36.6709
N1994	X93.9900	Y36.5770
N1996	X93.9581	Y36.4826
N1998	X93.9261	Y36.3880
N2000	X93.8939	Y36.2931
N2002	X93.8615	Y36.1979
N2004	X93.8286	Y36.1025
N2006	X93.7953	Y36.0070
N2008	X93.7615	Y35.9114
N2010	X93.7271	Y35.8157
N2012	X93.6920	Y35.7201
N2014	X93.6562	Y35.6245
N2016	X93.6195	Y35.5290
N2018	X93.5820	Y35.4336
N2020	X93.5435	Y35.3386
N2022	X93.5039	Y35.2438

N2024	X93.4632	Y35.1494
N2026	X93.4213	Y35.0554
N2028	X93.3782	Y34.9618
N2030	X93.3338	Y34.8689
N2032	X93.2880	Y34.7765
N2034	X93.2408	Y34.6849
N2036	X93.1920	Y34.5940
N2038	X93.1418	Y34.5039
N2040	X93.0899	Y34.4148
N2042	X93.0363	Y34.3266
N2044	X92.9810	Y34.2394
N2046	X92.9240	Y34.1534
N2048	X92.8652	Y34.0685
N2050	X92.8045	Y33.9849
N2052	X92.7419	Y33.9027
N2054	X92.6774	Y33.8219
N2056	X92.6109	Y33.7425
N2058	X92.5425	Y33.6647
N2060	X92.4720	Y33.5885
N2062	X92.3994	Y33.5139
N2064	X92.3248	Y33.4412
N2066	X92.2481	Y33.3703
N2068	X92.1693	Y33.3012
N2070	X92.0884	Y33.2341
N2072	X92.0053	Y33.1691
N2074	X91.9202	Y33.1061
N2076	X91.8329	Y33.0452
N2078	X91.7435	Y32.9866
N2080	X91.6519	Y32.9302

N2082	X91.5583	Y32.8761
N2084	X91.4626	Y32.8243
N2086	X91.3648	Y32.7750
N2088	X91.2649	Y32.7281
N2090	X91.1631	Y32.6836
N2092	X91.0592	Y32.6417
N2094	X90.9534	Y32.6024
N2096	X90.8456	Y32.5656
N2098	X90.7360	Y32.5315
N2100	X90.6245	Y32.5000
N2102	X90.5112	Y32.4711
N2104	X90.3962	Y32.4449
N2106	X90.2795	Y32.4215
N2108	X90.1611	Y32.4007
N2110	X90.0411	Y32.3826
N2112	X89.9196	Y32.3673
N2114	X89.7966	Y32.3547
N2116	X89.6723	Y32.3448
N2118	X89.5465	Y32.3376
N2120	X89.4196	Y32.3330
N2122	X89.2914	Y32.3312
N2124	X89.1621	Y32.3320
N2126	X89.0322	Y32.3352
N2128	X88.9018	Y32.3407
N2130	X88.7712	Y32.3483
N2132	X88.6408	Y32.3578
N2134	X88.5107	Y32.3691
N2136	X88.3813	Y32.3821
N2138	X88.2526	Y32.3966

N2140	X88.1250	Y32.4124
N2142	X87.9986	Y32.4294
N2144	X87.8735	Y32.4475
N2146	X87.7500	Y32.4666
N2148	X87.6282	Y32.4865
N2150	X87.5082	Y32.5071
N2152	X87.3901	Y32.5283
N2154	X87.2741	Y32.5500
N2156	X87.1603	Y32.5721
N2158	X87.0487	Y32.5944
N2160	X86.9394	Y32.6170
N2162	X86.8326	Y32.6397
N2164	X86.7283	Y32.6623
N2166	X86.6265	Y32.6850
N2168	X86.5273	Y32.7074
N2170	X86.4308	Y32.7297
N2172	X86.3370	Y32.7517
N2174	X86.2460	Y32.7733
N2176	X86.1577	Y32.7945
N2178	X86.0723	Y32.8152
N2180	X85.9898	Y32.8354
N2182	X85.9101	Y32.8550
N2184	X85.8333	Y32.8739
N2186	X85.7595	Y32.8921
N2188	X85.6887	Y32.9095
N2190	X85.6208	Y32.9262
N2192	X85.5560	Y32.9419
N2194	X85.4942	Y32.9568
N2196	X85.4354	Y32.9706

N2198	X85.3798	Y32.9835
N2200	X85.3273	Y32.9953
N2202	X85.2780	Y33.0059
N2204	X85.2318	Y33.0155
N2206	Y33.0238	
N2208	X85.1491	Y33.0308
N2210	X85.1127	Y33.0365
N2212	X85.0796	Y33.0409
N2214	X85.0499	Y33.0438
N2216	X85.0236	Y33.0453
N2218	X85.0008	Y33.0452
N2220	X84.9815	Y33.0436
N2222	X84.9658	Y33.0402
N2224	X84.9539	Y33.0352
N2226	X84.9456	Y33.0284
N2228	X84.9412	Y33.0197
N2230	X84.9403	Y33.0093
N2232	X84.9430	Y32.9971
N2234	X84.9492	Y32.9831
N2236	X84.9586	Y32.9674
N2238	X84.9714	Y32.9500
N2240	X84.9874	Y32.9309
N2242	X85.0065	Y32.9101
N2244	X85.0286	Y32.8876
N2246	X85.0538	Y32.8634
N2248	X85.0819	Y32.8375
N2250	X85.1129	Y32.8100
N2252	X85.1467	Y32.7807
N2254	X85.1834	Y32.7498

N2256	X85.2227	Y32.7171
N2258	X85.2648	Y32.6828
N2260	X85.3095	Y32.6467
N2262	X85.3568	Y32.6089
N2264	X85.4067	Y32.5694
N2266	X85.4591	Y32.5282
N2268	X85.5140	Y32.4852
N2270	X85.5714	Y32.4404
N2272	X85.6311	Y32.3939
N2274	X85.6932	Y32.3456
N2276	X85.7576	Y32.2955
N2278	X85.8242	Y32.2437
N2280	X85.8931	Y32.1900
N2282	X85.9642	Y32.1344
N2284	X86.0373	Y32.0771
N2286	X86.1125	Y32.0178
N2288	X86.1897	Y31.9568
N2290	X86.2688	Y31.8939
N2292	X86.3498	Y31.8291
N2294	X86.4326	Y31.7624
N2296	X86.5170	Y31.6939
N2298	X86.6031	Y31.6235
N2300	X86.6907	Y31.5513
N2302	X86.7798	Y31.4772
N2304	X86.8702	Y31.4013
N2306	X86.9618	Y31.3235
N2308	X87.0545	Y31.2439
N2310	X87.1482	Y31.1625
N2312	X87.2427	Y31.0794

N2314	X87.3379	Y30.9946
N2316	X87.4337	Y30.9080
N2318	X87.5298	Y30.8198
N2320	X87.6261	Y30.7300
N2322	X87.7224	Y30.6387
N2324	X87.8186	Y30.5459
N2326	X87.9143	Y30.4517
N2328	X88.0095	Y30.3562
N2330	X88.1037	Y30.2595
N2332	X88.1969	Y30.1616
N2334	X88.2888	Y30.0627
N2336	X88.3792	Y29.9628
N2338	X88.4680	Y29.8619
N2340	X88.5552	Y29.7599
N2342	X88.6406	Y29.6568
N2344	X88.7242	Y29.5526
N2346	X88.8059	Y29.4472
N2348	X88.8857	Y29.3406
N2350	X88.9634	Y29.2327
N2352	X89.0389	Y29.1236
N2354	X89.1122	Y29.0133
N2356	X89.1831	Y28.9016
N2358	X89.2517	Y28.7886
N2360	X89.3178	Y28.6743
N2362	X89.3812	Y28.5586
N2364	X89.4421	Y28.4416
N2366	X89.5002	Y28.3232
N2368	X89.5555	Y28.2034
N2370	X89.6079	Y28.0823

N2372 X89.6573 Y27.9598

N2374 X89.7037 Y27.8359

N2376 X89.7470 Y27.7107

N2378 X89.7871 Y27.5842

N2380 X89.8240 Y27.4563

N2382 X89.8576 Y27.3271

N2384 X89.8879 Y27.1966

N2386 X89.9147 Y27.0649

N2388 X89.9382 Y26.9319

N2390 X89.9582 Y26.7977

N2392 X89.9748 Y26.6624

N2394 X89.9878 Y26.5260

N2396 X89.9973 Y26.3886

N2398 X90.0032 Y26.2501

N2400 X90.0056 Y26.1107

N2402 X90.0044 Y25.9704

N2404 X89.9997 Y25.8292

N2406 X89.9915 Y25.6872

N2408 X89.9797 Y25.5446

N2410 X89.9645 Y25.4013

N2412 X89.9458 Y25.2574

N2414 X89.9237 Y25.1131

N2416 X89.8982 Y24.9683

N2418 X89.8695 Y24.8231

N2420 X89.8374 Y24.6777

N2422 X89.8022 Y24.5321

N2424 X89.7639 Y24.3864

N2426 X89.7225 Y24.2407

N2428 X89.6782 Y24.0950

N2430	X89.6310	Y23.9494
N2432	X89.5810	Y23.8041
N2434	X89.5284	Y23.6590
N2436	X89.4731	Y23.5144
N2438	X89.4154	Y23.3701
N2440	X89.3553	Y23.2264
N2442	X89.2930	Y23.0834
N2444	X89.2285	Y22.9409
N2446	X89.1619	Y22.7993
N2448	X89.0934	Y22.6585
N2450	X89.0232	Y22.5185
N2452	X88.9512	Y22.3796
N2454	X88.8776	Y22.2416
N2456	X88.8027	Y22.1047
N2458	X88.7263	Y21.9690
N2460	X88.6488	Y21.8344
N2462	X88.5702	Y21.7011
N2464	X88.4905	Y21.5691
N2466	X88.4100	Y21.4384
N2468	X88.3288	Y21.3091
N2470	X88.2469	Y21.1813
N2472	X88.1644	Y21.0548
N2474	X88.0815	Y20.9299
N2476	X87.9983	Y20.8065
N2478	X87.9149	Y20.6846
N2480	X87.8313	Y20.5643
N2482	X87.7476	Y20.4456
N2484	X87.6640	Y20.3285
N2486	X87.5805	Y20.2130

N2488	X87.4973	Y20.0991
N2490	X87.4143	Y19.9868
N2492	X87.3317	Y19.8762
N2494	X87.2495	Y19.7673
N2496	X87.1678	Y19.6600
N2498	X87.0867	Y19.5544
N2500	X87.0063	Y19.4505
N2502	X86.9267	Y19.3483
N2504	X86.8479	Y19.2479
N2506	X86.7701	Y19.1492
N2508	X86.6932	Y19.0524
N2510	X86.6174	Y18.9573
N2512	X86.5426	Y18.8641
N2514	X86.4690	Y18.7726
N2516	X86.3965	Y18.6828
N2518	X86.3253	Y18.5949
N2520	X86.2553	Y18.5086
N2522	X86.1866	Y18.4241
N2524	X86.1191	Y18.3412
N2526	X86.0529	Y18.2601
N2528	X85.9881	Y18.1805
N2530	X85.9245	Y18.1026
N2532	X85.8623	Y18.0262
N2534	X85.8014	Y17.9513
N2536	X85.7418	Y17.8780
N2538	X85.6835	Y17.8061
N2540	X85.6266	Y17.7357
N2542	X85.5709	Y17.6666
N2544	X85.5166	Y17.5989

N2546	X85.4635	Y17.5325
N2548	X85.4117	Y17.4674
N2550	X85.3612	Y17.4035
N2552	X85.3119	Y17.3408
N2554	X85.2639	Y17.2793
N2556	X85.2171	Y17.2189
N2558	X85.1715	Y17.1595
N2560	X85.1271	Y17.1013
N2562	X85.0839	Y17.0440
N2564	X85.0419	Y16.9877
N2566	X85.0009	Y16.9324
N2568	X84.9612	Y16.8779
N2570	X84.9225	Y16.8244
N2572	X84.8849	Y16.7716
N2574	X84.8484	Y16.7197
N2576	X84.8129	Y16.6685
N2578	X84.7785	Y16.6180
N2580	X84.7451	Y16.5683
N2582	X84.7127	Y16.5192
N2584	X84.6813	Y16.4708
N2586	X84.6509	Y16.4229
N2588	X84.6214	Y16.3757
N2590	X84.5929	Y16.3290
N2592	X84.5652	Y16.2828
N2594	X84.5385	Y16.2371
N2596	X84.5127	Y16.1918
N2598	X84.4878	Y16.1470
N2600	X84.4637	Y16.1026
N2602	X84.4405	Y16.0586

N2604 X84.4181 Y16.0149

N2606 X84.3965 Y15.9716

N2608 X84.3758 Y15.9286

N2610 X84.3558 Y15.8858

N2612 X84.3366 Y15.8433

N2614 X84.3182 Y15.8011

N2616 X84.3006 Y15.7590

N2618 X84.2837 Y15.7171

N2620 X84.2675 Y15.6754

N2622 X84.2521 Y15.6338

N2624 X84.2374 Y15.5924

N2626 X84.2234 Y15.5510

N2628 X84.2102 Y15.5097

N2630 X84.1976 Y15.4685

N2632 X84.1857 Y15.4273

N2634 X84.1744 Y15.3860

N2636 X84.1639 Y15.3448

N2638 X84.1540 Y15.3035

N2640 X84.1448 Y15.2622

N2642 X84.1362 Y15.2208

N2644 X84.1283 Y15.1793

N2646 X84.1210 Y15.1377

N2648 X84.1144 Y15.0959

N2650 X84.1083 Y15.0539

N2652 X84.1030 Y15.0118

N2654 X84.0982 Y14.9695

N2656 X84.0941 Y14.9269

N2658 Y14.8841

N2660 X84.0877 Y14.8409

N2662	X84.0854	Y14.7976
N2664	X84.0838	Y14.7538
N2666	X84.0827	Y14.7098
N2668	X84.0823	Y14.6654
N2670	X84.0825	Y14.6205
N2672	X84.0833	Y14.5753
N2674	X84.0848	Y14.5296
N2676	X84.0868	Y14.4835
N2678	X84.0895	Y14.4369
N2680	X84.0928	Y14.3898
N2682	X84.0967	Y14.3421
N2684	X84.1013	Y14.2939
N2686	X84.1065	Y14.2451
N2688	X84.1124	Y14.1956
N2690	X84.1189	Y14.1456
N2692	X84.1260	Y14.0948
N2694	X84.1338	Y14.0433
N2696	X84.1423	Y13.9911
N2698	X84.1514	Y13.9381
N2700	X84.1612	Y13.8843
N2702	X84.1718	Y13.8297
N2704	X84.1830	Y13.7742
N2706	X84.1949	Y13.7178
N2708	X84.2076	Y13.6604
N2710	X84.2210	Y13.6021
N2712	X84.2351	Y13.5427
N2714	X84.2500	Y13.4822
N2716	X84.2657	Y13.4207
N2718	X84.2822	Y13.3580

N2720	X84.2995	Y13.2940
N2722	X84.3176	Y13.2288
N2724	X84.3366	Y13.1624
N2726	X84.3565	Y13.0945
N2728	X84.3772	Y13.0253
N2730	X84.3988	Y12.9546
N2732	X84.4212	Y12.8826
N2734	X84.4444	Y12.8094
N2736	X84.4684	Y12.7348
N2738	X84.4931	Y12.6590
N2740	X84.5186	Y12.5820
N2742	X84.5447	Y12.5038
N2744	X84.5716	Y12.4245
N2746	X84.5990	Y12.3440
N2748	X84.6271	Y12.2625
N2750	X84.6557	
N2752	X84.6849	
N2754	X84.7147	Y12.0116
N2756	X84.7449	Y11.9261
N2758	X84.7757	Y11.8395
N2760	X84.8069	Y11.7521
N2762	X84.8385	Y11.6639
N2764	X84.8706	Y11.5748
N2766	X84.9030	Y11.4849
N2768	X84.9358	Y11.3942
N2770	X84.9689	Y11.3028
N2772	X85.0023	Y11.2107
N2774	X85.0360	Y11.1179
N2776	X85.0699	Y11.0245

N2778	X85.1041	Y10.9305
N2780	X85.1384	Y10.8360
N2782	X85.1729	Y10.7410
N2784	X85.2075	Y10.6455
N2786	X85.2422	Y10.5496
N2788	X85.2769	Y10.4533
N2790	X85.3117	Y10.3566
N2792	X85.3465	Y10.2597
N2794	X85.3812	Y10.1626
N2796	X85.4158	Y10.0652
N2798	X85.4503	Y9.9678
N2800	X85.4846	Y9.8703
N2802	X85.5187	Y9.7728
N2804	X85.5525	Y9.6753
N2806	X85.5861	Y9.5779
N2808	X85.6192	Y9.4808
N2810	X85.6520	Y9.3839
N2812	X85.6844	Y9.2873
N2814	X85.7162	Y9.1911
N2816	X85.7474	Y9.0955
N2818	X85.7781	Y9.0004
N2820	X85.8080	Y8.9060
N2822	X85.8372	Y8.8123
N2824	X85.8656	Y8.7196
N2826	X85.8931	Y8.6278
N2828	X85.9196	Y8.5371
N2830	X85.9451	Y8.4475
N2832	X85.9694	Y8.3593
N2834	X85.9926	Y8.2726

N2836	X86.0144	Y8.1874
N2838	X86.0349	Y8.1040
N2840	X86.0538	Y8.0224
N2842	X86.0712	Y7.9429
N2844	X86.0868	Y7.8656
N2846	X86.1006	Y7.7906
N2848	X86.1125	Y7.7182
N2850	X86.1225	Y7.6484
N2852	X86.1304	Y7.5811
N2854	X86.1364	Y7.5164
N2856	X86.1404	Y7.4543
N2858	X86.1423	Y7.3948
N2860	X86.1423	Y7.3379
N2862	X86.1401	Y7.2836
N2864	X86.1359	Y7.2319
N2866	X86.1295	Y7.1829
N2868	X86.1210	Y7.1366
N2870	X86.1104	Y7.0930
N2872	X86.0975	Y7.0522
N2874	X86.0825	Y7.0140
N2876	X86.0652	Y6.9787
N2878	X86.0456	Y6.9463
N2880	X86.0237	Y6.9166
N2882	X85.9994	Y6.8899
N2884	X85.9728	Y6.8662
N2886	X85.9437	Y6.8455
N2888	X85.9122	Y6.8278
N2890	X85.8781	Y6.8132
N2892	X85.8416	Y6.8018

N2894	X85.8025	Y6.7935
N2896	X85.7607	Y6.7886
N2898	X85.7163	Y6.7870
N2900	X85.6692	Y6.7888
N2902	X85.6194	Y6.7940
N2904	X85.5668	Y6.8028
N2906	X85.5114	Y6.8152
N2908	X85.4532	Y6.8312
N2910	X85.3921	Y6.8510
N2912	X85.3281	Y6.8746
N2914	X85.2612	Y6.9021
N2916	X85.1913	Y6.9335
N2918	X85.1184	Y6.9690
N2920	X85.0425	Y7.0086
N2922	X84.9637	Y7.0523
N2924	X84.8818	Y7.1003
N2926	X84.7968	Y7.1526
N2928	X84.7089	Y7.2093
N2930	X84.6180	Y7.2704
N2932	X84.5241	Y7.3360
N2934	X84.4272	Y7.4061
N2936	X84.3274	Y7.4809
N2938	X84.2248	Y7.5602
N2940	X84.1194	Y7.6441
N2942	X84.0114	Y7.7327
N2944	X83.9007	Y7.8260
N2946	X83.7875	Y7.9238
N2948	X83.6720	Y8.0263
N2950	X83.5542	Y8.1333

N2952	X83.4345	Y8.2448
N2954	X83.3129	Y8.3607
N2956	X83.1896	Y8.4810
N2958	X83.0650	Y8.6053
N2960	X82.9392	Y8.7337
N2962	X82.8126	Y8.8659
N2964	X82.6855	Y9.0018
N2966	X82.5581	Y9.1410
N2968	X82.4309	Y9.2833
N2970	X82.3043	Y9.4284
N2972	X82.1786	Y9.5759
N2974	X82.0543	Y9.7256
N2976	X81.9318	Y9.8769
N2978	X81.8117	Y10.0295
N2980	X81.6943	Y10.1828
N2982	X81.5803	Y10.3365
N2984	X81.4701	Y10.4899
N2986	X81.3639	Y10.6425
N2988	X81.2619	Y10.7940
N2990	X81.1641	Y10.9438
N2992	X81.0704	Y11.0915
N2994	X80.9809	Y11.2368
N2996	X80.8956	Y11.3792
N2998	X80.8143	Y11.5183
N3000	X80.7371	Y11.6538
N3002	X80.6637	Y11.7853
N3004	X80.5941	Y11.9124
N3006	X80.5280	Y12.0350
N3008	X80.4653	Y12.1528

N3010	X80.4059	Y12.2654
N3012	X80.3495	Y12.3727
N3014	X80.2959	Y12.4745
N3016	X80.2448	Y12.5705
N3018	X80.1960	Y12.6607
N3020	X80.1492	Y12.7449
N3022	X80.1042	Y12.8230
N3024	X80.0607	Y12.8949
N3026	X80.0184	Y12.9605
N3028	X79.9770	Y13.0198
N3030	X79.9362	
N3032	X79.8958	Y13.1189
N3034	X79.8554	Y13.1588
N3036	X79.8146	Y13.1921
N3038	X79.7733	Y13.2189
N3040	X79.7311	Y13.2391
N3042	X79.6876	Y13.2526
N3044	X79.6427	Y13.2597
N3046	X79.5965	Y13.2605
N3048	X79.5488	Y13.2553
N3050	X79.4998	Y13.2443
N3052	X79.4495	Y13.2278
N3054	X79.3978	Y13.2060
N3056	X79.3448	Y13.1791
N3058	X79.2905	Y13.1475
N3060	X79.2349	Y13.1112
N3062	X79.1781	Y13.0706
N3064	X79.1199	Y13.0258
N3066	X79.0606	Y12.9771

N3068 X79.0000 Y12.9248

N3070 X78.9382 Y12.8690

N3072 X78.8753 Y12.8099

N3074 X78.8112 Y12.7479

N3076 X78.7459 Y12.6831

N3078 X78.6795 Y12.6158

N3080 X78.6119 Y12.5462

N3082 X78.5433 Y12.4745

N3084 X78.4737 Y12.4009

N3086 X78.4030 Y12.3258

N3088 X78.3312 Y12.2493

N3090 X78.2585 Y12.1716

N3092 X78.1848 Y12.0931

N3094 X78.1101 Y12.0140

N3096 X78.0346 Y11.9345

N3098 X77.9581 Y11.8548

N3100 X77.8808 Y11.7752

N3102 X77.8027 Y11.6960

N3104 X77.7238 Y11.6175

N3106 X77.6441 Y11.5398

N3108 X77.5638 Y11.4633

N3110 X77.4827 Y11.3882

N3112 X77.4010 Y11.3147

N3114 X77.3187 Y11.2433

N3116 X77.2358 Y11.1740

N3118 X77.1525 Y11.1073

N3120 X77.0686 Y11.0434

N3122 X76.9843 Y10.9824

N3124 X76.8996 Y10.9246

N3126	X76.8146	Y10.8697
N3128	X76.7291	Y10.8179
N3130	X76.6433	Y10.7690
N3132	X76.5571	Y10.7230
N3134	X76.4706	Y10.6799
N3136	X76.3837	Y10.6397
N3138	X76.2966	Y10.6024
N3140	X76.2091	Y10.5679
N3142	X76.1214	Y10.5361
N3144	X76.0333	Y10.5071
N3146	X75.9451	Y10.4807
N3148	X75.8565	Y10.4571
N3150	X75.7678	Y10.4361
N3152	X75.6788	Y10.4177
N3154	X75.5896	Y10.4018
N3156	X75.5003	Y10.3885
N3158	X75.4107	Y10.3777
N3160	X75.3210	Y10.3693
N3162	X75.2311	Y10.3633
N3164	X75.1411	Y10.3597
N3166	X75.0510	Y10.3584
N3168	X74.9607	Y10.3594
N3170	X74.8703	Y10.3626
N3172	X74.7799	Y10.3680
N3174	X74.6893	Y10.3756
N3176	X74.5987	Y10.3854
N3178	X74.5080	Y10.3971
N3180	X74.4173	Y10.4110
N3182	X74.3266	Y10.4268

N3184	X74.2358	Y10.4446
N3186	X74.1450	Y10.4642
N3188	X74.0542	Y10.4857
N3190	X73.9635	Y10.5091
N3192	X73.8727	Y10.5342
N3194	X73.7820	Y10.5610
N3196	X73.6913	Y10.5895
N3198	X73.6007	Y10.6196
N3200	X73.5102	Y10.6513
N3202	X73.4197	Y10.6846
N3204	X73.3293	Y10.7193
N3206	X73.2390	Y10.7555
N3208	X73.1488	Y10.7931
N3210	X73.0587	Y10.8321
N3212	X72.9688	Y10.8723
N3214	X72.8789	Y10.9139
N3216	X72.7893	Y10.9566
N3218	X72.6997	Y11.0005
N3220	X72.6103	Y11.0455
N3222	X72.5211	Y11.0916
N3224	X72.4321	Y11.1387
N3226	X72.3432	Y11.1868
N3228	X72.2545	Y11.2359
N3230	X72.1661	Y11.2858
N3232	X72.0778	Y11.3365
N3234	X71.9898	Y11.3881
N3236	X71.9019	Y11.4404
N3238	X71.8143	Y11.4934
N3240	X71.7269	

N3242 X71.6398 Y11.6013

N3244 X71.5529 Y11.6561

N3246 X71.4663 Y11.7114

N3248 X71.3799 Y11.7672

N3250 X71.2938 Y11.8234

N3252 X71.2080 Y11.8799

N3254 X71.1225 Y11.9368

N3256 X71.0372 Y11.9940

N3258 X70.9522 Y12.0514

N3260 X70.8675 Y12.1090

N3262 X70.7832 Y12.1668

N3264 X70.6991 Y12.2246

N3266 X70.6153 Y12.2825

N3268 X70.5319 Y12.3404

N3270 X70.4487 Y12.3982

N3272 X70.3659 Y12.4560

N3274 X70.2834 Y12.5136

N3276 X70.2013 Y12.5710

N3278 X70.1194 Y12.6283

N3280 X70.0380 Y12.6854

N3282 X69.9568 Y12.7422

N3284 X69.8760 Y12.7989

N3286 X69.7955 Y12.8555

N3288 X69.7153 Y12.9118

N3290 X69.6354 Y12.9680

N3292 X69.5559 Y13.0240

N3294 X69.4767 Y13.0799

N3296 X69.3979 Y13.1356

N3298 X69.3193 Y13.1911

N3300	X69.2411	Y13.2465
N3302	X69.1632	Y13.3018
N3304	X69.0856	Y13.3569
N3306	X69.0084	Y13.4119
N3308	X68.9315	Y13.4667
N3310	X68.8549	Y13.5214
N3312	X68.7786	Y13.5760
N3314	X68.7026	Y13.6304
N3316	X68.6270	Y13.6848
N3318	X68.5516	Y13.7390
N3320	X68.4766	Y13.7931
N3322	X68.4019	Y13.8471
N3324	X68.3276	Y13.9010
N3326	X68.2535	Y13.9548
N3328	X68.1798	Y14.0085
N3330	X68.1063	Y14.0621
N3332	X68.0332	Y14.1156
N3334	X67.9604	Y14.1690
N3336	X67.8879	Y14.2224
N3338	X67.8158	Y14.2757
N3340	X67.7439	Y14.3289
N3342	X67.6723	Y14.3820
N3344	X67.6011	Y14.4351
N3346	X67.5302	Y14.4881
N3348	X67.4595	Y14.5410
N3350	X67.3892	Y14.5939
N3352	X67.3192	Y14.6468
N3354	X67.2495	Y14.6996
N3356	X67.1801	Y14.7523

N3358	X67.1110	Y14.8050
N3360	X67.0422	Y14.8577
N3362	X66.9737	Y14.9103
N3364	X66.9056	Y14.9630
N3366	X66.8377	Y15.0156
N3368	X66.7701	Y15.0681
N3370	X66.7028	Y15.1207
N3372	X66.6358	Y15.1732
N3374	X66.5692	Y15.2258
N3376	X66.5028	Y15.2783
N3378	X66.4367	Y15.3308
N3380	X66.3709	Y15.3834
N3382	X66.3055	Y15.4359
N3384	X66.2403	Y15.4885
N3386	X66.1754	Y15.5411
N3388	X66.1108	Y15.5936
N3390	X66.0465	Y15.6463
N3392	X65.9825	Y15.6989
N3394	X65.9188	Y15.7516
N3396	X65.8554	Y15.8043
N3398	X65.7922	Y15.8570
N3400	X65.7294	Y15.9098
N3402	X65.6668	Y15.9626
N3404	X65.6046	Y16.0155
N3406	X65.5426	Y16.0684
N3408	X65.4809	Y16.1214
N3410	X65.4196	Y16.1744
N3412	X65.3585	Y16.2275
N3414	X65.2976	Y16.2807

N3416	X65.2371	Y16.3339
N3418	X65.1769	Y16.3873
N3420	X65.1169	Y16.4407
N3422	X65.0572	Y16.4942
N3424	X64.9978	Y16.5478
N3426	X64.9386	Y16.6016
N3428	X64.8797	Y16.6555
N3430	X64.8211	Y16.7095
N3432	X64.7628	Y16.7636
N3434	X64.7047	Y16.8179
N3436	X64.6468	Y16.8724
N3438	X64.5892	Y16.9270
N3440	X64.5319	Y16.9818
N3442	X64.4748	Y17.0367
N3444	X64.4180	Y17.0919
N3446	X64.3614	Y17.1473
N3448	X64.3050	Y17.2029
N3450	X64.2489	Y17.2586
N3452	X64.1930	Y17.3147
N3454	X64.1373	Y17.3709
N3456	X64.0819	Y17.4274
N3458	X64.0267	Y17.4841
N3460	X63.9717	Y17.5411
N3462	X63.9169	Y17.5984
N3464	X63.8623	Y17.6560
N3466	X63.8080	Y17.7138
N3468	X63.7538	Y17.7719
N3470	X63.6999	Y17.8303
N3472	X63.6462	Y17.8890

N3474	X63.5927	Y17.9481
N3476	X63.5393	Y18.0074
N3478	X63.4862	Y18.0671
N3480	X63.4333	Y18.1272
N3482	X63.3805	Y18.1876
N3484	X63.3280	Y18.2483
N3486	X63.2756	Y18.3094
N3488	X63.2234	Y18.3709
N3490	X63.1714	Y18.4328
N3492	X63.1195	Y18.4951
N3494	X63.0679	Y18.5577
N3496	X63.0164	Y18.6208
N3498	X62.9650	Y18.6843
N3500	X62.9139	Y18.7482
N3502	X62.8629	Y18.8126
N3504	X62.8120	Y18.8774
N3506	X62.7613	Y18.9426
N3508	X62.7108	Y19.0083
N3510	X62.6604	Y19.0745
N3512	X62.6101	Y19.1411
N3514	X62.5600	Y19.2083
N3516	X62.5101	Y19.2759
N3518	X62.4603	Y19.3440
N3520	X62.4106	Y19.4127
N3522	X62.3610	Y19.4818
N3524	X62.3116	Y19.5515
N3526	X62.2623	Y19.6217
N3528	X62.2131	Y19.6925
N3530	X62.1640	Y19.7638

N3532	X62.1151	Y19.8356
N3534	X62.0663	Y19.9081
N3536	X62.0176	Y19.9811
N3538	X61.9690	Y20.0546
N3540	X61.9205	Y20.1288
N3542	X61.8721	Y20.2036
N3544	X61.8238	Y20.2790
N3546	X61.7756	Y20.3550
N3548	X61.7275	Y20.4317
N3550	X61.6795	Y20.5089
N3552	X61.6316	Y20.5868
N3554	X61.5837	Y20.6654
N3556	X61.5360	Y20.7446
N3558	X61.4883	Y20.8245
N3560	X61.4407	Y20.9051
N3562	X61.3932	Y20.9864
N3564	X61.3457	Y21.0683
N3566	X61.2984	Y21.1510
N3568	X61.2511	Y21.2343
N3570	X61.2038	Y21.3184
N3572	X61.1566	Y21.4032
N3574	X61.1095	Y21.4887
N3576	X61.0624	Y21.5750
N3578	X61.0154	Y21.6620
N3580	X60.9684	Y21.7498
N3582	X60.9215	Y21.8384
N3584	X60.8746	Y21.9277
N3586	X60.8277	Y22.0178
N3588	X60.7809	Y22.1088

N3590	X60.7342	Y22.2005
N3592	X60.6874	Y22.2930
N3594	X60.6407	Y22.3863
N3596	X60.5940	Y22.4805
N3598	X60.5473	Y22.5755
N3600	X60.5007	Y22.6714
N3602	X60.4541	Y22.7681
N3604	X60.4074	Y22.8656
N3606	X60.3608	Y22.9641
N3608	X60.3142	Y23.0634
N3610	X60.2676	Y23.1636
N3612	X60.2210	Y23.2646
N3614	X60.1745	Y23.3666
N3616	X60.1279	Y23.4695
N3618	X60.0813	Y23.5733
N3620	X60.0347	Y23.6781
N3622	X59.9880	Y23.7838
N3624	X59.9414	Y23.8904
N3626	X59.8947	Y23.9980
N3628	X59.8481	Y24.1065
N3630	X59.8014	Y24.2160
N3632	X59.7547	Y24.3265
N3634	X59.7079	Y24.4379
N3636	X59.6612	Y24.5502
N3638	X59.6144	Y24.6633
N3640	X59.5677	Y24.7772
N3642	X59.5209	Y24.8919
N3644	X59.4742	Y25.0073
N3646	X59.4275	Y25.1233

N3648	X59.3809	Y25.2400
N3650	X59.3343	Y25.3572
N3652	X59.2877	Y25.4749
N3654	X59.2413	Y25.5931
N3656	X59.1948	Y25.7117
N3658	X59.1485	Y25.8307
N3660	X59.1023	Y25.9501
N3662	X59.0561	Y26.0697
N3664	X59.0101	Y26.1895
N3666	X58.9641	Y26.3096
N3668	X58.9183	Y26.4297
N3670	X58.8726	Y26.5500
N3672	X58.8271	Y26.6704
N3674	X58.7816	Y26.7907
N3676	X58.7364	Y26.9110
N3678	X58.6913	Y27.0312
N3680	X58.6463	Y27.1513
N3682	X58.6016	Y27.2712
N3684	X58.5570	Y27.3908
N3686	X58.5126	Y27.5102
N3688	X58.4685	Y27.6293
N3690	X58.4245	Y27.7480
N3692	X58.3807	Y27.8663
N3694	X58.3372	Y27.9842
N3696	X58.2939	Y28.1015
N3698	X58.2508	Y28.2183
N3700	X58.2080	Y28.3344
N3702	X58.1655	Y28.4500
N3704	X58.1232	Y28.5648

N3706	X58.0812	Y28.6789
N3708	X58.0395	Y28.7922
N3710	X57.9980	Y28.9047
N3712	X57.9569	Y29.0163
N3714	X57.9160	Y29.1270
N3716	X57.8755	Y29.2367
N3718	X57.8353	Y29.3454
N3720	X57.7955	Y29.4531
N3722	X57.7559	Y29.5596
N3724	X57.7167	Y29.6650
N3726	X57.6779	Y29.7691
N3728	X57.6394	Y29.8721
N3730	X57.6013	Y29.9737
N3732	X57.5636	Y30.0740
N3734	X57.5263	Y30.1729
N3736	X57.4894	Y30.2703
N3738	X57.4528	Y30.3663
N3740	X57.4167	Y30.4607
N3742	X57.3810	Y30.5536
N3744	X57.3457	Y30.6449
N3746	X57.3109	Y30.7344
N3748	X57.2765	Y30.8223
N3750	X57.2426	Y30.9084
N3752	X57.2091	Y30.9927
N3754	X57.1761	Y31.0752
N3756	X57.1435	Y31.1557
N3758	X57.1115	Y31.2343
N3760	X57.0799	Y31.3110
N3762	X57.0489	

N3764	X57.0183	Y31.4580
N3766	X56.9883	Y31.5284
N3768	X56.9588	Y31.5966
N3770	X56.9298	Y31.6625
N3772	X56.9014	Y31.7262
N3774	X56.8735	Y31.7876
N3776	X56.8462	Y31.8466
N3778	X56.8194	Y31.9031
N3780	X56.7933	Y31.9573
N3782	X56.7677	Y32.0089
N3784	X56.7426	Y32.0580
N3786	X56.7182	Y32.1045
N3788	X56.6944	Y32.1483
N3790	X56.6712	Y32.1895
N3792	X56.6487	Y32.2279
N3794	X56.6267	Y32.2635
N3796	X56.6054	Y32.2963
N3798	X56.5848	Y32.3262
N3800	X56.5648	Y32.3532
N3802	X56.5455	Y32.3773
N3804	X56.5268	Y32.3983
N3806	X56.5088	Y32.4163
N3808	X56.4915	Y32.4311
N3810	X56.4749	Y32.4428
N3812	X56.4590	Y32.4513
N3814	X56.4438	Y32.4566
N3816	X56.4294	Y32.4585
N3818	X56.4156	Y32.4572
N3820	X56.4026	Y32.4524

N3822	X56.3904	Y32.4442
N3824	Y32.4325	
N3826	X56.3682	Y32.4173
N3828	X56.3582	Y32.3986
N3830	X56.3490	Y32.3762
N3832	X56.3406	Y32.3502
N3834	X56.3329	Y32.3208
N3836	X56.3260	Y32.2879
N3838	X56.3199	Y32.2516
N3840	X56.3145	Y32.2121
N3842	X56.3098	Y32.1693
N3844	X56.3059	Y32.1234
N3846	X56.3027	Y32.0744
N3848	X56.3002	Y32.0224
N3850	X56.2984	Y31.9675
N3852	X56.2974	Y31.9097
N3854	X56.2970	Y31.8491
N3856	X56.2973	Y31.7858
N3858	X56.2983	Y31.7198
N3860	X56.3000	Y31.6512
N3862	X56.3023	Y31.5802
N3864	X56.3053	Y31.5066
N3866	X56.3090	Y31.4307
N3868	X56.3133	Y31.3525
N3870	X56.3183	Y31.2721
N3872	X56.3239	Y31.1895
N3874	X56.3301	Y31.1048
N3876	X56.3369	Y31.0181
N3878	X56.3444	Y30.9294

N3880 X56.3525 Y30.8389

N3882 X56.3611 Y30.7465

N3884 X56.3704 Y30.6524

N3886 X56.3802 Y30.5567

N3888 X56.3907 Y30.4593

N3890 X56.4017 Y30.3604

N3892 X56.4132 Y30.2600

N3894 X56.4253 Y30.1583

N3896 X56.4380 Y30.0553

N3898 X56.4513 Y29.9509

N3900 X56.4650 Y29.8455

N3902 X56.4793 Y29.7389

N3904 X56.4941 Y29.6312

N3906 X56.5095 Y29.5227

N3908 X56.5253 Y29.4132

N3910 X56.5417 Y29.3029

N3912 X56.5585 Y29.1918

N3914 X56.5759 Y29.0801

N3916 X56.5937 Y28.9677

N3918 X56.6120 Y28.8548

N3920 X56.6307 Y28.7414

N3922 X56.6500 Y28.6276

N3924 X56.6697 Y28.5135

N3926 X56.6898 Y28.3992

N3928 X56.7104 Y28.2846

N3930 X56.7314 Y28.1699

N3932 X56.7528 Y28.0552

N3934 X56.7747 Y27.9405

N3936 X56.7970 Y27.8259

N3938	X56.8197	Y27.7114
N3940	X56.8428	Y27.5972
N3942	X56.8662	Y27.4833
N3944	X56.8901	Y27.3698
N3946	X56.9144	Y27.2567
N3948	X56.9390	Y27.1441
N3950	X56.9640	Y27.0321
N3952	X56.9893	Y26.9207
N3954	X57.0150	Y26.8101
N3956	X57.0410	Y26.7002
N3958	X57.0674	Y26.5909
N3960	X57.0940	Y26.4824
N3962	X57.1209	Y26.3746
N3964	X57.1480	Y26.2674
N3966	X57.1753	Y26.1609
N3968	X57.2028	Y26.0551
N3970	X57.2305	Y25.9499
N3972	X57.2583	Y25.8454
N3974	X57.2862	Y25.7415
N3976	X57.3143	Y25.6382
N3978	X57.3424	Y25.5356
N3980	X57.3706	Y25.4335
N3982	X57.3988	Y25.3321
N3984	X57.4270	Y25.2313
N3986	X57.4552	Y25.1311
N3988	X57.4833	Y25.0314
N3990	X57.5114	Y24.9324
N3992	X57.5394	Y24.8339
N3994	X57.5673	Y24.7359

N3996	X57.5951	Y24.6385
N3998	X57.6227	Y24.5417
N4000	X57.6501	Y24.4454
N4002	X57.6773	Y24.3496
N4004	X57.7043	Y24.2543
N4006	X57.7311	Y24.1596
N4008	X57.7576	Y24.0653
N4010	X57.7838	Y23.9716
N4012	X57.8097	Y23.8783
N4014	X57.8352	Y23.7855
N4016	X57.8604	Y23.6932
N4018	X57.8852	Y23.6014
N4020	X57.9096	Y23.5100
N4022	X57.9336	Y23.4190
N4024	X57.9571	Y23.3285
N4026	X57.9801	Y23.2385
N4028	X58.0027	Y23.1488
N4030	X58.0247	Y23.0596
N4032	X58.0462	Y22.9708
N4034	X58.0671	Y22.8823
N4036	X58.0874	Y22.7943
N4038	X58.1071	Y22.7067
N4040	X58.1261	Y22.6194
N4042	X58.1446	Y22.5325
N4044	X58.1623	Y22.4460
N4046	X58.1793	Y22.3598
N4048	X58.1956	Y22.2740
N4050	X58.2111	Y22.1884
N4052	X58.2259	Y22.1033

N4054	X58.2398	Y22.0184
N4056	X58.2530	Y21.9339
N4058	X58.2653	Y21.8497
N4060	X58.2767	Y21.7657
N4062	X58.2873	Y21.6821
N4064	X58.2969	Y21.5987
N4066	X58.3056	Y21.5156
N4068	X58.3134	Y21.4328
N4070	X58.3201	Y21.3502
N4072	X58.3259	Y21.2679
N4074	X58.3306	Y21.1858
N4076	X58.3343	Y21.1040
N4078	X58.3369	Y21.0224
N4080	X58.3384	Y20.9410
N4082	X58.3388	Y20.8598
N4084	X58.3381	Y20.7788
N4086	X58.3362	Y20.6980
N4088	X58.3331	Y20.6174
N4090	X58.3287	Y20.5370
N4092	X58.3232	Y20.4567
N4094	X58.3164	Y20.3766
N4096	X58.3083	Y20.2967
N4098	X58.2989	Y20.2169
N4100	X58.2882	Y20.1372
N4102	X58.2762	Y20.0577
N4104	X58.2627	Y19.9783
N4106	X58.2479	Y19.8990
N4108	X58.2317	Y19.8198
N4110	X58.2140	Y19.7407

N4112	X58.1949	Y19.6617
N4114	X58.1742	Y19.5828
N4116	X58.1521	Y19.5040
N4118	X58.1284	Y19.4252
N4120	X58.1032	Y19.3465
N4122	X58.0764	Y19.2679
N4124	X58.0480	Y19.1893
N4126	X58.0180	Y19.1107
N4128	X57.9863	Y19.0321
N4130	X57.9529	Y18.9536
N4132	X57.9179	Y18.8751
N4134	X57.8812	Y18.7966
N4136	X57.8427	Y18.7180
N4138	X57.8024	Y18.6395
N4140	X57.7604	Y18.5609
N4142	X57.7165	Y18.4824
N4144	X57.6709	Y18.4038
N4146	X57.6234	Y18.3252
N4148	X57.5742	Y18.2468
N4150	X57.5233	Y18.1686
N4152	X57.4708	Y18.0907
N4154	X57.4166	Y18.0131
N4156	X57.3608	Y17.9360
N4158	X57.3035	Y17.8594
N4160	X57.2447	Y17.7835
N4162	X57.1844	Y17.7082
N4164	X57.1228	Y17.6337
N4166	X57.0597	Y17.5600
N4168	X56.9953	Y17.4873

N4170	X56.9296	Y17.4155
N4172	X56.8627	Y17.3449
N4174	X56.7945	Y17.2754
N4176	X56.7252	Y17.2072
N4178	X56.6547	Y17.1403
N4180	X56.5831	Y17.0748
N4182	X56.5105	Y17.0108
N4184	X56.4369	Y16.9483
N4186	X56.3623	Y16.8875
N4188	X56.2868	Y16.8284
N4190	X56.2104	Y16.7712
N4192	X56.1331	Y16.7158
N4194	X56.0551	Y16.6624
N4196	X55.9762	Y16.6110
N4198	X55.8967	Y16.5617
N4200	X55.8165	Y16.5147
N4202	X55.7356	Y16.4699
N4204	X55.6541	Y16.4275
N4206	X55.5720	Y16.3876
N4208	X55.4895	Y16.3501
N4210	X55.4064	Y16.3153
N4212	X55.3230	Y16.2831
N4214	X55.2391	Y16.2538
N4216	X55.1548	Y16.2272
N4218	X55.0703	Y16.2036
N4220	X54.9854	Y16.1830
N4222	X54.9004	Y16.1654
N4224	X54.8151	Y16.1510
N4226	X54.7297	Y16.1399

N4228	X54.6441	Y16.1321
N4230	X54.5585	Y16.1277
N4232	X54.4729	Y16.1267
N4234	X54.3873	Y16.1293
N4236	X54.3017	Y16.1353
N4238	X54.2162	Y16.1447
N4240	X54.1309	Y16.1573
N4242	X54.0457	Y16.1732
N4244	X53.9607	Y16.1922
N4246	X53.8760	Y16.2142
N4248	X53.7916	Y16.2392
N4250	X53.7075	Y16.2671
N4252	X53.6238	Y16.2978
N4254	X53.5405	Y16.3312
N4256	X53.4577	Y16.3673
N4258	X53.3753	Y16.4059
N4260	X53.2935	Y16.4470
N4262	X53.2123	Y16.4905
N4264	X53.1317	Y16.5364
N4266	X53.0518	Y16.5845
N4268	X52.9726	Y16.6347
N4270	X52.8941	Y16.6871
N4272	X52.8164	Y16.7415
N4274	X52.7395	Y16.7977
N4276	X52.6635	Y16.8559
N4278	X52.5884	Y16.9158
N4280	X52.5143	Y16.9774
N4282	X52.4411	
N4284	X52.3690	Y17.1053

N4286	X52.2979	Y17.1715
N4288	X52.2280	Y17.2390
N4290	X52.1592	Y17.3079
N4292	X52.0916	Y17.3779
N4294	X52.0252	Y17.4491
N4296	X51.9601	Y17.5213
N4298	X51.8964	Y17.5945
N4300	X51.8340	Y17.6686
N4302	X51.7729	Y17.7435
N4304	X51.7134	Y17.8192
N4306	X51.6552	Y17.8955
N4308	X51.5987	Y17.9723
N4310	X51.5436	Y18.0497
N4312	X51.4902	Y18.1275
N4314	X51.4384	Y18.2056
N4316	X51.3883	Y18.2839
N4318	X51.3400	Y18.3625
N4320	X51.2934	Y18.4411
N4322	X51.2486	Y18.5197
N4324	X51.2056	Y18.5983
N4326	X51.1644	Y18.6769
N4328	X51.1250	Y18.7555
N4330	X51.0873	Y18.8341
N4332	X51.0514	Y18.9127
N4334	X51.0172	Y18.9912
N4336	X50.9847	Y19.0698
N4338	X50.9538	Y19.1485
N4340	X50.9245	Y19.2271
N4342	X50.8969	Y19.3058

N4344	X50.8709	Y19.3846
N4346	X50.8464	Y19.4634
N4348	X50.8235	Y19.5422
N4350	X50.8021	Y19.6212
N4352	X50.7822	Y19.7002
N4354	X50.7638	Y19.7793
N4356	X50.7468	Y19.8585
N4358	X50.7313	Y19.9378
N4360	X50.7171	Y20.0172
N4362	X50.7044	Y20.0968
N4364	X50.6930	Y20.1764
N4366	X50.6830	Y20.2563
N4368	X50.6742	Y20.3362
N4370	X50.6668	Y20.4163
N4372	X50.6606	Y20.4966
N4374	X50.6557	Y20.5770
N4376	X50.6520	Y20.6576
N4378	X50.6495	Y20.7384
N4380	X50.6482	Y20.8194
N4382	X50.6480	Y20.9005
N4384	X50.6490	Y20.9819
N4386	X50.6510	Y21.0635
N4388	X50.6542	Y21.1454
N4390	X50.6584	Y21.2274
N4392	X50.6637	Y21.3097
N4394	X50.6700	Y21.3922
N4396	X50.6772	Y21.4750
N4398	X50.6855	Y21.5581
N4400	X50.6946	Y21.6414

N4402	X50.7048	Y21.7250
N4404	X50.7158	Y21.8089
N4406	X50.7277	Y21.8931
N4408	X50.7404	Y21.9776
N4410	X50.7540	Y22.0624
N4412	X50.7684	Y22.1475
N4414	X50.7835	Y22.2330
N4416	X50.7995	Y22.3187
N4418	X50.8162	Y22.4049
N4420	X50.8335	Y22.4913
N4422	X50.8516	Y22.5782
N4424	X50.8704	Y22.6654
N4426	X50.8898	Y22.7529
N4428	X50.9098	Y22.8409
N4430	X50.9305	Y22.9292
N4432	X50.9517	Y23.0179
N4434	X50.9735	Y23.1071
N4436	X50.9958	Y23.1966
N4438	X51.0186	Y23.2866
N4440	X51.0419	Y23.3770
N4442	X51.0656	Y23.4679
N4444	X51.0898	Y23.5591
N4446	X51.1145	Y23.6509
N4448	X51.1395	Y23.7431
N4450	X51.1649	Y23.8357
N4452	X51.1906	Y23.9289
N4454	X51.2167	Y24.0225
N4456	X51.2431	Y24.1166
N4458	X51.2697	Y24.2113

N4460	X51.2966	Y24.3064
N4462	X51.3238	Y24.4020
N4464	X51.3511	Y24.4982
N4466	X51.3787	Y24.5949
N4468	X51.4064	Y24.6922
N4470	X51.4342	Y24.7899
N4472	X51.4622	Y24.8883
N4474	X51.4903	Y24.9872
N4476	X51.5184	Y25.0867
N4478	X51.5466	Y25.1867
N4480	X51.5748	Y25.2874
N4482	X51.6030	Y25.3886
N4484	X51.6312	Y25.4905
N4486	X51.6594	Y25.5929
N4488	X51.6875	Y25.6960
N4490	X51.7155	Y25.7997
N4492	X51.7434	Y25.9041
N4494	X51.7712	Y26.0090
N4496	X51.7988	Y26.1147
N4498	X51.8262	Y26.2210
N4500	X51.8534	Y26.3279
N4502	X51.8804	Y26.4356
N4504	X51.9072	Y26.5439
N4506	X51.9337	Y26.6529
N4508	X51.9599	Y26.7626
N4510	X51.9857	Y26.8730
N4512	X52.0112	Y26.9841
N4514	X52.0364	Y27.0959
N4516	X52.0612	Y27.2084

N4518	X52.0857	Y27.3213
N4520	X52.1097	Y27.4348
N4522	X52.1334	Y27.5487
N4524	X52.1566	Y27.6629
N4526	X52.1795	Y27.7774
N4528	X52.2020	Y27.8921
N4530	X52.2241	Y28.0069
N4532	X52.2457	Y28.1217
N4534	X52.2669	Y28.2365
N4536	X52.2877	Y28.3513
N4538	X52.3080	Y28.4659
N4540	X52.3279	Y28.5802
N4542	X52.3473	Y28.6943
N4544	X52.3663	Y28.8080
N4546	X52.3848	Y28.9212
N4548	X52.4028	Y29.0340
N4550	X52.4204	Y29.1461
N4552	X52.4374	Y29.2576
N4554	X52.4540	Y29.3684
N4556	X52.4701	Y29.4783
N4558	X52.4856	Y29.5874
N4560	X52.5006	Y29.6956
N4562	X52.5151	Y29.8027
N4564	X52.5291	Y29.9088
N4566	X52.5426	Y30.0137
N4568	X52.5554	Y30.1174
N4570	X52.5678	Y30.2198
N4572	X52.5796	Y30.3208
N4574	X52.5908	Y30.4204

N4576	X52.6014	Y30.5185
N4578	X52.6115	Y30.6150
N4580	X52.6210	Y30.7098
N4582	X52.6299	Y30.8030
N4584	X52.6381	Y30.8943
N4586	X52.6458	Y30.9838
N4588	X52.6529	Y31.0713
N4590	X52.6593	Y31.1569
N4592	X52.6651	Y31.2403
N4594	X52.6703	Y31.3217
N4596	X52.6749	Y31.4008
N4598	X52.6788	Y31.4776
N4600	X52.6820	Y31.5520
N4602	X52.6846	Y31.6240
N4604	X52.6865	Y31.6936
N4606	X52.6877	Y31.7605
N4608	X52.6882	Y31.8248
N4610	X52.6881	Y31.8864
N4612	X52.6872	Y31.9452
N4614	X52.6857	Y32.0012
N4616	X52.6834	Y32.0542
N4618	X52.6805	Y32.1042
N4620	X52.6768	Y32.1512
N4622	X52.6723	Y32.1950
N4624	X52.6672	Y32.2356
N4626	X52.6612	Y32.2729
N4628	X52.6546	Y32.3069
N4630	X52.6471	Y32.3375
N4632	X52.6390	Y32.3645

N4634	X52.6300	Y32.3880
N4636	X52.6202	Y32.4079
N4638	X52.6097	Y32.4242
N4640	X52.5984	Y32.4369
N4642	X52.5864	Y32.4462
N4644	X52.5736	Y32.4520
N4646	X52.5601	Y32.4544
N4648	X52.5459	Y32.4535
N4650	X52.5309	Y32.4493
N4652	X52.5152	Y32.4418
N4654	X52.4988	Y32.4311
N4656	X52.4817	Y32.4173
N4658	X52.4640	Y32.4003
N4660	X52.4455	Y32.3803
N4662	X52.4264	Y32.3572
N4664	X52.4066	Y32.3312
N4666	X52.3861	Y32.3022
N4668	X52.3650	Y32.2703
N4670	X52.3433	Y32.2356
N4672	X52.3209	Y32.1981
N4674	X52.2979	Y32.1579
N4676	X52.2743	Y32.1149
N4678	X52.2500	Y32.0693
N4680	X52.2252	Y32.0211
N4682	X52.1998	Y31.9704
N4684	X52.1738	Y31.9171
N4686	X52.1472	Y31.8613
N4688	X52.1200	Y31.8031
N4690	X52.0923	Y31.7426

N4692	X52.0641	Y31.6797
N4694	X52.0353	Y31.6145
N4696	X52.0059	Y31.5471
N4698	X51.9761	Y31.4775
N4700	X51.9457	Y31.4058
N4702	X51.9148	Y31.3319
N4704	X51.8834	Y31.2561
N4706	X51.8515	Y31.1782
N4708	X51.8191	Y31.0983
N4710	X51.7862	Y31.0165
N4712	X51.7529	Y30.9329
N4714	X51.7191	Y30.8474
N4716	X51.6848	Y30.7602
N4718	X51.6501	Y30.6712
N4720	X51.6150	Y30.5806
N4722	X51.5794	Y30.4883
N4724	X51.5435	Y30.3945
N4726	X51.5071	Y30.2991
N4728	X51.4702	Y30.2021
N4730	X51.4330	Y30.1038
N4732	X51.3954	Y30.0040
N4734	X51.3575	Y29.9029
N4736	X51.3191	Y29.8005
N4738	X51.2804	Y29.6968
N4740	X51.2413	Y29.5919
N4742	X51.2019	Y29.4858
N4744	X51.1622	Y29.3786
N4746	X51.1221	Y29.2703
N4748	X51.0816	Y29.1610

N4750	X51.0409	Y29.0507
N4752	X50.9999	Y28.9395
N4754	X50.9585	Y28.8273
N4756	X50.9169	Y28.7143
N4758	X50.8749	Y28.6005
N4760	X50.8328	Y28.4860
N4762	X50.7903	Y28.3707
N4764	X50.7476	Y28.2548
N4766	X50.7046	Y28.1383
N4768	X50.6614	Y28.0212
N4770	X50.6179	Y27.9036
N4772	X50.5742	Y27.7855
N4774	X50.5303	Y27.6670
N4776	X50.4862	Y27.5480
N4778	X50.4419	Y27.4288
N4780	X50.3974	Y27.3092
N4782	X50.3527	Y27.1895
N4784	X50.3078	Y27.0695
N4786	X50.2627	Y26.9493
N4788	X50.2175	Y26.8291
N4790	X50.1722	Y26.7088
N4792	X50.1267	Y26.5884
N4794	X50.0810	Y26.4682
N4796	X50.0352	Y26.3480
N4798	X49.9893	Y26.2279
N4800	X49.9433	Y26.1080
N4802	X49.8972	Y25.9883
N4804	X49.8510	Y25.8688
N4806	X49.8047	Y25.7497

N4808	X49.7583	Y25.6309
N4810	X49.7118	Y25.5126
N4812	X49.6653	Y25.3947
N4814	X49.6187	Y25.2773
N4816	X49.5721	Y25.1604
N4818	X49.5254	Y25.0441
N4820	X49.4787	Y24.9285
N4822	X49.4319	Y24.8135
N4824	X49.3852	Y24.6993
N4826	X49.3384	Y24.5858
N4828	X49.2917	Y24.4731
N4830	X49.2449	Y24.3614
N4832	X49.1982	Y24.2505
N4834	X49.1515	Y24.1406
N4836	X49.1048	Y24.0317
N4838	X49.0581	Y23.9237
N4840	X49.0115	Y23.8167
N4842	X48.9649	Y23.7106
N4844	X48.9183	Y23.6055
N4846	X48.8717	Y23.5014
N4848	X48.8251	Y23.3981
N4850	X48.7785	Y23.2958
N4852	X48.7319	Y23.1944
N4854	X48.6853	Y23.0938
N4856	X48.6387	Y22.9942
N4858	X48.5922	Y22.8954
N4860	X48.5456	Y22.7976
N4862	X48.4990	Y22.7006
N4864	X48.4523	Y22.6044

N4866	X48.4057	Y22.5091
N4868	X48.3590	Y22.4147
N4870	X48.3123	Y22.3210
N4872	X48.2656	Y22.2282
N4874	X48.2189	Y22.1363
N4876	X48.1721	Y22.0451
N4878	X48.1253	Y21.9547
N4880	X48.0785	Y21.8651
N4882	X48.0316	Y21.7763
N4884	X47.9847	Y21.6883
N4886	X47.9377	Y21.6010
N4888	X47.8907	Y21.5145
N4890	X47.8436	Y21.4288
N4892	X47.7965	Y21.3438
N4894	X47.7493	Y21.2595
N4896	X47.7020	Y21.1759
N4898	X47.6547	Y21.0931
N4900	X47.6073	Y21.0109
N4902	X47.5599	Y20.9295
N4904	X47.5123	Y20.8487
N4906	X47.4647	Y20.7687
N4908	X47.4171	Y20.6893
N4910	X47.3693	Y20.6106
N4912	X47.3215	Y20.5325
N4914	X47.2735	Y20.4551
N4916	X47.2255	Y20.3783
N4918	X47.1774	Y20.3021
N4920	X47.1292	Y20.2266
N4922	X47.0809	Y20.1517

N4924	X47.0325	Y20.0773
N4926	X46.9839	Y20.0036
N4928	X46.9353	Y19.9305
N4930	X46.8866	Y19.8579
N4932	X46.8377	Y19.7860
N4934	X46.7888	Y19.7145
N4936	X46.7397	Y19.6437
N4938	X46.6905	Y19.5734
N4940	X46.6411	Y19.5036
N4942	X46.5917	Y19.4344
N4944	X46.5421	Y19.3656
N4946	X46.4924	Y19.2974
N4948	X46.4425	Y19.2297
N4950	X46.3925	Y19.1625
N4952	X46.3424	Y19.0958
N4954	X46.2921	Y19.0295
N4956	X46.2416	Y18.9638
N4958	X46.1911	Y18.8985
N4960	X46.1403	Y18.8336
N4962	X46.0894	Y18.7692
N4964	X46.0384	Y18.7052
N4966	X45.9871	Y18.6417
N4968	X45.9357	Y18.5785
N4970	X45.8842	Y18.5158
N4972	X45.8325	Y18.4535
N4974	X45.7806	Y18.3916
N4976	X45.7285	Y18.3301
N4978	X45.6762	Y18.2689
N4980	X45.6238	Y18.2081

N4982	X45.5711	Y18.1477
N4984	X45.5183	Y18.0876
N4986	X45.4653	Y18.0279
N4988	X45.4121	Y17.9685
N4990	X45.3587	Y17.9095
N4992	X45.3051	Y17.8507
N4994	X45.2513	Y17.7923
N4996	X45.1973	Y17.7342
N4998	X45.1430	Y17.6763
N5000	X45.0886	Y17.6188
N5002	X45.0339	Y17.5615
N5004	X44.9791	Y17.5045
N5006	X44.9240	Y17.4478
N5008	X44.8687	Y17.3913
N5010	X44.8131	Y17.3350
N5012	X44.7573	Y17.2790
N5014	X44.7013	Y17.2232
N5016	X44.6451	Y17.1676
N5018	X44.5886	Y17.1123
N5020	X44.5319	Y17.0571
N5022	X44.4749	Y17.0022
N5024	X44.4177	Y16.9474
N5026	X44.3602	Y16.8928
N5028	X44.3025	Y16.8383
N5030	X44.2446	Y16.7840
N5032	X44.1863	Y16.7299
N5034	X44.1278	Y16.6759
N5036	X44.0691	Y16.6221
N5038	X44.0100	Y16.5683

N5040 X43.9507 Y16.5147

N5042 X43.8912 Y16.4612

N5044 X43.8313 Y16.4078

N5046 X43.7712 Y16.3545

N5048 X43.7108 Y16.3013

N5050 X43.6501 Y16.2481

N5052 X43.5891 Y16.1950

N5054 X43.5278 Y16.1420

N5056 X43.4663 Y16.0890

N5058 X43.4044 Y16.0361

N5060 X43.3423 Y15.9832

N5062 X43.2799 Y15.9304

N5064 X43.2171 Y15.8777

N5066 X43.1541 Y15.8250

N5068 X43.0908 Y15.7723

N5070 X43.0272 Y15.7196

N5072 X42.9633 Y15.6670

N5074 X42.8992 Y15.6144

N5076 X42.8347 Y15.5618

N5078 X42.7699 Y15.5093

N5080 X42.7048 Y15.4568

N5082 X42.6395 Y15.4042

N5084 X42.5738 Y15.3517

N5086 X42.5079 Y15.2992

N5088 X42.4416 Y15.2467

N5090 X42.3751 Y15.1942

N5092 X42.3082 Y15.1417

N5094 X42.2410 Y15.0891

N5096 X42.1736 Y15.0366

N5098	X42.1058	Y14.9840
N5100	X42.0378	Y14.9314
N5102	X41.9694	Y14.8788
N5104	X41.9008	Y14.8261
N5106	X41.8318	Y14.7735
N5108	X41.7625	Y14.7207
N5110	X41.6930	Y14.6680
N5112	X41.6231	Y14.6152
N5114	X41.5529	Y14.5623
N5116	X41.4824	Y14.5094
N5118	X41.4116	Y14.4565
N5120	X41.3405	Y14.4034
N5122	X41.2691	Y14.3503
N5124	X41.1974	Y14.2972
N5126	X41.1253	Y14.2440
N5128	X41.0530	Y14.1907
N5130	X40.9803	Y14.1373
N5132	X40.9074	Y14.0838
N5134	X40.8341	Y14.0303
N5136	X40.7605	Y13.9766
N5138	X40.6866	Y13.9229
N5140	X40.6124	Y13.8690
N5142	X40.5379	Y13.8151
N5144	X40.4630	Y13.7611
N5146	X40.3879	Y13.7069
N5148	X40.3124	Y13.6527
N5150	X40.2366	Y13.5983
N5152	X40.1605	Y13.5438
N5154	X40.0841	Y13.4892

N5156	X40.0073	Y13.4344
N5158	X39.9303	Y13.3795
N5160	X39.8529	Y13.3245
N5162	X39.7752	Y13.2694
N5164	X39.6971	Y13.2141
N5166	X39.6188	Y13.1586
N5168	X39.5401	Y13.1030
N5170	X39.4611	Y13.0473
N5172	X39.3818	Y12.9914
N5174	X39.3022	Y12.9353
N5176	X39.2222	Y12.8790
N5178	X39.1420	Y12.8226
N5180	X39.0613	Y12.7661
N5182	X38.9804	Y12.7093
N5184	X38.8991	Y12.6524
N5186	X38.8176	Y12.5953
N5188	X38.7356	Y12.5379
N5190	X38.6534	Y12.4804
N5192	X38.5708	Y12.4228
N5194	X38.4879	Y12.3650
N5196	X38.4047	Y12.3072
N5198	X38.3212	Y12.2494
N5200	X38.2374	Y12.1916
N5202	X38.1532	Y12.1338
N5204	X38.0688	Y12.0762
N5206	X37.9841	Y12.0187
N5208	X37.8991	Y11.9615
N5210	X37.8137	Y11.9045
N5212	X37.7282	Y11.8478

N5214	X37.6423	Y11.7914
N5216	X37.5562	Y11.7355
N5218	X37.4698	Y11.6800
N5220	X37.3832	Y11.6249
N5222	X37.2963	Y11.5705
N5224	X37.2092	Y11.5165
N5226	X37.1218	Y11.4633
N5228	X37.0342	Y11.4107
N5230	X36.9464	Y11.3588
N5232	X36.8584	Y11.3077
N5234	X36.7701	Y11.2574
N5236	X36.6817	Y11.2079
N5238	X36.5931	Y11.1594
N5240	X36.5043	Y11.1118
N5242	X36.4153	Y11.0652
N5244	X36.3261	Y11.0197
N5246	X36.2368	Y10.9753
N5248	X36.1473	Y10.9320
N5250	X36.0577	Y10.8899
N5252	X35.9679	Y10.8490
N5254	X35.8781	Y10.8095
N5256	X35.7881	Y10.7712
N5258	X35.6980	Y10.7343
N5260	X35.6078	Y10.6989
N5262	X35.5175	Y10.6649
N5264	X35.4271	Y10.6324
N5266	X35.3367	Y10.6015
N5268	X35.2462	Y10.5723
N5270	X35.1556	Y10.5446

N5272	X35.0650	Y10.5187
N5274	X34.9744	Y10.4945
N5276	X34.8838	Y10.4720
N5278	X34.7931	Y10.4515
N5280	X34.7025	Y10.4328
N5282	X34.6119	Y10.4160
N5284	X34.5213	Y10.4012
N5286	X34.4308	Y10.3884
N5288	X34.3402	Y10.3776
N5290	X34.2498	Y10.3689
N5292	X34.1594	Y10.3624
N5294	X34.0692	Y10.3581
N5296	X33.9790	Y10.3560
N5298	X33.8889	Y10.3561
N5300	X33.7989	Y10.3586
N5302	X33.7091	Y10.3633
N5304	X33.6195	Y10.3705
N5306	X33.5299	Y10.3801
N5308	X33.4406	Y10.3922
N5310	X33.3514	Y10.4068
N5312	X33.2625	Y10.4239
N5314	X33.1737	Y10.4436
N5316	X33.0852	Y10.4659
N5318	X32.9969	Y10.4908
N5320	X32.9089	Y10.5185
N5322	X32.8211	Y10.5488
N5324	X32.7336	Y10.5820
N5326	X32.6464	Y10.6179
N5328	X32.5595	Y10.6566

N5330	X32.4729	Y10.6982
N5332	X32.3866	Y10.7427
N5334	X32.3007	Y10.7900
N5336	X32.2151	Y10.8404
N5338	X32.1300	Y10.8937
N5340	X32.0451	Y10.9500
N5342	X31.9607	Y11.0094
N5344	X31.8767	Y11.0718
N5346	X31.7932	Y11.1371
N5348	X31.7101	Y11.2051
N5350	X31.6276	Y11.2754
N5352	X31.5456	Y11.3479
N5354	X31.4643	Y11.4221
N5356	X31.3836	Y11.4980
N5358	X31.3036	Y11.5751
N5360	X31.2243	Y11.6533
N5362	X31.1459	Y11.7322
N5364	X31.0682	Y11.8116
N5366	X30.9913	Y11.8913
N5368	X30.9153	Y11.9709
N5370	X30.8402	Y12.0503
N5372	X30.7661	Y12.1292
N5374	X30.6928	Y12.2073
N5376	X30.6206	Y12.2845
N5378	X30.5494	Y12.3604
N5380	X30.4792	Y12.4348
N5382	X30.4100	Y12.5075
N5384	X30.3419	Y12.5782
N5386	X30.2749	Y12.6467

N5388	X30.2091	Y12.7129
N5390	X30.1443	Y12.7764
N5392	X30.0807	Y12.8370
N5394	X30.0183	Y12.8945
N5396	X29.9571	Y12.9486
N5398	X29.8971	Y12.9992
N5400	X29.8383	Y13.0460
N5402	X29.7808	Y13.0888
N5404	X29.7245	Y13.1273
N5406	X29.6695	Y13.1614
N5408	X29.6158	Y13.1907
N5410	X29.5634	Y13.2151
N5412	X29.5123	Y13.2344
N5414	X29.4625	Y13.2482
N5416	X29.4141	Y13.2563
N5418	X29.3671	Y13.2586
N5420	X29.3214	Y13.2548
N5422	X29.2772	Y13.2445
N5424	X29.2343	Y13.2277
N5426	X29.1925	Y13.2042
N5428	X29.1514	Y13.1742
N5430	X29.1108	Y13.1376
N5432	X29.0703	Y13.0945
N5434	X29.0297	Y13.0449
N5436	X28.9886	Y12.9888
N5438	X28.9467	Y12.9263
N5440	X28.9038	Y12.8575
N5442	X28.8595	Y12.7824
N5444	X28.8136	Y12.7011

N5446	X28.7658	Y12.6138
N5448	X28.7158	Y12.5205
N5450	X28.6633	Y12.4214
N5452	X28.6082	Y12.3167
N5454	X28.5502	Y12.2065
N5456	X28.4891	Y12.0911
N5458	X28.4247	Y11.9707
N5460	X28.3567	Y11.8456
N5462	X28.2851	Y11.7160
N5464	X28.2096	Y11.5822
N5466	X28.1302	Y11.4447
N5468	X28.0468	Y11.3036
N5470	X27.9592	Y11.1595
N5472	X27.8675	Y11.0127
N5474	X27.7715	Y10.8635
N5476	X27.6714	Y10.7126
N5478	X27.5671	Y10.5602
N5480	X27.4587	Y10.4069
N5482	X27.3462	Y10.2530
N5484	X27.2303	Y10.0992
N5486	X27.1112	Y9.9459
N5488	X26.9895	Y9.7937
N5490	X26.8658	Y9.6429
N5492	X26.7405	Y9.4940
N5494	X26.6140	Y9.3475
N5496	X26.4867	Y9.2035
N5498	X26.3591	Y9.0626
N5500	X26.2316	Y8.9249
N5502	X26.1044	Y8.7907

N5504	X25.9779	Y8.6603
N5506	X25.8525	Y8.5338
N5508	X25.7283	Y8.4115
N5510	X25.6056	Y8.2933
N5512	X25.4847	Y8.1796
N5514	X25.3658	Y8.0703
N5516	X25.2490	Y7.9656
N5518	X25.1345	Y7.8655
N5520	X25.0224	Y7.7700
N5522	X24.9129	Y7.6792
N5524	X24.8060	Y7.5931
N5526	X24.7020	Y7.5115
N5528	X24.6007	Y7.4347
N5530	X24.5023	Y7.3624
N5532	X24.4069	Y7.2947
N5534	X24.3145	Y7.2316
N5536	X24.2250	Y7.1729
N5538	X24.1386	Y7.1186
N5540	X24.0553	Y7.0687
N5542	X23.9749	Y7.0231
N5544	X23.8976	Y6.9817
N5546	X23.8233	Y6.9445
N5548	X23.7520	Y6.9113
N5550	X23.6837	Y6.8821
N5552	X23.6183	Y6.8569
N5554	X23.5559	Y6.8355
N5556	X23.4963	Y6.8179
N5558	X23.4397	Y6.8040
N5560	X23.3858	Y6.7938

N5562	X23.3348	Y6.7871
N5564	X23.2865	Y6.7839
N5566	X23.2410	Y6.7842
N5568	X23.1981	Y6.7878
N5570	X23.1578	Y6.7948
N5572	X23.1202	Y6.8050
N5574	X23.0851	Y6.8183
N5576	X23.0525	Y6.8349
N5578	X23.0225	Y6.8545
N5580	X22.9948	Y6.8771
N5582	X22.9696	Y6.9027
N5584	X22.9468	Y6.9313
N5586	X22.9263	Y6.9627
N5588	X22.9081	Y6.9970
N5590	X22.8922	Y7.0342
N5592	X22.8785	Y7.0741
N5594	X22.8670	Y7.1168
N5596	X22.8577	Y7.1622
N5598	X22.8506	Y7.2103
N5600	X22.8456	Y7.2611
N5602	X22.8427	Y7.3145
N5604	X22.8419	Y7.3706
N5606	X22.8431	Y7.4293
N5608	X22.8464	Y7.4906
N5610	X22.8517	Y7.5545
N5612	X22.8591	Y7.6210
N5614	X22.8684	Y7.6901
N5616	X22.8796	Y7.7618
N5618	X22.8929	Y7.8360

N5620	X22.9079	Y7.9127
N5622	X22.9247	Y7.9915
N5624	X22.9432	Y8.0725
N5626	X22.9631	Y8.1554
N5628	X22.9845	Y8.2400
N5630	X23.0072	Y8.3262
N5632	X23.0311	Y8.4139
N5634	X23.0562	Y8.5030
N5636	X23.0823	Y8.5932
N5638	X23.1094	Y8.6846
N5640	X23.1374	Y8.7770
N5642	X23.1663	Y8.8703
N5644	X23.1959	Y8.9644
N5646	X23.2263	Y9.0591
N5648	X23.2573	Y9.1545
N5650	X23.2888	Y9.2504
N5652	X23.3209	Y9.3467
N5654	X23.3535	Y9.4433
N5656	X23.3864	Y9.5403
N5658	X23.4198	Y9.6375
N5660	X23.4534	Y9.7348
N5662	X23.4874	Y9.8321
N5664	X23.5215	Y9.9295
N5666	X23.5559	Y10.0269
N5668	X23.5903	Y10.1241
N5670	X23.6249	Y10.2212
N5672	X23.6596	Y10.3180
N5674	X23.6943	Y10.4146
N5676	X23.7289	Y10.5109

N5678	X23.7636	Y10.6068
N5680	X23.7981	Y10.7023
N5682	X23.8326	Y10.7974
N5684	X23.8669	Y10.8920
N5686	X23.9010	Y10.9860
N5688	X23.9349	Y11.0795
N5690	X23.9686	Y11.1724
N5692	X24.0021	Y11.2646
N5694	X24.0352	Y11.3562
N5696	X24.0681	Y11.4470
N5698	X24.1006	Y11.5371
N5700	X24.1327	Y11.6264
N5702	X24.1645	Y11.7149
N5704	X24.1958	Y11.8026
N5706	X24.2266	Y11.8893
N5708	X24.2570	Y11.9752
N5710	X24.2869	Y12.0601
N5712	X24.3163	Y12.1440
N5714	X24.3451	Y12.2269
N5716	X24.3734	Y12.3088
N5718	X24.4011	Y12.3897
N5720	X24.4281	Y12.4694
N5722	X24.4545	Y12.5480
N5724	X24.4802	Y12.6255
N5726	X24.5052	Y12.7017
N5728	X24.5295	Y12.7768
N5730	X24.5530	Y12.8506
N5732	X24.5757	Y12.9231
N5734	X24.5976	Y12.9943

N5736	X24.6187	Y13.0641
N5738	X24.6389	Y13.1325
N5740	X24.6583	Y13.1996
N5742	X24.6767	Y13.2653
N5744	X24.6944	Y13.3298
N5746	X24.7112	Y13.3930
N5748	X24.7272	Y13.4551
N5750	X24.7424	Y13.5160
N5752	X24.7569	Y13.5759
N5754	X24.7706	Y13.6347
N5756	X24.7836	Y13.6924
N5758	X24.7958	Y13.7493
N5760	X24.8073	Y13.8052
N5762	X24.8181	Y13.8602
N5764	X24.8282	Y13.9143
N5766	X24.8377	Y13.9677
N5768	X24.8464	Y14.0202
N5770	X24.8545	Y14.0720
N5772	X24.8619	Y14.1231
N5774	X24.8687	Y14.1735
N5776	X24.8748	Y14.2232
N5778	X24.8802	Y14.2723
N5780	X24.8851	Y14.3208
N5782	X24.8892	Y14.3687
N5784	X24.8928	Y14.4161
N5786	X24.8957	Y14.4629
N5788	X24.8981	Y14.5093
N5790	X24.8997	Y14.5551
N5792	X24.9008	Y14.6006

N5794	X24.9013	Y14.6456
N5796	X24.9011	Y14.6902
N5798	X24.9003	Y14.7344
N5800	X24.8989	Y14.7783
N5802	X24.8969	Y14.8219
N5804	X24.8942	Y14.8651
N5806	X24.8910	Y14.9081
N5808	X24.8871	Y14.9508
N5810	X24.8826	Y14.9933
N5812	X24.8775	Y15.0355
N5814	X24.8717	Y15.0776
N5816	X24.8653	Y15.1194
N5818	X24.8583	Y15.1612
N5820	X24.8506	Y15.2027
N5822	X24.8423	Y15.2442
N5824	X24.8333	Y15.2856
N5826	X24.8237	Y15.3269
N5828	X24.8134	Y15.3682
N5830	X24.8025	Y15.4095
N5832	X24.7908	Y15.4507
N5834	X24.7785	Y15.4920
N5836	X24.7655	Y15.5333
N5838	X24.7518	Y15.5747
N5840	X24.7374	Y15.6161
N5842	X24.7222	Y15.6577
N5844	X24.7064	Y15.6994
N5846	X24.6898	Y15.7412
N5848	X24.6724	Y15.7833
N5850	X24.6543	Y15.8255

N5852 X24.6355 Y15.8679

N5854 X24.6158 Y15.9106

N5856 X24.5953 Y15.9536

N5858 X24.5741 Y15.9968

N5860 X24.5520 Y16.0404

N5862 X24.5291 Y16.0843

N5864 X24.5054 Y16.1286

N5866 X24.4808 Y16.1733

N5868 X24.4553 Y16.2184

N5870 Y16.2639

N5872 X24.4017 Y16.3100

N5874 X24.3735 Y16.3565

N5876 X24.3443 Y16.4036

N5878 X24.3143 Y16.4513

N5880 X24.2832 Y16.4995

N5882 X24.2512 Y16.5484

N5884 X24.2182 Y16.5979

N5886 X24.1842 Y16.6481

N5888 X24.1491 Y16.6991

N5890 X24.1130 Y16.7508

N5892 X24.0758 Y16.8032

N5894 X24.0375 Y16.8565

N5896 X23.9982 Y16.9107

N5898 X23.9577 Y16.9657

N5900 X23.9160 Y17.0217

N5902 X23.8733 Y17.0786

N5904 X23.8293 Y17.1366

N5906 X23.7841 Y17.1955

N5908 X23.7378 Y17.2556

N5910	X23.6902	Y17.3167
N5912	X23.6414	Y17.3790
N5914	X23.5913	Y17.4425
N5916	X23.5400	Y17.5072
N5918	X23.4874	Y17.5731
N5920	X23.4335	Y17.6404
N5922	X23.3783	Y17.7090
N5924	X23.3218	Y17.7790
N5926	X23.2640	Y17.8504
N5928	X23.2049	Y17.9232
N5930	X23.1444	Y17.9976
N5932	X23.0827	Y18.0734
N5934	X23.0195	Y18.1508
N5936	X22.9551	Y18.2299
N5938	X22.8894	Y18.3105
N5940	X22.8224	Y18.3928
N5942	X22.7540	Y18.4767
N5944	X22.6844	Y18.5624
N5946	X22.6136	Y18.6498
N5948	X22.5415	Y18.7390
N5950	X22.4683	Y18.8299
N5952	X22.3938	Y18.9226
N5954	X22.3183	Y19.0171
N5956	X22.2417	Y19.1134
N5958	X22.1641	Y19.2114
N5960	X22.0855	Y19.3113
N5962	X22.0061	Y19.4129
N5964	X21.9259	Y19.5163
N5966	X21.8449	Y19.6214

N5968 X21.7633 Y19.7282

N5970 X21.6811 Y19.8367

N5972 X21.5985 Y19.9468

N5974 X21.5155 Y20.0586

N5976 X21.4322 Y20.1720

N5978 X21.3486 Y20.2870

N5980 X21.2649 Y20.4037

N5982 X21.1811 Y20.5219

N5984 X21.0973 Y20.6418

N5986 X21.0136 Y20.7632

N5988 X20.9301 Y20.8862

N5990 X20.8469 Y21.0106

N5992 X20.7642 Y21.1366

N5994 X20.6819 Y21.2641

N5996 X20.6002 Y21.3929

N5998 X20.5193 Y21.5232

N6000 X20.4392 Y21.6548

N6002 X20.3600 Y21.7877

N6004 X20.2819 Y21.9219

N6006 X20.2050 Y22.0573

N6008 X20.1294 Y22.1938

N6010 X20.0551 Y22.3314

N6012 X19.9825 Y22.4701

N6014 X19.9114 Y22.6097

N6016 X19.8422 Y22.7503

N6018 X19.7748 Y22.8917

N6020 X19.7094 Y23.0338

N6022 X19.6461 Y23.1767

N6024 X19.5851 Y23.3201

N6026 X19.5264 Y23.4642

N6028 Y23.6087

N6030 Y23.7536

N6032 X19.3655 Y23.8988

N6034 X19.3172 Y24.0442

N6036 X19.2718 Y24.1898

N6038 X19.2293 Y24.3355

N6040 X19.1898 Y24.4811

N6042 X19.1534 Y24.6267

N6044 X19.1202 Y24.7721

N6046 X19.0902 Y24.9172

N6048 X19.0635 Y25.0620

N6050 X19.0402 Y25.2065

N6052 X19.0203 Y25.3504

N6054 X19.0038 Y25.4938

N6056 X18.9908 Y25.6365

N6058 X18.9813 Y25.7786

N6060 X18.9754 Y25.9199

N6062 X18.9729 Y26.0604

N6064 X18.9741 Y26.2000

N6066 X18.9788 Y26.3386

N6068 X18.9870 Y26.4763

N6070 X18.9988 Y26.6129

N6072 X19.0141 Y26.7484

N6074 X19.0329 Y26.8828

N6076 X19.0552 Y27.0160

N6078 X19.0810 Y27.1479

N6080 X19.1101 Y27.2787

N6082 X19.1426 Y27.4081

N6084	X19.1784	Y27.5363
N6086	X19.2174	Y27.6632
N6088	X19.2596	Y27.7887
N6090	X19.3050	Y27.9128
N6092	X19.3534	Y28.0356
N6094	X19.4048	Y28.1571
N6096	X19.4591	Y28.2772
N6098	X19.5162	Y28.3959
N6100	X19.5762	Y28.5132
N6102	X19.6388	Y28.6292
N6104	X19.7040	Y28.7438
N6106	X19.7717	Y28.8571
N6108	X19.8419	Y28.9691
N6110	X19.9144	Y29.0798
N6112	X19.9892	Y29.1892
N6114	X20.0661	Y29.2974
N6116	X20.1452	Y29.4043
N6118	X20.2263	Y29.5101
N6120	X20.3092	Y29.6146
N6122	X20.3941	Y29.7181
N6124	X20.4807	Y29.8204
N6126	X20.5689	Y29.9216
N6128	X20.6588	Y30.0219
N6130	X20.7501	Y30.1211
N6132	X20.8429	Y30.2193
N6134	X20.9367	Y30.3163
N6136	X21.0315	Y30.4122
N6138	X21.1269	Y30.5068
N6140	X21.2228	Y30.6000

N6142	X21.3189	Y30.6917
N6144	X21.4151	Y30.7820
N6146	X21.5110	Y30.8706
N6148	X21.6067	Y30.9576
N6150	X21.7019	Y31.0429
N6152	X21.7964	Y31.1265
N6154	X21.8901	Y31.2084
N6156	X21.9829	Y31.2885
N6158	X22.0747	Y31.3667
N6160	X22.1652	Y31.4432
N6162	X22.2545	Y31.5178
N6164	X22.3424	Y31.5906
N6166	X22.4287	Y31.6615
N6168	X22.5135	Y31.7306
N6170	X22.5966	Y31.7978
N6172	X22.6780	Y31.8631
N6174	X22.7575	Y31.9266
N6176	X22.8352	Y31.9882
N6178	X22.9109	Y32.0480
N6180	X22.9845	Y32.1059
N6182	X23.0561	Y32.1620
N6184	X23.1256	Y32.2163
N6186	X23.1929	Y32.2688
N6188	X23.2580	Y32.3195
N6190	X23.3207	Y32.3684
N6192	X23.3812	Y32.4155
N6194	X23.4393	Y32.4609
N6196	X23.4950	Y32.5045
N6198	X23.5482	Y32.5464

N6200	X23.5990	Y32.5865
N6202	X23.6472	Y32.6249
N6204	X23.6929	Y32.6616
N6206	X23.7359	Y32.6967
N6208	X23.7763	Y32.7300
N6210	X23.8140	Y32.7616
N6212	X23.8489	Y32.7916
N6214	X23.8811	Y32.8199
N6216	X23.9104	Y32.8465
N6218	X23.9368	Y32.8714
N6220	X23.9603	Y32.8947
N6222	X23.9807	Y32.9162
N6224	X23.9981	Y32.9361
N6226	X24.0123	Y32.9544
N6228	X24.0233	Y32.9709
N6230	X24.0311	Y32.9857
N6232	X24.0354	Y32.9988
N6234	X24.0363	Y33.0101
N6236	X24.0337	Y33.0197
N6238	X24.0274	Y33.0275
N6240	X24.0173	Y33.0336
N6242	X24.0036	Y33.0378
N6244	X23.9862	Y33.0404
N6246	X23.9653	Y33.0414
N6248	X23.9409	Y33.0408
N6250	X23.9131	Y33.0388
N6252	X23.8819	Y33.0353
N6254	X23.8473	Y33.0304
N6256	X23.8095	Y33.0242

N6258 X23.7684 Y33.0167

N6260 X23.7241 Y33.0080

N6262 X23.6766 Y32.9981

N6264 X23.6260 Y32.9871

N6266 X23.5722 Y32.9750

N6268 X23.5154 Y32.9619

N6270 X23.4555 Y32.9477

N6272 X23.3925 Y32.9327

N6274 X23.3266 Y32.9167

N6276 X23.2576 Y32.8999

N6278 X23.1857 Y32.8824

N6280 X23.1108 Y32.8641

N6282 X23.0330 Y32.8451

N6284 X22.9524 Y32.8254

N6286 X22.8688 Y32.8052

N6288 X22.7824 Y32.7845

N6290 X22.6933 Y32.7633

N6292 X22.6013 Y32.7418

N6294 X22.5066 Y32.7199

N6296 X22.4093 Y32.6977

N6298 X22.3093 Y32.6754

N6300 X22.2067 Y32.6529

N6302 X22.1016 Y32.6304

N6304 X21.9940 Y32.6080

N6306 X21.8841 Y32.5857

N6308 X21.7718 Y32.5636

N6310 X21.6574 Y32.5418

N6312 X21.5408 Y32.5205

N6314 X21.4221 Y32.4997

N6316 X21.3016 Y32.4795

N6318 X21.1793 Y32.4600

N6320 X21.0553 Y32.4414

N6322 X20.9299 Y32.4238

N6324 X20.8031 Y32.4072

N6326 X20.6752 Y32.3920

N6328 X20.5463 Y32.3780

N6330 X20.4166 Y32.3657

N6332 X20.2864 Y32.3549

N6334 X20.1559 Y32.3460

N6336 X20.0254 Y32.3391

N6338 X19.8951 Y32.3343

N6340 X19.7652 Y32.3318

N6342 X19.6362 Y32.3317

N6344 X19.5082 Y32.3343

N6346 X19.3813 Y32.3394

N6348 X19.2557 Y32.3472

N6350 X19.1314 Y32.3577

N6352 X19.0084 Y32.3708

N6354 X18.8868 Y32.3865

N6356 X18.7667 Y32.4049

N6358 X18.6482 Y32.4260

N6360 X18.5312 Y32.4497

N6362 X18.4159 Y32.4760

N6364 X18.3022 Y32.5050

N6366 X18.1903 Y32.5365

N6368 X18.0801 Y32.5707

N6370 X17.9718 Y32.6073

N6372 X17.8654 Y32.6466

N6374	X17.7609	Y32.6883
N6376	X17.6582	Y32.7324
N6378	X17.5576	Y32.7790
N6380	X17.4589	Y32.8280
N6382	X17.3623	Y32.8794
N6384	X17.2677	Y32.9330
N6386	X17.1751	Y32.9889
N6388	X17.0846	Y33.0470
N6390	X16.9962	Y33.1072
N6392	X16.9098	Y33.1696
N6394	X16.8255	Y33.2339
N6396	X16.7433	Y33.3003
N6398	X16.6631	Y33.3685
N6400	X16.5850	Y33.4386
N6402	X16.5090	Y33.5105
N6404	X16.4350	Y33.5842
N6406	X16.3630	Y33.6595
N6408	X16.2929	Y33.7364
N6410	X16.2249	Y33.8148
N6412	X16.1588	Y33.8946
N6414	X16.0945	Y33.9759
N6416	X16.0322	Y34.0585
N6418	X15.9717	Y34.1423
N6420	X15.9129	Y34.2273
N6422	X15.8560	Y34.3134
N6424	X15.8007	Y34.4005
N6426	X15.7471	Y34.4886
N6428	X15.6950	Y34.5776
N6430	X15.6446	Y34.6675

N6432	X15.5956	Y34.7580
N6434	X15.5480	Y34.8493
N6436	X15.5019	Y34.9413
N6438	X15.4571	Y35.0337
N6440	X15.4135	Y35.1267
N6442	X15.3711	Y35.2201
N6444	X15.3299	Y35.3139
N6446	X15.2897	Y35.4080
N6448	X15.2505	Y35.5024
N6450	X15.2123	Y35.5970
N6452	X15.1749	Y35.6917
N6454	X15.1383	Y35.7864
N6456	X15.1024	Y35.8813
N6458	X15.0672	Y35.9761
N6460	X15.0325	Y36.0708
N6462	X14.9984	Y36.1654
N6464	X14.9646	Y36.2598
N6466	X14.9312	Y36.3541
N6468	X14.8981	Y36.4480
N6470	X14.8651	Y36.5417
N6472	X14.8323	Y36.6351
N6474	X14.7995	Y36.7280
N6476	X14.7666	Y36.8206
N6478	X14.7337	Y36.9127
N6480	X14.7006	Y37.0043
N6482	X14.6673	Y37.0955
N6484	X14.6339	Y37.1863
N6486	X14.6003	Y37.2766
N6488	X14.5666	Y37.3664

N6490	X14.5328	Y37.4559
N6492	X14.4988	Y37.5449
N6494	X14.4646	Y37.6335
N6496	X14.4303	Y37.7217
N6498	X14.3959	Y37.8095
N6500	X14.3614	Y37.8969
N6502	X14.3267	Y37.9840
N6504	X14.2918	Y38.0706
N6506	X14.2568	Y38.1569
N6508	X14.2217	Y38.2427
N6510	X14.1865	Y38.3283
N6512	X14.1511	Y38.4134
N6514	X14.1156	Y38.4982
N6516	X14.0800	Y38.5827
N6518	X14.0442	Y38.6668
N6520	X14.0083	Y38.7506
N6522	X13.9723	Y38.8340
N6524	X13.9361	Y38.9172
N6526	X13.8999	Y39.0000
N6528	X13.8635	Y39.0825
N6530	X13.8269	Y39.1647
N6532	X13.7903	Y39.2466
N6534	X13.7535	Y39.3282
N6536	X13.7166	Y39.4095
N6538	X13.6796	Y39.4906
N6540	X13.6425	Y39.5714
N6542	X13.6053	Y39.6519
N6544	X13.5680	Y39.7321
N6546	X13.5305	Y39.8121

N6548	X13.4929	Y39.8918
N6550	X13.4552	Y39.9714
N6552	X13.4174	Y40.0506
N6554	X13.3795	Y40.1296
N6556	X13.3415	Y40.2085
N6558	X13.3034	Y40.2870
N6560	X13.2652	Y40.3654
N6562	X13.2269	Y40.4436
N6564	X13.1884	Y40.5216
N6566	X13.1499	Y40.5994
N6568	X13.1113	Y40.6769
N6570	X13.0726	Y40.7544
N6572	X13.0337	Y40.8316
N6574	X12.9948	Y40.9087
N6576	X12.9558	Y40.9856
N6578	X12.9167	Y41.0623
N6580	X12.8775	Y41.1389
N6582	X12.8382	Y41.2154
N6584	X12.7988	Y41.2917
N6586	X12.7593	Y41.3679
N6588	X12.7197	Y41.4439
N6590	X12.6800	Y41.5199
N6592	X12.6403	Y41.5957
N6594	X12.6004	Y41.6714
N6596	X12.5605	Y41.7470
N6598	X12.5205	Y41.8225
N6600	X12.4804	Y41.8980
N6602	X12.4403	Y41.9733
N6604	X12.4000	Y42.0486

N6606	X12.3597	Y42.1238
N6608	X12.3193	Y42.1989
N6610	X12.2788	Y42.2740
N6612	X12.2382	Y42.3490
N6614	X12.1976	Y42.4240
N6616	X12.1569	Y42.4990
N6618	X12.1161	Y42.5739
N6620	X12.0753	Y42.6487
N6622	X12.0344	Y42.7236
N6624	X11.9934	Y42.7985
N6626	X11.9523	Y42.8733
N6628	X11.9112	Y42.9481
N6630	X11.8700	Y43.0229
N6632	X11.8288	Y43.0978
N6634	X11.7875	Y43.1726
N6636	X11.7461	Y43.2475
N6638	X11.7046	Y43.3224
N6640	X11.6631	Y43.3974
N6642	X11.6216	Y43.4724
N6644	X11.5800	Y43.5474
N6646	X11.5383	Y43.6225
N6648	X11.4966	Y43.6976
N6650	X11.4548	Y43.7728
N6652	X11.4130	Y43.8481
N6654	X11.3711	Y43.9234
N6656	X11.3292	Y43.9989
N6658	X11.2872	Y44.0744
N6660	X11.2452	Y44.1500
N6662	X11.2031	Y44.2257

N6664 X11.1610 Y44.3015

N6666 X11.1188 Y44.3775

N6668 X11.0766 Y44.4535

N6670 X11.0344 Y44.5297

N6672 X10.9921 Y44.6060

N6674 X10.9497 Y44.6825

N6676 X10.9074 Y44.7591

N6678 X10.8650 Y44.8358

N6680 X10.8225 Y44.9128

N6682 X10.7800 Y44.9898

N6684 X10.7375 Y45.0671

N6686 X10.6950 Y45.1445

N6688 X10.6524 Y45.2221

N6690 X10.6098 Y45.2999

N6692 X10.5671 Y45.3778

N6694 X10.5245 Y45.4560

N6696 X10.4818 Y45.5344

N6698 X10.4390 Y45.6130

N6700 X10.3963 Y45.6918

N6702 X10.3535 Y45.7708

N6704 X10.3107 Y45.8501

N6706 X10.2679 Y45.9296

N6708 X10.2251 Y46.0093

N6710 X10.1822 Y46.0893

N6712 X10.1393 Y46.1696

N6714 X10.0964 Y46.2501

N6716 X10.0535 Y46.3309

N6718 X10.0106 Y46.4119

N6720 X9.9677 Y46.4932

N6722 X9.9247 Y46.5749

N6724 X9.8818 Y46.6568

N6726 X9.8388 Y46.7390

N6728 X9.7958 Y46.8215

N6730 X9.7528 Y46.9043

N6732 X9.7098 Y46.9874

N6734 X9.6668 Y47.0709

N6736 X9.6238 Y47.1547

N6738 X9.5808 Y47.2388

N6740 X9.5378 Y47.3233

N6742 X9.4948 Y47.4081

N6744 X9.4518 Y47.4932

N6746 X9.4088 Y47.5788

N6748 X9.3657 Y47.6647

N6750 X9.3227 Y47.7509

N6752 X9.2797 Y47.8376

N6754 X9.2367 Y47.9246

N6756 X9.1937 Y48.0120

N6758 X9.1508 Y48.0998

N6760 X9.1078 Y48.1880

N6762 X9.0649 Y48.2766

N6764 X9.0219 Y48.3655

N6766 X8.9790 Y48.4549

N6768 X8.9362 Y48.5446

N6770 X8.8934 Y48.6346

N6772 X8.8506 Y48.7250

N6774 X8.8079 Y48.8158

N6776 X8.7653 Y48.9069

N6778 X8.7228 Y48.9983

N6780 X8.6803 Y49.0901

N6782 X8.6379 Y49.1821

N6784 X8.5956 Y49.2745

N6786 X8.5534 Y49.3672

N6788 X8.5112 Y49.4602

N6790 X8.4693 Y49.5535

N6792 X8.4274 Y49.6471

N6794 X8.3856 Y49.7409

N6796 X8.3440 Y49.8350

N6798 X8.3025 Y49.9294

N6800 X8.2611 Y50.0240

N6802 X8.2199 Y50.1189

N6804 X8.1789 Y50.2141

N6806 X8.1380 Y50.3094

N6808 X8.0972 Y50.4051

N6810 X8.0567 Y50.5009

N6812 X8.0163 Y50.5969

N6814 X7.9761 Y50.6932

N6816 X7.9361 Y50.7897

N6818 X7.8964 Y50.8863

N6820 X7.8568 Y50.9832

N6822 X7.8174 Y51.0803

N6824 X7.7782 Y51.1775

N6826 X7.7393 Y51.2749

N6828 X7.7006 Y51.3724

N6830 X7.6621 Y51.4702

N6832 X7.6239 Y51.5680

N6834 X7.5860 Y51.6660

N6836 X7.5482 Y51.7642

N6838 X7.5108 Y51.8625

N6840 X7.4736 Y51.9609

N6842 X7.4367 Y52.0594

N6844 X7.4001 Y52.1581

N6846 X7.3638 Y52.2568

N6848 X7.3277 Y52.3557

N6850 X7.2920 Y52.4546

N6852 X7.2566 Y52.5536

N6854 X7.2215 Y52.6527

N6856 X7.1867 Y52.7519

N6858 X7.1522 Y52.8512

N6860 X7.1181 Y52.9505

N6862 X7.0843 Y53.0498

N6864 X7.0508 Y53.1492

N6866 X7.0178 Y53.2486

N6868 X6.9850 Y53.3481

N6870 X6.9527 Y53.4476

N6872 X6.9207 Y53.5471

N6874 X6.8891 Y53.6466

N6876 X6.8578 Y53.7461

N6878 X6.8270 Y53.8457

N6880 X6.7966 Y53.9452

N6882 X6.7665 Y54.0447

N6884 X6.7369 Y54.1442

N6886 X6.7077 Y54.2436

N6888 X6.6789 Y54.3430

N6890 X6.6506 Y54.4424

N6892 X6.6226 Y54.5417

N6894 X6.5952 Y54.6410

N6896 X6.5681 Y54.7402

N6898 X6.5416 Y54.8394

N6900 X6.5155 Y54.9384

N6902 X6.4898 Y55.0374

N6904 X6.4647 Y55.1363

N6906 X6.4400 Y55.2351

N6908 X6.4158 Y55.3338

N6910 X6.3921 Y55.4324

N6912 X6.3689 Y55.5309

N6914 X6.3462 Y55.6292

N6916 X6.3240 Y55.7274

N6918 X6.3023 Y55.8255

N6920 X6.2812 Y55.9235

N6922 X6.2606 Y56.0213

N6924 X6.2405 Y56.1189

N6926 X6.2210 Y56.2164

N6928 X6.2021 Y56.3137

N6930 X6.1837 Y56.4109

N6932 X6.1658 Y56.5078

N6934 X6.1485 Y56.6046

N6936 X6.1318 Y56.7012

N6938 X6.1157 Y56.7975

N6940 X6.1002 Y56.8937

N6942 X6.0853 Y56.9896

N6944 X6.0710 Y57.0854

N6946 X6.0573 Y57.1809

N6948 X6.0442 Y57.2761

N6950 X6.0317 Y57.3711

N6952 X6.0198 Y57.4659

N6954 X6.0086 Y57.5604

N6956 X5.9981 Y57.6547

N6958 X5.9881 Y57.7487

N6960 X5.9789 Y57.8424

N6962 X5.9703 Y57.9358

N6964 X5.9623 Y58.0290

N6966 X5.9551 Y58.1218

N6968 X5.9485 Y58.2143

N6970 X5.9426 Y58.3066

N6972 X5.9374 Y58.3985

N6974 X5.9329 Y58.4901

N6976 X5.9291 Y58.5814

N6978 X5.9260 Y58.6723

N6980 X5.9236 Y58.7629

N6982 X5.9220 Y58.8531

N6984 X5.9210 Y58.9430

N6986 X5.9209 Y59.0325

N6988 X5.9214 Y59.1217

N6990 X5.9227 Y59.2105

N6992 X5.9248 Y59.2989

N6994 X5.9277 Y59.3869

N6996 X5.9313 Y59.4745

N6998 X5.9356 Y59.5617

N7000 X5.9408 Y59.6485

N7002 X5.9468 Y59.7349

N7004 X5.9535 Y59.8209

N7006 X5.9611 Y59.9064

N7008 X5.9694 Y59.9915

N7010 X5.9786 Y60.0762

N7012 X5.9886 Y60.1604

N7014 X5.9994 Y60.2441

N7016 X6.0111 Y60.3274

N7018 X6.0236 Y60.4102

N7020 X6.0369 Y60.4926

N7022 X6.0511 Y60.5744

N7024 X6.0661 Y60.6558

N7026 X6.0821 Y60.7367

N7028 X6.0989 Y60.8171

N7030 X6.1165 Y60.8970

N7032 X6.1351 Y60.9763

N7034 X6.1545 Y61.0551

N7036 X6.1749 Y61.1334

N7038 X6.1961 Y61.2112

N7040 X6.2183 Y61.2884

N7042 X6.2414 Y61.3651

N7044 X6.2654 Y61.4412

N7046 X6.2903 Y61.5168

N7048 X6.3162 Y61.5918

N7050 X6.3430 Y61.6662

N7052 X6.3707 Y61.7400

N7054 X6.3994 Y61.8133

N7056 X6.4291 Y61.8859

N7058 X6.4598 Y61.9580

N7060 X6.4914 Y62.0294

N7062 X6.5240 Y62.1002

N7064 X6.5576 Y62.1704

N7066 X6.5922 Y62.2400

N7068 X6.6277 Y62.3089

N7070 X6.6643 Y62.3772

N7072 X6.7019 Y62.4448

N7074 X6.7406 Y62.5118

N7076 X6.7802 Y62.5781

N7078 X6.8209 Y62.6438

N7080 X6.8626 Y62.7088

N7082 X6.9054 Y62.7731

N7084 X6.9492 Y62.8367

N7086 X6.9941 Y62.8996

N7088 X7.0400 Y62.9618

N7090 X7.0870 Y63.0233

N7092 X7.1351 Y63.0841

N7094 X7.1843 Y63.1441

N7096 X7.2345 Y63.2035

N7098 X7.2859 Y63.2621

N7100 X7.3383 Y63.3199

N7102 X7.3919 Y63.3770

N7104 X7.4466 Y63.4334

N7106 X7.5024 Y63.4890

N7108 X7.5593 Y63.5438

N7110 X7.6173 Y63.5979

N7112 X7.6764 Y63.6512

N7114 X7.7365 Y63.7037

N7116 X7.7977 Y63.7556

N7118 X7.8599 Y63.8066

N7120 X7.9231 Y63.8569

N7122 X7.9873 Y63.9065

N7124 X8.0525 Y63.9554

N7126 X8.1187 Y64.0035

N7128 X8.1858 Y64.0508

N7130 X8.2539 Y64.0974

N7132 X8.3229 Y64.1433

N7134 X8.3928 Y64.1885

N7136 X8.4636 Y64.2329

N7138 X8.5353 Y64.2767

N7140 X8.6079 Y64.3196

N7142 X8.6813 Y64.3619

N7144 X8.7556 Y64.4035

N7146 X8.8307 Y64.4443

N7148 X8.9066 Y64.4844

N7150 X8.9833 Y64.5238

N7152 X9.0608 Y64.5625

N7154 X9.1390 Y64.6005

N7156 X9.2180 Y64.6377

N7158 X9.2978 Y64.6743

N7160 X9.3782 Y64.7102

N7162 X9.4594 Y64.7453

N7164 X9.5413 Y64.7798

N7166 X9.6238 Y64.8136

N7168 X9.7070 Y64.8467

N7170 X9.7909 Y64.8791

N7172 X9.8754 Y64.9108

N7174 X9.9605 Y64.9418

N7176 X10.0462 Y64.9721

N7178 X10.1325 Y65.0018

N7180 X10.2194 Y65.0307

N7182 X10.3069 Y65.0590

N7184 X10.3949 Y65.0866

N7186	X10.4834	Y65.1136
N7188	X10.5725	Y65.1398
N7190	X10.6620	Y65.1654
N7192	X10.7521	Y65.1904
N7194	X10.8426	Y65.2146
N7196	X10.9336	Y65.2382
N7198	X11.0250	Y65.2612
N7200	X11.1168	Y65.2835
N7202	X11.2091	Y65.3051
N7204	X11.3018	Y65.3261
N7206	X11.3949	Y65.3464
N7208	X11.4883	Y65.3661
N7210	X11.5821	Y65.3851
N7212	X11.6762	Y65.4035
N7214	X11.7707	Y65.4212
N7216	X11.8655	Y65.4383
N7218	X11.9606	Y65.4548
N7220	X12.0559	Y65.4706
N7222	X12.1516	Y65.4858
N7224	X12.2474	Y65.5003
N7226	X12.3436	Y65.5142
N7228	X12.4399	Y65.5275
N7230	X12.5365	Y65.5402
N7232	X12.6333	Y65.5523
N7234	X12.7302	Y65.5637
N7236	X12.8273	Y65.5745
N7238	X12.9246	Y65.5847
N7240	X13.0220	Y65.5942
N7242	X13.1195	Y65.6032

N7244	X13.2172	Y65.6116
N7246	X13.3149	Y65.6193
N7248	X13.4127	Y65.6264
N7250	X13.5106	Y65.6330
N7252	X13.6085	Y65.6389
N7254	X13.7065	Y65.6442
N7256	X13.8045	Y65.6489
N7258	X13.9025	Y65.6531
N7260	X14.0005	Y65.6566
N7262	X14.0984	Y65.6596
N7264	X14.1964	Y65.6619
N7266	X14.2943	Y65.6637
N7268	X14.3921	Y65.6649
N7270	X14.4898	Y65.6655
N7272	X14.5874	Y65.6656
N7274	X14.6850	Y65.6650
N7276	X14.7824	Y65.6639
N7278	X14.8796	Y65.6622
N7280	X14.9767	Y65.6599
N7282	X15.0737	Y65.6571
N7284	X15.1704	Y65.6537
N7286	X15.2670	Y65.6497
N7288	X15.3634	Y65.6452
N7290	X15.4597	Y65.6401
N7292	X15.5557	Y65.6345
N7294	X15.6516	Y65.6283
N7296	X15.7473	Y65.6215
N7298	X15.8428	Y65.6143
N7300	X15.9382	Y65.6064

N7302	X16.0333	Y65.5981
N7304	X16.1283	Y65.5892
N7306	X16.2232	Y65.5798
N7308	X16.3178	Y65.5699
N7310	X16.4123	Y65.5594
N7312	X16.5066	Y65.5484
N7314	X16.6007	Y65.5369
N7316	X16.6946	Y65.5249
N7318	X16.7884	Y65.5124
N7320	X16.8820	Y65.4994
N7322	X16.9754	Y65.4858
N7324	X17.0686	Y65.4718
N7326	X17.1617	Y65.4573
N7328	X17.2546	Y65.4423
N7330	X17.3473	Y65.4268
N7332	X17.4398	Y65.4108
N7334	X17.5322	Y65.3943
N7336	X17.6244	Y65.3774
N7338	X17.7164	Y65.3599
N7340	X17.8083	Y65.3420
N7342	X17.8999	Y65.3237
N7344	X17.9914	Y65.3048
N7346	X18.0828	Y65.2856
N7348	X18.1739	Y65.2658
N7350	X18.2649	Y65.2456
N7352	X18.3557	Y65.2249
N7354	X18.4464	Y65.2038
N7356	X18.5368	Y65.1823
N7358	X18.6271	Y65.1603

N7360	X18.7173	Y65.1379
N7362	X18.8072	Y65.1150
N7364	X18.8970	Y65.0917
N7366	X18.9866	Y65.0680
N7368	X19.0761	Y65.0439
N7370	X19.1653	Y65.0193
N7372	X19.2544	Y64.9943
N7374	X19.3433	Y64.9689
N7376	X19.4321	Y64.9431
N7378	X19.5207	Y64.9169
N7380	X19.6091	Y64.8903
N7382	X19.6974	Y64.8632
N7384	X19.7854	Y64.8358
N7386	X19.8733	Y64.8080
N7388	X19.9611	Y64.7798
N7390	X20.0486	Y64.7512
N7392	X20.1360	Y64.7222
N7394	X20.2233	Y64.6929
N7396	X20.3103	Y64.6631
N7398	X20.3972	Y64.6330
N7400	X20.4840	Y64.6025
N7402	X20.5705	Y64.5717
N7404	X20.6569	Y64.5405
N7406	X20.7431	Y64.5089
N7408	X20.8292	Y64.4770
N7410	X20.9151	Y64.4447
N7412	X21.0008	Y64.4121
N7414	X21.0863	Y64.3791
N7416	X21.1717	Y64.3458

N7418	X21.2569	Y64.3121
N7420	X21.3420	Y64.2782
N7422	X21.4269	Y64.2438
N7424	X21.5116	Y64.2092
N7426	X21.5961	Y64.1742
N7428	X21.6805	Y64.1389
N7430	X21.7647	Y64.1032
N7432	X21.8488	Y64.0673
N7434	X21.9327	Y64.0311
N7436	X22.0164	Y63.9945
N7438	X22.1000	Y63.9576
N7440	X22.1834	Y63.9204
N7442	X22.2666	Y63.8830
N7444	X22.3496	Y63.8452
N7446	X22.4325	Y63.8071
N7448	X22.5153	Y63.7688
N7450	X22.5978	Y63.7302
N7452	X22.6802	Y63.6912
N7454	X22.7625	Y63.6520
N7456	X22.8446	Y63.6126
N7458	X22.9265	Y63.5728
N7460	X23.0082	Y63.5328
N7462	X23.0898	Y63.4925
N7464	X23.1712	Y63.4520
N7466	X23.2525	Y63.4112
N7468	X23.3336	Y63.3701
N7470	X23.4145	Y63.3288
N7472	X23.4953	Y63.2873
N7474	X23.5759	Y63.2455

N7476	X23.6563	Y63.2034
N7478	X23.7366	Y63.1612
N7480	X23.8167	Y63.1186
N7482	X23.8967	Y63.0759
N7484	X23.9765	Y63.0329
N7486	X24.0561	Y62.9897
N7488	X24.1356	Y62.9463
N7490	X24.2149	Y62.9027
N7492	X24.2941	Y62.8588
N7494	X24.3731	Y62.8147
N7496	X24.4519	Y62.7705
N7498	X24.5306	Y62.7260
N7500	X24.6091	Y62.6813
N7502	X24.6874	Y62.6364
N7504	X24.7656	Y62.5914
N7506	X24.8437	Y62.5461
N7508	X24.9215	Y62.5006
N7510	X24.9993	Y62.4550
N7512	X25.0768	Y62.4092
N7514	X25.1542	Y62.3632
N7516	X25.2314	Y62.3170
N7518	X25.3085	Y62.2707
N7520	X25.3854	Y62.2242
N7522	X25.4622	Y62.1775
N7524	X25.5388	Y62.1307
N7526	X25.6152	Y62.0837
N7528	X25.6915	Y62.0366
N7530	X25.7677	Y61.9893
N7532	X25.8436	Y61.9419

N7534	X25.9194	Y61.8943
N7536	X25.9951	Y61.8466
N7538	X26.0706	Y61.7987
N7540	X26.1459	Y61.7507
N7542	X26.2211	Y61.7026
N7544	X26.2962	Y61.6543
N7546	X26.3710	Y61.6060
N7548	X26.4457	Y61.5575
N7550	X26.5203	Y61.5089
N7552	X26.5947	Y61.4602
N7554	X26.6689	Y61.4113
N7556	X26.7430	Y61.3624
N7558	X26.8170	Y61.3134
N7560	X26.8908	Y61.2642
N7562	X26.9644	Y61.2150
N7564	X27.0378	Y61.1657
N7566	X27.1112	Y61.1162
N7568	X27.1843	Y61.0667
N7570	X27.2573	Y61.0171
N7572	X27.3302	Y60.9675
N7574	X27.4029	Y60.9177
N7576	X27.4754	Y60.8679
N7578	X27.5478	Y60.8180
N7580	X27.6200	Y60.7681
N7582	X27.6921	Y60.7181
N7584	X27.7641	Y60.6680
N7586	X27.8358	Y60.6179
N7588	X27.9075	Y60.5677
N7590	X27.9789	Y60.5175

N7592	X28.0502	Y60.4672
N7594	X28.1214	Y60.4169
N7596	X28.1924	Y60.3666
N7598	X28.2633	Y60.3162
N7600	X28.3340	Y60.2657
N7602	X28.4045	Y60.2153
N7604	X28.4749	Y60.1648
N7606	X28.5452	Y60.1143
N7608	X28.6153	Y60.0638
N7610	X28.6852	Y60.0133
N7612	X28.7550	Y59.9628
N7614	X28.8247	Y59.9122
N7616	X28.8942	Y59.8617
N7618	X28.9635	Y59.8111
N7620	X29.0327	Y59.7605
N7622	X29.1018	Y59.7100
N7624	X29.1707	Y59.6595
N7626	X29.2394	Y59.6089
N7628	X29.3080	Y59.5584
N7630	X29.3765	Y59.5079
N7632	X29.4447	Y59.4575
N7634	X29.5129	Y59.4070
N7636	X29.5809	Y59.3566
N7638	X29.6487	Y59.3062
N7640	X29.7164	Y59.2559
N7642	X29.7840	Y59.2056
N7644	X29.8514	Y59.1553
N7646	X29.9187	Y59.1051
N7648	X29.9858	Y59.0549

N7650	X30.0528	Y59.0047
N7652	X30.1196	Y58.9546
N7654	X30.1863	Y58.9045
N7656	X30.2530	Y58.8545
N7658	X30.3195	Y58.8045
N7660	X30.3858	Y58.7545
N7662	X30.4521	Y58.7045
N7664	X30.5183	Y58.6546
N7666	X30.5843	Y58.6047
N7668	X30.6503	Y58.5548
N7670	X30.7162	Y58.5049
N7672	X30.7820	Y58.4551
N7674	X30.8477	Y58.4053
N7676	X30.9134	Y58.3555
N7678	X30.9789	Y58.3057
N7680	X31.0444	Y58.2559
N7682	X31.1098	Y58.2061
N7684	X31.1752	Y58.1564
N7686	X31.2405	Y58.1066
N7688	X31.3058	Y58.0569
N7690	X31.3710	Y58.0072
N7692	X31.4361	Y57.9575
N7694	X31.5013	Y57.9078
N7696	X31.5663	Y57.8580
N7698	X31.6314	Y57.8083
N7700	X31.6964	Y57.7586
N7702	X31.7614	Y57.7089
N7704	X31.8264	Y57.6592
N7706	X31.8914	Y57.6095

N7708	X31.9563	Y57.5598
N7710	X32.0213	Y57.5101
N7712	X32.0862	Y57.4604
N7714	X32.1511	Y57.4107
N7716	X32.2161	Y57.3610
N7718	X32.2810	Y57.3112
N7720	X32.3460	Y57.2615
N7722	X32.4109	Y57.2117
N7724	X32.4759	Y57.1619
N7726	X32.5409	Y57.1122
N7728	X32.6060	Y57.0624
N7730	X32.6710	Y57.0125
N7732	X32.7361	Y56.9627
N7734	X32.8013	Y56.9129
N7736	X32.8664	Y56.8630
N7738	X32.9317	Y56.8131
N7740	X32.9969	Y56.7632
N7742	X33.0623	Y56.7133
N7744	X33.1276	Y56.6633
N7746	X33.1931	Y56.6134
N7748	X33.2586	Y56.5634
N7750	X33.3241	Y56.5133
N7752	X33.3898	Y56.4633
N7754	X33.4555	Y56.4132
N7756	X33.5213	Y56.3631
N7758	X33.5872	Y56.3130
N7760	X33.6531	Y56.2628
N7762	X33.7191	Y56.2126
N7764	X33.7853	Y56.1624

N7766	X33.8515	Y56.1121
N7768	X33.9178	Y56.0618
N7770	X33.9842	Y56.0115
N7772	X34.0508	Y55.9612
N7774	X34.1174	Y55.9108
N7776	X34.1842	Y55.8603
N7778	X34.2510	Y55.8099
N7780	X34.3180	Y55.7594
N7782	X34.3851	Y55.7088
N7784	X34.4523	Y55.6582
N7786	X34.5197	Y55.6076
N7788	X34.5872	Y55.5570
N7790	X34.6548	Y55.5063
N7792	X34.7225	Y55.4555
N7794	X34.7904	Y55.4047
N7796	X34.8585	Y55.3539
N7798	X34.9267	Y55.3030
N7800	X34.9950	Y55.2521
N7802	X35.0635	Y55.2012
N7804	X35.1321	Y55.1502
N7806	X35.2009	Y55.0991
N7808	X35.2699	Y55.0480
N7810	X35.3390	Y54.9969
N7812	X35.4083	Y54.9457
N7814	X35.4778	Y54.8944
N7816	X35.5474	Y54.8432
N7818	X35.6172	Y54.7918
N7820	X35.6872	Y54.7405
N7822	X35.7574	Y54.6890

N7824	X35.8277	Y54.6375
N7826	X35.8982	Y54.5860
N7828	X35.9690	Y54.5344
N7830	X36.0399	Y54.4828
N7832	X36.1110	Y54.4311
N7834	X36.1823	Y54.3794
N7836	X36.2538	Y54.3276
N7838	X36.3256	Y54.2758
N7840	X36.3975	Y54.2239
N7842	X36.4696	Y54.1720
N7844	X36.5419	Y54.1200
N7846	X36.6145	Y54.0679
N7848	X36.6872	Y54.0158
N7850	X36.7602	Y53.9637
N7852	X36.8334	Y53.9115
N7854	X36.9068	Y53.8592
N7856	X36.9805	Y53.8069
N7858	X37.0544	Y53.7546
N7860	X37.1285	Y53.7021
N7862	X37.2028	Y53.6497
N7864	X37.2773	Y53.5971
N7866	X37.3521	Y53.5446
N7868	X37.4272	Y53.4919
N7870	X37.5024	Y53.4392
N7872	X37.5780	Y53.3865
N7874	X37.6537	Y53.3337
N7876	X37.7297	Y53.2809
N7878	X37.8060	Y53.2280
N7880	X37.8825	Y53.1750

N7882	X37.9592	Y53.1220
N7884	X38.0362	Y53.0689
N7886	X38.1135	Y53.0158
N7888	X38.1910	Y52.9626
N7890	X38.2687	Y52.9094
N7892	X38.3468	Y52.8561
N7894	X38.4250	Y52.8028
N7896	X38.5036	Y52.7494
N7898	X38.5824	Y52.6960
N7900	X38.6615	Y52.6425
N7902	X38.7408	Y52.5889
N7904	X38.8205	Y52.5353
N7906	X38.9003	Y52.4817
N7908	X38.9805	Y52.4280
N7910	X39.0609	Y52.3742
N7912	X39.1416	Y52.3204
N7914	X39.2226	Y52.2666
N7916	X39.3039	Y52.2127
N7918	X39.3854	Y52.1587
N7920	X39.4672	Y52.1047
N7922	X39.5493	Y52.0507
N7924	X39.6317	Y51.9966
N7926	X39.7143	Y51.9424
N7928	X39.7973	Y51.8882
N7930	X39.8805	Y51.8340
N7932	X39.9640	Y51.7797
N7934	X40.0478	Y51.7253
N7936	X40.1319	Y51.6709
N7938	X40.2163	Y51.6165

N7940	X40.3009	Y51.5620
N7942	X40.3859	Y51.5075
N7944	X40.4711	Y51.4529
N7946	X40.5567	Y51.3983
N7948	X40.6425	Y51.3437
N7950	X40.7286	Y51.2890
N7952	X40.8150	Y51.2342
N7954	X40.9017	Y51.1794
N7956	X40.9887	Y51.1246
N7958	X41.0760	Y51.0697
N7960	X41.1636	Y51.0148
N7962	X41.2514	Y50.9599
N7964	X41.3396	Y50.9049
N7966	X41.4281	Y50.8499
N7968	X41.5169	Y50.7948
N7970	X41.6059	Y50.7398
N7972	X41.6953	Y50.6846
N7974	X41.7849	Y50.6295
N7976	X41.8749	Y50.5743
N7978	X41.9651	Y50.5190
N7980	X42.0557	Y50.4638
N7982	X42.1465	Y50.4085
N7984	X42.2377	Y50.3532
N7986	X42.3291	Y50.2978
N7988	X42.4208	Y50.2424
N7990	X42.5128	Y50.1870
N7992	X42.6052	Y50.1316
N7994	X42.6978	Y50.0761
N7996	X42.7907	Y50.0206

N7998	X42.8839	Y49.9651
N8000	X42.9774	Y49.9096
N8002	X43.0712	Y49.8540
N8004	X43.1653	Y49.7984
N8006	X43.2597	Y49.7428
N8008	X43.3543	Y49.6872
N8010	X43.4493	Y49.6315
N8012	X43.5445	Y49.5759
N8014	X43.6401	Y49.5202
N8016	X43.7359	Y49.4645
N8018	X43.8320	Y49.4088
N8020	X43.9285	Y49.3531
N8022	X44.0251	Y49.2973
N8024	X44.1221	Y49.2416
N8026	X44.2194	Y49.1858
N8028	X44.3170	Y49.1300
N8030	X44.4148	Y49.0742
N8032	X44.5129	Y49.0185
N8034	X44.6113	Y48.9627
N8036	X44.7100	Y48.9068
N8038	X44.8090	Y48.8510
N8040	X44.9082	Y48.7952
N8042	X45.0077	Y48.7394
N8044	X45.1075	Y48.6836
N8046	X45.2076	Y48.6278
N8048	X45.3079	Y48.5720
N8050	X45.4085	Y48.5162
N8052	X45.5094	Y48.4604
N8054	X45.6104	Y48.4047

N8056	X45.7116	Y48.3491
N8058	X45.8129	Y48.2936
N8060	X45.9143	Y48.2384
N8062	X46.0158	Y48.1833
N8064	X46.1173	Y48.1285
N8066	X46.2188	Y48.0739
N8068	X46.3203	Y48.0197
N8070	X46.4216	Y47.9658
N8072	X46.5229	Y47.9122
N8074	X46.6241	Y47.8591
N8076	X46.7250	Y47.8064
N8078	X46.8258	Y47.7541
N8080	X46.9264	Y47.7024
N8082	X47.0267	Y47.6511
N8084	X47.1267	Y47.6005
N8086	X47.2265	Y47.5504
N8088	X47.3259	Y47.5009
N8090	X47.4250	Y47.4520
N8092	X47.5236	Y47.4038
N8094	X47.6219	Y47.3563
N8096	X47.7198	Y47.3095
N8098	X47.8172	Y47.2634
N8100	X47.9141	Y47.2181
N8102	X48.0105	Y47.1737
N8104	X48.1065	Y47.1300
N8106	X48.2019	Y47.0872
N8108	X48.2967	Y47.0452
N8110	X48.3909	Y47.0042
N8112	X48.4846	Y46.9641

N8114	X48.5776	Y46.9249
N8116	X48.6700	Y46.8867
N8118	X48.7618	Y46.8495
N8120	X48.8529	Y46.8133
N8122	X48.9433	Y46.7782
N8124	X49.0330	Y46.7441
N8126	X49.1219	Y46.7112
N8128	X49.2101	Y46.6793
N8130	X49.2976	Y46.6486
N8132	X49.3843	Y46.6191
N8134	X49.4702	Y46.5908
N8136	X49.5553	Y46.5637
N8138	X49.6396	Y46.5378
N8140	X49.7230	Y46.5132
N8142	X49.8056	Y46.4900
N8144	X49.8873	Y46.4680
N8146	X49.9682	Y46.4474
N8148	X50.0481	Y46.4281
N8150	X50.1272	Y46.4103
N8152	X50.2053	Y46.3938
N8154	X50.2825	Y46.3789
N8156	X50.3587	Y46.3654
N8158	X50.4340	Y46.3534
N8160	X50.5083	Y46.3429
N8162	X50.5816	Y46.3340
N8164	X50.6539	Y46.3267
N8166	X50.7252	Y46.3210
N8168	X50.7955	Y46.3170
N8170	X50.8647	Y46.3146

N8172	X50.9329	Y46.3140
N8174	X51.0000	Y46.3151
N8176	X51.0660	Y46.3179
N8178	X51.1309	Y46.3226
N8180	X51.1947	Y46.3291
N8182	X51.2573	Y46.3375
N8184	X51.3188	Y46.3478
N8186	X51.3792	Y46.3600
N8188	X51.4384	Y46.3742
N8190	X51.4964	Y46.3905
N8192	X51.5532	Y46.4088
N8194	X51.6087	Y46.4292
N8196	X51.6631	Y46.4518
N8198	X51.7162	Y46.4765
N8200	X51.7680	Y46.5035
N8202	X51.8185	Y46.5327
N8204	X51.8677	Y46.5643
N8206	X51.9157	Y46.5983
N8208	X51.9622	Y46.6346
N8210	X52.0074	Y46.6735
N8212	X52.0512	Y46.7148
N8214	X52.0937	Y46.7588
N8216	X52.1347	Y46.8054
N8218	X52.1742	Y46.8547
N8220	X52.2123	Y46.9068
N8222	X52.2489	Y46.9617
N8224	X52.2840	Y47.0194
N8226	X52.3177	Y47.0800
N8228	X52.3500	Y47.1434

N8230	X52.3810	Y47.2096
N8232	X52.4109	Y47.2785
N8234	X52.4396	Y47.3501
N8236	X52.4672	Y47.4243
N8238	X52.4939	Y47.5011
N8240	X52.5196	Y47.5805
N8242	X52.5444	Y47.6623
N8244	X52.5685	Y47.7466
N8246	X52.5918	Y47.8333
N8248	X52.6145	Y47.9223
N8250	X52.6366	Y48.0136
N8252	X52.6582	Y48.1070
N8254	X52.6794	Y48.2026
N8256	X52.7002	Y48.3003
N8258	X52.7206	Y48.3999
N8260	X52.7409	Y48.5014
N8262	X52.7610	Y48.6047
N8264	X52.7810	Y48.7097
N8266	X52.8010	Y48.8163
N8268	X52.8210	Y48.9244
N8270	X52.8412	Y49.0339
N8272	X52.8616	Y49.1447
N8274	X52.8822	Y49.2566
N8276	X52.9032	Y49.3696
N8278	X52.9245	Y49.4834
N8280	X52.9464	Y49.5980
N8282	X52.9689	Y49.7130
N8284	X52.9920	Y49.8281
N8286	X53.0159	Y49.9432

N8288	X53.0406	Y50.0580
N8290	X53.0662	Y50.1721
N8292	X53.0929	Y50.2855
N8294	X53.1207	Y50.3977
N8296	X53.1497	Y50.5086
N8298	X53.1800	Y50.6178
N8300	X53.2117	Y50.7253
N8302	X53.2449	Y50.8306
N8304	X53.2796	Y50.9336
N8306	X53.3160	Y51.0339
N8308	X53.3542	Y51.1315
N8310	X53.3942	Y51.2260
N8312	X53.4362	Y51.3171
N8314	X53.4801	Y51.4047
N8316	X53.5262	Y51.4885
N8318	X53.5745	Y51.5683
N8320	X53.6251	Y51.6439
N8322	X53.6781	Y51.7150
N8324	X53.7336	Y51.7813
N8326	X53.7917	Y51.8428
N8328	X53.8524	Y51.8991
N8330	X53.9160	Y51.9500
N8332	X53.9823	Y51.9953
N8334	X54.0516	Y52.0349
N8336	X54.1240	Y52.0684
N8338	X54.1995	Y52.0957
N8340	X54.4930	Y52.1390
