# Supplementary material for: High-Speed Precision Machining and Surface Roughness Determination of Freeform Curves Using Galerkin-NURBS Interpolation and Jerk-Limited Trajectory Planning
Source: Sensors (Basel). 2026 Jul 13;26(14):4441. doi: 10.3390/s26144441 (PMC13417550; doi:10.3390/s26144441)
Supplement: Supplementary file 1 [file sensors-26-04441-s001.zip › sensors-4388769-Supplementary S2.pdf]

```
N110    (Horse-contour)
N112    G00     G54     G90     X0.5600 Y128.7500
N114    G43     H1      Z135.5
N116    Z40.5
N118    Z17.
N120    G01     Z15.
N122    X0.6356 Y128.7560
N124    X0.7109 Y128.7622
N126    X0.7859 Y128.7684
N128    X0.8607 Y128.7748
N130    X0.9353 Y128.7813
N132    X1.0096 Y128.7879
N134    X1.0836 Y128.7946
N136    X1.1574 Y128.8014
N138    X1.2310 Y128.8083
N140    X1.3043 Y128.8154
N142    X1.3774 Y128.8226
N144    X1.4503 Y128.8299
N146    X1.5229 Y128.8373
N148    X1.5953 Y128.8448
N150    X1.6675 Y128.8525
N152    X1.7394 Y128.8602
N154    X1.8112 Y128.8681
N156    X1.8826 Y128.8761
N158    X1.9539 Y128.8841
N160    X2.0250 Y128.8924
N162    X2.0958 Y128.9007
N164    X2.1665 Y128.9091
N166    X2.2369 Y128.9177
```

N168 X2.3071 Y128.9263

N170 X2.3771 Y128.9351

N172 X2.4469 Y128.9440

N174 X2.5165 Y128.9530

N176 X2.5859 Y128.9621

N178 X2.6551 Y128.9714

N180 X2.7241 Y128.9807

N182 X2.7929 Y128.9902

N184 X2.8615 Y128.9998

N186 X2.9299 Y129.0094

N188 X2.9981 Y129.0192

N190 X3.0662 Y129.0291

N192 X3.1340 Y129.0392

N194 X3.2017 Y129.0493

N196 X3.2692 Y129.0596

N198 X3.3366 Y129.0699

N200 X3.4037 Y129.0804

N202 X3.4707 Y129.0910

N204 X3.5375 Y129.1017

N206 X3.6041 Y129.1125

N208 X3.6706 Y129.1234

N210 X3.7369 Y129.1344

N212 X3.8030 Y129.1456

N214 X3.8690 Y129.1568

N216 X3.9348 Y129.1682

N218 X4.0005 Y129.1796

N220 X4.0660 Y129.1912

N222 X4.1313 Y129.2029

N224 X4.1965 Y129.2147

N226 X4.2616 Y129.2266

N228 X4.3265 Y129.2387

N230 X4.3913 Y129.2508

N232 X4.4559 Y129.2631

N234 X4.5204 Y129.2754

N236 X4.5847 Y129.2879

N238 X4.6489 Y129.3005

N240 X4.7130 Y129.3131

N242 X4.7770 Y129.3259

N244 X4.8408 Y129.3388

N246 X4.9045 Y129.3519

N248 X4.9680 Y129.3650

N250 X5.0315 Y129.3782

N252 X5.0948 Y129.3915

N254 X5.1580 Y129.4050

N256 X5.2211 Y129.4186

N258 X5.2840 Y129.4322

N260 X5.3469 Y129.4460

N262 X5.4097 Y129.4599

N264 X5.4723 Y129.4739

N266 X5.5348 Y129.4880

N268 X5.5973 Y129.5022

N270 X5.6596 Y129.5165

N272 X5.7218 Y129.5309

N274 X5.7839 Y129.5454

N276 X5.8460 Y129.5601

N278 X5.9079 Y129.5748

N280 X5.9698 Y129.5897

N282 X6.0315 Y129.6046

N284 X6.0932 Y129.6197

N286 X6.1548 Y129.6349

N288 X6.2163 Y129.6502

N290 X6.2777 Y129.6655

N292 X6.3391 Y129.6810

N294 X6.4004 Y129.6966

N296 X6.4615 Y129.7123

N298 X6.5227 Y129.7281

N300 X6.5837 Y129.7441

N302 X6.6447 Y129.7601

N304 X6.7056 Y129.7762

N306 X6.7665 Y129.7925

N308 X6.8273 Y129.8088

N310 X6.8880 Y129.8252

N312 X6.9487 Y129.8418

N314 X7.0093 Y129.8584

N316 X7.0699 Y129.8752

N318 X7.1304 Y129.8921

N320 X7.1909 Y129.9090

N322 X7.2513 Y129.9261

N324 X7.3117 Y129.9433

N326 X7.3720 Y129.9606

N328 X7.4323 Y129.9780

N330 X7.4925 Y129.9955

N332 X7.5528 Y130.0131

N334 X7.6129 Y130.0308

N336 X7.6731 Y130.0486

N338 X7.7332 Y130.0665

N340 X7.7933 Y130.0845

N342 X7.8534 Y130.1026

N344 X7.9134 Y130.1208

N346 X7.9734 Y130.1392

N348 X8.0334 Y130.1576

N350 X8.0934 Y130.1761

N352 X8.1534 Y130.1948

N354 X8.2133 Y130.2135

N356 X8.2733 Y130.2323

N358 X8.3332 Y130.2513

N360 X8.3931 Y130.2703

N362 X8.4531 Y130.2895

N364 X8.5130 Y130.3087

N366 X8.5729 Y130.3281

N368 X8.6328 Y130.3475

N370 X8.6928 Y130.3671

N372 X8.7527 Y130.3867

N374 X8.8126 Y130.4065

N376 X8.8726 Y130.4263

N378 X8.9325 Y130.4463

N380 X8.9925 Y130.4664

N382 X9.0525 Y130.4865

N384 X9.1125 Y130.5068

N386 X9.1725 Y130.5272

N388 X9.2326 Y130.5476

N390 X9.2927 Y130.5682

N392 X9.3528 Y130.5889

N394 X9.4129 Y130.6096

N396 X9.4731 Y130.6305

N398 X9.5333 Y130.6515

N400 X9.5935 Y130.6725

N402 X9.6538 Y130.6937

N404 X9.7141 Y130.7150

N406 X9.7744 Y130.7363

N408 X9.8348 Y130.7578

N410 X9.8952 Y130.7794

N412 X9.9557 Y130.8011

N414 X10.0163 Y130.8228

N416 X10.0769 Y130.8447

N418 X10.1375 Y130.8667

N420 X10.1982 Y130.8887

N422 X10.2590 Y130.9109

N424 X10.3198 Y130.9331

N426 X10.3807 Y130.9555

N428 X10.4416 Y130.9780

N430 X10.5026 Y131.0005

N432 X10.5637 Y131.0232

N434 X10.6248 Y131.0459

N436 X10.6861 Y131.0688

N438 X10.7474 Y131.0917

N440 X10.8087 Y131.1148

N442 X10.8702 Y131.1379

N444 X10.9317 Y131.1612

N446 X10.9934 Y131.1845

N448 X11.0551 Y131.2079

N450 X11.1169 Y131.2315

N452 X11.1788 Y131.2551

N454 X11.2407 Y131.2788

N456 X11.3028 Y131.3027

N458	X11.3650	Y131.3266
N460	X11.4272	Y131.3506
N462	X11.4896	Y131.3747
N464	X11.5521	Y131.3989
N466	X11.6147	Y131.4232
N468	X11.6773	Y131.4476
N470	X11.7401	Y131.4721
N472	X11.8030	Y131.4967
N474	X11.8660	Y131.5214
N476	X11.9292	Y131.5462
N478	X11.9924	Y131.5711
N480	X12.0558	Y131.5961
N482	X12.1193	Y131.6211
N484	X12.1829	Y131.6463
N486	X12.2466	Y131.6716
N488	X12.3105	Y131.6969
N490	X12.3745	Y131.7224
N492	X12.4386	Y131.7479
N494	X12.5029	Y131.7736
N496	X12.5673	Y131.7993
N498	X12.6318	Y131.8252
N500	X12.6965	Y131.8511
N502	X12.7613	Y131.8771
N504	X12.8263	Y131.9032
N506	X12.8914	Y131.9294
N508	X12.9567	Y131.9557
N510	X13.0221	Y131.9821
N512	X13.0877	Y132.0086
N514	X13.1534	Y132.0352

N516	X13.2193	Y132.0619
N518	X13.2853	Y132.0886
N520	X13.3515	Y132.1155
N522	X13.4179	Y132.1425
N524	X13.4844	Y132.1695
N526	X13.5511	Y132.1966
N528	X13.6180	Y132.2239
N530	X13.6850	Y132.2512
N532	X13.7522	Y132.2786
N534	X13.8196	Y132.3061
N536	X13.8872	Y132.3337
N538	X13.9549	Y132.3614
N540	X14.0229	Y132.3892
N542	X14.0910	Y132.4171
N544	X14.1593	Y132.4451
N546	X14.2278	Y132.4731
N548	X14.2965	Y132.5013
N550	X14.3654	Y132.5295
N552	X14.4344	Y132.5579
N554	X14.5037	Y132.5863
N556	X14.5732	Y132.6148
N558	X14.6428	Y132.6434
N560	X14.7127	Y132.6721
N562	X14.7828	Y132.7009
N564	X14.8531	Y132.7298
N566	X14.9236	Y132.7587
N568	X14.9943	Y132.7878
N570	X15.0652	Y132.8170
N572	X15.1364	Y132.8462

N574	X15.2078	Y132.8755
N576	X15.2793	Y132.9049
N578	X15.3511	Y132.9345
N580	X15.4232	Y132.9641
N582	X15.4954	Y132.9937
N584	X15.5679	Y133.0235
N586	X15.6406	Y133.0534
N588	X15.7136	Y133.0833
N590	X15.7868	Y133.1134
N592	X15.8602	Y133.1435
N594	X15.9339	Y133.1737
N596	X16.0078	Y133.2041
N598	X16.0819	Y133.2345
N600	X16.1563	Y133.2649
N602	X16.2310	Y133.2955
N604	X16.3059	Y133.3262
N606	X16.3810	Y133.3569
N608	X16.4564	Y133.3878
N610	X16.5321	Y133.4187
N612	X16.6080	Y133.4497
N614	X16.6842	Y133.4808
N616	X16.7606	Y133.5120
N618	X16.8373	Y133.5433
N620	X16.9143	Y133.5747
N622	X16.9915	Y133.6061
N624	X17.0691	Y133.6377
N626	X17.1468	Y133.6693
N628	X17.2249	Y133.7010
N630	X17.3032	Y133.7328

N632	X17.3819	Y133.7647
N634	X17.4608	Y133.7967
N636	X17.5400	Y133.8287
N638	X17.6194	Y133.8609
N640	X17.6992	Y133.8931
N642	X17.7792	Y133.9254
N644	X17.8596	Y133.9578
N646	X17.9402	Y133.9903
N648	X18.0212	Y134.0229
N650	X18.1024	Y134.0555
N652	X18.1839	Y134.0883
N654	X18.2658	Y134.1211
N656	X18.3479	Y134.1540
N658	X18.4304	Y134.1869
N660	X18.5134	Y134.2199
N662	X18.5968	Y134.2528
N664	X18.6807	Y134.2857
N666	X18.7652	Y134.3185
N668	X18.8503	Y134.3511
N670	X18.9361	Y134.3836
N672	X19.0226	Y134.4159
N674	X19.1099	Y134.4480
N676	X19.1980	Y134.4798
N678	X19.2869	Y134.5113
N680	X19.3768	Y134.5425
N682	X19.4676	Y134.5733
N684	X19.5595	Y134.6037
N686	X19.6524	Y134.6336
N688	X19.7464	Y134.6631

N690	X19.8417	Y134.6921
N692	X19.9381	Y134.7206
N694	X20.0358	Y134.7485
N696	X20.1348	Y134.7758
N698	X20.2352	Y134.8025
N700	X20.3370	Y134.8285
N702	X20.4403	Y134.8537
N704	X20.5451	Y134.8783
N706	X20.6515	Y134.9020
N708	X20.7595	Y134.9250
N710	X20.8692	Y134.9471
N712	X20.9806	Y134.9684
N714	X21.0937	Y134.9887
N716	X21.2087	Y135.0081
N718	X21.3256	Y135.0265
N720	X21.4444	Y135.0439
N722	X21.5651	Y135.0603
N724	X21.6879	Y135.0755
N726	X21.8127	Y135.0897
N728	X21.9397	Y135.1027
N730	X22.0688	Y135.1145
N732	X22.2002	Y135.1252
N734	X22.3338	Y135.1345
N736	X22.4698	Y135.1426
N738	X22.6081	Y135.1494
N740	X22.7488	Y135.1548
N742	X22.8921	Y135.1588
N744	X23.0378	Y135.1614
N746	X23.1861	Y135.1626

N748	X23.3371	Y135.1622
N750	X23.4907	Y135.1604
N752	X23.6471	Y135.1570
N754	X23.8062	Y135.1520
N756	X23.9681	Y135.1453
N758	X24.1329	Y135.1371
N760	X24.3007	Y135.1271
N762	X24.4714	Y135.1154
N764	X24.6452	Y135.1019
N766	X24.8220	Y135.0867
N768	X25.0019	Y135.0696
N770	X25.1850	Y135.0506
N772	X25.3714	Y135.0298
N774	X25.5610	Y135.0070
N776	X25.7540	Y134.9823
N778	X25.9503	Y134.9555
N780	X26.1500	Y134.9268
N782	X26.3533	Y134.8959
N784	X26.5600	Y134.8630
N786	X26.7704	Y134.8279
N788	X26.9843	Y134.7907
N790	X27.2018	Y134.7513
N792	X27.4228	Y134.7098
N794	X27.6470	Y134.6663
N796	X27.8743	Y134.6208
N798	X28.1046	Y134.5734
N800	X28.3378	Y134.5240
N802	X28.5736	Y134.4729
N804	X28.8119	Y134.4200

N806	X29.0527	Y134.3654
N808	X29.2957	Y134.3091
N810	X29.5408	Y134.2512
N812	X29.7878	Y134.1918
N814	X30.0366	Y134.1308
N816	X30.2871	Y134.0685
N818	X30.5391	Y134.0047
N820	X30.7924	Y133.9396
N822	X31.0470	Y133.8733
N824	X31.3026	Y133.8057
N826	X31.5591	Y133.7370
N828	X31.8164	Y133.6671
N830	X32.0743	Y133.5963
N832	X32.3326	Y133.5244
N834	X32.5913	Y133.4515
N836	X32.8502	Y133.3778
N838	X33.1091	Y133.3033
N840	X33.3679	Y133.2279
N842	X33.6264	Y133.1519
N844	X33.8845	Y133.0752
N846	X34.1421	Y132.9978
N848	X34.3989	Y132.9199
N850	X34.6549	Y132.8415
N852	X34.9099	Y132.7627
N854	X35.1637	Y132.6835
N856	X35.4162	Y132.6039
N858	X35.6673	Y132.5240
N860	X35.9168	Y132.4439
N862	X36.1645	Y132.3637

N864	X36.4104	Y132.2833
N866	X36.6542	Y132.2029
N868	X36.8959	Y132.1224
N870	X37.1352	Y132.0420
N872	X37.3720	Y131.9617
N874	X37.6062	Y131.8816
N876	X37.8376	Y131.8016
N878	X38.0662	Y131.7220
N880	X38.2916	Y131.6426
N882	X38.5138	Y131.5637
N884	X38.7327	Y131.4851
N886	X38.9481	Y131.4071
N888	X39.1598	Y131.3296
N890	X39.3677	Y131.2527
N892	X39.5716	Y131.1764
N894	X39.7715	Y131.1009
N896	X39.9671	Y131.0261
N898	X40.1583	Y130.9521
N900	X40.3450	Y130.8790
N902	X40.5269	Y130.8068
N904	X40.7041	Y130.7357
N906	X40.8762	Y130.6655
N908	X41.0433	Y130.5964
N910	X41.2050	Y130.5285
N912	X41.3613	Y130.4618
N914	X41.5121	Y130.3964
N916	X41.6571	Y130.3322
N918	X41.7962	Y130.2694
N920	X41.9294	Y130.2081

N922	X42.0564	Y130.1482
N924	X42.1773	Y130.0897
N926	X42.2924	Y130.0327
N928	X42.4018	Y129.9769
N930	X42.5056	Y129.9225
N932	X42.6040	Y129.8692
N934	X42.6972	Y129.8172
N936	X42.7853	Y129.7663
N938	X42.8686	Y129.7164
N940	X42.9470	Y129.6676
N942	X43.0209	Y129.6197
N944	X43.0904	Y129.5727
N946	X43.1556	Y129.5266
N948	X43.2168	Y129.4814
N950	X43.2740	Y129.4368
N952	X43.3274	Y129.3930
N954	X43.3773	Y129.3498
N956	X43.4236	Y129.3072
N958	X43.4668	Y129.2652
N960	X43.5067	Y129.2236
N962	X43.5438	Y129.1825
N964	X43.5780	Y129.1418
N966	X43.6097	Y129.1014
N968	X43.6388	Y129.0613
N970	X43.6657	Y129.0214
N972	X43.6904	Y128.9817
N974	X43.7131	Y128.9422
N976	X43.7340	Y128.9026
N978	X43.7533	Y128.8632

N980 X43.7710 Y128.8236

N982 X43.7875 Y128.7840

N984 X43.8028 Y128.7443

N986 X43.8170 Y128.7044

N988 X43.8305 Y128.6642

N990 Y128.6237

N992 Y128.5829

N994 X43.8673 Y128.5417

N996 X43.8790 Y128.5000

N998 X43.8907 Y128.4578

N1000 X43.9025 Y128.4151

N1002 X43.9146 Y128.3717

N1004 X43.9271 Y128.3277

N1006 X43.9403 Y128.2830

N1008 X43.9543 Y128.2375

N1010 X43.9692 Y128.1911

N1012 X43.9852 Y128.1440

N1014 X44.0025 Y128.0958

N1016 X44.0212 Y128.0467

N1018 X44.0415 Y127.9966

N1020 X44.0636 Y127.9454

N1022 X44.0876 Y127.8930

N1024 X44.1137 Y127.8395

N1026 X44.1420 Y127.7847

N1028 X44.1727 Y127.7286

N1030 Y127.6711

N1032 X44.2421 Y127.6123

N1034 X44.2811 Y127.5520

N1036 X44.3231 Y127.4902

N1038	X44.3683	Y127.4269
N1040	X44.4169	Y127.3619
N1042	X44.4691	Y127.2953
N1044	X44.5250	Y127.2270
N1046	X44.5847	Y127.1569
N1048	X44.6485	Y127.0849
N1050	X44.7165	Y127.0111
N1052	X44.7888	Y126.9354
N1054	X44.8657	Y126.8577
N1056	X44.9471	Y126.7780
N1058	X45.0331	Y126.6964
N1060	X45.1234	Y126.6128
N1062	X45.2180	Y126.5274
N1064	X45.3167	Y126.4403
N1066	X45.4193	Y126.3515
N1068	X45.5258	Y126.2610
N1070	X45.6359	Y126.1689
N1072	X45.7496	Y126.0753
N1074	X45.8668	Y125.9802
N1076	X45.9873	Y125.8837
N1078	X46.1109	Y125.7859
N1080	X46.2375	Y125.6868
N1082	X46.3671	Y125.5865
N1084	X46.4994	Y125.4850
N1086	X46.6343	Y125.3824
N1088	X46.7718	Y125.2788
N1090	X46.9116	Y125.1742
N1092	X47.0536	Y125.0687
N1094	X47.1978	Y124.9623

N1096 X47.3439 Y124.8551

N1098 X47.4918 Y124.7472

N1100 X47.6414 Y124.6386

N1102 X47.7926 Y124.5293

N1104 X47.9452 Y124.4196

N1106 X48.0991 Y124.3093

N1108 X48.2542 Y124.1986

N1110 X48.4103 Y124.0875

N1112 X48.5673 Y123.9762

N1114 X48.7250 Y123.8645

N1116 X48.8834 Y123.7527

N1118 X49.0423 Y123.6407

N1120 X49.2015 Y123.5287

N1122 X49.3610 Y123.4166

N1124 X49.5206 Y123.3046

N1126 X49.6801 Y123.1928

N1128 X49.8395 Y123.0811

N1130 X49.9985 Y122.9696

N1132 X50.1571 Y122.8584

N1134 X50.3152 Y122.7476

N1136 X50.4725 Y122.6371

N1138 X50.6290 Y122.5272

N1140 X50.7845 Y122.4178

N1142 X50.9389 Y122.3090

N1144 X51.0921 Y122.2008

N1146 X51.2439 Y122.0934

N1148 X51.3942 Y121.9867

N1150 X51.5428 Y121.8809

N1152 X51.6897 Y121.7760

N1154	X51.8346	Y121.6720
N1156	X51.9776	Y121.5691
N1158	X52.1183	Y121.4672
N1160	X52.2568	Y121.3665
N1162	X52.3928	Y121.2670
N1164	X52.5262	Y121.1687
N1166	X52.6569	Y121.0718
N1168	X52.7848	Y120.9763
N1170	X52.9097	Y120.8822
N1172	X53.0315	Y120.7896
N1174	X53.1500	Y120.6985
N1176	X53.2652	Y120.6091
N1178	X53.3768	Y120.5214
N1180	X53.4849	Y120.4355
N1182	X53.5891	Y120.3513
N1184	X53.6894	Y120.2690
N1186	X53.7857	Y120.1886
N1188	X53.8778	Y120.1103
N1190	X53.9658	Y120.0339
N1192	X54.0497	Y119.9594
N1194	X54.1296	Y119.8869
N1196	X54.2058	Y119.8161
N1198	X54.2782	Y119.7471
N1200	X54.3472	Y119.6798
N1202	X54.4127	Y119.6141
N1204	X54.4749	Y119.5500
N1206	X54.5339	Y119.4874
N1208	X54.5899	Y119.4263
N1210	X54.6430	Y119.3666

N1212	X54.6932	Y119.3082
N1214	X54.7409	Y119.2511
N1216	X54.7860	Y119.1952
N1218	X54.8286	Y119.1405
N1220	X54.8690	Y119.0869
N1222	X54.9073	Y119.0343
N1224	X54.9435	Y118.9827
N1226	X54.9778	Y118.9320
N1228	X55.0103	Y118.8822
N1230	X55.0412	Y118.8332
N1232	X55.0706	Y118.7850
N1234	X55.0985	Y118.7374
N1236	X55.1252	Y118.6905
N1238	X55.1508	Y118.6441
N1240	X55.1753	Y118.5982
N1242	X55.1990	Y118.5528
N1244	X55.2218	Y118.5078
N1246	X55.2441	Y118.4631
N1248	X55.2658	Y118.4186
N1250	X55.2872	Y118.3744
N1252	X55.3083	Y118.3303
N1254	X55.3293	Y118.2864
N1256	X55.3502	Y118.2424
N1258	X55.3713	Y118.1985
N1260	X55.3927	Y118.1544
N1262	X55.4144	Y118.1102
N1264	X55.4367	Y118.0658
N1266	X55.4595	Y118.0211
N1268	X55.4831	Y117.9761

N1270	X55.5077	Y117.9308
N1272	X55.5332	Y117.8849
N1274	X55.5598	Y117.8386
N1276	X55.5878	Y117.7918
N1278	X55.6171	Y117.7443
N1280	X55.6479	Y117.6961
N1282	X55.6804	Y117.6472
N1284	X55.7146	Y117.5974
N1286	X55.7508	Y117.5469
N1288	X55.7890	Y117.4954
N1290	X55.8293	Y117.4429
N1292	X55.8719	Y117.3894
N1294	X55.9169	Y117.3348
N1296	X55.9644	Y117.2791
N1298	X56.0147	Y117.2221
N1300	X56.0676	Y117.1639
N1302	X56.1235	Y117.1043
N1304	X56.1825	Y117.0433
N1306	X56.2446	Y116.9809
N1308	X56.3100	Y116.9170
N1310	X56.3788	Y116.8515
N1312	X56.4512	Y116.7844
N1314	X56.5272	Y116.7156
N1316	X56.6070	Y116.6450
N1318	X56.6908	Y116.5726
N1320	X56.7786	Y116.4984
N1322	X56.8706	Y116.4223
N1324	X56.9666	Y116.3442
N1326	X57.0667	Y116.2643

N1328	X57.1705	Y116.1827
N1330	X57.2781	Y116.0993
N1332	X57.3892	Y116.0143
N1334	X57.5038	Y115.9276
N1336	X57.6216	Y115.8394
N1338	X57.7426	Y115.7497
N1340	X57.8667	Y115.6586
N1342	X57.9936	Y115.5660
N1344	X58.1233	Y115.4722
N1346	X58.2557	Y115.3771
N1348	X58.3905	Y115.2808
N1350	X58.5277	Y115.1833
N1352	X58.6671	Y115.0847
N1354	X58.8086	Y114.9851
N1356	X58.9521	Y114.8845
N1358	X59.0974	Y114.7830
N1360	X59.2444	Y114.6806
N1362	X59.3929	Y114.5774
N1364	X59.5429	Y114.4735
N1366	X59.6942	Y114.3688
N1368	X59.8466	Y114.2635
N1370	X60.0001	Y114.1576
N1372	X60.1545	Y114.0512
N1374	X60.3096	Y113.9444
N1376	X60.4653	Y113.8371
N1378	X60.6216	Y113.7294
N1380	X60.7782	Y113.6215
N1382	X60.9350	Y113.5133
N1384	X61.0919	Y113.4049

N1386	X61.2488	Y113.2964
N1388	X61.4055	Y113.1878
N1390	X61.5619	Y113.0792
N1392	X61.7179	Y112.9707
N1394	X61.8732	Y112.8622
N1396	X62.0279	Y112.7539
N1398	X62.1817	Y112.6458
N1400	X62.3346	Y112.5380
N1402	X62.4863	Y112.4305
N1404	X62.6368	Y112.3234
N1406	X62.7860	Y112.2167
N1408	X62.9336	Y112.1106
N1410	X63.0795	Y112.0049
N1412	X63.2237	Y111.8999
N1414	X63.3660	Y111.7956
N1416	X63.5063	Y111.6920
N1418	X63.6443	Y111.5891
N1420	X63.7801	Y111.4871
N1422	X63.9134	Y111.3860
N1424	X64.0441	Y111.2859
N1426	X64.1721	Y111.1867
N1428	X64.2973	Y111.0886
N1430	X64.4195	Y110.9917
N1432	X64.5386	Y110.8959
N1434	X64.6544	Y110.8013
N1436	X64.7669	Y110.7081
N1438	X64.8758	Y110.6162
N1440	X64.9811	Y110.5257
N1442	X65.0826	Y110.4366

N1444 X65.1802 Y110.3491

N1446 X65.2738 Y110.2632

N1448 X65.3632 Y110.1789

N1450 X65.4482 Y110.0962

N1452 X65.5288 Y110.0154

N1454 X65.6049 Y109.9363

N1456 X65.6763 Y109.8591

N1458 X65.7431 Y109.7837

N1460 X65.8055 Y109.7101

N1462 X65.8637 Y109.6382

N1464 X65.9177 Y109.5680

N1466 X65.9677 Y109.4995

N1468 X66.0138 Y109.4326

N1470 X66.0562 Y109.3673

N1472 X66.0950 Y109.3036

N1474 X66.1304 Y109.2415

N1476 X66.1624 Y109.1808

N1478 X66.1912

N1480 X66.2170 Y109.0637

N1482 X66.2399 Y109.0073

N1484 X66.2600 Y108.9522

N1486 X66.2774 Y108.8985

N1488 X66.2923 Y108.8460

N1490 X66.3049 Y108.7947

N1492 X66.3152 Y108.7447

N1494 X66.3235 Y108.6958

N1496 X66.3297 Y108.6481

N1498 X66.3342 Y108.6014

N1500 X66.3369 Y108.5559

N1502	X66.3381	Y108.5113
N1504	X66.3379	Y108.4678
N1506	X66.3363	Y108.4252
N1508	X66.3337	Y108.3835
N1510	X66.3300	Y108.3427
N1512	X66.3254	Y108.3027
N1514	X66.3201	Y108.2635
N1516	X66.3142	Y108.2252
N1518	X66.3079	Y108.1875
N1520	X66.3012	Y108.1506
N1522	X66.2943	Y108.1143
N1524	X66.2873	Y108.0787
N1526	X66.2805	Y108.0437
N1528	X66.2738	Y108.0092
N1530	X66.2675	Y107.9752
N1532	X66.2616	Y107.9418
N1534	X66.2564	Y107.9088
N1536	X66.2520	Y107.8762
N1538	X66.2484	Y107.8439
N1540	X66.2459	Y107.8121
N1542	X66.2445	Y107.7805
N1544	X66.2444	Y107.7492
N1546	X66.2458	Y107.7181
N1548	X66.2487	Y107.6873
N1550	X66.2534	Y107.6566
N1552	X66.2599	Y107.6260
N1554	X66.2683	Y107.5956
N1556	X66.2789	Y107.5652
N1558	X66.2918	Y107.5348

N1560 X66.3070 Y107.5044

N1562 X66.3247 Y107.4739

N1564 X66.3452 Y107.4434

N1566 X66.3684 Y107.4127

N1568 X66.3945 Y107.3819

N1570 X66.4237 Y107.3509

N1572 X66.4561 Y107.3196

N1574 X66.4919 Y107.2881

N1576 X66.5311 Y107.2563

N1578 X66.5739 Y107.2241

N1580 X66.6205 Y107.1916

N1582 X66.6710 Y107.1587

N1584 X66.7255 Y107.1253

N1586 X66.7842 Y107.0915

N1588 X66.8471 Y107.0571

N1590 X66.9143 Y107.0222

N1592 X66.9858 Y106.9868

N1594 X67.0613 Y106.9508

N1596 X67.1406 Y106.9143

N1598 X67.2238 Y106.8774

N1600 X67.3106 Y106.8399

N1602 X67.4010 Y106.8019

N1604 X67.4947 Y106.7635

N1606 X67.5916 Y106.7245

N1608 X67.6917 Y106.6851

N1610 X67.7947 Y106.6452

N1612 X67.9006 Y106.6049

N1614 X68.0092 Y106.5641

N1616 X68.1204 Y106.5229

N1618	X68.2340	Y106.4812
N1620	X68.3500	Y106.4391
N1622	X68.4681	Y106.3966
N1624	X68.5883	Y106.3536
N1626	X68.7103	Y106.3103
N1628	X68.8342	Y106.2665
N1630	X68.9597	Y106.2224
N1632	X69.0867	Y106.1778
N1634	X69.2150	Y106.1329
N1636	X69.3446	Y106.0876
N1638	X69.4754	Y106.0419
N1640	X69.6071	Y105.9958
N1642	X69.7396	Y105.9494
N1644	X69.8729	Y105.9027
N1646	X70.0067	Y105.8556
N1648	X70.1410	Y105.8082
N1650	X70.2755	Y105.7604
N1652	X70.4103	Y105.7123
N1654	X70.5451	Y105.6639
N1656	X70.6797	Y105.6152
N1658	X70.8142	Y105.5662
N1660	X70.9483	Y105.5169
N1662	X71.0819	Y105.4673
N1664	X71.2149	Y105.4175
N1666	X71.3471	Y105.3673
N1668	X71.4784	Y105.3169
N1670	X71.6087	Y105.2662
N1672	X71.7379	Y105.2153
N1674	X71.8657	Y105.1641

N1676	X71.9921	Y105.1127
N1678	X72.1170	Y105.0611
N1680	X72.2401	Y105.0092
N1682	X72.3614	Y104.9571
N1684	X72.4808	Y104.9048
N1686	X72.5981	Y104.8523
N1688	X72.7131	Y104.7996
N1690	X72.8257	Y104.7467
N1692	X72.9359	Y104.6936
N1694	X73.0434	Y104.6403
N1696	X73.1482	Y104.5869
N1698	X73.2501	Y104.5333
N1700	X73.3489	Y104.4795
N1702	X73.4446	Y104.4256
N1704	X73.5370	Y104.3715
N1706	X73.6259	Y104.3173
N1708	X73.7113	Y104.2630
N1710	X73.7929	Y104.2085
N1712	X73.8708	Y104.1540
N1714	X73.9446	Y104.0993
N1716	X74.0144	Y104.0445
N1718	X74.0799	Y103.9896
N1720	X74.1411	Y103.9347
N1722	X74.1978	Y103.8796
N1724	X74.2501	Y103.8245
N1726	X74.2983	Y103.7694
N1728	X74.3425	Y103.7142
N1730	X74.3831	Y103.6591
N1732	X74.4202	Y103.6040

N1734	X74.4540	Y103.5490
N1736	X74.4849	Y103.4941
N1738	X74.5129	Y103.4393
N1740	X74.5384	Y103.3846
N1742	X74.5616	Y103.3301
N1744	X74.5826	Y103.2759
N1746	X74.6018	Y103.2218
N1748	X74.6193	Y103.1681
N1750	X74.6354	Y103.1146
N1752	X74.6503	Y103.0614
N1754	X74.6642	Y103.0086
N1756	X74.6773	Y102.9561
N1758	X74.6899	Y102.9040
N1760	X74.7023	Y102.8523
N1762	X74.7145	Y102.8011
N1764	X74.7269	Y102.7503
N1766	X74.7397	Y102.7001
N1768	X74.7531	Y102.6503
N1770	X74.7673	Y102.6011
N1772	X74.7826	Y102.5525
N1774	X74.7992	Y102.5045
N1776	X74.8173	Y102.4571
N1778	X74.8371	Y102.4104
N1780	X74.8589	Y102.3643
N1782	X74.8829	Y102.3190
N1784	X74.9093	Y102.2744
N1786	X74.9383	Y102.2306
N1788	X74.9703	Y102.1875
N1790	X75.0053	Y102.1453

N1792	X75.0436	Y102.1039
N1794	X75.0855	Y102.0634
N1796	X75.1312	Y102.0237
N1798	X75.1808	Y101.9850
N1800	X75.2347	Y101.9473
N1802	X75.2930	Y101.9105
N1804	X75.3561	Y101.8747
N1806	X75.4240	Y101.8400
N1808	X75.4971	Y101.8063
N1810	X75.5755	Y101.7736
N1812	X75.6595	Y101.7421
N1814	X75.7493	Y101.7118
N1816	X75.8452	Y101.6825
N1818	X75.9473	Y101.6545
N1820	X76.0559	Y101.6277
N1822	X76.1713	Y101.6021
N1824	X76.2935	Y101.5778
N1826	X76.4230	Y101.5548
N1828	X76.5598	Y101.5331
N1830	X76.7043	Y101.5127
N1832	X76.8566	Y101.4938
N1834	X77.0170	Y101.4762
N1836	X77.1857	Y101.4600
N1838	X77.3629	Y101.4454
N1840	X77.5489	Y101.4321
N1842	X77.7438	Y101.4204
N1844	X77.9480	Y101.4102
N1846	X78.1616	Y101.4016
N1848	X78.3848	Y101.3946

N1850	X78.6180	Y101.3892
N1852	X78.8612	Y101.3854
N1854	X79.1148	Y101.3833
N1856	X79.3789	Y101.3828
N1858	X79.6532	Y101.3840
N1860	X79.9376	Y101.3868
N1862	X80.2317	Y101.3912
N1864	X80.5354	Y101.3970
N1866	X80.8484	Y101.4043
N1868	X81.1703	Y101.4130
N1870	X81.5010	Y101.4230
N1872	X81.8402	Y101.4343
N1874	X82.1876	Y101.4469
N1876	X82.5430	Y101.4607
N1878	X82.9062	Y101.4757
N1880	X83.2768	Y101.4917
N1882	X83.6546	Y101.5089
N1884	X84.0394	Y101.5270
N1886	X84.4309	Y101.5461
N1888	X84.8289	Y101.5661
N1890	X85.2330	Y101.5870
N1892	X85.6431	Y101.6087
N1894	X86.0588	Y101.6312
N1896	X86.4800	Y101.6544
N1898	X86.9064	Y101.6783
N1900	X87.3376	Y101.7028
N1902	X87.7736	Y101.7279
N1904	X88.2139	Y101.7535
N1906	X88.6584	Y101.7796

N1908	X89.1067	Y101.8061
N1910	X89.5587	Y101.8330
N1912	X90.0141	Y101.8603
N1914	X90.4726	Y101.8879
N1916	X90.9339	Y101.9156
N1918	X91.3979	Y101.9436
N1920	X91.8642	Y101.9717
N1922	X92.3326	Y102.0000
N1924	X92.8029	Y102.0282
N1926	X93.2747	Y102.0565
N1928	X93.7479	Y102.0847
N1930	X94.2222	Y102.1129
N1932	X94.6973	Y102.1408
N1934	X95.1729	Y102.1686
N1936	X95.6488	Y102.1962
N1938	X96.1248	Y102.2234
N1940	X96.6006	Y102.2504
N1942	X97.0759	Y102.2769
N1944	X97.5505	Y102.3030
N1946	X98.0242	Y102.3286
N1948	X98.4966	Y102.3537
N1950	X98.9675	Y102.3782
N1952	X99.4367	Y102.4021
N1954	X99.9039	Y102.4253
N1956	X100.3688	Y102.4478
N1958	X100.8313	Y102.4695
N1960	X101.2909	Y102.4904
N1962	X101.7476	Y102.5104
N1964	X102.2009	Y102.5295

N1966 X102.6508 Y102.5476

N1968 X103.0968 Y102.5648

N1970 X103.5388 Y102.5808

N1972 X103.9765 Y102.5958

N1974 X104.4097 Y102.6096

N1976 X104.8380 Y102.6222

N1978 X105.2613 Y102.6335

N1980 X105.6792 Y102.6436

N1982 X106.0916 Y102.6522

N1984 X106.4981 Y102.6595

N1986 X106.8986 Y102.6654

N1988 X107.2927 Y102.6697

N1990 X107.6803 Y102.6725

N1992 X108.0615 Y102.6739

N1994 X108.4365 Y102.6738

N1996 X108.8052 Y102.6723

N1998 X109.1677 Y102.6694

N2000 X109.5243 Y102.6652

N2002 X109.8748 Y102.6596

N2004 X110.2195 Y102.6528

N2006 X110.5584 Y102.6447

N2008 X110.8915 Y102.6355

N2010 X111.2191 Y102.6250

N2012 X111.5411 Y102.6134

N2014 X111.8576 Y102.6008

N2016 X112.1688 Y102.5870

N2018 X112.4747 Y102.5722

N2020 X112.7755 Y102.5564

N2022 X113.0711 Y102.5396

N2024	X113.3617	Y102.5219
N2026	X113.6473	Y102.5033
N2028	X113.9281	Y102.4838
N2030	X114.2042	Y102.4635
N2032	X114.4756	Y102.4424
N2034	X114.7423	Y102.4205
N2036	X115.0046	Y102.3979
N2038	X115.2624	Y102.3745
N2040	X115.5160	Y102.3506
N2042	X115.7652	Y102.3260
N2044	X116.0103	Y102.3008
N2046	X116.2514	Y102.2750
N2048	X116.4884	Y102.2487
N2050	X116.7215	Y102.2219
N2052	X116.9508	Y102.1947
N2054	X117.1764	Y102.1670
N2056	X117.3984	Y102.1389
N2058	X117.6167	Y102.1105
N2060	X117.8316	Y102.0817
N2062	X118.0431	Y102.0527
N2064	X118.2513	Y102.0234
N2066	X118.4563	Y101.9939
N2068	X118.6582	Y101.9642
N2070	X118.8570	Y101.9343
N2072	X119.0528	Y101.9044
N2074	X119.2458	Y101.8743
N2076	X119.4360	Y101.8442
N2078	X119.6234	Y101.8140
N2080	X119.8083	Y101.7839

N2082 X119.9906 Y101.7539

N2084 X120.1705 Y101.7239

N2086 X120.3481 Y101.6940

N2088 X120.5233 Y101.6643

N2090 X120.6964 Y101.6348

N2092 X120.8674 Y101.6055

N2094 X121.0363 Y101.5764

N2096 X121.2033 Y101.5477

N2098 X121.3685 Y101.5192

N2100 X121.5319 Y101.4911

N2102 X121.6937 Y101.4634

N2104 X121.8538 Y101.4362

N2106 X122.0125 Y101.4094

N2108 X122.1697 Y101.3830

N2110 X122.3257 Y101.3573

N2112 X122.4803 Y101.3320

N2114 X122.6338 Y101.3074

N2116 X122.7863 Y101.2834

N2118 X122.9378 Y101.2600

N2120 X123.0883 Y101.2374

N2122 X123.2381 Y101.2155

N2124 X123.3870 Y101.1943

N2126 X123.5353 Y101.1738

N2128 X123.6828 Y101.1540

N2130 X123.8296 Y101.1349

N2132 X123.9757 Y101.1164

N2134 X124.1211 Y101.0985

N2136 X124.2659 Y101.0812

N2138 X124.4101 Y101.0645

N2140	X124.5537	Y101.0483
N2142	X124.6967	Y101.0327
N2144	X124.8392	Y101.0176
N2146	X124.9811	Y101.0030
N2148	X125.1225	Y100.9889
N2150	X125.2635	Y100.9752
N2152	X125.4039	Y100.9619
N2154	X125.5439	Y100.9490
N2156	X125.6835	Y100.9365
N2158	X125.8227	Y100.9244
N2160	X125.9615	Y100.9126
N2162	X126.1000	Y100.9011
N2164	X126.2381	Y100.8899
N2166	X126.3759	Y100.8790
N2168	X126.5134	Y100.8684
N2170	X126.6506	Y100.8580
N2172	X126.7876	Y100.8477
N2174	X126.9244	Y100.8377
N2176	X127.0609	Y100.8279
N2178	X127.1973	Y100.8181
N2180	X127.3335	Y100.8085
N2182	X127.4695	Y100.7990
N2184	X127.6055	Y100.7896
N2186	X127.7413	Y100.7803
N2188	X127.8771	Y100.7710
N2190	X128.0128	Y100.7617
N2192	X128.1484	Y100.7523
N2194	X128.2841	Y100.7430
N2196	X128.4197	Y100.7336

N2198 X128.5554 Y100.7241

N2200 X128.6912 Y100.7146

N2202 X128.8270 Y100.7049

N2204 X128.9629 Y100.6951

N2206 X129.0989 Y100.6851

N2208 X129.2351 Y100.6750

N2210 X129.3714 Y100.6646

N2212 X129.5079 Y100.6541

N2214 X129.6446 Y100.6432

N2216 X129.7816 Y100.6321

N2218 X129.9188 Y100.6208

N2220 X130.0562 Y100.6091

N2222 X130.1940 Y100.5971

N2224 X130.3320 Y100.5847

N2226 X130.4704 Y100.5720

N2228 X130.6092 Y100.5588

N2230 X130.7483 Y100.5453

N2232 X130.8878 Y100.5313

N2234 X131.0278 Y100.5168

N2236 X131.1682 Y100.5019

N2238 X131.3090 Y100.4864

N2240 X131.4503 Y100.4705

N2242 X131.5922 Y100.4539

N2244 X131.7345 Y100.4369

N2246 X131.8775 Y100.4192

N2248 X132.0210 Y100.4009

N2250 X132.1650 Y100.3820

N2252 X132.3097 Y100.3624

N2254 X132.4551 Y100.3421

N2256 X132.6011 Y100.3212

N2258 X132.7477 Y100.2994

N2260 X132.8951 Y100.2769

N2262 X133.0430 Y100.2535

N2264 X133.1917 Y100.2291

N2266 X133.3411 Y100.2037

N2268 X133.4911 Y100.1772

N2270 X133.6419 Y100.1496

N2272 X133.7933 Y100.1208

N2274 X133.9455 Y100.0908

N2276 X134.0983 Y100.0594

N2278 X134.2519 Y100.0267

N2280 X134.4062 Y99.9924

N2282 X134.5612 Y99.9567

N2284 X134.7169 Y99.9194

N2286 X134.8734 Y99.8805

N2288 X135.0306 Y99.8399

N2290 X135.1885 Y99.7975

N2292 X135.3473 Y99.7533

N2294 X135.5067 Y99.7072

N2296 X135.6669 Y99.6592

N2298 X135.8279 Y99.6091

N2300 X135.9896 Y99.5570

N2302 X136.1522 Y99.5027

N2304 X136.3155 Y99.4463

N2306 X136.4795 Y99.3876

N2308 X136.6444 Y99.3265

N2310 X136.8101 Y99.2631

N2312 X136.9765 Y99.1972

N2314 X137.1438 Y99.1289

N2316 X137.3119 Y99.0579

N2318 X137.4808 Y98.9843

N2320 X137.6505 Y98.9080

N2322 Y98.8290

N2324 X137.9923 Y98.7471

N2326 X138.1645 Y98.6624

N2328 X138.3376 Y98.5747

N2330 X138.5114 Y98.4840

N2332 X138.6862 Y98.3902

N2334 X138.8617 Y98.2933

N2336 X139.0382 Y98.1932

N2338 X139.2155 Y98.0898

N2340 X139.3936 Y97.9831

N2342 X139.5727 Y97.8731

N2344 X139.7526 Y97.7595

N2346 X139.9334 Y97.6425

N2348 X140.1151 Y97.5219

N2350 X140.2976 Y97.3977

N2352 X140.4811 Y97.2697

N2354 X140.6655 Y97.1381

N2356 X140.8508 Y97.0025

N2358 X141.0370 Y96.8631

N2360 X141.2241 Y96.7198

N2362 X141.4122 Y96.5724

N2364 X141.6011 Y96.4210

N2366 X141.7910 Y96.2655

N2368 X141.9819 Y96.1057

N2370 X142.1737 Y95.9417

N2372 X142.3664 Y95.7734

N2374 X142.5601 Y95.6006

N2376 X142.7547 Y95.4235

N2378 X142.9503 Y95.2418

N2380 X143.1469 Y95.0555

N2382 X143.3445 Y94.8647

N2384 X143.5430 Y94.6691

N2386 X143.7425 Y94.4688

N2388 X143.9430 Y94.2636

N2390 X144.1445 Y94.0536

N2392 X144.3468 Y93.8389

N2394 X144.5501 Y93.6195

N2396 X144.7541 Y93.3955

N2398 X144.9589 Y93.1671

N2400 X145.1643 Y92.9343

N2402 X145.3703 Y92.6972

N2404 X145.5769 Y92.4559

N2406 X145.7840 Y92.2105

N2408 X145.9915 Y91.9611

N2410 X146.1994 Y91.7078

N2412 X146.4076 Y91.4507

N2414 X146.6160 Y91.1898

N2416 X146.8247 Y90.9252

N2418 X147.0334 Y90.6572

N2420 X147.2422 Y90.3856

N2422 X147.4511 Y90.1107

N2424 X147.6598 Y89.8325

N2426 X147.8685 Y89.5512

N2428 X148.0770 Y89.2667

N2430	X148.2852	Y88.9792
N2432	X148.4932	Y88.6889
N2434	X148.7007	Y88.3957
N2436	X148.9079	Y88.0998
N2438	X149.1146	Y87.8012
N2440	X149.3208	Y87.5001
N2442	X149.5263	Y87.1966
N2444	X149.7312	Y86.8907
N2446	X149.9354	Y86.5826
N2448	X150.1388	Y86.2722
N2450	X150.3413	Y85.9598
N2452	X150.5430	Y85.6454
N2454	X150.7436	Y85.3291
N2456	X150.9433	Y85.0110
N2458	X151.1419	Y84.6912
N2460	X151.3393	Y84.3698
N2462	X151.5356	Y84.0468
N2464	X151.7305	Y83.7224
N2466	X151.9242	Y83.3967
N2468	X152.1164	Y83.0697
N2470	X152.3072	Y82.7415
N2472	X152.4965	Y82.4123
N2474	X152.6842	Y82.0821
N2476	X152.8704	Y81.7510
N2478	X153.0548	Y81.4191
N2480	X153.2374	Y81.0865
N2482	X153.4183	Y80.7533
N2484	X153.5973	Y80.4196
N2486	X153.7743	Y80.0854

N2488	X153.9494	Y79.7510
N2490	X154.1224	Y79.4163
N2492	X154.2933	Y79.0814
N2494	X154.4620	Y78.7465
N2496	X154.6285	Y78.4116
N2498	X154.7927	Y78.0768
N2500	X154.9546	Y77.7422
N2502	X155.1140	Y77.4080
N2504	X155.2709	Y77.0742
N2506	X155.4253	Y76.7408
N2508	X155.5772	Y76.4081
N2510	X155.7263	Y76.0760
N2512	X155.8728	Y75.7447
N2514	X156.0164	Y75.4142
N2516	X156.1573	Y75.0847
N2518	X156.2952	Y74.7563
N2520	X156.4302	Y74.4290
N2522	X156.5621	Y74.1029
N2524	X156.6911	Y73.7781
N2526	X156.8170	Y73.4545
N2528	X156.9401	Y73.1323
N2530	X157.0603	Y72.8114
N2532	X157.1777	Y72.4918
N2534	X157.2924	Y72.1736
N2536	X157.4043	Y71.8568
N2538	X157.5136	Y71.5413
N2540	X157.6202	Y71.2272
N2542	X157.7244	Y70.9145
N2544	X157.8260	Y70.6033

N2546	X157.9252	Y70.2934
N2548	X158.0219	Y69.9851
N2550	X158.1164	Y69.6782
N2552	X158.2085	Y69.3727
N2554	X158.2984	Y69.0688
N2556	X158.3861	Y68.7664
N2558	X158.4716	Y68.4655
N2560	X158.5551	Y68.1661
N2562	X158.6365	Y67.8683
N2564	X158.7160	Y67.5721
N2566	X158.7935	Y67.2775
N2568	X158.8691	Y66.9844
N2570	X158.9429	Y66.6930
N2572	X159.0149	Y66.4032
N2574	X159.0851	Y66.1150
N2576	X159.1537	Y65.8285
N2578	X159.2207	Y65.5436
N2580	X159.2860	Y65.2605
N2582	X159.3499	Y64.9790
N2584	X159.4123	Y64.6993
N2586	X159.4732	Y64.4213
N2588	X159.5328	Y64.1450
N2590	X159.5911	Y63.8705
N2592	X159.6480	Y63.5978
N2594	X159.7038	Y63.3269
N2596	X159.7584	Y63.0577
N2598	X159.8119	Y62.7904
N2600	X159.8643	Y62.5249
N2602	X159.9157	Y62.2613

N2604	X159.9662	Y61.9995
N2606	X160.0157	Y61.7396
N2608	X160.0644	Y61.4816
N2610	X160.1122	Y61.2255
N2612	X160.1593	Y60.9714
N2614	X160.2057	Y60.7191
N2616	X160.2515	Y60.4688
N2618	X160.2966	Y60.2205
N2620	X160.3412	Y59.9742
N2622	X160.3853	Y59.7298
N2624	X160.4290	Y59.4875
N2626	X160.4722	Y59.2472
N2628	X160.5151	Y59.0089
N2630	X160.5577	Y58.7727
N2632	X160.6000	Y58.5385
N2634	X160.6422	Y58.3065
N2636	X160.6842	Y58.0765
N2638	X160.7262	Y57.8487
N2640	X160.7680	Y57.6229
N2642	X160.8099	Y57.3993
N2644	X160.8519	Y57.1779
N2646	X160.8940	Y56.9586
N2648	X160.9362	Y56.7416
N2650	X160.9787	Y56.5267
N2652	X161.0214	Y56.3141
N2654	X161.0645	Y56.1036
N2656	X161.1079	Y55.8954
N2658	X161.1516	Y55.6895
N2660	X161.1957	Y55.4858

N2662	X161.2400	Y55.2844
N2664	X161.2846	Y55.0851
N2666	X161.3293	Y54.8882
N2668	X161.3742	Y54.6934
N2670	X161.4192	Y54.5009
N2672	X161.4643	Y54.3107
N2674	X161.5094	Y54.1226
N2676	X161.5546	Y53.9368
N2678	X161.5996	Y53.7532
N2680	X161.6447	Y53.5719
N2682	X161.6896	Y53.3928
N2684	X161.7343	Y53.2159
N2686	X161.7789	Y53.0412
N2688	X161.8233	Y52.8687
N2690	X161.8674	Y52.6985
N2692	X161.9112	Y52.5305
N2694	X161.9546	Y52.3646
N2696	X161.9977	Y52.2010
N2698	X162.0404	Y52.0397
N2700	X162.0826	Y51.8805
N2702	X162.1243	Y51.7235
N2704	X162.1656	Y51.5688
N2706	X162.2062	Y51.4162
N2708	X162.2463	Y51.2659
N2710	X162.2857	Y51.1177
N2712	X162.3244	Y50.9718
N2714	X162.3625	Y50.8281
N2716	X162.3997	Y50.6865
N2718	X162.4362	Y50.5472

N2720	X162.4719	Y50.4100
N2722	X162.5067	Y50.2751
N2724	X162.5406	Y50.1423
N2726	X162.5736	Y50.0117
N2728	X162.6055	Y49.8833
N2730	X162.6365	Y49.7571
N2732	X162.6664	Y49.6331
N2734	X162.6953	Y49.5113
N2736	X162.7230	Y49.3916
N2738	X162.7495	Y49.2741
N2740	X162.7749	Y49.1588
N2742	X162.7990	Y49.0457
N2744	X162.8218	Y48.9348
N2746	X162.8433	Y48.8260
N2748	X162.8635	Y48.7194
N2750	X162.8823	Y48.6150
N2752	X162.8996	Y48.5127
N2754	X162.9155	Y48.4126
N2756	X162.9299	Y48.3147
N2758	X162.9427	Y48.2190
N2760	X162.9540	Y48.1254
N2762	X162.9636	Y48.0339
N2764	X162.9716	Y47.9446
N2766	X162.9779	Y47.8575
N2768	X162.9825	Y47.7725
N2770	X162.9853	Y47.6897
N2772	X162.9863	Y47.6091
N2774	X162.9854	Y47.5306
N2776	X162.9827	Y47.4542

N2778	X162.9781	Y47.3800
N2780	X162.9715	Y47.3079
N2782	X162.9629	Y47.2380
N2784	X162.9523	Y47.1702
N2786	X162.9396	Y47.1046
N2788	X162.9249	Y47.0411
N2790	X162.9080	Y46.9797
N2792	X162.8890	Y46.9205
N2794	X162.8679	Y46.8633
N2796	X162.8448	Y46.8082
N2798	X162.8197	Y46.7551
N2800	X162.7926	Y46.7041
N2802	X162.7636	Y46.6550
N2804	X162.7327	Y46.6079
N2806	X162.6999	Y46.5628
N2808	X162.6652	Y46.5196
N2810	X162.6287	Y46.4783
N2812	X162.5905	Y46.4388
N2814	X162.5504	Y46.4012
N2816	X162.5087	Y46.3655
N2818	X162.4652	Y46.3315
N2820	X162.4201	Y46.2994
N2822	X162.3733	Y46.2690
N2824	X162.3250	Y46.2403
N2826	X162.2750	Y46.2134
N2828	X162.2236	Y46.1881
N2830	X162.1706	Y46.1645
N2832	X162.1161	Y46.1426
N2834	X162.0602	Y46.1222

N2836	X162.0028	Y46.1035
N2838	X161.9441	Y46.0864
N2840	X161.8840	Y46.0708
N2842	X161.8226	Y46.0567
N2844	X161.7599	Y46.0441
N2846	X161.6959	Y46.0330
N2848	X161.6306	Y46.0234
N2850	X161.5642	Y46.0152
N2852	X161.4966	Y46.0085
N2854	X161.4279	Y46.0031
N2856	X161.3580	Y45.9990
N2858	X161.2871	Y45.9964
N2860	X161.2151	Y45.9950
N2862	X161.1420	Y45.9949
N2864	X161.0680	Y45.9961
N2866	X160.9931	Y45.9986
N2868	X160.9172	Y46.0023
N2870	X160.8404	Y46.0072
N2872	X160.7627	Y46.0132
N2874	X160.6842	Y46.0204
N2876	X160.6049	Y46.0288
N2878	X160.5248	Y46.0382
N2880	X160.4440	Y46.0488
N2882	X160.3625	Y46.0604
N2884	X160.2802	Y46.0730
N2886	X160.1974	Y46.0867
N2888	X160.1139	Y46.1014
N2890	X160.0298	Y46.1170
N2892	X159.9451	Y46.1336

N2894	X159.8599	Y46.1511
N2896	X159.7743	Y46.1695
N2898	X159.6881	Y46.1888
N2900	X159.6015	Y46.2089
N2902	X159.5145	Y46.2298
N2904	X159.4272	Y46.2516
N2906	X159.3395	Y46.2742
N2908	X159.2514	Y46.2975
N2910	X159.1631	Y46.3215
N2912	X159.0746	Y46.3463
N2914	X158.9858	Y46.3717
N2916	X158.8968	Y46.3979
N2918	X158.8076	Y46.4246
N2920	X158.7184	Y46.4520
N2922	X158.6290	Y46.4800
N2924	X158.5395	Y46.5086
N2926	X158.4500	Y46.5378
N2928	X158.3603	Y46.5676
N2930	X158.2706	Y46.5980
N2932	X158.1807	Y46.6291
N2934	X158.0907	Y46.6609
N2936	X158.0005	Y46.6933
N2938	X157.9102	Y46.7264
N2940	X157.8198	Y46.7603
N2942	X157.7292	Y46.7948
N2944	X157.6385	Y46.8301
N2946	X157.5476	Y46.8661
N2948	X157.4565	Y46.9029
N2950	X157.3653	Y46.9405

N2952	X157.2739	Y46.9788
N2954	X157.1822	Y47.0180
N2956	X157.0904	Y47.0579
N2958	X156.9984	Y47.0987
N2960	X156.9061	Y47.1403
N2962	X156.8137	Y47.1828
N2964	X156.7210	Y47.2262
N2966	X156.6281	Y47.2704
N2968	X156.5349	Y47.3156
N2970	X156.4415	Y47.3616
N2972	X156.3478	Y47.4086
N2974	X156.2539	Y47.4565
N2976	X156.1597	Y47.5054
N2978	X156.0652	Y47.5552
N2980	X155.9705	Y47.6061
N2982	X155.8755	Y47.6579
N2984	X155.7801	Y47.7107
N2986	X155.6845	Y47.7645
N2988	X155.5885	Y47.8194
N2990	X155.4923	Y47.8753
N2992	X155.3957	Y47.9323
N2994	X155.2988	Y47.9904
N2996	X155.2015	Y48.0495
N2998	X155.1039	Y48.1098
N3000	X155.0060	Y48.1712
N3002	X154.9077	Y48.2337
N3004	X154.8090	Y48.2973
N3006	X154.7100	Y48.3621
N3008	X154.6106	Y48.4281

N3010	X154.5108	Y48.4953
N3012	X154.4106	Y48.5637
N3014	X154.3100	Y48.6333
N3016	X154.2090	Y48.7041
N3018	X154.1076	Y48.7762
N3020	X154.0057	Y48.8495
N3022	X153.9035	Y48.9241
N3024	X153.8008	Y48.9999
N3026	X153.6977	Y49.0771
N3028	X153.5941	Y49.1556
N3030	X153.4901	Y49.2354
N3032	X153.3856	Y49.3166
N3034	X153.2806	Y49.3991
N3036	X153.1752	Y49.4829
N3038	X153.0693	Y49.5682
N3040	X152.9629	Y49.6548
N3042	X152.8560	Y49.7428
N3044	X152.7486	Y49.8323
N3046	X152.6407	Y49.9232
N3048	X152.5323	Y50.0156
N3050	X152.4233	Y50.1094
N3052	X152.3139	Y50.2047
N3054	X152.2038	Y50.3015
N3056	X152.0933	Y50.3998
N3058	X151.9822	Y50.4996
N3060	X151.8707	Y50.6010
N3062	X151.7586	Y50.7041
N3064	X151.6461	Y50.8089
N3066	X151.5332	Y50.9154

N3068	X151.4198	Y51.0238
N3070	X151.3061	Y51.1339
N3072	X151.1921	Y51.2460
N3074	X151.0777	Y51.3600
N3076	X150.9629	Y51.4760
N3078	X150.8479	Y51.5941
N3080	X150.7327	Y51.7142
N3082	X150.6172	Y51.8365
N3084	X150.5015	Y51.9610
N3086	X150.3856	Y52.0877
N3088	X150.2695	Y52.2168
N3090	X150.1533	Y52.3481
N3092	X150.0370	Y52.4819
N3094	X149.9205	Y52.6181
N3096	X149.8040	Y52.7568
N3098	X149.6875	Y52.8980
N3100	X149.5709	Y53.0419
N3102	X149.4544	Y53.1883
N3104	X149.3379	Y53.3375
N3106	X149.2214	Y53.4894
N3108	X149.1050	Y53.6441
N3110	X148.9886	Y53.8016
N3112	X148.8724	Y53.9620
N3114	X148.7564	Y54.1254
N3116	X148.6405	Y54.2917
N3118	X148.5248	Y54.4611
N3120	X148.4093	Y54.6336
N3122	X148.2941	Y54.8092
N3124	X148.1791	Y54.9880

N3126	X148.0644	Y55.1700
N3128	X147.9501	Y55.3554
N3130	X147.8360	Y55.5440
N3132	X147.7224	Y55.7361
N3134	X147.6091	Y55.9316
N3136	X147.4962	Y56.1305
N3138	X147.3837	Y56.3330
N3140	X147.2717	Y56.5391
N3142	X147.1602	Y56.7488
N3144	X147.0492	Y56.9622
N3146	X146.9387	Y57.1794
N3148	X146.8287	Y57.4003
N3150	X146.7194	Y57.6250
N3152	X146.6106	Y57.8537
N3154	X146.5024	Y58.0863
N3156	X146.3949	Y58.3228
N3158	X146.2881	Y58.5634
N3160	X146.1820	Y58.8080
N3162	X146.0765	Y59.0568
N3164	X145.9718	Y59.3098
N3166	X145.8679	Y59.5670
N3168	X145.7648	Y59.8285
N3170	X145.6625	Y60.0943
N3172	X145.5610	Y60.3645
N3174	X145.4604	Y60.6391
N3176	X145.3606	Y60.9182
N3178	X145.2618	Y61.2019
N3180	X145.1639	Y61.4901
N3182	X145.0670	Y61.7829

N3184	X144.9710	Y62.0804
N3186	X144.8761	Y62.3827
N3188	X144.7821	Y62.6897
N3190	X144.6893	Y63.0015
N3192	X144.5974	Y63.3179
N3194	X144.5065	Y63.6388
N3196	X144.4166	Y63.9640
N3198	X144.3276	Y64.2934
N3200	X144.2396	Y64.6267
N3202	X144.1524	Y64.9639
N3204	X144.0661	Y65.3047
N3206	X143.9807	Y65.6491
N3208	X143.8961	Y65.9967
N3210	X143.8122	Y66.3476
N3212	X143.7292	Y66.7015
N3214	X143.6469	Y67.0583
N3216	X143.5653	Y67.4178
N3218	X143.4843	Y67.7798
N3220	X143.4041	Y68.1442
N3222	X143.3245	Y68.5108
N3224	X143.2455	Y68.8795
N3226	X143.1671	Y69.2500
N3228	X143.0893	Y69.6223
N3230	X143.0120	Y69.9962
N3232	X142.9352	Y70.3715
N3234	X142.8589	Y70.7481
N3236	X142.7831	Y71.1257
N3238	X142.7077	Y71.5043
N3240	X142.6327	Y71.8837

N3242	X142.5582	Y72.2637
N3244	X142.4839	Y72.6441
N3246	X142.4101	Y73.0248
N3248	X142.3365	Y73.4056
N3250	X142.2632	Y73.7864
N3252	X142.1902	Y74.1671
N3254	X142.1175	Y74.5473
N3256	X142.0449	Y74.9271
N3258	X141.9726	Y75.3062
N3260	X141.9004	Y75.6844
N3262	X141.8283	Y76.0616
N3264	X141.7564	Y76.4377
N3266	X141.6845	Y76.8125
N3268	X141.6127	Y77.1858
N3270	X141.5410	Y77.5574
N3272	X141.4692	Y77.9273
N3274	X141.3975	Y78.2952
N3276	X141.3257	Y78.6610
N3278	X141.2538	Y79.0244
N3280	X141.1819	Y79.3855
N3282	X141.1098	Y79.7439
N3284	X141.0376	Y80.0996
N3286	X140.9652	Y80.4523
N3288	X140.8927	Y80.8020
N3290	X140.8199	Y81.1484
N3292	X140.7469	Y81.4914
N3294	X140.6737	Y81.8309
N3296	X140.6001	Y82.1666
N3298	X140.5262	Y82.4984

N3300	X140.4520	Y82.8261
N3302	X140.3774	Y83.1497
N3304	X140.3025	Y83.4689
N3306	X140.2271	Y83.7835
N3308	X140.1513	Y84.0934
N3310	X140.0750	Y84.3985
N3312	X139.9982	Y84.6986
N3314	X139.9209	Y84.9935
N3316	X139.8431	Y85.2831
N3318	X139.7647	Y85.5671
N3320	X139.6857	Y85.8455
N3322	X139.6061	Y86.1181
N3324	X139.5259	Y86.3848
N3326	X139.4451	Y86.6457
N3328	X139.3638	Y86.9008
N3330	X139.2820	Y87.1502
N3332	X139.1998	Y87.3940
N3334	X139.1171	Y87.6321
N3336	X139.0341	Y87.8647
N3338	X138.9508	Y88.0919
N3340	X138.8671	Y88.3136
N3342	X138.7833	Y88.5300
N3344	X138.6992	Y88.7410
N3346	X138.6150	Y88.9468
N3348	X138.5306	Y89.1474
N3350	X138.4462	Y89.3429
N3352	X138.3617	Y89.5333
N3354	X138.2773	Y89.7186
N3356	X138.1928	Y89.8990

N3358	X138.1085	Y90.0745
N3360	X138.0243	Y90.2452
N3362	X137.9402	Y90.4110
N3364	X137.8564	Y90.5721
N3366	X137.7728	Y90.7285
N3368	X137.6895	Y90.8804
N3370	X137.6065	Y91.0276
N3372	X137.5239	Y91.1703
N3374	X137.4417	Y91.3086
N3376	X137.3600	Y91.4425
N3378	X137.2788	Y91.5721
N3380	X137.1980	Y91.6973
N3382	X137.1179	Y91.8184
N3384	X137.0384	Y91.9353
N3386	X136.9595	Y92.0481
N3388	X136.8813	Y92.1568
N3390	X136.8039	Y92.2615
N3392	X136.7272	Y92.3623
N3394	X136.6514	Y92.4593
N3396	X136.5764	Y92.5524
N3398	X136.5024	Y92.6417
N3400	X136.4292	Y92.7273
N3402	X136.3571	Y92.8093
N3404	X136.2860	Y92.8877
N3406	X136.2159	Y92.9626
N3408	X136.1470	Y93.0339
N3410	X136.0791	Y93.1019
N3412	X136.0125	Y93.1665
N3414	X135.9471	Y93.2278

N3416 X135.8830 Y93.2858

N3418 X135.8202 Y93.3406

N3420 X135.7587 Y93.3923

N3422 X135.6987 Y93.4410

N3424 X135.6400 Y93.4866

N3426 X135.5829 Y93.5292

N3428 X135.5272 Y93.5690

N3430 X135.4731 Y93.6058

N3432 X135.4206 Y93.6399

N3434 X135.3698 Y93.6713

N3436 X135.3206 Y93.7000

N3438 X135.2731 Y93.7260

N3440 X135.2274 Y93.7495

N3442 X135.1835 Y93.7705

N3444 X135.1415 Y93.7891

N3446 X135.1013 Y93.8052

N3448 X135.0630 Y93.8190

N3450 X135.0267 Y93.8306

N3452 X134.9925 Y93.8399

N3454 X134.9602 Y93.8470

N3456 X134.9301 Y93.8521

N3458 X134.9020 Y93.8550

N3460 X134.8759 Y93.8558

N3462 X134.8518 Y93.8546

N3464 X134.8297 Y93.8512

N3466 X134.8096 Y93.8456

N3468 X134.7913 Y93.8379

N3470 X134.7748 Y93.8281

N3472 X134.7602 Y93.8161

N3474	X134.7474	Y93.8020
N3476	X134.7363	Y93.7856
N3478	X134.7269	Y93.7671
N3480	X134.7192	Y93.7464
N3482	X134.7131	Y93.7236
N3484	X134.7086	Y93.6985
N3486	X134.7057	Y93.6711
N3488	X134.7044	Y93.6416
N3490	X134.7045	Y93.6098
N3492	X134.7061	Y93.5758
N3494	X134.7091	Y93.5396
N3496	X134.7134	Y93.5011
N3498	X134.7192	Y93.4603
N3500	X134.7263	Y93.4172
N3502	X134.7346	Y93.3719
N3504	X134.7442	Y93.3243
N3506	X134.7550	Y93.2744
N3508	X134.7669	Y93.2222
N3510	X134.7800	Y93.1677
N3512	X134.7942	Y93.1108
N3514	X134.8095	Y93.0516
N3516	X134.8258	Y92.9901
N3518	X134.8431	Y92.9263
N3520	X134.8613	Y92.8600
N3522	X134.8805	Y92.7915
N3524	X134.9006	Y92.7205
N3526	X134.9215	Y92.6472
N3528	X134.9432	Y92.5715
N3530	X134.9657	Y92.4934

N3532	X134.9889	Y92.4129
N3534	X135.0129	Y92.3300
N3536	X135.0375	Y92.2446
N3538	X135.0627	Y92.1568
N3540	X135.0886	Y92.0666
N3542	X135.1150	Y91.9740
N3544	X135.1420	Y91.8789
N3546	X135.1694	Y91.7813
N3548	X135.1973	Y91.6813
N3550	X135.2256	Y91.5788
N3552	X135.2543	Y91.4738
N3554	X135.2834	Y91.3664
N3556	X135.3127	Y91.2564
N3558	X135.3424	Y91.1439
N3560	X135.3723	Y91.0289
N3562	X135.4023	Y90.9113
N3564	X135.4326	Y90.7913
N3566	X135.4630	Y90.6687
N3568	X135.4935	Y90.5435
N3570	X135.5240	Y90.4158
N3572	X135.5546	Y90.2855
N3574	X135.5852	Y90.1526
N3576	X135.6157	Y90.0172
N3578	X135.6462	Y89.8792
N3580	X135.6765	Y89.7385
N3582	X135.7067	Y89.5953
N3584	X135.7367	Y89.4494
N3586	X135.7664	Y89.3009
N3588	X135.7959	Y89.1498

N3590	X135.8252	Y88.9961
N3592	X135.8541	Y88.8398
N3594	X135.8828	Y88.6809
N3596	X135.9111	Y88.5195
N3598	X135.9393	Y88.3557
N3600	X135.9672	Y88.1896
N3602	X135.9948	Y88.0210
N3604	X136.0222	Y87.8502
N3606	X136.0494	Y87.6771
N3608	X136.0764	Y87.5019
N3610	X136.1031	Y87.3245
N3612	X136.1297	Y87.1450
N3614	X136.1560	Y86.9634
N3616	X136.1822	Y86.7799
N3618	X136.2082	Y86.5944
N3620	X136.2340	Y86.4070
N3622	X136.2597	Y86.2178
N3624	X136.2852	Y86.0267
N3626	X136.3105	Y85.8339
N3628	X136.3357	Y85.6394
N3630	X136.3608	Y85.4432
N3632	X136.3858	Y85.2455
N3634	X136.4107	Y85.0461
N3636	X136.4354	Y84.8453
N3638	X136.4601	Y84.6430
N3640	X136.4847	Y84.4393
N3642	X136.5091	Y84.2342
N3644	X136.5336	Y84.0279
N3646	X136.5579	Y83.8202

N3648	X136.5822	Y83.6114
N3650	X136.6065	Y83.4013
N3652	X136.6307	Y83.1902
N3654	X136.6549	Y82.9780
N3656	X136.6790	Y82.7648
N3658	X136.7032	Y82.5506
N3660	X136.7273	Y82.3354
N3662	X136.7514	Y82.1194
N3664	X136.7756	Y81.9026
N3666	X136.7997	Y81.6850
N3668	X136.8239	Y81.4667
N3670	X136.8481	Y81.2478
N3672	X136.8724	Y81.0281
N3674	X136.8967	Y80.8079
N3676	X136.9211	Y80.5872
N3678	X136.9455	Y80.3660
N3680	X136.9700	Y80.1444
N3682	X136.9946	Y79.9224
N3684	X137.0193	Y79.7000
N3686	X137.0441	Y79.4774
N3688	X137.0690	Y79.2545
N3690	X137.0940	Y79.0315
N3692	X137.1191	Y78.8083
N3694	X137.1443	Y78.5850
N3696	X137.1697	Y78.3617
N3698	X137.1953	Y78.1384
N3700	X137.2210	Y77.9152
N3702	X137.2468	Y77.6920
N3704	X137.2729	Y77.4690

N3706	X137.2991	Y77.2463
N3708	X137.3255	Y77.0238
N3710	X137.3521	Y76.8015
N3712	X137.3789	Y76.5797
N3714	X137.4059	Y76.3582
N3716	X137.4331	Y76.1372
N3718	X137.4606	Y75.9167
N3720	X137.4883	Y75.6967
N3722	X137.5162	Y75.4774
N3724	X137.5444	Y75.2586
N3726	X137.5729	Y75.0405
N3728	X137.6016	Y74.8229
N3730	X137.6307	Y74.6060
N3732	X137.6600	Y74.3896
N3734	X137.6897	Y74.1737
N3736	X137.7198	Y73.9584
N3738	X137.7502	Y73.7436
N3740	X137.7810	Y73.5292
N3742	X137.8122	Y73.3154
N3744	X137.8438	Y73.1020
N3746	X137.8758	Y72.8891
N3748	X137.9082	Y72.6767
N3750	X137.9411	Y72.4646
N3752	X137.9745	Y72.2530
N3754	X138.0083	Y72.0418
N3756	X138.0426	Y71.8309
N3758	X138.0775	Y71.6204
N3760	X138.1129	Y71.4103
N3762	X138.1488	Y71.2005

N3764	X138.1853	Y70.9910
N3766	X138.2223	Y70.7818
N3768	X138.2600	Y70.5729
N3770	X138.2982	Y70.3643
N3772	X138.3371	Y70.1560
N3774	X138.3766	Y69.9479
N3776	X138.4167	Y69.7400
N3778	X138.4575	Y69.5323
N3780	X138.4990	Y69.3249
N3782	X138.5412	Y69.1176
N3784	X138.5841	Y68.9105
N3786	X138.6277	Y68.7035
N3788	X138.6721	Y68.4967
N3790	X138.7172	Y68.2900
N3792	X138.7630	Y68.0834
N3794	X138.8097	Y67.8769
N3796	X138.8572	Y67.6705
N3798	X138.9054	Y67.4641
N3800	X138.9545	Y67.2578
N3802	X139.0045	Y67.0516
N3804	X139.0553	Y66.8453
N3806	X139.1070	Y66.6391
N3808	X139.1595	Y66.4328
N3810	X139.2130	Y66.2265
N3812	X139.2674	Y66.0202
N3814	X139.3228	Y65.8138
N3816	X139.3790	Y65.6074
N3818	X139.4363	Y65.4008
N3820	X139.4945	Y65.1942

N3822	X139.5537	Y64.9874
N3824	X139.6140	Y64.7805
N3826	X139.6752	Y64.5735
N3828	X139.7376	Y64.3663
N3830	X139.8009	Y64.1589
N3832	X139.8653	Y63.9513
N3834	X139.9309	Y63.7435
N3836	X139.9975	Y63.5355
N3838	X140.0652	Y63.3273
N3840	X140.1341	Y63.1188
N3842	X140.2041	Y62.9100
N3844	X140.2753	Y62.7010
N3846	X140.3476	Y62.4916
N3848	X140.4212	Y62.2820
N3850	X140.4959	Y62.0720
N3852	X140.5719	Y61.8617
N3854	X140.6491	Y61.6510
N3856	X140.7276	Y61.4399
N3858	X140.8073	Y61.2285
N3860	X140.8881	Y61.0168
N3862	X140.9701	Y60.8048
N3864	X141.0532	Y60.5926
N3866	X141.1373	Y60.3802
N3868	X141.2224	Y60.1676
N3870	X141.3084	Y59.9549
N3872	X141.3953	Y59.7422
N3874	X141.4831	Y59.5294
N3876	X141.5717	Y59.3166
N3878	X141.6610	Y59.1039

N3880	X141.7510	Y58.8913
N3882	X141.8417	Y58.6788
N3884	X141.9330	Y58.4664
N3886	X142.0249	Y58.2543
N3888	X142.1173	Y58.0424
N3890	X142.2101	Y57.8307
N3892	X142.3034	Y57.6194
N3894	X142.3970	Y57.4085
N3896	X142.4910	Y57.1980
N3898	X142.5853	Y56.9879
N3900	X142.6798	Y56.7783
N3902	X142.7744	Y56.5692
N3904	X142.8692	Y56.3606
N3906	X142.9642	Y56.1527
N3908	X143.0591	Y55.9454
N3910	X143.1541	Y55.7388
N3912	X143.2490	Y55.5329
N3914	X143.3438	Y55.3278
N3916	X143.4384	Y55.1234
N3918	X143.5329	Y54.9199
N3920	X143.6271	Y54.7173
N3922	X143.7211	Y54.5156
N3924	X143.8147	Y54.3148
N3926	X143.9079	Y54.1150
N3928	X144.0007	Y53.9163
N3930	X144.0930	Y53.7187
N3932	X144.1849	Y53.5221
N3934	X144.2761	Y53.3267
N3936	X144.3667	Y53.1325

N3938 X144.4567 Y52.9396

N3940 X144.5460 Y52.7479

N3942 X144.6345 Y52.5575

N3944 X144.7222 Y52.3685

N3946 X144.8090 Y52.1809

N3948 X144.8950 Y51.9946

N3950 X144.9800 Y51.8099

N3952 X145.0640 Y51.6267

N3954 X145.1470 Y51.4450

N3956 X145.2289 Y51.2649

N3958 X145.3096 Y51.0864

N3960 X145.3892 Y50.9097

N3962 X145.4676 Y50.7346

N3964 X145.5446 Y50.5612

N3966 X145.6204 Y50.3897

N3968 X145.6948 Y50.2199

N3970 X145.7677 Y50.0521

N3972 X145.8392 Y49.8861

N3974 X145.9092 Y49.7221

N3976 X145.9777 Y49.5601

N3978 X146.0445 Y49.4001

N3980 X146.1097 Y49.2421

N3982 X146.1731 Y49.0863

N3984 X146.2349 Y48.9326

N3986 X146.2948 Y48.7810

N3988 X146.3529 Y48.6317

N3990 X146.4092 Y48.4847

N3992 X146.4636 Y48.3398

N3994 X146.5162 Y48.1970

N3996	X146.5671	Y48.0561
N3998	X146.6163	Y47.9172
N4000	X146.6639	Y47.7801
N4002	X146.7099	Y47.6447
N4004	X146.7544	Y47.5109
N4006	X146.7975	Y47.3787
N4008	X146.8391	Y47.2479
N4010	X146.8794	Y47.1185
N4012	X146.9184	Y46.9904
N4014	X146.9561	Y46.8634
N4016	X146.9926	Y46.7375
N4018	X147.0280	Y46.6127
N4020	X147.0623	Y46.4887
N4022	X147.0956	Y46.3656
N4024	X147.1278	Y46.2432
N4026	X147.1591	Y46.1214
N4028	X147.1896	Y46.0002
N4030	X147.2192	Y45.8795
N4032	X147.2481	Y45.7591
N4034	X147.2762	Y45.6390
N4036	X147.3036	Y45.5191
N4038	X147.3305	Y45.3993
N4040	X147.3567	Y45.2796
N4042	X147.3825	Y45.1597
N4044	X147.4078	Y45.0397
N4046	X147.4327	Y44.9195
N4048	X147.4572	Y44.7989
N4050	X147.4814	Y44.6778
N4052	X147.5054	Y44.5563

N4054	X147.5292	Y44.4341
N4056	X147.5529	Y44.3113
N4058	X147.5765	Y44.1876
N4060	X147.6000	Y44.0630
N4062	X147.6235	Y43.9375
N4064	X147.6471	Y43.8110
N4066	X147.6709	Y43.6832
N4068	X147.6948	Y43.5543
N4070	X147.7189	Y43.4240
N4072	X147.7434	Y43.2923
N4074	X147.7681	Y43.1590
N4076	X147.7933	Y43.0242
N4078	X147.8188	Y42.8877
N4080	X147.8449	Y42.7494
N4082	X147.8715	Y42.6093
N4084	X147.8987	Y42.4672
N4086	X147.9266	Y42.3230
N4088	X147.9552	Y42.1767
N4090	X147.9845	Y42.0282
N4092	X148.0146	Y41.8774
N4094	X148.0456	Y41.7241
N4096	X148.0776	Y41.5684
N4098	X148.1104	Y41.4100
N4100	X148.1443	Y41.2490
N4102	X148.1793	Y41.0853
N4104	X148.2154	Y40.9187
N4106	X148.2527	Y40.7491
N4108	X148.2912	Y40.5765
N4110	X148.3310	Y40.4008

N4112	X148.3721	Y40.2218
N4114	X148.4147	Y40.0396
N4116	X148.4586	Y39.8540
N4118	X148.5041	Y39.6649
N4120	X148.5511	Y39.4722
N4122	X148.5997	Y39.2759
N4124	X148.6499	Y39.0758
N4126	X148.7017	Y38.8722
N4128	X148.7551	Y38.6652
N4130	X148.8099	Y38.4548
N4132	X148.8661	Y38.2411
N4134	X148.9237	Y38.0243
N4136	X148.9826	Y37.8045
N4138	X149.0428	Y37.5818
N4140	X149.1042	Y37.3563
N4142	X149.1668	Y37.1281
N4144	X149.2305	Y36.8973
N4146	X149.2954	Y36.6641
N4148	X149.3612	Y36.4284
N4150	X149.4280	Y36.1906
N4152	X149.4958	Y35.9506
N4154	X149.5644	Y35.7085
N4156	X149.6339	Y35.4646
N4158	X149.7042	Y35.2188
N4160	X149.7752	Y34.9714
N4162	X149.8469	Y34.7223
N4164	X149.9192	Y34.4718
N4166	X149.9921	Y34.2199
N4168	X150.0656	Y33.9668

N4170	X150.1396	Y33.7125
N4172	X150.2139	Y33.4572
N4174	X150.2887	Y33.2009
N4176	X150.3639	Y32.9439
N4178	X150.4393	Y32.6861
N4180	X150.5150	Y32.4278
N4182	X150.5908	Y32.1689
N4184	X150.6669	Y31.9097
N4186	X150.7430	Y31.6503
N4188	X150.8191	Y31.3907
N4190	X150.8953	Y31.1310
N4192	X150.9714	Y30.8714
N4194	X151.0474	Y30.6120
N4196	X151.1233	Y30.3529
N4198	X151.1990	Y30.0942
N4200	X151.2744	Y29.8360
N4202	X151.3496	Y29.5784
N4204	X151.4244	Y29.3215
N4206	X151.4988	Y29.0655
N4208	X151.5727	Y28.8105
N4210	X151.6462	Y28.5565
N4212	X151.7191	Y28.3036
N4214	X151.7915	Y28.0521
N4216	X151.8632	Y27.8020
N4218	X151.9342	Y27.5533
N4220	X152.0045	Y27.3063
N4222	X152.0740	Y27.0610
N4224	X152.1427	Y26.8176
N4226	X152.2104	Y26.5761

N4228	X152.2773	Y26.3366
N4230	X152.3432	Y26.0993
N4232	X152.4080	Y25.8643
N4234	X152.4718	Y25.6317
N4236	X152.5344	Y25.4016
N4238	X152.5958	Y25.1741
N4240	X152.6561	Y24.9493
N4242	X152.7150	Y24.7274
N4244	X152.7726	Y24.5083
N4246	X152.8289	Y24.2924
N4248	X152.8837	Y24.0796
N4250	X152.9371	Y23.8700
N4252	X152.9889	Y23.6639
N4254	X153.0392	Y23.4612
N4256	X153.0878	Y23.2622
N4258	X153.1348	Y23.0667
N4260	X153.1803	Y22.8748
N4262	X153.2241	Y22.6864
N4264	X153.2663	Y22.5014
N4266	X153.3070	Y22.3198
N4268	X153.3462	Y22.1415
N4270	X153.3838	Y21.9665
N4272	X153.4199	Y21.7946
N4274	X153.4545	Y21.6259
N4276	X153.4877	Y21.4603
N4278	X153.5194	Y21.2977
N4280	X153.5497	Y21.1381
N4282	X153.5785	Y20.9814
N4284	X153.6059	Y20.8276

N4286 X153.6320 Y20.6765

N4288 X153.6567 Y20.5282

N4290 X153.6800 Y20.3826

N4292 X153.7020 Y20.2396

N4294 X153.7227 Y20.0992

N4296 X153.7420 Y19.9612

N4298 X153.7601 Y19.8257

N4300 X153.7769 Y19.6926

N4302 X153.7925 Y19.5619

N4304 X153.8068 Y19.4334

N4306 X153.8200 Y19.3071

N4308 X153.8319 Y19.1830

N4310 X153.8426 Y19.0610

N4312 X153.8522 Y18.9411

N4314 X153.8606 Y18.8231

N4316 X153.8679 Y18.7071

N4318 X153.8741 Y18.5929

N4320 X153.8792 Y18.4805

N4322 X153.8832 Y18.3699

N4324 X153.8861 Y18.2610

N4326 X153.8880 Y18.1538

N4328 X153.8889 Y18.0481

N4330 X153.8887 Y17.9440

N4332 X153.8876 Y17.8413

N4334 X153.8855 Y17.7400

N4336 X153.8824 Y17.6401

N4338 X153.8783 Y17.5414

N4340 X153.8734 Y17.4441

N4342 X153.8675 Y17.3478

N4344	X153.8608	Y17.2527
N4346	X153.8532	Y17.1587
N4348	X153.8447	Y17.0657
N4350	X153.8353	Y16.9736
N4352	X153.8252	Y16.8824
N4354	X153.8142	Y16.7920
N4356	X153.8025	Y16.7025
N4358	X153.7900	Y16.6136
N4360	X153.7767	Y16.5254
N4362	X153.7627	Y16.4378
N4364	X153.7479	Y16.3507
N4366	X153.7325	Y16.2641
N4368	X153.7164	Y16.1780
N4370	X153.6996	Y16.0922
N4372	X153.6821	Y16.0067
N4374	X153.6640	Y15.9215
N4376	X153.6453	Y15.8364
N4378	X153.6260	Y15.7515
N4380	X153.6061	Y15.6667
N4382	X153.5856	Y15.5819
N4384	X153.5646	Y15.4971
N4386	X153.5431	Y15.4122
N4388	X153.5210	Y15.3271
N4390	X153.4984	Y15.2419
N4392	X153.4753	Y15.1564
N4394	X153.4517	Y15.0708
N4396	X153.4275	Y14.9850
N4398	X153.4028	Y14.8991
N4400	X153.3775	Y14.8131

N4402 X153.3515 Y14.7270

N4404 X153.3250 Y14.6409

N4406 X153.2978 Y14.5547

N4408 X153.2699 Y14.4685

N4410 X153.2414 Y14.3823

N4412 X153.2122 Y14.2961

N4414 X153.1823 Y14.2099

N4416 X153.1516 Y14.1238

N4418 X153.1202 Y14.0378

N4420 X153.0880 Y13.9520

N4422 X153.0551 Y13.8662

N4424 X153.0213 Y13.7806

N4426 X152.9868 Y13.6952

N4428 X152.9514 Y13.6099

N4430 X152.9151 Y13.5249

N4432 X152.8780 Y13.4401

N4434 X152.8400 Y13.3556

N4436 X152.8011 Y13.2714

N4438 X152.7612 Y13.1874

N4440 X152.7205 Y13.1038

N4442 X152.6787 Y13.0205

N4444 X152.6360 Y12.9376

N4446 X152.5923 Y12.8550

N4448 X152.5476 Y12.7729

N4450 X152.5019 Y12.6912

N4452 X152.4551 Y12.6100

N4454 X152.4072 Y12.5292

N4456 X152.3583 Y12.4489

N4458 X152.3083 Y12.3692

N4460	X152.2571	Y12.2899
N4462	X152.2049	Y12.2113
N4464	X152.1514	Y12.1332
N4466	X152.0968	Y12.0557
N4468	X152.0410	Y11.9788
N4470	X151.9840	Y11.9026
N4472	X151.9258	Y11.8270
N4474	X151.8664	Y11.7521
N4476	X151.8056	Y11.6780
N4478	X151.7437	Y11.6045
N4480	X151.6804	Y11.5318
N4482	X151.6158	Y11.4599
N4484	X151.5498	Y11.3888
N4486	X151.4826	Y11.3185
N4488	X151.4139	Y11.2490
N4490	X151.3439	Y11.1803
N4492	X151.2725	Y11.1126
N4494	X151.1997	Y11.0458
N4496	X151.1254	Y10.9798
N4498	X151.0497	Y10.9148
N4500	X150.9725	Y10.8508
N4502	X150.8938	Y10.7878
N4504	X150.8136	Y10.7257
N4506	X150.7319	Y10.6647
N4508	X150.6487	Y10.6048
N4510	X150.5639	Y10.5459
N4512	X150.4775	Y10.4881
N4514	X150.3895	Y10.4314
N4516	X150.2999	Y10.3759

N4518	X150.2087	Y10.3215
N4520	X150.1158	Y10.2683
N4522	X150.0213	Y10.2163
N4524	X149.9251	Y10.1655
N4526	X149.8274	Y10.1159
N4528	X149.7281	Y10.0675
N4530	X149.6273	Y10.0204
N4532	X149.5252	Y9.9745
N4534	X149.4218	Y9.9299
N4536	X149.3171	Y9.8865
N4538	X149.2113	Y9.8444
N4540	X149.1043	Y9.8036
N4542	X148.9963	Y9.7640
N4544	X148.8874	Y9.7257
N4546	X148.7775	Y9.6887
N4548	X148.6669	Y9.6530
N4550	X148.5555	Y9.6186
N4552	X148.4433	Y9.5855
N4554	X148.3306	Y9.5537
N4556	X148.2173	Y9.5232
N4558	X148.1036	Y9.4940
N4560	X147.9894	Y9.4662
N4562	X147.8749	Y9.4397
N4564	X147.7602	Y9.4145
N4566	X147.6452	Y9.3907
N4568	X147.5301	Y9.3682
N4570	X147.4150	Y9.3471
N4572	X147.2998	Y9.3274
N4574	X147.1847	Y9.3091

N4576	X147.0698	Y9.2921
N4578	X146.9551	Y9.2765
N4580	X146.8407	Y9.2623
N4582	X146.7266	Y9.2495
N4584	X146.6130	Y9.2381
N4586	X146.4999	Y9.2281
N4588	X146.3873	Y9.2196
N4590	X146.2754	Y9.2124
N4592	X146.1641	Y9.2067
N4594	X146.0537	Y9.2024
N4596	X145.9440	Y9.1996
N4598	X145.8353	Y9.1982
N4600	X145.7276	Y9.1983
N4602	X145.6209	Y9.1998
N4604	X145.5154	Y9.2029
N4606	X145.4110	Y9.2073
N4608	X145.3079	Y9.2133
N4610	X145.2061	Y9.2207
N4612	X145.1058	Y9.2297
N4614	X145.0068	Y9.2401
N4616	X144.9095	Y9.2521
N4618	X144.8137	Y9.2655
N4620	X144.7196	Y9.2805
N4622	X144.6273	Y9.2970
N4624	X144.5368	Y9.3150
N4626	X144.4481	Y9.3346
N4628	X144.3614	Y9.3557
N4630	X144.2768	Y9.3784
N4632	X144.1942	Y9.4026

N4634	X144.1138	Y9.4284
N4636	X144.0356	Y9.4557
N4638	X143.9598	Y9.4847
N4640	X143.8863	Y9.5152
N4642	X143.8152	Y9.5473
N4644	X143.7467	Y9.5810
N4646	X143.6807	Y9.6162
N4648	X143.6174	Y9.6531
N4650	X143.5568	Y9.6917
N4652	X143.4990	Y9.7318
N4654	X143.4440	Y9.7735
N4656	X143.3920	Y9.8169
N4658	X143.3429	Y9.8619
N4660	X143.2966	Y9.9085
N4662	X143.2532	Y9.9566
N4664	X143.2126	Y10.0062
N4666	X143.1747	Y10.0572
N4668	X143.1394	Y10.1096
N4670	X143.1067	Y10.1634
N4672	X143.0766	Y10.2186
N4674	X143.0490	Y10.2750
N4676	Y10.3326	
N4678	X143.0010	Y10.3915
N4680	X142.9806	Y10.4515
N4682	X142.9625	Y10.5127
N4684	X142.9466	Y10.5749
N4686	X142.9328	Y10.6382
N4688	X142.9212	Y10.7024
N4690	X142.9117	Y10.7677

N4692	X142.9042	Y10.8338
N4694	X142.8986	Y10.9008
N4696	X142.8950	Y10.9687
N4698	X142.8933	Y11.0373
N4700	X142.8933	Y11.1067
N4702	X142.8951	Y11.1768
N4704	X142.8986	Y11.2476
N4706	X142.9038	Y11.3190
N4708	X142.9105	Y11.3910
N4710	X142.9188	Y11.4636
N4712	X142.9286	Y11.5366
N4714	X142.9398	Y11.6101
N4716	X142.9524	Y11.6841
N4718	X142.9663	Y11.7584
N4720	X142.9815	Y11.8331
N4722	X142.9979	Y11.9081
N4724	X143.0155	Y11.9834
N4726	X143.0342	Y12.0589
N4728	X143.0539	Y12.1345
N4730	X143.0747	Y12.2103
N4732	X143.0965	Y12.2862
N4734	X143.1191	Y12.3622
N4736	X143.1426	Y12.4382
N4738	X143.1669	Y12.5141
N4740	X143.1919	Y12.5900
N4742	X143.2176	Y12.6658
N4744	X143.2440	Y12.7415
N4746	X143.2709	Y12.8169
N4748	X143.2984	Y12.8922

N4750	X143.3263	Y12.9671
N4752	X143.3547	Y13.0418
N4754	X143.3834	Y13.1161
N4756	X143.4125	Y13.1900
N4758	X143.4418	Y13.2635
N4760	X143.4713	Y13.3365
N4762	X143.5010	Y13.4091
N4764	X143.5308	Y13.4810
N4766	X143.5606	Y13.5524
N4768	X143.5905	Y13.6231
N4770	X143.6202	Y13.6931
N4772	X143.6499	Y13.7625
N4774	X143.6794	Y13.8311
N4776	X143.7087	Y13.8988
N4778	X143.7378	Y13.9658
N4780	X143.7665	Y14.0318
N4782	X143.7948	Y14.0970
N4784	X143.8227	Y14.1612
N4786	X143.8501	Y14.2243
N4788	X143.8770	Y14.2865
N4790	X143.9034	Y14.3475
N4792	X143.9291	Y14.4075
N4794	X143.9543	Y14.4665
N4796	X143.9789	Y14.5244
N4798	X144.0029	Y14.5813
N4800	X144.0265	Y14.6372
N4802	X144.0496	Y14.6921
N4804	X144.0721	Y14.7461
N4806	X144.0942	Y14.7991

N4808	X144.1158	Y14.8512
N4810	X144.1370	Y14.9024
N4812	X144.1578	Y14.9528
N4814	X144.1781	Y15.0023
N4816	X144.1980	Y15.0509
N4818	X144.2176	Y15.0988
N4820	X144.2367	Y15.1458
N4822	X144.2556	Y15.1921
N4824	X144.2740	Y15.2376
N4826	X144.2922	Y15.2823
N4828	X144.3100	Y15.3263
N4830	X144.3275	Y15.3697
N4832	X144.3448	Y15.4123
N4834	X144.3618	Y15.4543
N4836	X144.3785	Y15.4957
N4838	X144.3950	Y15.5364
N4840	X144.4113	Y15.5765
N4842	X144.4274	Y15.6161
N4844	X144.4432	Y15.6550
N4846	X144.4589	Y15.6935
N4848	X144.4745	Y15.7314
N4850	X144.4898	Y15.7687
N4852	X144.5051	Y15.8056
N4854	X144.5202	Y15.8421
N4856	X144.5352	Y15.8781
N4858	X144.5502	Y15.9136
N4860	X144.5651	Y15.9488
N4862	X144.5799	Y15.9835
N4864	X144.5946	Y16.0179

N4866	X144.6094	Y16.0520
N4868	X144.6241	Y16.0857
N4870	X144.6388	Y16.1191
N4872	X144.6535	Y16.1522
N4874	X144.6683	Y16.1850
N4876	X144.6831	Y16.2176
N4878	X144.6980	Y16.2499
N4880	X144.7129	Y16.2820
N4882	X144.7280	Y16.3140
N4884	X144.7431	Y16.3457
N4886	X144.7584	Y16.3773
N4888	X144.7738	Y16.4088
N4890	X144.7893	Y16.4401
N4892	X144.8051	Y16.4714
N4894	X144.8210	Y16.5025
N4896	X144.8370	Y16.5336
N4898	X144.8533	Y16.5647
N4900	X144.8699	Y16.5957
N4902	X144.8866	Y16.6268
N4904	X144.9036	Y16.6578
N4906	X144.9209	Y16.6889
N4908	X144.9385	Y16.7201
N4910	X144.9564	Y16.7513
N4912	X144.9745	Y16.7827
N4914	X144.9931	Y16.8141
N4916	X145.0119	Y16.8457
N4918	X145.0311	Y16.8774
N4920	X145.0507	Y16.9093
N4922	X145.0707	Y16.9414

N4924	X145.0911	Y16.9737
N4926	X145.1118	Y17.0063
N4928	X145.1329	Y17.0390
N4930	X145.1544	Y17.0720
N4932	X145.1762	Y17.1053
N4934	X145.1984	Y17.1388
N4936	X145.2209	Y17.1725
N4938	X145.2436	Y17.2066
N4940	X145.2667	Y17.2409
N4942	X145.2900	Y17.2755
N4944	X145.3136	Y17.3104
N4946	X145.3375	Y17.3456
N4948	X145.3616	Y17.3812
N4950	X145.3859	Y17.4170
N4952	X145.4104	Y17.4532
N4954	X145.4351	Y17.4897
N4956	X145.4600	Y17.5266
N4958	X145.4851	Y17.5639
N4960	X145.5103	Y17.6015
N4962	X145.5356	Y17.6395
N4964	X145.5611	Y17.6778
N4966	X145.5867	Y17.7166
N4968	X145.6124	Y17.7558
N4970	X145.6382	Y17.7954
N4972	X145.6640	Y17.8354
N4974	X145.6899	Y17.8758
N4976	X145.7159	Y17.9167
N4978	X145.7419	Y17.9581
N4980	X145.7679	Y17.9998

N4982	X145.7939	Y18.0421
N4984	X145.8199	Y18.0848
N4986	X145.8459	Y18.1281
N4988	X145.8719	Y18.1718
N4990	X145.8978	Y18.2160
N4992	X145.9236	Y18.2607
N4994	X145.9494	Y18.3059
N4996	X145.9751	Y18.3517
N4998	X146.0006	Y18.3980
N5000	X146.0261	Y18.4448
N5002	X146.0514	Y18.4922
N5004	X146.0766	Y18.5402
N5006	X146.1016	Y18.5887
N5008	X146.1265	Y18.6379
N5010	X146.1512	Y18.6876
N5012	X146.1756	Y18.7379
N5014	X146.1999	Y18.7888
N5016	X146.2239	Y18.8403
N5018	X146.2477	Y18.8924
N5020	X146.2713	Y18.9452
N5022	X146.2946	Y18.9987
N5024	X146.3176	Y19.0527
N5026	X146.3403	Y19.1075
N5028	X146.3627	Y19.1629
N5030	X146.3848	Y19.2190
N5032	X146.4066	Y19.2757
N5034	X146.4280	Y19.3332
N5036	X146.4490	Y19.3914
N5038	X146.4697	Y19.4502

N5040	X146.4900	Y19.5098
N5042	X146.5099	Y19.5702
N5044	X146.5294	Y19.6312
N5046	X146.5484	Y19.6930
N5048	X146.5670	Y19.7556
N5050	X146.5852	Y19.8190
N5052	X146.6029	Y19.8831
N5054	X146.6201	Y19.9480
N5056	X146.6368	Y20.0137
N5058	X146.6530	Y20.0801
N5060	X146.6687	Y20.1474
N5062	X146.6839	Y20.2154
N5064	X146.6986	Y20.2843
N5066	X146.7128	Y20.3538
N5068	X146.7265	Y20.4242
N5070	X146.7397	Y20.4953
N5072	X146.7524	Y20.5671
N5074	X146.7646	Y20.6397
N5076	X146.7762	Y20.7130
N5078	X146.7874	Y20.7870
N5080	X146.7981	Y20.8617
N5082	X146.8082	Y20.9372
N5084	X146.8178	Y21.0133
N5086	X146.8270	Y21.0902
N5088	X146.8356	Y21.1677
N5090	X146.8437	Y21.2459
N5092	X146.8512	Y21.3248
N5094	X146.8583	Y21.4044
N5096	X146.8649	Y21.4846

N5098	X146.8709	Y21.5655
N5100	X146.8764	Y21.6470
N5102	X146.8815	Y21.7292
N5104	X146.8859	Y21.8120
N5106	X146.8899	Y21.8954
N5108	X146.8934	Y21.9794
N5110	X146.8963	Y22.0641
N5112	X146.8988	Y22.1493
N5114	X146.9007	Y22.2352
N5116	X146.9020	Y22.3217
N5118	X146.9029	Y22.4087
N5120	X146.9032	Y22.4963
N5122	X146.9031	Y22.5845
N5124	X146.9024	Y22.6733
N5126	X146.9011	Y22.7626
N5128	X146.8994	Y22.8524
N5130	X146.8971	Y22.9428
N5132	X146.8943	Y23.0338
N5134	X146.8910	Y23.1252
N5136	X146.8871	Y23.2172
N5138	X146.8827	Y23.3097
N5140	X146.8778	Y23.4028
N5142	X146.8724	Y23.4963
N5144	X146.8664	Y23.5903
N5146	X146.8599	Y23.6848
N5148	X146.8529	Y23.7798
N5150	X146.8453	Y23.8752
N5152	X146.8372	Y23.9712
N5154	X146.8286	Y24.0676

N5156 X146.8194 Y24.1644

N5158 X146.8098 Y24.2617

N5160 X146.7995 Y24.3594

N5162 X146.7888 Y24.4576

N5164 X146.7775 Y24.5562

N5166 X146.7656 Y24.6552

N5168 X146.7533 Y24.7546

N5170 X146.7404 Y24.8544

N5172 X146.7269 Y24.9546

N5174 X146.7129

N5176 X146.6984 Y25.1562

N5178 X146.6833 Y25.2576

N5180 X146.6677 Y25.3594

N5182 X146.6516 Y25.4615

N5184 X146.6349 Y25.5639

N5186 X146.6177 Y25.6668

N5188 X146.5999 Y25.7699

N5190 X146.5816 Y25.8734

N5192 X146.5627 Y25.9773

N5194 X146.5432 Y26.0815

N5196 X146.5232 Y26.1860

N5198 X146.5025 Y26.2910

N5200 X146.4811 Y26.3963

N5202 X146.4591 Y26.5021

N5204 X146.4364 Y26.6082

N5206 X146.4131 Y26.7148

N5208 X146.3889 Y26.8218

N5210 X146.3641 Y26.9292

N5212 X146.3384 Y27.0371

N5214	X146.3120	Y27.1455
N5216	X146.2848	Y27.2544
N5218	X146.2567	Y27.3637
N5220	X146.2278	Y27.4736
N5222	X146.1980	Y27.5839
N5224	X146.1673	Y27.6948
N5226	X146.1357	Y27.8062
N5228	X146.1032	Y27.9182
N5230	X146.0697	Y28.0307
N5232	X146.0353	Y28.1438
N5234	X145.9998	Y28.2575
N5236	X145.9633	Y28.3718
N5238	X145.9258	Y28.4867
N5240	X145.8872	Y28.6022
N5242	X145.8476	Y28.7183
N5244	X145.8068	Y28.8351
N5246	X145.7650	Y28.9525
N5248	X145.7220	Y29.0705
N5250	X145.6778	Y29.1893
N5252	X145.6324	Y29.3087
N5254	X145.5859	Y29.4288
N5256	X145.5381	Y29.5497
N5258	X145.4890	Y29.6712
N5260	X145.4387	Y29.7935
N5262	X145.3871	Y29.9165
N5264	X145.3343	Y30.0402
N5266	X145.2800	Y30.1648
N5268	X145.2245	Y30.2901
N5270	X145.1675	Y30.4161

N5272	X145.1092	Y30.5430
N5274	X145.0494	Y30.6707
N5276	X144.9883	Y30.7992
N5278	X144.9256	Y30.9285
N5280	X144.8615	Y31.0587
N5282	X144.7959	Y31.1898
N5284	X144.7288	Y31.3216
N5286	X144.6602	Y31.4544
N5288	X144.5900	Y31.5881
N5290	X144.5182	Y31.7226
N5292	X144.4448	Y31.8581
N5294	X144.3698	Y31.9945
N5296	X144.2932	Y32.1318
N5298	X144.2149	Y32.2700
N5300	X144.1349	Y32.4092
N5302	X144.0532	Y32.5494
N5304	X143.9698	Y32.6906
N5306	X143.8847	Y32.8327
N5308	X143.7978	Y32.9758
N5310	X143.7091	Y33.1200
N5312	X143.6186	Y33.2652
N5314	X143.5262	Y33.4114
N5316	X143.4321	Y33.5586
N5318	X143.3360	Y33.7069
N5320	X143.2381	Y33.8563
N5322	X143.1383	Y34.0067
N5324	X143.0365	Y34.1583
N5326	X142.9329	Y34.3109
N5328	X142.8273	Y34.4645

N5330 X142.7199 Y34.6192

N5332 X142.6107 Y34.7748

N5334 X142.4996 Y34.9314

N5336 X142.3868 Y35.0890

N5338 X142.2722 Y35.2475

N5340 X142.1558 Y35.4069

N5342 X142.0378 Y35.5672

N5344 X141.9180 Y35.7283

N5346 X141.7965 Y35.8903

N5348 X141.6734 Y36.0531

N5350 X141.5486 Y36.2167

N5352 X141.4222 Y36.3810

N5354 X141.2942 Y36.5461

N5356 X141.1647 Y36.7120

N5358 X141.0336 Y36.8785

N5360 X140.9010 Y37.0457

N5362 X140.7668 Y37.2136

N5364 X140.6312 Y37.3821

N5366 X140.4941 Y37.5512

N5368 X140.3556 Y37.7209

N5370 X140.2157 Y37.8911

N5372 X140.0743 Y38.0619

N5374 X139.9316 Y38.2332

N5376 X139.7875 Y38.4051

N5378 X139.6421 Y38.5774

N5380 X139.4954 Y38.7501

N5382 X139.3474 Y38.9233

N5384 X139.1982 Y39.0969

N5386 X139.0477 Y39.2709

N5388	X138.8960	Y39.4452
N5390	X138.7431	Y39.6199
N5392	X138.5890	Y39.7948
N5394	X138.4337	Y39.9701
N5396	X138.2773	Y40.1457
N5398	X138.1198	Y40.3215
N5400	X137.9613	Y40.4975
N5402	X137.8016	Y40.6738
N5404	X137.6409	Y40.8502
N5406	X137.4792	Y41.0268
N5408	X137.3165	Y41.2035
N5410	X137.1528	Y41.3803
N5412	X136.9882	Y41.5572
N5414	X136.8226	Y41.7342
N5416	X136.6561	Y41.9113
N5418	X136.4887	Y42.0883
N5420	X136.3204	Y42.2654
N5422	X136.1513	Y42.4424
N5424	X135.9814	Y42.6194
N5426	X135.8107	Y42.7963
N5428	X135.6391	Y42.9732
N5430	X135.4669	Y43.1499
N5432	X135.2939	Y43.3265
N5434	X135.1201	Y43.5029
N5436	X134.9457	Y43.6792
N5438	X134.7706	Y43.8552
N5440	X134.5949	Y44.0311
N5442	X134.4185	Y44.2067
N5444	X134.2416	Y44.3820

N5446 X134.0640 Y44.5570

N5448 X133.8859 Y44.7317

N5450 X133.7072 Y44.9060

N5452 X133.5281 Y45.0800

N5454 X133.3484 Y45.2537

N5456 X133.1683 Y45.4269

N5458 X132.9877 Y45.5997

N5460 X132.8067 Y45.7720

N5462 X132.6253 Y45.9437

N5464 X132.4437 Y46.1150

N5466 X132.2617 Y46.2856

N5468 X132.0795 Y46.4555

N5470 X131.8971 Y46.6248

N5472 X131.7145 Y46.7934

N5474 X131.5317 Y46.9612

N5476 X131.3489 Y47.1282

N5478 X131.1660 Y47.2943

N5480 X130.9832 Y47.4595

N5482 X130.8003 Y47.6238

N5484 X130.6175 Y47.7872

N5486 X130.4348 Y47.9495

N5488 X130.2522 Y48.1107

N5490 X130.0698 Y48.2709

N5492 X129.8877 Y48.4299

N5494 X129.7058 Y48.5877

N5496 X129.5241 Y48.7443

N5498 X129.3429 Y48.8996

N5500 X129.1620 Y49.0537

N5502 X128.9815 Y49.2063

N5504	X128.8014	Y49.3576
N5506	X128.6219	Y49.5075
N5508	X128.4429	Y49.6559
N5510	X128.2644	Y49.8027
N5512	X128.0866	Y49.9481
N5514	X127.9094	Y50.0918
N5516	X127.7329	Y50.2338
N5518	X127.5571	Y50.3742
N5520	X127.3821	Y50.5129
N5522	X127.2079	Y50.6498
N5524	X127.0345	Y50.7849
N5526	X126.8620	Y50.9182
N5528	X126.6904	Y51.0495
N5530	X126.5198	Y51.1790
N5532	X126.3502	Y51.3065
N5534	X126.1816	Y51.4319
N5536	X126.0141	Y51.5553
N5538	X125.8477	Y51.6766
N5540	X125.6825	Y51.7958
N5542	X125.5184	Y51.9128
N5544	X125.3556	Y52.0275
N5546	X125.1941	Y52.1401
N5548	X125.0338	Y52.2503
N5550	X124.8749	Y52.3581
N5552	X124.7174	Y52.4636
N5554	X124.5613	Y52.5666
N5556	X124.4067	Y52.6672
N5558	X124.2536	Y52.7653
N5560	X124.1020	Y52.8608

N5562	X123.9520	Y52.9537
N5564	X123.8037	Y53.0440
N5566	X123.6570	Y53.1316
N5568	X123.5120	Y53.2165
N5570	X123.3687	Y53.2987
N5572	X123.2272	Y53.3780
N5574	X123.0875	Y53.4545
N5576	X122.9497	Y53.5281
N5578	X122.8137	Y53.5988
N5580	X122.6797	Y53.6665
N5582	X122.5477	Y53.7312
N5584	X122.4177	Y53.7929
N5586	X122.2897	Y53.8515
N5588	X122.1638	Y53.9069
N5590	X122.0401	Y53.9592
N5592	X121.9184	Y54.0083
N5594	X121.7989	Y54.0543
N5596	X121.6814	Y54.0973
N5598	X121.5659	Y54.1372
N5600	X121.4525	Y54.1742
N5602	X121.3411	Y54.2083
N5604	X121.2317	Y54.2394
N5606	X121.1242	Y54.2678
N5608	X121.0187	Y54.2933
N5610	X120.9151	Y54.3161
N5612	X120.8133	Y54.3363
N5614	X120.7135	Y54.3537
N5616	X120.6154	Y54.3686
N5618	X120.5192	Y54.3809

N5620	X120.4248	Y54.3906
N5622	X120.3321	Y54.3979
N5624	X120.2412	Y54.4028
N5626	X120.1520	Y54.4053
N5628	X120.0645	Y54.4055
N5630	X119.9787	Y54.4033
N5632	X119.8945	Y54.3990
N5634	X119.8120	Y54.3924
N5636	X119.7311	Y54.3837
N5638	X119.6517	Y54.3728
N5640	X119.5740	Y54.3599
N5642	X119.4977	Y54.3450
N5644	X119.4230	Y54.3281
N5646	X119.3497	Y54.3092
N5648	X119.2780	Y54.2885
N5650	X119.2076	Y54.2660
N5652	X119.1387	Y54.2416
N5654	X119.0712	Y54.2155
N5656	X119.0051	Y54.1878
N5658	X118.9403	Y54.1583
N5660	X118.8768	Y54.1272
N5662	X118.8147	Y54.0946
N5664	X118.7538	Y54.0604
N5666	X118.6942	Y54.0248
N5668	X118.6358	Y53.9878
N5670	X118.5786	Y53.9493
N5672	X118.5226	Y53.9095
N5674	X118.4678	Y53.8684
N5676	X118.4142	Y53.8261

N5678 X118.3616 Y53.7825

N5680 X118.3101 Y53.7378

N5682 X118.2597 Y53.6920

N5684 X118.2104 Y53.6451

N5686 X118.1621 Y53.5972

N5688 X118.1148 Y53.5483

N5690 X118.0684 Y53.4985

N5692 X118.0230 Y53.4478

N5694 X117.9786 Y53.3962

N5696 X117.9350 Y53.3439

N5698 X117.8924 Y53.2908

N5700 X117.8506 Y53.2369

N5702 X117.8096 Y53.1825

N5704 X117.7695 Y53.1274

N5706 X117.7301 Y53.0717

N5708 X117.6916 Y53.0155

N5710 X117.6537 Y52.9588

N5712 X117.6166 Y52.9017

N5714 X117.5802 Y52.8442

N5716 X117.5445 Y52.7863

N5718 X117.5094 Y52.7281

N5720 X117.4750 Y52.6697

N5722 X117.4411 Y52.6111

N5724 X117.4078 Y52.5523

N5726 X117.3752 Y52.4933

N5728 X117.3431 Y52.4342

N5730 X117.3115 Y52.3749

N5732 X117.2806 Y52.3154

N5734 X117.2502 Y52.2558

N5736 X117.2204 Y52.1960

N5738 X117.1911 Y52.1360

N5740 X117.1624 Y52.0758

N5742 X117.1342 Y52.0155

N5744 X117.1066 Y51.9550

N5746 X117.0795 Y51.8944

N5748 X117.0530 Y51.8336

N5750 X117.0270 Y51.7726

N5752 X117.0015 Y51.7114

N5754 X116.9766 Y51.6501

N5756 X116.9522 Y51.5886

N5758 X116.9283 Y51.5269

N5760 X116.9049 Y51.4651

N5762 X116.8820 Y51.4031

N5764 X116.8597 Y51.3409

N5766 X116.8378 Y51.2786

N5768 X116.8165 Y51.2161

N5770 X116.7957 Y51.1534

N5772 X116.7753 Y51.0906

N5774 X116.7555 Y51.0276

N5776 X116.7361 Y50.9644

N5778 X116.7172 Y50.9011

N5780 X116.6988 Y50.8375

N5782 X116.6809 Y50.7739

N5784 X116.6634 Y50.7100

N5786 X116.6464 Y50.6460

N5788 X116.6299 Y50.5818

N5790 X116.6139 Y50.5175

N5792 X116.5983 Y50.4530

N5794	X116.5831	Y50.3883
N5796	X116.5684	Y50.3234
N5798	X116.5542	Y50.2584
N5800	X116.5404	Y50.1932
N5802	X116.5270	Y50.1279
N5804	X116.5141	Y50.0624
N5806	X116.5016	Y49.9967
N5808	X116.4896	Y49.9308
N5810	X116.4779	Y49.8648
N5812	X116.4667	Y49.7986
N5814	X116.4559	Y49.7323
N5816	X116.4455	Y49.6657
N5818	X116.4355	Y49.5991
N5820	X116.4260	Y49.5322
N5822	X116.4168	Y49.4652
N5824	X116.4080	Y49.3980
N5826	X116.3997	Y49.3306
N5828	X116.3917	Y49.2631
N5830	X116.3841	Y49.1954
N5832	X116.3769	Y49.1276
N5834	X116.3700	Y49.0595
N5836	X116.3636	Y48.9913
N5838	X116.3575	Y48.9230
N5840	X116.3518	Y48.8545
N5842	X116.3464	Y48.7858
N5844	X116.3415	Y48.7169
N5846	X116.3368	Y48.6479
N5848	X116.3326	Y48.5787
N5850	X116.3286	Y48.5093

N5852 X116.3251 Y48.4398

N5854 X116.3218 Y48.3701

N5856 X116.3189 Y48.3003

N5858 X116.3164 Y48.2303

N5860 X116.3141 Y48.1601

N5862 X116.3122 Y48.0897

N5864 X116.3105 Y48.0192

N5866 X116.3091 Y47.9485

N5868 X116.3080 Y47.8776

N5870 X116.3071 Y47.8065

N5872 X116.3064 Y47.7353

N5874 X116.3059 Y47.6638

N5876 X116.3055 Y47.5922

N5878 X116.3054 Y47.5204

N5880 X116.3054 Y47.4484

N5882 X116.3055 Y47.3763

N5884 X116.3058 Y47.3039

N5886 X116.3062 Y47.2313

N5888 X116.3066 Y47.1586

N5890 X116.3071 Y47.0856

N5892 X116.3077 Y47.0125

N5894 X116.3083 Y46.9391

N5896 X116.3089 Y46.8655

N5898 X116.3095 Y46.7918

N5900 X116.3102 Y46.7178

N5902 X116.3107 Y46.6436

N5904 X116.3113 Y46.5693

N5906 X116.3118 Y46.4947

N5908 X116.3122 Y46.4198

N5910 X116.3125 Y46.3448

N5912 X116.3127 Y46.2696

N5914 X116.3127 Y46.1941

N5916 X116.3127 Y46.1184

N5918 X116.3124 Y46.0425

N5920 X116.3120 Y45.9663

N5922 X116.3114 Y45.8900

N5924 X116.3106 Y45.8134

N5926 X116.3096 Y45.7366

N5928 X116.3083 Y45.6595

N5930 X116.3068 Y45.5822

N5932 X116.3050 Y45.5047

N5934 X116.3029 Y45.4269

N5936 X116.3005 Y45.3489

N5938 X116.2978 Y45.2706

N5940 X116.2947 Y45.1921

N5942 X116.2913 Y45.1134

N5944 X116.2875 Y45.0344

N5946 X116.2833 Y44.9551

N5948 X116.2787 Y44.8756

N5950 X116.2737 Y44.7959

N5952 X116.2683 Y44.7159

N5954 X116.2624 Y44.6356

N5956 X116.2560 Y44.5551

N5958 X116.2492 Y44.4743

N5960 X116.2418 Y44.3932

N5962 X116.2339 Y44.3119

N5964 X116.2255 Y44.2304

N5966 X116.2165 Y44.1485

N5968 X116.2070 Y44.0664

N5970 X116.1969 Y43.9840

N5972 X116.1862 Y43.9013

N5974 X116.1748 Y43.8184

N5976 X116.1629 Y43.7351

N5978 X116.1503 Y43.6516

N5980 X116.1370 Y43.5679

N5982 X116.1230 Y43.4838

N5984 X116.1083 Y43.3994

N5986 X116.0930 Y43.3148

N5988 X116.0768 Y43.2298

N5990 X116.0600 Y43.1446

N5992 X116.0423 Y43.0591

N5994 X116.0239 Y42.9732

N5996 X116.0047 Y42.8871

N5998 X115.9847 Y42.8006

N6000 X115.9638 Y42.7137

N6002 X115.9420 Y42.6265

N6004 X115.9194 Y42.5388

N6006 X115.8959 Y42.4508

N6008 X115.8715 Y42.3624

N6010 X115.8461 Y42.2736

N6012 X115.8198 Y42.1843

N6014 X115.7925 Y42.0946

N6016 X115.7642 Y42.0044

N6018 X115.7349 Y41.9138

N6020 X115.7045 Y41.8226

N6022 X115.6731 Y41.7310

N6024 X115.6407 Y41.6388

N6026 X115.6071 Y41.5462

N6028 X115.5725 Y41.4530

N6030 X115.5367 Y41.3592

N6032 X115.4998 Y41.2649

N6034 X115.4617 Y41.1700

N6036 X115.4224 Y41.0744

N6038 X115.3820 Y40.9783

N6040 X115.3403 Y40.8816

N6042 X115.2974 Y40.7843

N6044 X115.2532 Y40.6862

N6046 X115.2077 Y40.5876

N6048 X115.1610 Y40.4882

N6050 X115.1129 Y40.3882

N6052 X115.0635 Y40.2875

N6054 X115.0128 Y40.1861

N6056 X114.9607 Y40.0839

N6058 X114.9072 Y39.9810

N6060 X114.8522 Y39.8774

N6062 X114.7959 Y39.7730

N6064 X114.7381 Y39.6678

N6066 X114.6789 Y39.5618

N6068 X114.6181 Y39.4551

N6070 X114.5559 Y39.3475

N6072 X114.4921 Y39.2391

N6074 X114.4268 Y39.1298

N6076 X114.3600 Y39.0197

N6078 X114.2915 Y38.9087

N6080 X114.2215 Y38.7968

N6082 X114.1498 Y38.6840

N6084 X114.0765 Y38.5703

N6086 X114.0016 Y38.4557

N6088 X113.9250 Y38.3402

N6090 X113.8467 Y38.2237

N6092 X113.7667 Y38.1063

N6094 X113.6849 Y37.9878

N6096 X113.6014 Y37.8684

N6098 X113.5162 Y37.7480

N6100 X113.4291 Y37.6266

N6102 X113.3403 Y37.5041

N6104 X113.2496 Y37.3806

N6106 X113.1571 Y37.2561

N6108 X113.0627 Y37.1304

N6110 X112.9665 Y37.0037

N6112 X112.8683 Y36.8759

N6114 X112.7682 Y36.7470

N6116 X112.6662 Y36.6170

N6118 X112.5622 Y36.4858

N6120 X112.4563 Y36.3535

N6122 X112.3484 Y36.2200

N6124 X112.2384 Y36.0854

N6126 X112.1265 Y35.9496

N6128 X112.0128 Y35.8127

N6130 X111.8971 Y35.6747

N6132 X111.7797 Y35.5357

N6134 X111.6606 Y35.3956

N6136 X111.5397 Y35.2545

N6138 X111.4173 Y35.1124

N6140 X111.2933 Y34.9694

N6142	X111.1678	Y34.8254
N6144	X111.0409	Y34.6806
N6146	X110.9125	Y34.5348
N6148	X110.7828	Y34.3883
N6150	X110.6519	Y34.2409
N6152	X110.5197	Y34.0927
N6154	X110.3864	Y33.9438
N6156	X110.2520	Y33.7941
N6158	X110.1165	Y33.6438
N6160	X109.9800	Y33.4927
N6162	X109.8426	Y33.3411
N6164	X109.7043	Y33.1888
N6166	X109.5651	Y33.0359
N6168	X109.4252	Y32.8824
N6170	X109.2846	Y32.7284
N6172	X109.1434	Y32.5739
N6174	X109.0015	Y32.4189
N6176	X108.8591	Y32.2634
N6178	X108.7162	Y32.1076
N6180	X108.5729	Y31.9513
N6182	X108.4292	Y31.7946
N6184	X108.2852	Y31.6376
N6186	X108.1410	Y31.4803
N6188	X107.9965	Y31.3227
N6190	X107.8519	Y31.1649
N6192	X107.7072	Y31.0068
N6194	X107.5625	Y30.8485
N6196	X107.4178	Y30.6900
N6198	X107.2732	Y30.5314

N6200	X107.1287	Y30.3726
N6202	X106.9844	Y30.2138
N6204	X106.8403	Y30.0549
N6206	X106.6966	Y29.8959
N6208	X106.5532	Y29.7369
N6210	X106.4103	Y29.5780
N6212	X106.2678	Y29.4191
N6214	X106.1259	Y29.2602
N6216	X105.9845	Y29.1015
N6218	X105.8438	Y28.9429
N6220	X105.7038	Y28.7844
N6222	X105.5646	Y28.6261
N6224	X105.4262	Y28.4681
N6226	X105.2886	Y28.3102
N6228	X105.1520	Y28.1526
N6230	X105.0164	Y27.9954
N6232	X104.8818	Y27.8384
N6234	X104.7483	Y27.6818
N6236	X104.6160	Y27.5255
N6238	X104.4848	Y27.3697
N6240	X104.3550	Y27.2143
N6242	X104.2265	Y27.0593
N6244	X104.0993	Y26.9049
N6246	X103.9736	Y26.7509
N6248	X103.8494	Y26.5975
N6250	X103.7267	Y26.4447
N6252	X103.6057	Y26.2924
N6254	X103.4863	Y26.1408
N6256	X103.3686	Y25.9898

N6258 X103.2527 Y25.8395

N6260 X103.1385 Y25.6899

N6262 X103.0261 Y25.5410

N6264 X102.9153 Y25.3927

N6266 X102.8063 Y25.2452

N6268 X102.6989 Y25.0983

N6270 X102.5932 Y24.9522

N6272 X102.4891 Y24.8068

N6274 X102.3867 Y24.6622

N6276 X102.2859 Y24.5183

N6278 X102.1867 Y24.3751

N6280 X102.0890 Y24.2327

N6282 X101.9929 Y24.0911

N6284 X101.8984 Y23.9502

N6286 X101.8054 Y23.8101

N6288 X101.7140 Y23.6708

N6290 X101.6240 Y23.5322

N6292 X101.5355 Y23.3945

N6294 X101.4485 Y23.2576

N6296 X101.3630 Y23.1215

N6298 X101.2789 Y22.9862

N6300 X101.1962 Y22.8518

N6302 X101.1149 Y22.7182

N6304 X101.0350 Y22.5854

N6306 X100.9565 Y22.4535

N6308 X100.8794 Y22.3224

N6310 X100.8036 Y22.1923

N6312 X100.7291 Y22.0630

N6314 X100.6560 Y21.9345

N6316	X100.5841	Y21.8070
N6318	X100.5136	Y21.6804
N6320	X100.4443	Y21.5546
N6322	X100.3762	Y21.4298
N6324	X100.3094	Y21.3059
N6326	X100.2438	Y21.1829
N6328	X100.1794	Y21.0609
N6330	X100.1162	Y20.9398
N6332	X100.0542	Y20.8197
N6334	X99.9934	Y20.7005
N6336	X99.9336	Y20.5823
N6338	X99.8750	Y20.4650
N6340	X99.8175	Y20.3488
N6342	X99.7612	Y20.2335
N6344	X99.7058	Y20.1192
N6346	X99.6516	Y20.0060
N6348	X99.5984	Y19.8937
N6350	X99.5462	Y19.7824
N6352	X99.4951	Y19.6722
N6354	X99.4449	Y19.5630
N6356	X99.3957	Y19.4549
N6358	X99.3475	Y19.3478
N6360	X99.3003	Y19.2418
N6362	X99.2539	Y19.1368
N6364	X99.2085	Y19.0329
N6366	X99.1641	Y18.9300
N6368	X99.1205	Y18.8283
N6370	X99.0777	Y18.7276
N6372	X99.0359	Y18.6281

N6374	X98.9948	Y18.5297
N6376	X98.9546	Y18.4323
N6378	X98.9153	Y18.3361
N6380	X98.8767	Y18.2411
N6382	X98.8388	Y18.1471
N6384	X98.8018	Y18.0543
N6386	Y17.9627	
N6388	X98.7299	Y17.8722
N6390	X98.6951	Y17.7829
N6392	X98.6609	Y17.6948
N6394	X98.6274	Y17.6078
N6396	X98.5946	Y17.5220
N6398	X98.5625	Y17.4374
N6400	X98.5309	Y17.3539
N6402	X98.5000	Y17.2715
N6404	X98.4697	Y17.1903
N6406	X98.4399	Y17.1102
N6408	X98.4107	Y17.0313
N6410	X98.3820	Y16.9535
N6412	X98.3538	Y16.8768
N6414	X98.3262	Y16.8012
N6416	X98.2990	Y16.7268
N6418	X98.2723	Y16.6534
N6420	X98.2460	Y16.5812
N6422	X98.2202	Y16.5101
N6424	X98.1947	Y16.4401
N6426	X98.1697	Y16.3711
N6428	X98.1450	Y16.3033
N6430	X98.1207	Y16.2365

N6432	X98.0967	Y16.1709
N6434	X98.0730	Y16.1063
N6436	X98.0497	Y16.0428
N6438	X98.0266	Y15.9803
N6440	X98.0038	Y15.9189
N6442	X97.9812	Y15.8586
N6444	X97.9588	Y15.7993
N6446	X97.9367	Y15.7411
N6448	X97.9147	Y15.6839
N6450	X97.8929	Y15.6278
N6452	X97.8713	Y15.5727
N6454	X97.8498	Y15.5186
N6456	X97.8285	Y15.4656
N6458	X97.8072	Y15.4136
N6460	X97.7860	Y15.3626
N6462	X97.7649	Y15.3126
N6464	X97.7438	Y15.2637
N6466	X97.7227	Y15.2157
N6468	X97.7017	Y15.1688
N6470	X97.6806	Y15.1229
N6472	X97.6595	Y15.0779
N6474	X97.6384	Y15.0339
N6476	X97.6172	Y14.9910
N6478	X97.5959	Y14.9490
N6480	X97.5745	Y14.9080
N6482	X97.5530	Y14.8679
N6484	X97.5314	Y14.8289
N6486	X97.5096	Y14.7907
N6488	X97.4877	Y14.7536

N6490	X97.4655	Y14.7174
N6492	X97.4431	Y14.6822
N6494	X97.4205	Y14.6479
N6496	X97.3977	Y14.6145
N6498	X97.3746	Y14.5821
N6500	X97.3512	Y14.5506
N6502	X97.3275	Y14.5201
N6504	X97.3035	Y14.4904
N6506	X97.2792	Y14.4617
N6508	X97.2545	Y14.4340
N6510	X97.2294	Y14.4071
N6512	X97.2040	Y14.3811
N6514	X97.1781	Y14.3561
N6516	X97.1518	Y14.3319
N6518	X97.1251	Y14.3086
N6520	X97.0979	Y14.2862
N6522	X97.0702	Y14.2647
N6524	X97.0420	Y14.2441
N6526	X97.0134	Y14.2244
N6528	X96.9842	Y14.2054
N6530	X96.9546	Y14.1873
N6532	X96.9245	Y14.1699
N6534	X96.8940	Y14.1532
N6536	X96.8631	Y14.1372
N6538	X96.8318	Y14.1219
N6540	X96.8001	Y14.1072
N6542	X96.7680	Y14.0932
N6544	X96.7355	Y14.0797
N6546	X96.7027	Y14.0667

N6548	X96.6695	Y14.0542
N6550	X96.6360	Y14.0422
N6552	X96.6021	Y14.0307
N6554	X96.5680	Y14.0195
N6556	X96.5336	Y14.0088
N6558	X96.4989	Y13.9984
N6560	X96.4639	Y13.9883
N6562	X96.4287	Y13.9785
N6564	X96.3932	Y13.9690
N6566	X96.3575	Y13.9596
N6568	X96.3216	Y13.9505
N6570	X96.2855	Y13.9415
N6572	X96.2493	Y13.9327
N6574	X96.2128	Y13.9240
N6576	X96.1762	Y13.9153
N6578	X96.1394	Y13.9067
N6580	X96.1025	Y13.8980
N6582	X96.0655	Y13.8894
N6584	X96.0284	Y13.8806
N6586	X95.9912	Y13.8718
N6588	X95.9539	Y13.8629
N6590	X95.9165	Y13.8538
N6592	X95.8791	Y13.8445
N6594	X95.8417	Y13.8350
N6596	X95.8042	Y13.8253
N6598	X95.7667	Y13.8153
N6600	X95.7292	Y13.8050
N6602	X95.6917	Y13.7943
N6604	X95.6542	Y13.7832

N6606	X95.6168	Y13.7718
N6608	X95.5794	Y13.7599
N6610	X95.5421	Y13.7476
N6612	X95.5049	Y13.7347
N6614	X95.4678	Y13.7213
N6616	X95.4307	Y13.7074
N6618	X95.3938	Y13.6929
N6620	X95.3570	Y13.6777
N6622	X95.3204	Y13.6619
N6624	X95.2839	Y13.6454
N6626	X95.2476	Y13.6281
N6628	X95.2114	Y13.6102
N6630	X95.1755	Y13.5914
N6632	X95.1398	Y13.5718
N6634	X95.1043	Y13.5514
N6636	X95.0690	Y13.5301
N6638	X95.0340	Y13.5079
N6640	X94.9992	Y13.4848
N6642	X94.9647	Y13.4607
N6644	X94.9305	Y13.4356
N6646	X94.8966	Y13.4094
N6648	X94.8631	Y13.3822
N6650	X94.8298	Y13.3539
N6652	X94.7969	Y13.3245
N6654	X94.7644	Y13.2939
N6656	X94.7322	Y13.2621
N6658	X94.7004	Y13.2291
N6660	X94.6689	Y13.1950
N6662	X94.6377	Y13.1597

N6664	X94.6067	Y13.1233
N6666	X94.5760	Y13.0858
N6668	X94.5454	Y13.0472
N6670	X94.5150	Y13.0076
N6672	X94.4847	Y12.9671
N6674	X94.4545	Y12.9255
N6676	X94.4242	Y12.8831
N6678	X94.3940	Y12.8397
N6680	X94.3637	Y12.7955
N6682	X94.3333	Y12.7504
N6684	X94.3028	Y12.7046
N6686	X94.2721	Y12.6579
N6688	X94.2412	Y12.6105
N6690	X94.2101	Y12.5624
N6692	X94.1786	Y12.5136
N6694	X94.1469	Y12.4642
N6696	X94.1148	Y12.4141
N6698	X94.0823	Y12.3634
N6700	X94.0493	Y12.3122
N6702	X94.0159	Y12.2605
N6704	X93.9820	Y12.2082
N6706	X93.9475	Y12.1555
N6708	X93.9124	Y12.1023
N6710	X93.8767	Y12.0488
N6712	X93.8404	Y11.9948
N6714	X93.8033	Y11.9406
N6716	X93.7655	Y11.8860
N6718	X93.7269	Y11.8311
N6720	X93.6875	Y11.7759

N6722	X93.6472	Y11.7206
N6724	X93.6060	Y11.6650
N6726	X93.5639	Y11.6093
N6728	X93.5209	Y11.5535
N6730	X93.4768	Y11.4975
N6732	X93.4316	Y11.4415
N6734	X93.3854	Y11.3854
N6736	X93.3381	Y11.3294
N6738	X93.2895	Y11.2734
N6740	X93.2398	Y11.2174
N6742	X93.1888	Y11.1615
N6744	X93.1366	Y11.1057
N6746	X93.0830	Y11.0501
N6748	X93.0281	Y10.9946
N6750	X92.9718	Y10.9393
N6752	X92.9141	Y10.8843
N6754	X92.8548	Y10.8296
N6756	X92.7941	Y10.7751
N6758	X92.7318	Y10.7210
N6760	X92.6680	Y10.6673
N6762	X92.6025	Y10.6139
N6764	X92.5353	Y10.5610
N6766	X92.4665	Y10.5085
N6768	X92.3959	Y10.4565
N6770	X92.3235	Y10.4050
N6772	X92.2493	Y10.3541
N6774	X92.1733	Y10.3037
N6776	X92.0953	Y10.2540
N6778	X92.0154	Y10.2049

N6780 X91.9336 Y10.1564

N6782 X91.8498 Y10.1087

N6784 X91.7639 Y10.0617

N6786 X91.6759 Y10.0154

N6788 X91.5858 Y9.9700

N6790 X91.4935 Y9.9253

N6792 X91.3991 Y9.8815

N6794 X91.3027 Y9.8386

N6796 X91.2043 Y9.7965

N6798 X91.1040 Y9.7553

N6800 X91.0019 Y9.7149

N6802 X90.8981 Y9.6754

N6804 X90.7926 Y9.6369

N6806 X90.6856 Y9.5992

N6808 X90.5771 Y9.5624

N6810 X90.4673 Y9.5265

N6812 X90.3561 Y9.4916

N6814 X90.2437 Y9.4576

N6816 X90.1302 Y9.4245

N6818 X90.0156 Y9.3924

N6820 X89.9001 Y9.3612

N6822 X89.7837 Y9.3310

N6824 X89.6664 Y9.3018

N6826 X89.5484 Y9.2736

N6828 X89.4298 Y9.2463

N6830 X89.3107 Y9.2201

N6832 X89.1910 Y9.1948

N6834 X89.0710 Y9.1706

N6836 X88.9507 Y9.1473

N6838 X88.8301 Y9.1251

N6840 X88.7094 Y9.1040

N6842 X88.5886 Y9.0839

N6844 X88.4679 Y9.0648

N6846 X88.3472 Y9.0468

N6848 X88.2268 Y9.0299

N6850 X88.1066 Y9.0141

N6852 X87.9868 Y8.9993

N6854 X87.8674 Y8.9856

N6856 X87.7485 Y8.9731

N6858 X87.6303 Y8.9616

N6860 X87.5127 Y8.9513

N6862 X87.3959 Y8.9421

N6864 Y8.9340

N6866 X87.1649 Y8.9271

N6868 X87.0509 Y8.9213

N6870 X86.9380 Y8.9167

N6872 X86.8263 Y8.9133

N6874 X86.7158 Y8.9110

N6876 X86.6067 Y8.9099

N6878 X86.4991 Y8.9101

N6880 X86.3929 Y8.9114

N6882 X86.2883 Y8.9139

N6884 X86.1855 Y8.9177

N6886 X86.0844 Y8.9226

N6888 X85.9851 Y8.9288

N6890 X85.8877 Y8.9363

N6892 X85.7924 Y8.9450

N6894 X85.6992 Y8.9549

N6896	X85.6081	Y8.9662
N6898	X85.5194	Y8.9787
N6900	X85.4329	Y8.9924
N6902	X85.3489	Y9.0075
N6904	X85.2674	Y9.0239
N6906	X85.1885	Y9.0416
N6908	X85.1122	Y9.0606
N6910	X85.0388	Y9.0809
N6912	X84.9681	Y9.1025
N6914	X84.9004	Y9.1255
N6916	X84.8357	Y9.1499
N6918	X84.7741	Y9.1756
N6920	X84.7156	Y9.2027
N6922	X84.6605	Y9.2311
N6924	X84.6086	Y9.2609
N6926	X84.5600	Y9.2921
N6928	X84.5147	Y9.3246
N6930	X84.4726	Y9.3583
N6932	X84.4336	Y9.3934
N6934	X84.3976	Y9.4296
N6936	X84.3647	Y9.4670
N6938	X84.3347	Y9.5055
N6940	X84.3077	Y9.5452
N6942	X84.2834	Y9.5859
N6944	X84.2620	Y9.6277
N6946	X84.2432	Y9.6704
N6948	X84.2272	Y9.7142
N6950	X84.2137	Y9.7588
N6952	X84.2028	Y9.8044

N6954	X84.1944	Y9.8508
N6956	X84.1884	Y9.8980
N6958	X84.1848	Y9.9460
N6960	X84.1835	Y9.9948
N6962	X84.1844	Y10.0443
N6964	X84.1876	Y10.0945
N6966	X84.1929	Y10.1453
N6968	X84.2003	Y10.1968
N6970	X84.2098	Y10.2488
N6972	X84.2212	Y10.3014
N6974	X84.2345	Y10.3544
N6976	X84.2496	Y10.4080
N6978	X84.2666	Y10.4620
N6980	X84.2853	Y10.5164
N6982	X84.3057	Y10.5711
N6984	X84.3278	Y10.6262
N6986	X84.3513	Y10.6816
N6988	X84.3764	Y10.7373
N6990	X84.4030	Y10.7931
N6992	X84.4309	Y10.8492
N6994	X84.4602	Y10.9054
N6996	X84.4908	Y10.9618
N6998	X84.5226	Y11.0182
N7000	X84.5555	Y11.0747
N7002	X84.5896	Y11.1312
N7004	X84.6247	Y11.1877
N7006	X84.6607	Y11.2441
N7008	X84.6978	Y11.3005
N7010	X84.7357	Y11.3567

N7012	X84.7744	Y11.4127
N7014	X84.8138	Y11.4686
N7016	X84.8540	Y11.5242
N7018	X84.8948	Y11.5796
N7020	X84.9362	Y11.6347
N7022	X84.9782	Y11.6894
N7024	X85.0206	Y11.7437
N7026	X85.0634	Y11.7977
N7028	X85.1065	Y11.8512
N7030	X85.1500	Y11.9042
N7032	X85.1937	Y11.9568
N7034	X85.2376	Y12.0087
N7036	X85.2816	Y12.0601
N7038	X85.3257	Y12.1109
N7040	X85.3697	Y12.1610
N7042	X85.4138	Y12.2105
N7044	X85.4577	Y12.2592
N7046	X85.5015	Y12.3071
N7048	X85.5450	Y12.3543
N7050	X85.5883	Y12.4006
N7052	X85.6312	Y12.4461
N7054	X85.6737	Y12.4907
N7056	X85.7158	Y12.5343
N7058	X85.7573	Y12.5770
N7060	X85.7984	Y12.6188
N7062	X85.8390	Y12.6596
N7064	X85.8791	Y12.6995
N7066	X85.9186	Y12.7386
N7068	X85.9577	Y12.7767

N7070	X85.9963	Y12.8140
N7072	X86.0344	Y12.8505
N7074	X86.0720	Y12.8862
N7076	X86.1091	Y12.9211
N7078	X86.1458	Y12.9552
N7080	X86.1819	Y12.9885
N7082	X86.2175	Y13.0211
N7084	X86.2527	Y13.0530
N7086	X86.2874	Y13.0841
N7088	X86.3216	Y13.1146
N7090	X86.3553	Y13.1444
N7092	X86.3885	Y13.1736
N7094	X86.4213	Y13.2021
N7096	X86.4536	Y13.2300
N7098	X86.4854	Y13.2574
N7100	X86.5167	Y13.2841
N7102	X86.5476	Y13.3103
N7104	X86.5779	Y13.3359
N7106	X86.6078	Y13.3611
N7108	X86.6373	Y13.3857
N7110	X86.6663	Y13.4098
N7112	X86.6947	Y13.4335
N7114	X86.7228	Y13.4567
N7116	X86.7503	Y13.4795
N7118	X86.7774	Y13.5018
N7120	X86.8041	Y13.5238
N7122	X86.8303	Y13.5454
N7124	X86.8560	Y13.5666
N7126	X86.8812	Y13.5875

N7128	X86.9060	Y13.6081
N7130	X86.9304	Y13.6283
N7132	X86.9542	Y13.6483
N7134	X86.9777	Y13.6680
N7136	X87.0006	Y13.6875
N7138	X87.0232	Y13.7067
N7140	X87.0452	Y13.7257
N7142	X87.0668	Y13.7445
N7144	X87.0880	Y13.7632
N7146	X87.1087	Y13.7816
N7148	X87.1290	Y13.8000
N7150	X87.1488	Y13.8182
N7152	X87.1682	Y13.8363
N7154	X87.1872	Y13.8543
N7156	X87.2057	Y13.8723
N7158	X87.2237	Y13.8902
N7160	X87.2413	Y13.9081
N7162	X87.2585	Y13.9259
N7164	X87.2753	Y13.9438
N7166	X87.2916	Y13.9617
N7168	X87.3074	Y13.9796
N7170	X87.3229	Y13.9976
N7172	X87.3379	Y14.0156
N7174	X87.3525	Y14.0338
N7176	X87.3666	Y14.0520
N7178	X87.3803	Y14.0704
N7180	X87.3936	Y14.0890
N7182	X87.4065	Y14.1077
N7184	X87.4189	Y14.1266

N7186	X87.4310	Y14.1457
N7188	X87.4426	Y14.1650
N7190	X87.4537	Y14.1846
N7192	X87.4645	Y14.2044
N7194	X87.4750	Y14.2246
N7196	X87.4852	Y14.2452
N7198	X87.4951	Y14.2662
N7200	X87.5050	Y14.2878
N7202	X87.5147	Y14.3099
N7204	X87.5244	Y14.3326
N7206	X87.5341	Y14.3561
N7208	X87.5439	Y14.3802
N7210	X87.5539	Y14.4052
N7212	X87.5640	Y14.4311
N7214	X87.5744	Y14.4578
N7216	X87.5852	Y14.4856
N7218	X87.5963	Y14.5143
N7220	X87.6079	Y14.5442
N7222	X87.6200	Y14.5753
N7224	X87.6326	Y14.6075
N7226	X87.6459	Y14.6410
N7228	X87.6599	Y14.6759
N7230	X87.6746	Y14.7121
N7232	X87.6901	Y14.7498
N7234	X87.7064	Y14.7890
N7236	X87.7237	Y14.8298
N7238	X87.7420	Y14.8721
N7240	X87.7613	Y14.9162
N7242	X87.7817	Y14.9620

N7244	X87.8033	Y15.0096
N7246	X87.8261	Y15.0590
N7248	X87.8501	Y15.1104
N7250	X87.8755	Y15.1638
N7252	X87.9024	Y15.2191
N7254	X87.9306	Y15.2766
N7256	X87.9604	Y15.3362
N7258	X87.9918	Y15.3980
N7260	X88.0248	Y15.4621
N7262	X88.0595	Y15.5285
N7264	X88.0960	Y15.5973
N7266	X88.1343	Y15.6686
N7268	X88.1744	Y15.7423
N7270	X88.2165	Y15.8186
N7272	X88.2606	Y15.8975
N7274	X88.3068	Y15.9791
N7276	X88.3551	Y16.0634
N7278	X88.4055	Y16.1505
N7280	X88.4582	Y16.2405
N7282	X88.5132	Y16.3334
N7284	X88.5705	Y16.4292
N7286	X88.6303	Y16.5281
N7288	X88.6925	Y16.6300
N7290	X88.7573	Y16.7351
N7292	X88.8246	Y16.8434
N7294	X88.8946	Y16.9550
N7296	X88.9673	Y17.0699
N7298	X89.0428	Y17.1881
N7300	X89.1212	Y17.3098

N7302	X89.2024	Y17.4350
N7304	X89.2866	Y17.5637
N7306	X89.3737	Y17.6960
N7308	X89.4640	Y17.8321
N7310	X89.5574	Y17.9718
N7312	X89.6540	Y18.1153
N7314	X89.7538	Y18.2627
N7316	X89.8570	Y18.4140
N7318	X89.9635	Y18.5692
N7320	X90.0734	Y18.7284
N7322	X90.1868	Y18.8918
N7324	X90.3038	Y19.0592
N7326	X90.4243	Y19.2307
N7328	X90.5481	Y19.4062
N7330	X90.6751	Y19.5855
N7332	X90.8054	Y19.7686
N7334	X90.9386	Y19.9553
N7336	X91.0749	Y20.1456
N7338	X91.2139	Y20.3393
N7340	X91.3557	Y20.5363
N7342	X91.5001	Y20.7365
N7344	X91.6469	Y20.9399
N7346	X91.7962	Y21.1462
N7348	X91.9478	Y21.3555
N7350	X92.1016	Y21.5675
N7352	X92.2575	Y21.7823
N7354	X92.4153	Y21.9996
N7356	X92.5750	Y22.2194
N7358	X92.7364	Y22.4416

N7360	X92.8995	Y22.6660
N7362	X93.0642	Y22.8927
N7364	X93.2302	Y23.1213
N7366	X93.3976	Y23.3520
N7368	X93.5663	Y23.5845
N7370	X93.7360	Y23.8187
N7372	X93.9067	Y24.0545
N7374	X94.0784	Y24.2919
N7376	X94.2508	Y24.5307
N7378	X94.4239	Y24.7709
N7380	X94.5976	Y25.0122
N7382	X94.7717	Y25.2546
N7384	X94.9462	Y25.4981
N7386	X95.1210	Y25.7425
N7388	X95.2959	Y25.9876
N7390	X95.4709	Y26.2334
N7392	X95.6458	Y26.4798
N7394	X95.8205	Y26.7267
N7396	X95.9949	Y26.9740
N7398	X96.1689	Y27.2215
N7400	X96.3424	Y27.4692
N7402	X96.5154	Y27.7169
N7404	X96.6876	Y27.9646
N7406	X96.8590	Y28.2121
N7408	X97.0294	Y28.4593
N7410	X97.1989	Y28.7062
N7412	X97.3671	Y28.9526
N7414	X97.5342	Y29.1985
N7416	X97.6998	Y29.4436

N7418 X97.8640 Y29.6879

N7420 X98.0267 Y29.9314

N7422 X98.1876 Y30.1738

N7424 X98.3468 Y30.4152

N7426 X98.5041 Y30.6553

N7428 X98.6593 Y30.8941

N7430 X98.8125 Y31.1315

N7432 X98.9634 Y31.3673

N7434 X99.1121 Y31.6016

N7436 X99.2582 Y31.8340

N7438 X99.4019 Y32.0646

N7440 X99.5429 Y32.2933

N7442 X99.6811 Y32.5199

N7444 X99.8165 Y32.7444

N7446 X99.9489 Y32.9665

N7448 X100.0782 Y33.1863

N7450 X100.2044 Y33.4037

N7452 X100.3273 Y33.6184

N7454 X100.4467 Y33.8304

N7456 X100.5627 Y34.0397

N7458 X100.6750 Y34.2461

N7460 X100.7838 Y34.4497

N7462 X100.8891 Y34.6505

N7464 X100.9909 Y34.8487

N7466 X101.0893 Y35.0444

N7468 X101.1842 Y35.2375

N7470 X101.2758 Y35.4283

N7472 X101.3640 Y35.6167

N7474 X101.4489 Y35.8029

N7476	X101.5306	Y35.9870
N7478	X101.6090	Y36.1689
N7480	X101.6842	Y36.3488
N7482	X101.7563	Y36.5269
N7484	X101.8253	Y36.7030
N7486	X101.8912	Y36.8774
N7488	X101.9540	Y37.0502
N7490	X102.0138	Y37.2213
N7492	X102.0707	Y37.3909
N7494	X102.1246	Y37.5590
N7496	X102.1757	Y37.7258
N7498	X102.2239	Y37.8913
N7500	X102.2692	Y38.0555
N7502	X102.3118	Y38.2187
N7504	X102.3517	Y38.3808
N7506	X102.3888	Y38.5419
N7508	X102.4232	Y38.7022
N7510	X102.4550	Y38.8616
N7512	X102.4843	Y39.0204
N7514	X102.5109	Y39.1784
N7516	X102.5351	Y39.3359
N7518	X102.5567	Y39.4929
N7520	X102.5759	Y39.6495
N7522	X102.5927	Y39.8058
N7524	X102.6071	Y39.9618
N7526	X102.6191	Y40.1177
N7528	X102.6289	Y40.2734
N7530	X102.6364	Y40.4292
N7532	X102.6417	Y40.5850

N7534 X102.6448 Y40.7410

N7536 X102.6457 Y40.8972

N7538 X102.6445 Y41.0537

N7540 X102.6412 Y41.2106

N7542 X102.6359 Y41.3680

N7544 X102.6286 Y41.5259

N7546 X102.6193 Y41.6845

N7548 X102.6081 Y41.8437

N7550 X102.5949 Y42.0038

N7552 X102.5800 Y42.1647

N7554 X102.5632 Y42.3266

N7556 X102.5446 Y42.4895

N7558 X102.5243 Y42.6535

N7560 X102.5022 Y42.8186

N7562 X102.4785 Y42.9851

N7564 X102.4532 Y43.1529

N7566 X102.4262 Y43.3221

N7568 X102.3977 Y43.4929

N7570 X102.3677 Y43.6652

N7572 X102.3362 Y43.8392

N7574 X102.3032 Y44.0149

N7576 X102.2688 Y44.1925

N7578 X102.2330 Y44.3719

N7580 X102.1959 Y44.5534

N7582 X102.1575 Y44.7369

N7584 X102.1179 Y44.9225

N7586 X102.0770 Y45.1104

N7588 X102.0349 Y45.3006

N7590 X101.9916 Y45.4932

N7592	X101.9472	Y45.6881
N7594	X101.9017	Y45.8854
N7596	X101.8549	Y46.0847
N7598	X101.8070	Y46.2862
N7600	X101.7578	Y46.4895
N7602	X101.7073	Y46.6947
N7604	X101.6555	Y46.9016
N7606	X101.6025	Y47.1101
N7608	X101.5481	Y47.3200
N7610	X101.4923	Y47.5314
N7612	X101.4352	Y47.7440
N7614	X101.3767	Y47.9577
N7616	X101.3167	Y48.1725
N7618	X101.2553	Y48.3882
N7620	X101.1924	Y48.6047
N7622	X101.1280	Y48.8219
N7624	X101.0621	Y49.0397
N7626	X100.9946	Y49.2579
N7628	X100.9255	Y49.4765
N7630	X100.8549	Y49.6954
N7632	X100.7827	Y49.9144
N7634	X100.7088	Y50.1334
N7636	X100.6332	Y50.3523
N7638	X100.5559	Y50.5710
N7640	X100.4770	Y50.7894
N7642	X100.3963	Y51.0074
N7644	X100.3138	Y51.2248
N7646	X100.2295	Y51.4416
N7648	X100.1435	Y51.6576

N7650	X100.0556	Y51.8728
N7652	X99.9658	Y52.0869
N7654	X99.8742	Y52.2999
N7656	X99.7806	Y52.5118
N7658	X99.6851	Y52.7223
N7660	X99.5877	Y52.9313
N7662	X99.4883	Y53.1388
N7664	X99.3869	Y53.3446
N7666	X99.2835	Y53.5487
N7668	X99.1780	Y53.7509
N7670	X99.0705	Y53.9510
N7672	X98.9608	Y54.1491
N7674	X98.8491	Y54.3449
N7676	X98.7352	Y54.5384
N7678	X98.6191	Y54.7294
N7680	X98.5008	Y54.9178
N7682	X98.3803	Y55.1036
N7684	X98.2576	Y55.2866
N7686	X98.1327	Y55.4667
N7688	X98.0054	Y55.6437
N7690	X97.8758	Y55.8177
N7692	X97.7439	Y55.9884
N7694	X97.6097	Y56.1557
N7696	X97.4730	Y56.3196
N7698	X97.3340	Y56.4799
N7700	X97.1925	Y56.6365
N7702	X97.0486	Y56.7893
N7704	X96.9021	Y56.9382
N7706	X96.7532	Y57.0831

N7708	X96.6018	Y57.2238
N7710	X96.4478	Y57.3603
N7712	X96.2913	Y57.4924
N7714	X96.1321	Y57.6200
N7716	X95.9704	Y57.7431
N7718	X95.8060	Y57.8614
N7720	X95.6389	Y57.9749
N7722	X95.4691	Y58.0835
N7724	X95.2967	Y58.1871
N7726	X95.1215	Y58.2856
N7728	X94.9437	Y58.3793
N7730	X94.7633	Y58.4681
N7732	Y58.5521	
N7734	Y58.6316	
N7736	X94.2069	Y58.7064
N7738	X94.0166	Y58.7767
N7740	X93.8238	Y58.8427
N7742	X93.6288	Y58.9044
N7744	X93.4314	Y58.9618
N7746	X93.2319	Y59.0151
N7748	X93.0301	Y59.0643
N7750	X92.8261	Y59.1096
N7752	X92.6201	Y59.1511
N7754	X92.4120	Y59.1887
N7756	X92.2018	Y59.2227
N7758	X91.9897	Y59.2530
N7760	X91.7757	Y59.2798
N7762	X91.5598	Y59.3032
N7764	X91.3420	Y59.3232

N7766	X91.1224	Y59.3400
N7768	X90.9011	Y59.3536
N7770	X90.6781	Y59.3641
N7772	X90.4534	Y59.3717
N7774	X90.2271	Y59.3763
N7776	X89.9992	Y59.3781
N7778	X89.7698	Y59.3771
N7780	X89.5389	Y59.3735
N7782	X89.3066	Y59.3674
N7784	X89.0729	Y59.3588
N7786	X88.8378	Y59.3477
N7788	X88.6014	Y59.3344
N7790	X88.3638	Y59.3189
N7792	X88.1249	Y59.3013
N7794	X87.8849	Y59.2816
N7796	X87.6437	Y59.2600
N7798	X87.4015	Y59.2365
N7800	X87.1582	Y59.2112
N7802	X86.9139	Y59.1843
N7804	X86.6687	Y59.1557
N7806	X86.4226	Y59.1257
N7808	X86.1756	Y59.0942
N7810	X85.9278	Y59.0614
N7812	X85.6792	Y59.0273
N7814	X85.4299	Y58.9920
N7816	X85.1799	Y58.9557
N7818	X84.9293	Y58.9184
N7820	X84.6780	Y58.8802
N7822	X84.4263	Y58.8412

N7824	X84.1740	Y58.8015
N7826	X83.9213	Y58.7611
N7828	X83.6681	Y58.7202
N7830	X83.4146	Y58.6788
N7832	X83.1608	Y58.6370
N7834	X82.9066	Y58.5950
N7836	X82.6523	Y58.5527
N7838	X82.3977	Y58.5104
N7840	X82.1430	Y58.4680
N7842	X81.8882	Y58.4256
N7844	X81.6334	Y58.3834
N7846	X81.3785	Y58.3415
N7848	X81.1237	Y58.2998
N7850	X80.8689	Y58.2586
N7852	X80.6142	Y58.2179
N7854	X80.3598	Y58.1777
N7856	X80.1055	Y58.1383
N7858	X79.8515	Y58.0995
N7860	X79.5978	Y58.0615
N7862	X79.3444	Y58.0242
N7864	X79.0915	Y57.9874
N7866	X78.8392	Y57.9512
N7868	X78.5873	Y57.9156
N7870	X78.3361	Y57.8804
N7872	X78.0856	Y57.8456
N7874	X77.8358	Y57.8113
N7876	X77.5868	Y57.7773
N7878	X77.3387	Y57.7435
N7880	X77.0915	Y57.7101

N7882	X76.8453	Y57.6768
N7884	X76.6001	Y57.6437
N7886	X76.3561	Y57.6107
N7888	X76.1132	Y57.5777
N7890	X75.8715	Y57.5448
N7892	X75.6311	Y57.5119
N7894	X75.3921	Y57.4789
N7896	X75.1545	Y57.4457
N7898	X74.9184	Y57.4124
N7900	X74.6837	Y57.3789
N7902	X74.4507	Y57.3451
N7904	X74.2194	Y57.3111
N7906	X73.9897	Y57.2766
N7908	X73.7619	Y57.2418
N7910	X73.5358	Y57.2066
N7912	X73.3117	Y57.1708
N7914	X73.0895	Y57.1346
N7916	X72.8694	Y57.0977
N7918	X72.6513	Y57.0603
N7920	X72.4354	Y57.0221
N7922	X72.2217	Y56.9833
N7924	X72.0102	Y56.9437
N7926	X71.8011	Y56.9032
N7928	X71.5943	Y56.8620
N7930	X71.3900	Y56.8198
N7932	X71.1883	Y56.7767
N7934	X70.9890	Y56.7326
N7936	X70.7924	Y56.6874
N7938	X70.5985	Y56.6412

N7940 X70.4074 Y56.5939

N7942 X70.2190 Y56.5453

N7944 X70.0336 Y56.4956

N7946 X69.8510 Y56.4446

N7948 X69.6715 Y56.3923

N7950 X69.4950 Y56.3386

N7952 X69.3216 Y56.2835

N7954 X69.1514 Y56.2270

N7956 X68.9845 Y56.1689

N7958 X68.8208 Y56.1094

N7960 X68.6605 Y56.0482

N7962 X68.5037 Y55.9854

N7964 X68.3503 Y55.9210

N7966 X68.2004 Y55.8548

N7968 X68.0542 Y55.7868

N7970 X67.9116 Y55.7170

N7972 X67.7727 Y55.6454

N7974 X67.6377 Y55.5718

N7976 X67.5065 Y55.4963

N7978 X67.3791 Y55.4188

N7980 X67.2558 Y55.3393

N7982 X67.1365 Y55.2577

N7984 X67.0213 Y55.1739

N7986 X66.9102 Y55.0879

N7988 X66.8033 Y54.9997

N7990 X66.7007 Y54.9093

N7992 X66.6024 Y54.8165

N7994 X66.5083 Y54.7216

N7996 X66.4184 Y54.6245

N7998	X66.3326	Y54.5253
N8000	X66.2507	Y54.4241
N8002	X66.1729	Y54.3210
N8004	X66.0989	Y54.2160
N8006	X66.0287	Y54.1092
N8008	X65.9623	Y54.0006
N8010	X65.8996	Y53.8904
N8012	X65.8405	Y53.7785
N8014	X65.7849	Y53.6651
N8016	X65.7328	Y53.5502
N8018	X65.6842	Y53.4340
N8020	X65.6389	Y53.3164
N8022	X65.5968	Y53.1975
N8024	X65.5580	Y53.0774
N8026	X65.5223	Y52.9562
N8028	X65.4897	Y52.8339
N8030	X65.4602	Y52.7106
N8032	X65.4335	Y52.5863
N8034	X65.4098	Y52.4612
N8036	X65.3888	Y52.3353
N8038	X65.3706	Y52.2087
N8040	X65.3551	Y52.0814
N8042	X65.3422	Y51.9535
N8044	X65.3318	Y51.8250
N8046	X65.3239	Y51.6961
N8048	X65.3184	Y51.5668
N8050	X65.3153	Y51.4372
N8052	X65.3144	Y51.3073
N8054	X65.3157	Y51.1772

N8056	X65.3192	Y51.0469
N8058	X65.3248	Y50.9167
N8060	X65.3323	Y50.7864
N8062	X65.3418	Y50.6562
N8064	X65.3532	Y50.5261
N8066	X65.3663	Y50.3962
N8068	X65.3812	Y50.2666
N8070	X65.3978	Y50.1374
N8072	X65.4160	Y50.0085
N8074	X65.4357	Y49.8802
N8076	X65.4568	Y49.7523
N8078	X65.4794	Y49.6251
N8080	X65.5033	Y49.4986
N8082	X65.5285	Y49.3728
N8084	X65.5549	Y49.2478
N8086	X65.5824	Y49.1237
N8088	X65.6110	Y49.0006
N8090	X65.6405	Y48.8784
N8092	X65.6710	Y48.7574
N8094	X65.7024	Y48.6374
N8096	X65.7346	Y48.5187
N8098	X65.7674	Y48.4013
N8100	X65.8010	Y48.2853
N8102	X65.8351	Y48.1706
N8104	X65.8698	Y48.0574
N8106	X65.9050	Y47.9458
N8108	X65.9405	Y47.8358
N8110	X65.9763	Y47.7275
N8112	X66.0124	Y47.6210

N8114	X66.0488	Y47.5162
N8116	X66.0852	Y47.4134
N8118	X66.1217	Y47.3125
N8120	X66.1581	Y47.2136
N8122	X66.1945	Y47.1168
N8124	X66.2308	Y47.0222
N8126	X66.2669	Y46.9297
N8128	X66.3028	Y46.8393
N8130	X66.3385	Y46.7510
N8132	X66.3741	Y46.6647
N8134	X66.4094	Y46.5804
N8136	X66.4445	Y46.4981
N8138	X66.4794	Y46.4176
N8140	X66.5141	Y46.3389
N8142	X66.5486	Y46.2621
N8144	X66.5829	Y46.1871
N8146	X66.6168	Y46.1138
N8148	X66.6506	Y46.0421
N8150	X66.6841	Y45.9721
N8152	X66.7173	Y45.9037
N8154	X66.7503	Y45.8368
N8156	X66.7830	Y45.7715
N8158	X66.8154	Y45.7076
N8160	X66.8475	Y45.6451
N8162	X66.8793	Y45.5840
N8164	X66.9109	Y45.5243
N8166	X66.9421	Y45.4658
N8168	X66.9730	Y45.4086
N8170	X67.0035	Y45.3527

N8172	X67.0338	Y45.2978
N8174	X67.0637	Y45.2441
N8176	X67.0932	Y45.1915
N8178	X67.1224	Y45.1400
N8180	X67.1513	Y45.0894
N8182	X67.1797	Y45.0398
N8184	X67.2079	Y44.9911
N8186	X67.2356	Y44.9432
N8188	X67.2629	Y44.8962
N8190	X67.2899	Y44.8500
N8192	X67.3164	Y44.8045
N8194	X67.3426	Y44.7597
N8196	X67.3683	Y44.7156
N8198	X67.3936	Y44.6721
N8200	X67.4185	Y44.6291
N8202	X67.4429	Y44.5867
N8204	Y44.5448	
N8206	Y44.5033	
N8208	X67.5136	Y44.4622
N8210	X67.5362	Y44.4214
N8212	X67.5584	Y44.3810
N8214	X67.5801	Y44.3408
N8216	X67.6013	Y44.3009
N8218	X67.6220	Y44.2611
N8220	X67.6422	Y44.2215
N8222	X67.6619	Y44.1820
N8224	X67.6811	Y44.1426
N8226	X67.6998	Y44.1032
N8228	X67.7180	Y44.0637

N8230	X67.7356	Y44.0242
N8232	X67.7527	Y43.9845
N8234	X67.7692	Y43.9447
N8236	X67.7852	Y43.9047
N8238	X67.8006	Y43.8645
N8240	X67.8155	Y43.8239
N8242	X67.8298	Y43.7831
N8244	X67.8435	Y43.7419
N8246	X67.8566	Y43.7002
N8248	X67.8691	Y43.6581
N8250	X67.8810	Y43.6155
N8252	X67.8923	Y43.5724
N8254	X67.9030	Y43.5287
N8256	X67.9131	Y43.4843
N8258	X67.9226	Y43.4393
N8260	X67.9314	Y43.3936
N8262	X67.9397	Y43.3472
N8264	X67.9474	Y43.3000
N8266	X67.9547	Y43.2520
N8268	X67.9614	Y43.2032
N8270	X67.9677	Y43.1536
N8272	X67.9736	Y43.1031
N8274	X67.9790	Y43.0517
N8276	X67.9841	Y42.9994
N8278	X67.9889	Y42.9461
N8280	X67.9934	Y42.8918
N8282	X67.9976	Y42.8365
N8284	X68.0015	Y42.7802
N8286	X68.0053	Y42.7227

N8288	X68.0088	Y42.6642
N8290	X68.0122	Y42.6046
N8292	X68.0155	Y42.5438
N8294	X68.0186	Y42.4818
N8296	X68.0217	Y42.4185
N8298	X68.0248	Y42.3541
N8300	X68.0278	Y42.2883
N8302	X68.0309	Y42.2213
N8304	X68.0340	Y42.1529
N8306	X68.0372	Y42.0832
N8308	X68.0405	Y42.0120
N8310	X68.0439	Y41.9395
N8312	X68.0475	Y41.8655
N8314	X68.0514	Y41.7900
N8316	X68.0554	Y41.7130
N8318	X68.0597	Y41.6345
N8320	X68.0643	Y41.5544
N8322	X68.0692	Y41.4727
N8324	X68.0744	Y41.3894
N8326	X68.0800	Y41.3044
N8328	X68.0860	Y41.2178
N8330	X68.0925	Y41.1294
N8332	X68.0994	Y41.0393
N8334	X68.1068	Y40.9475
N8336	X68.1148	Y40.8538
N8338	X68.1232	Y40.7583
N8340	X68.1323	Y40.6610
N8342	X68.1420	Y40.5618
N8344	X68.1523	Y40.4607

N8346	X68.1633	Y40.3576
N8348	X68.1750	Y40.2526
N8350	X68.1874	Y40.1455
N8352	X68.2006	Y40.0365
N8354	X68.2146	Y39.9253
N8356	X68.2294	Y39.8121
N8358	X68.2450	Y39.6968
N8360	X68.2615	Y39.5793
N8362	X68.2790	Y39.4597
N8364	X68.2973	Y39.3379
N8366	X68.3166	Y39.2138
N8368	X68.3370	Y39.0875
N8370	X68.3583	Y38.9589
N8372	X68.3807	Y38.8279
N8374	X68.4042	Y38.6947
N8376	X68.4288	Y38.5590
N8378	X68.4546	Y38.4210
N8380	X68.4815	Y38.2805
N8382	X68.5096	Y38.1375
N8384	X68.5390	Y37.9921
N8386	X68.5696	Y37.8442
N8388	X68.6015	Y37.6937
N8390	X68.6347	Y37.5406
N8392	X68.6693	Y37.3849
N8394	X68.7051	Y37.2268
N8396	X68.7423	Y37.0663
N8398	X68.7807	Y36.9034
N8400	X68.8202	Y36.7384
N8402	X68.8610	Y36.5712

N8404	X68.9029	Y36.4019
N8406	X68.9460	Y36.2307
N8408	X68.9901	Y36.0575
N8410	X69.0353	Y35.8826
N8412	X69.0815	Y35.7060
N8414	X69.1287	Y35.5277
N8416	X69.1768	Y35.3479
N8418	X69.2259	Y35.1667
N8420	X69.2759	Y34.9841
N8422	X69.3268	Y34.8002
N8424	X69.3785	Y34.6151
N8426	X69.4310	Y34.4289
N8428	X69.4843	Y34.2416
N8430	X69.5383	Y34.0535
N8432	X69.5931	Y33.8645
N8434	X69.6485	Y33.6747
N8436	X69.7046	Y33.4842
N8438	X69.7614	Y33.2932
N8440	X69.8187	Y33.1016
N8442	X69.8766	Y32.9096
N8444	X69.9350	Y32.7173
N8446	X69.9939	Y32.5247
N8448	X70.0533	Y32.3320
N8450	X70.1131	Y32.1392
N8452	X70.1733	Y31.9464
N8454	X70.2339	Y31.7537
N8456	X70.2949	Y31.5612
N8458	X70.3562	Y31.3689
N8460	X70.4177	Y31.1770

N8462	X70.4796	Y30.9856
N8464	X70.5416	Y30.7946
N8466	X70.6038	Y30.6043
N8468	X70.6662	Y30.4147
N8470	X70.7288	Y30.2259
N8472	X70.7914	Y30.0379
N8474	X70.8541	Y29.8509
N8476	X70.9168	Y29.6649
N8478	X70.9796	Y29.4800
N8480	X71.0423	Y29.2964
N8482	X71.1050	Y29.1141
N8484	X71.1676	Y28.9331
N8486	X71.2300	Y28.7536
N8488	X71.2924	Y28.5757
N8490	X71.3545	Y28.3995
N8492	X71.4165	Y28.2249
N8494	X71.4782	Y28.0522
N8496	X71.5396	Y27.8814
N8498	X71.6007	Y27.7125
N8500	X71.6616	Y27.5457
N8502	X71.7220	Y27.3811
N8504	X71.7820	Y27.2188
N8506	X71.8417	Y27.0588
N8508	X71.9009	Y26.9012
N8510	X71.9595	Y26.7460
N8512	X72.0177	Y26.5935
N8514	X72.0753	Y26.4437
N8516	X72.1324	Y26.2966
N8518	X72.1888	Y26.1524

N8520	X72.2446	Y26.0111
N8522	X72.2998	Y25.8728
N8524	X72.3542	Y25.7377
N8526	X72.4079	Y25.6056
N8528	X72.4609	Y25.4766
N8530	X72.5132	Y25.3506
N8532	X72.5648	Y25.2275
N8534	X72.6156	Y25.1073
N8536	X72.6656	Y24.9899
N8538	X72.7149	Y24.8753
N8540	X72.7635	Y24.7634
N8542	X72.8113	Y24.6542
N8544	X72.8584	Y24.5475
N8546	X72.9047	Y24.4434
N8548	X72.9502	Y24.3418
N8550	X72.9949	Y24.2426
N8552	X73.0389	Y24.1458
N8554	X73.0820	Y24.0513
N8556	X73.1244	Y23.9591
N8558	X73.1660	Y23.8691
N8560	X73.2068	Y23.7812
N8562	X73.2468	Y23.6954
N8564	X73.2859	
N8566	X73.3243	Y23.5298
N8568	X73.3618	Y23.4499
N8570	X73.3985	Y23.3719
N8572	X73.4344	Y23.2957
N8574	X73.4694	Y23.2213
N8576	X73.5036	Y23.1485

N8578 X73.5370 Y23.0774

N8580 X73.5694 Y23.0079

N8582 X73.6011 Y22.9398

N8584 X73.6319 Y22.8733

N8586 X73.6618 Y22.8082

N8588 X73.6908 Y22.7444

N8590 X73.7190 Y22.6819

N8592 X73.7463 Y22.6206

N8594 X73.7726 Y22.5606

N8596 X73.7982 Y22.5017

N8598 X73.8228 Y22.4438

N8600 X73.8465 Y22.3870

N8602 X73.8693 Y22.3311

N8604 X73.8911 Y22.2761

N8606 X73.9121 Y22.2220

N8608 X73.9322 Y22.1686

N8610 X73.9513 Y22.1160

N8612 X73.9695 Y22.0640

N8614 X73.9867 Y22.0127

N8616 X74.0030 Y21.9620

N8618 X74.0184 Y21.9117

N8620 X74.0328 Y21.8619

N8622 X74.0463 Y21.8125

N8624 X74.0588 Y21.7634

N8626 X74.0703 Y21.7146

N8628 X74.0808 Y21.6661

N8630 X74.0904 Y21.6177

N8632 X74.0990 Y21.5694

N8634 X74.1066 Y21.5212

N8636	X74.1132	Y21.4729
N8638	X74.1188	Y21.4246
N8640	X74.1235	Y21.3762
N8642	X74.1271	Y21.3276
N8644	X74.1297	Y21.2788
N8646	X74.1312	Y21.2297
N8648	X74.1318	Y21.1803
N8650	X74.1313	Y21.1304
N8652	X74.1298	Y21.0801
N8654	X74.1273	Y21.0293
N8656	X74.1237	Y20.9780
N8658	X74.1191	Y20.9260
N8660	X74.1134	Y20.8733
N8662	X74.1067	Y20.8201
N8664	X74.0990	Y20.7662
N8666	X74.0903	Y20.7117
N8668	X74.0806	Y20.6566
N8670	X74.0699	Y20.6008
N8672	X74.0583	Y20.5445
N8674	X74.0457	Y20.4874
N8676	X74.0321	Y20.4298
N8678	X74.0176	Y20.3716
N8680	X74.0022	Y20.3127
N8682	X73.9859	Y20.2532
N8684	X73.9686	Y20.1931
N8686	X73.9505	Y20.1324
N8688	X73.9315	Y20.0710
N8690	X73.9116	Y20.0090
N8692	X73.8909	Y19.9464

N8694	X73.8694	Y19.8832
N8696	X73.8469	Y19.8194
N8698	X73.8237	Y19.7550
N8700	X73.7997	Y19.6899
N8702	X73.7749	Y19.6242
N8704	X73.7492	Y19.5579
N8706	X73.7228	Y19.4910
N8708	X73.6957	Y19.4235
N8710	X73.6677	Y19.3554
N8712	X73.6391	Y19.2867
N8714	X73.6097	Y19.2173
N8716	X73.5796	Y19.1474
N8718	X73.5487	Y19.0768
N8720	X73.5172	Y19.0056
N8722	X73.4850	Y18.9338
N8724	X73.4521	Y18.8614
N8726	X73.4186	Y18.7884
N8728	X73.3844	Y18.7148
N8730	X73.3495	Y18.6406
N8732	X73.3141	Y18.5658
N8734	X73.2780	Y18.4904
N8736	X73.2413	Y18.4144
N8738	X73.2040	Y18.3378
N8740	X73.1661	Y18.2605
N8742	X73.1277	Y18.1827
N8744	X73.0887	Y18.1043
N8746	X73.0491	Y18.0252
N8748	X73.0090	Y17.9456
N8750	X72.9684	Y17.8654

N8752	X72.9272	Y17.7846
N8754	X72.8856	Y17.7031
N8756	X72.8435	Y17.6211
N8758	X72.8008	Y17.5385
N8760	X72.7577	Y17.4553
N8762	X72.7142	Y17.3715
N8764	X72.6702	Y17.2871
N8766	X72.6257	Y17.2021
N8768	X72.5809	Y17.1165
N8770	X72.5356	Y17.0304
N8772	X72.4899	Y16.9436
N8774	X72.4438	Y16.8562
N8776	X72.3974	Y16.7683
N8778	X72.3506	Y16.6798
N8780	X72.3034	Y16.5906
N8782	X72.2558	Y16.5009
N8784	X72.2080	Y16.4106
N8786	X72.1598	Y16.3197
N8788	X72.1113	Y16.2283
N8790	X72.0625	Y16.1362
N8792	X72.0134	Y16.0436
N8794	X71.9640	Y15.9504
N8796	X71.9142	Y15.8567
N8798	X71.8641	Y15.7625
N8800	X71.8137	Y15.6679
N8802	X71.7628	Y15.5729
N8804	X71.7116	Y15.4774
N8806	X71.6599	Y15.3817
N8808	X71.6078	Y15.2856

N8810	X71.5553	Y15.1892
N8812	X71.5022	Y15.0925
N8814	X71.4487	Y14.9957
N8816	X71.3947	Y14.8986
N8818	X71.3401	Y14.8014
N8820	X71.2850	Y14.7040
N8822	X71.2294	Y14.6066
N8824	X71.1731	Y14.5090
N8826	X71.1163	Y14.4115
N8828	X71.0589	Y14.3139
N8830	X71.0008	Y14.2164
N8832	X70.9421	Y14.1189
N8834	X70.8827	Y14.0216
N8836	X70.8226	Y13.9243
N8838	X70.7619	Y13.8272
N8840	X70.7004	Y13.7304
N8842	X70.6381	Y13.6337
N8844	X70.5752	Y13.5373
N8846	X70.5114	Y13.4411
N8848	X70.4469	Y13.3453
N8850	X70.3815	Y13.2499
N8852	X70.3154	Y13.1548
N8854	X70.2483	Y13.0601
N8856	X70.1805	Y12.9659
N8858	X70.1117	Y12.8722
N8860	X70.0421	Y12.7789
N8862	X69.9715	Y12.6862
N8864	X69.9000	Y12.5941
N8866	X69.8276	Y12.5026

N8868	X69.7542	Y12.4118
N8870	X69.6798	Y12.3216
N8872	X69.6044	Y12.2321
N8874	X69.5281	Y12.1433
N8876	X69.4506	Y12.0553
N8878	X69.3721	Y11.9681
N8880	X69.2926	Y11.8818
N8882	X69.2119	Y11.7963
N8884	X69.1302	Y11.7116
N8886	X69.0473	Y11.6280
N8888	X68.9633	Y11.5452
N8890	X68.8781	Y11.4635
N8892	X68.7918	Y11.3828
N8894	X68.7042	Y11.3031
N8896	X68.6155	Y11.2246
N8898	X68.5255	Y11.1471
N8900	X68.4343	Y11.0708
N8902	X68.3418	Y10.9957
N8904	X68.2480	Y10.9218
N8906	X68.1529	Y10.8491
N8908	X68.0565	Y10.7777
N8910	X67.9588	Y10.7077
N8912	X67.8597	Y10.6389
N8914	X67.7592	Y10.5716
N8916	X67.6574	Y10.5056
N8918	X67.5542	Y10.4411
N8920	X67.4495	Y10.3781
N8922	X67.3434	Y10.3166
N8924	X67.2358	Y10.2566

N8926	X67.1269	Y10.1981
N8928	X67.0165	Y10.1412
N8930	X66.9049	Y10.0858
N8932	X66.7920	Y10.0319
N8934	X66.6781	Y9.9795
N8936	X66.5630	Y9.9287
N8938	X66.4470	Y9.8793
N8940	X66.3300	Y9.8315
N8942	X66.2121	Y9.7851
N8944	X66.0935	Y9.7401
N8946	X65.9742	Y9.6967
N8948	X65.8542	Y9.6547
N8950	X65.7337	Y9.6141
N8952	X65.6127	Y9.5750
N8954	X65.4912	Y9.5373
N8956	X65.3694	Y9.5011
N8958	X65.2473	Y9.4662
N8960	X65.1250	Y9.4328
N8962	X65.0025	Y9.4007
N8964	X64.8800	Y9.3701
N8966	X64.7575	Y9.3408
N8968	X64.6350	Y9.3129
N8970	X64.5128	Y9.2864
N8972	X64.3907	Y9.2612
N8974	X64.2689	Y9.2374
N8976	X64.1475	Y9.2149
N8978	X64.0265	Y9.1938
N8980	X63.9060	Y9.1740
N8982	X63.7861	Y9.1555

N8984 X63.6668 Y9.1383

N8986 X63.5483 Y9.1224

N8988 X63.4306 Y9.1078

N8990 X63.3137 Y9.0945

N8992 X63.1977 Y9.0824

N8994 Y9.0717

N8996 Y9.0622

N8998 X62.8562 Y9.0539

N9000 X62.7447 Y9.0469

N9002 X62.6345 Y9.0411

N9004 X62.5256 Y9.0366

N9006 X62.4182 Y9.0332

N9008 X62.3123 Y9.0311

N9010 X62.2080 Y9.0302

N9012 X62.1053 Y9.0305

N9014 X62.0044 Y9.0320

N9016 X61.9052 Y9.0346

N9018 X61.8079 Y9.0384

N9020 X61.7126 Y9.0434

N9022 X61.6192 Y9.0496

N9024 X61.5280 Y9.0568

N9026 X61.4389 Y9.0653

N9028 X61.3520 Y9.0748

N9030 X61.2674 Y9.0855

N9032 X61.1851 Y9.0973

N9034 X61.1053 Y9.1102

N9036 X61.0280 Y9.1242

N9038 X60.9533 Y9.1393

N9040 X60.8813 Y9.1554

N9042	X60.8119	Y9.1727
N9044	X60.7454	Y9.1910
N9046	X60.6817	Y9.2104
N9048	X60.6210	Y9.2308
N9050	X60.5633	Y9.2522
N9052	X60.5087	Y9.2747
N9054	X60.4572	Y9.2982
N9056	X60.4089	Y9.3227
N9058	X60.3639	Y9.3483
N9060	X60.3222	Y9.3748
N9062	X60.2837	Y9.4023
N9064	X60.2484	Y9.4308
N9066	X60.2162	Y9.4603
N9068	X60.1870	Y9.4907
N9070	X60.1609	Y9.5220
N9072	X60.1378	Y9.5543
N9074	X60.1175	Y9.5875
N9076	X60.1001	Y9.6215
N9078	X60.0856	Y9.6565
N9080	X60.0738	Y9.6924
N9082	X60.0648	Y9.7291
N9084	X60.0584	Y9.7666
N9086	X60.0546	Y9.8050
N9088	X60.0534	Y9.8443
N9090	X60.0547	Y9.8843
N9092	X60.0585	Y9.9252
N9094	X60.0647	Y9.9668
N9096	X60.0733	Y10.0093
N9098	X60.0843	Y10.0525

N9100	X60.0975	Y10.0964
N9102	X60.1129	Y10.1411
N9104	X60.1305	Y10.1866
N9106	X60.1502	Y10.2327
N9108	X60.1720	Y10.2796
N9110	X60.1958	Y10.3272
N9112	X60.2216	Y10.3754
N9114	X60.2493	Y10.4243
N9116	X60.2790	Y10.4739
N9118	X60.3104	Y10.5241
N9120	X60.3436	Y10.5750
N9122	X60.3785	Y10.6265
N9124	X60.4152	Y10.6786
N9126	X60.4534	Y10.7313
N9128	X60.4933	Y10.7846
N9130	X60.5346	Y10.8385
N9132	X60.5775	Y10.8929
N9134	X60.6217	Y10.9479
N9136	X60.6674	Y11.0034
N9138	X60.7144	Y11.0595
N9140	X60.7627	Y11.1161
N9142	X60.8122	Y11.1732
N9144	X60.8629	Y11.2308
N9146	X60.9147	Y11.2888
N9148	X60.9676	Y11.3473
N9150	X61.0216	Y11.4063
N9152	X61.0765	Y11.4658
N9154	X61.1324	Y11.5256
N9156	X61.1891	Y11.5859

N9158 X61.2467 Y11.6466

N9160 X61.3051 Y11.7077

N9162 X61.3642 Y11.7692

N9164 X61.4240 Y11.8310

N9166 X61.4845 Y11.8932

N9168 X61.5455 Y11.9558

N9170 X61.6071 Y12.0187

N9172 X61.6692 Y12.0819

N9174 X61.7317 Y12.1454

N9176 X61.7946 Y12.2093

N9178 X61.8578 Y12.2734

N9180 X61.9213 Y12.3378

N9182 X61.9851 Y12.4024

N9184 X62.0491 Y12.4674

N9186 X62.1132 Y12.5325

N9188 X62.1774 Y12.5979

N9190 X62.2416 Y12.6635

N9192 X62.3059 Y12.7293

N9194 X62.3701 Y12.7954

N9196 X62.4343 Y12.8616

N9198 X62.4985 Y12.9281

N9200 X62.5626 Y12.9949

N9202 X62.6266 Y13.0619

N9204 X62.6905 Y13.1291

N9206 X62.7543 Y13.1967

N9208 X62.8179 Y13.2645

N9210 X62.8814 Y13.3326

N9212 X62.9448 Y13.4010

N9214 X63.0079 Y13.4698

N9216	X63.0709	Y13.5388
N9218	X63.1337	Y13.6082
N9220	X63.1962	Y13.6779
N9222	X63.2585	Y13.7480
N9224	X63.3205	Y13.8185
N9226	X63.3822	Y13.8893
N9228	X63.4437	Y13.9604
N9230	X63.5048	Y14.0320
N9232	X63.5656	Y14.1040
N9234	X63.6261	Y14.1764
N9236	X63.6862	Y14.2492
N9238	X63.7459	Y14.3224
N9240	X63.8052	Y14.3961
N9242	X63.8641	Y14.4702
N9244	X63.9226	Y14.5447
N9246	X63.9807	Y14.6197
N9248	X64.0383	Y14.6952
N9250	X64.0954	Y14.7712
N9252	X64.1520	Y14.8477
N9254	X64.2081	Y14.9247
N9256	X64.2637	Y15.0021
N9258	X64.3187	Y15.0801
N9260	X64.3732	Y15.1587
N9262	X64.4272	Y15.2378
N9264	X64.4805	Y15.3174
N9266	X64.5332	Y15.3976
N9268	X64.5853	Y15.4783
N9270	X64.6368	Y15.5597
N9272	X64.6876	Y15.6416

N9274	X64.7377	Y15.7241
N9276	X64.7871	Y15.8072
N9278	X64.8359	Y15.8909
N9280	X64.8839	Y15.9753
N9282	X64.9312	Y16.0603
N9284	X64.9777	Y16.1459
N9286	X65.0234	Y16.2322
N9288	X65.0684	Y16.3192
N9290	X65.1126	Y16.4068
N9292	X65.1559	Y16.4951
N9294	X65.1984	Y16.5841
N9296	X65.2401	Y16.6738
N9298	X65.2809	Y16.7643
N9300	X65.3208	Y16.8554
N9302	X65.3598	Y16.9473
N9304	X65.3979	Y17.0399
N9306	X65.4350	Y17.1332
N9308	X65.4712	Y17.2273
N9310	X65.5064	Y17.3222
N9312	X65.5407	Y17.4179
N9314	X65.5739	Y17.5143
N9316	X65.6062	Y17.6115
N9318	X65.6373	Y17.7096
N9320	X65.6675	Y17.8084
N9322	X65.6966	Y17.9081
N9324	X65.7246	Y18.0086
N9326	X65.7515	Y18.1100
N9328	X65.7773	Y18.2122
N9330	X65.8020	Y18.3152

N9332	X65.8255	Y18.4191
N9334	X65.8479	Y18.5238
N9336	X65.8691	Y18.6294
N9338	X65.8892	Y18.7359
N9340	X65.9080	Y18.8432
N9342	X65.9257	Y18.9513
N9344	X65.9421	Y19.0604
N9346	X65.9573	Y19.1702
N9348	X65.9713	Y19.2810
N9350	X65.9840	Y19.3926
N9352	X65.9955	Y19.5051
N9354	X66.0056	Y19.6185
N9356	X66.0145	Y19.7328
N9358	X66.0221	Y19.8479
N9360	X66.0283	Y19.9639
N9362	X66.0332	Y20.0808
N9364	X66.0368	Y20.1986
N9366	X66.0390	Y20.3173
N9368	X66.0398	Y20.4368
N9370	X66.0393	Y20.5573
N9372	X66.0373	Y20.6786
N9374	X66.0339	Y20.8009
N9376	X66.0291	Y20.9241
N9378	X66.0229	Y21.0481
N9380	X66.0152	Y21.1731
N9382	X66.0061	Y21.2990
N9384	X65.9954	Y21.4258
N9386	X65.9833	Y21.5535
N9388	X65.9697	Y21.6821

N9390 X65.9545 Y21.8116

N9392 X65.9379 Y21.9421

N9394 X65.9196 Y22.0735

N9396 X65.8998 Y22.2058

N9398 X65.8785 Y22.3391

N9400 X65.8555 Y22.4733

N9402 X65.8310 Y22.6084

N9404 X65.8049 Y22.7444

N9406 X65.7771 Y22.8814

N9408 X65.7477 Y23.0194

N9410 X65.7166 Y23.1582

N9412 X65.6839 Y23.2981

N9414 X65.6495 Y23.4388

N9416 X65.6134 Y23.5806

N9418 X65.5756 Y23.7233

N9420 X65.5361 Y23.8669

N9422 X65.4949 Y24.0115

N9424 X65.4519 Y24.1571

N9426 X65.4072 Y24.3036

N9428 X65.3607 Y24.4511

N9430 X65.3124 Y24.5995

N9432 X65.2623 Y24.7490

N9434 X65.2104 Y24.8994

N9436 X65.1567 Y25.0508

N9438 X65.1011 Y25.2031

N9440 X65.0437 Y25.3565

N9442 X64.9845 Y25.5108

N9444 X64.9233 Y25.6661

N9446 X64.8603 Y25.8225

N9448	X64.7954	Y25.9798
N9450	X64.7286	Y26.1381
N9452	X64.6598	Y26.2973
N9454	X64.5891	Y26.4576
N9456	X64.5165	Y26.6189
N9458	X64.4418	Y26.7812
N9460	X64.3653	Y26.9445
N9462	X64.2869	Y27.1087
N9464	X64.2066	Y27.2737
N9466	X64.1246	Y27.4395
N9468	X64.0408	Y27.6061
N9470	X63.9554	Y27.7734
N9472	X63.8684	Y27.9413
N9474	X63.7798	Y28.1099
N9476	X63.6896	Y28.2789
N9478	X63.5980	Y28.4485
N9480	X63.5050	Y28.6185
N9482	X63.4107	Y28.7889
N9484	X63.3150	Y28.9596
N9486	X63.2180	Y29.1306
N9488	X63.1199	Y29.3018
N9490	X63.0206	Y29.4732
N9492	X62.9202	Y29.6447
N9494	X62.8187	Y29.8163
N9496	X62.7162	Y29.9879
N9498	X62.6128	Y30.1594
N9500	X62.5085	Y30.3309
N9502	X62.4034	Y30.5022
N9504	X62.2974	Y30.6733

N9506	X62.1907	Y30.8442
N9508	X62.0833	Y31.0147
N9510	X61.9753	Y31.1850
N9512	X61.8667	Y31.3548
N9514	X61.7575	Y31.5241
N9516	X61.6479	Y31.6929
N9518	X61.5378	Y31.8612
N9520	X61.4273	Y32.0288
N9522	X61.3165	Y32.1958
N9524	X61.2054	Y32.3621
N9526	X61.0941	Y32.5275
N9528	X60.9827	Y32.6922
N9530	X60.8710	Y32.8559
N9532	X60.7594	Y33.0188
N9534	X60.6477	Y33.1806
N9536	X60.5360	Y33.3414
N9538	X60.4244	Y33.5011
N9540	X60.3129	Y33.6597
N9542	X60.2017	Y33.8170
N9544	X60.0906	Y33.9731
N9546	X59.9799	Y34.1279
N9548	X59.8695	Y34.2813
N9550	X59.7594	Y34.4334
N9552	X59.6499	Y34.5839
N9554	X59.5408	Y34.7330
N9556	X59.4322	Y34.8805
N9558	X59.3243	Y35.0263
N9560	X59.2170	Y35.1705
N9562	X59.1104	Y35.3130

N9564 X59.0045 Y35.4537

N9566 X58.8995 Y35.5925

N9568 X58.7953 Y35.7295

N9570 X58.6920 Y35.8645

N9572 X58.5897 Y35.9975

N9574 X58.4884 Y36.1285

N9576 X58.3881 Y36.2574

N9578 X58.2890 Y36.3842

N9580 X58.1910 Y36.5087

N9582 X58.0942 Y36.6310

N9584 X57.9987 Y36.7510

N9586 X57.9045 Y36.8686

N9588 X57.8117 Y36.9838

N9590 X57.7203 Y37.0965

N9592 X57.6304 Y37.2068

N9594 X57.5419 Y37.3145

N9596 X57.4549 Y37.4199

N9598 X57.3693 Y37.5228

N9600 X57.2852 Y37.6235

N9602 X57.2024 Y37.7218

N9604 X57.1211 Y37.8178

N9606 X57.0413 Y37.9116

N9608 X56.9628 Y38.0032

N9610 X56.8857 Y38.0927

N9612 X56.8100 Y38.1801

N9614 X56.7356 Y38.2655

N9616 X56.6627 Y38.3488

N9618 X56.5911 Y38.4301

N9620 X56.5208 Y38.5095

N9622 X56.4519 Y38.5870

N9624 X56.3843 Y38.6626

N9626 X56.3181 Y38.7364

N9628 X56.2532 Y38.8085

N9630 X56.1896 Y38.8788

N9632 X56.1272 Y38.9474

N9634 X56.0662 Y39.0144

N9636 X56.0065 Y39.0797

N9638 X55.9481 Y39.1435

N9640 X55.8909 Y39.2058

N9642 X55.8350 Y39.2665

N9644 X55.7803 Y39.3259

N9646 X55.7269 Y39.3838

N9648 X55.6747 Y39.4403

N9650 X55.6237 Y39.4955

N9652 X55.5740 Y39.5495

N9654 X55.5255 Y39.6022

N9656 X55.4782 Y39.6537

N9658 X55.4321 Y39.7040

N9660 X55.3871 Y39.7532

N9662 X55.3434 Y39.8013

N9664 X55.3008 Y39.8484

N9666 X55.2594 Y39.8945

N9668 X55.2191 Y39.9397

N9670 X55.1800 Y39.9839

N9672 X55.1420 Y40.0272

N9674 X55.1052 Y40.0697

N9676 X55.0695 Y40.1114

N9678 X55.0348 Y40.1524

N9680 X55.0013 Y40.1926

N9682 X54.9689 Y40.2322

N9684 X54.9376 Y40.2712

N9686 X54.9074 Y40.3095

N9688 X54.8782 Y40.3473

N9690 X54.8501 Y40.3846

N9692 X54.8231 Y40.4215

N9694 X54.7971 Y40.4579

N9696 X54.7721 Y40.4939

N9698 X54.7482 Y40.5296

N9700 X54.7253 Y40.5650

N9702 X54.7034 Y40.6001

N9704 X54.6825 Y40.6350

N9706 X54.6626 Y40.6697

N9708 X54.6437 Y40.7042

N9710 X54.6258 Y40.7387

N9712 X54.6089 Y40.7731

N9714 X54.5929 Y40.8075

N9716 X54.5779 Y40.8419

N9718 X54.5639 Y40.8764

N9720 X54.5507 Y40.9110

N9722 X54.5385 Y40.9457

N9724 X54.5273 Y40.9806

N9726 X54.5169 Y41.0158

N9728 X54.5074 Y41.0512

N9730 X54.4987 Y41.0869

N9732 X54.4907 Y41.1229

N9734 X54.4834 Y41.1593

N9736 X54.4768 Y41.1961

N9738	X54.4707	Y41.2333
N9740	X54.4653	Y41.2709
N9742	X54.4603	Y41.3091
N9744	X54.4557	
N9746	X54.4515	Y41.3870
N9748	X54.4477	Y41.4269
N9750	X54.4441	Y41.4674
N9752	X54.4408	Y41.5085
N9754	X54.4377	Y41.5504
N9756	X54.4348	Y41.5930
N9758	X54.4319	Y41.6363
N9760	X54.4290	Y41.6805
N9762	X54.4261	Y41.7255
N9764	X54.4232	Y41.7713
N9766	X54.4201	Y41.8181
N9768	X54.4169	Y41.8658
N9770	X54.4135	Y41.9144
N9772	X54.4097	Y41.9641
N9774	X54.4057	Y42.0148
N9776	X54.4013	Y42.0666
N9778	X54.3965	Y42.1195
N9780	X54.3912	Y42.1735
N9782	X54.3853	Y42.2287
N9784	X54.3789	Y42.2851
N9786	X54.3719	Y42.3427
N9788	X54.3642	Y42.4017
N9790	X54.3558	Y42.4619
N9792	X54.3466	Y42.5235
N9794	X54.3365	Y42.5864

N9796	X54.3256	Y42.6508
N9798	X54.3137	Y42.7166
N9800	X54.3009	Y42.7839
N9802	X54.2870	Y42.8527
N9804	X54.2721	Y42.9230
N9806	X54.2560	Y42.9949
N9808	X54.2387	Y43.0685
N9810	X54.2202	Y43.1437
N9812	X54.2004	Y43.2205
N9814	X54.1793	Y43.2991
N9816	X54.1568	Y43.3795
N9818	X54.1328	Y43.4616
N9820	X54.1074	Y43.5455
N9822	X54.0804	Y43.6313
N9824	X54.0518	Y43.7190
N9826	X54.0216	Y43.8086
N9828	X53.9896	Y43.9001
N9830	X53.9560	Y43.9936
N9832	X53.9205	Y44.0892
N9834	X53.8832	Y44.1868
N9836	X53.8440	Y44.2865
N9838	X53.8028	Y44.3883
N9840	X53.7597	Y44.4923
N9842	X53.7145	Y44.5984
N9844	X53.6672	Y44.7068
N9846	X53.6177	Y44.8175
N9848	X53.5661	Y44.9304
N9850	X53.5122	Y45.0457
N9852	X53.4560	Y45.1633

N9854	X53.3974	Y45.2833
N9856	X53.3364	Y45.4058
N9858	X53.2730	Y45.5307
N9860	X53.2071	Y45.6580
N9862	X53.1389	Y45.7876
N9864	X53.0682	Y45.9194
N9866	X52.9952	Y46.0533
N9868	X52.9199	Y46.1892
N9870	X52.8422	Y46.3271
N9872	X52.7624	Y46.4668
N9874	X52.6803	Y46.6081
N9876	X52.5961	Y46.7511
N9878	X52.5097	Y46.8957
N9880	X52.4212	Y47.0416
N9882	X52.3306	Y47.1889
N9884	X52.2379	Y47.3375
N9886	X52.1433	Y47.4871
N9888	X52.0466	Y47.6378
N9890	X51.9481	Y47.7895
N9892	X51.8476	Y47.9420
N9894	X51.7452	Y48.0952
N9896	X51.6410	Y48.2491
N9898	X51.5349	Y48.4035
N9900	X51.4271	Y48.5584
N9902	X51.3175	Y48.7137
N9904	X51.2062	Y48.8692
N9906	X51.0933	Y49.0248
N9908	X50.9787	Y49.1806
N9910	X50.8624	Y49.3363

N9912 X50.7446 Y49.4918

N9914 X50.6253 Y49.6472

N9916 X50.5044 Y49.8022

N9918 X50.3820 Y49.9568

N9920 X50.2582 Y50.1109

N9922 X50.1330 Y50.2643

N9924 X50.0064 Y50.4171

N9926 X49.8785 Y50.5690

N9928 X49.7492 Y50.7201

N9930 X49.6187 Y50.8701

N9932 X49.4869 Y51.0190

N9934 X49.3539 Y51.1667

N9936 X49.2197 Y51.3131

N9938 X49.0843 Y51.4582

N9940 X48.9479 Y51.6017

N9942 X48.8103 Y51.7436

N9944 X48.6718 Y51.8839

N9946 X48.5322 Y52.0223

N9948 X48.3916 Y52.1589

N9950 X48.2501 Y52.2935

N9952 X48.1076 Y52.4261

N9954 X47.9643 Y52.5564

N9956 X47.8201 Y52.6845

N9958 X47.6751 Y52.8102

N9960 X47.5294 Y52.9334

N9962 X47.3829 Y53.0541

N9964 X47.2356 Y53.1722

N9966 X47.0877 Y53.2874

N9968 X46.9391 Y53.3998

N9970	X46.7900	Y53.5093
N9972	X46.6402	Y53.6157
N9974	X46.4899	Y53.7189
N9976	X46.3391	Y53.8189
N9978	X46.1878	Y53.9156
N9980	X46.0360	Y54.0088
N9982	X45.8838	Y54.0985
N9984	X45.7313	Y54.1845
N9986	X45.5784	Y54.2669
N9988	X45.4252	Y54.3453
N9990	X45.2717	Y54.4199
N9992	X45.1179	Y54.4905
N9994	X44.9639	Y54.5571
N9996	X44.8096	Y54.6198
N9998	X44.6551	Y54.6787
N10000	X44.5003	Y54.7338
N10002	X44.3452	Y54.7853
N10004	X44.1899	Y54.8331
N10006	X44.0343	Y54.8774
N10008	X43.8784	Y54.9182
N10010	X43.7222	Y54.9556
N10012	X43.5657	Y54.9896
N10014	X43.4089	Y55.0203
N10016	X43.2518	Y55.0478
N10018	X43.0944	Y55.0722
N10020	X42.9366	Y55.0934
N10022	X42.7786	Y55.1117
N10024	X42.6203	Y55.1270
N10026	X42.4616	Y55.1394

N10028 X42.3026 Y55.1489

N10030 X42.1432 Y55.1557

N10032 X41.9835 Y55.1599

N10034 X41.8235 Y55.1614

N10036 X41.6631 Y55.1603

N10038 X41.5024 Y55.1568

N10040 X41.3413 Y55.1508

N10042 X41.1799 Y55.1425

N10044 X41.0181 Y55.1319

N10046 X40.8559 Y55.1190

N10048 X40.6933 Y55.1040

N10050 X40.5304 Y55.0870

N10052 X40.3671 Y55.0679

N10054 X40.2034 Y55.0468

N10056 X40.0393 Y55.0238

N10058 X39.8748 Y54.9991

N10060 X39.7099 Y54.9725

N10062 X39.5446 Y54.9443

N10064 X39.3789 Y54.9145

N10066 X39.2128 Y54.8831

N10068 X39.0463 Y54.8502

N10070 X38.8793 Y54.8158

N10072 X38.7119 Y54.7802

N10074 X38.5441 Y54.7432

N10076 X38.3758 Y54.7050

N10078 X38.2071 Y54.6657

N10080 X38.0380 Y54.6252

N10082 X37.8684 Y54.5837

N10084 X37.6983 Y54.5413

N10086 X37.5278 Y54.4980

N10088 X37.3568 Y54.4539

N10090 X37.1854 Y54.4090

N10092 X37.0135 Y54.3634

N10094 X36.8411 Y54.3172

N10096 X36.6682 Y54.2704

N10098 X36.4949 Y54.2231

N10100 X36.3210 Y54.1754

N10102 X36.1467 Y54.1274

N10104 X35.9718 Y54.0790

N10106 X35.7965 Y54.0305

N10108 X35.6206 Y53.9817

N10110 X35.4443 Y53.9329

N10112 X35.2674 Y53.8841

N10114 X35.0900 Y53.8353

N10116 X34.9121 Y53.7866

N10118 X34.7336 Y53.7381

N10120 X34.5546 Y53.6898

N10122 X34.3751 Y53.6418

N10124 X34.1950 Y53.5942

N10126 X34.0144 Y53.5470

N10128 X33.8333 Y53.5002

N10130 X33.6519 Y53.4537

N10132 X33.4700 Y53.4075

N10134 X33.2878 Y53.3616

N10136 X33.1054 Y53.3160

N10138 X32.9228 Y53.2706

N10140 X32.7400 Y53.2253

N10142 X32.5572 Y53.1803

N10144 X32.3743 Y53.1353

N10146 X32.1914 Y53.0904

N10148 X32.0086 Y53.0456

N10150 X31.8259 Y53.0008

N10152 X31.6435 Y52.9560

N10154 X31.4612 Y52.9111

N10156 X31.2792 Y52.8662

N10158 X31.0976 Y52.8212

N10160 X30.9164 Y52.7760

N10162 X30.7357 Y52.7307

N10164 X30.5554 Y52.6851

N10166 X30.3757 Y52.6394

N10168 X30.1967 Y52.5933

N10170 X30.0183 Y52.5470

N10172 X29.8406 Y52.5003

N10174 X29.6638 Y52.4533

N10176 Y52.4059

N10178 X29.3126 Y52.3581

N10180 X29.1384 Y52.3098

N10182 X28.9652 Y52.2610

N10184 X28.7931 Y52.2117

N10186 X28.6221 Y52.1619

N10188 X28.4523 Y52.1114

N10190 X28.2836 Y52.0604

N10192 X28.1163 Y52.0087

N10194 X27.9503 Y51.9563

N10196 X27.7856 Y51.9032

N10198 X27.6224 Y51.8494

N10200 X27.4607 Y51.7948

N10202 X27.3006 Y51.7394

N10204 X27.1421 Y51.6831

N10206 X26.9852 Y51.6260

N10208 X26.8300 Y51.5679

N10210 X26.6766 Y51.5090

N10212 X26.5250 Y51.4490

N10214 X26.3753 Y51.3881

N10216 X26.2276 Y51.3261

N10218 X26.0818 Y51.2631

N10220 X25.9381 Y51.1989

N10222 X25.7964 Y51.1337

N10224 X25.6569 Y51.0672

N10226 X25.5196 Y50.9996

N10228 X25.3846 Y50.9308

N10230 X25.2519 Y50.8607

N10232 X25.1216 Y50.7893

N10234 X24.9937 Y50.7166

N10236 X24.8682 Y50.6425

N10238 X24.7453 Y50.5671

N10240 X24.6250 Y50.4902

N10242 X24.5074 Y50.4119

N10244 X24.3924 Y50.3321

N10246 X24.2802 Y50.2509

N10248 X24.1708 Y50.1680

N10250 X24.0643 Y50.0836

N10252 X23.9606 Y49.9976

N10254 X23.8600 Y49.9099

N10256 X23.7624 Y49.8206

N10258 X23.6678 Y49.7295

N10260 X23.5763 Y49.6368

N10262 X23.4879 Y49.5424

N10264 X23.4024 Y49.4464

N10266 X23.3199 Y49.3487

N10268 X23.2403 Y49.2496

N10270 X23.1636 Y49.1489

N10272 X23.0897 Y49.0467

N10274 X23.0185 Y48.9430

N10276 X22.9501 Y48.8379

N10278 X22.8844 Y48.7314

N10280 X22.8214 Y48.6236

N10282 X22.7609 Y48.5144

N10284 X22.7030 Y48.4039

N10286 X22.6477 Y48.2922

N10288 X22.5948 Y48.1792

N10290 X22.5444 Y48.0651

N10292 X22.4964 Y47.9498

N10294 X22.4507 Y47.8333

N10296 X22.4073 Y47.7158

N10298 X22.3663 Y47.5972

N10300 X22.3274 Y47.4775

N10302 X22.2908 Y47.3569

N10304 X22.2562 Y47.2353

N10306 X22.2238 Y47.1128

N10308 X22.1935 Y46.9893

N10310 X22.1652 Y46.8651

N10312 X22.1388 Y46.7399

N10314 X22.1144 Y46.6140

N10316 X22.0919 Y46.4874

N10318 X22.0712 Y46.3600

N10320 X22.0524 Y46.2319

N10322 X22.0353 Y46.1031

N10324 X22.0200 Y45.9737

N10326 X22.0063 Y45.8437

N10328 X21.9943 Y45.7131

N10330 X21.9838 Y45.5821

N10332 X21.9749 Y45.4505

N10334 X21.9676 Y45.3184

N10336 X21.9617 Y45.1859

N10338 X21.9572 Y45.0531

N10340 X21.9542 Y44.9198

N10342 X21.9525 Y44.7862

N10344 X21.9521 Y44.6524

N10346 X21.9529 Y44.5182

N10348 X21.9550 Y44.3839

N10350 X21.9582 Y44.2493

N10352 X21.9626 Y44.1146

N10354 X21.9681 Y43.9797

N10356 X21.9746 Y43.8448

N10358 X21.9822 Y43.7097

N10360 X21.9907 Y43.5747

N10362 X22.0001 Y43.4396

N10364 X22.0104 Y43.3046

N10366 X22.0216 Y43.1696

N10368 X22.0336 Y43.0348

N10370 X22.0463 Y42.9001

N10372 X22.0597 Y42.7655

N10374 X22.0738 Y42.6312

N10376 X22.0886 Y42.4971

N10378 X22.1039 Y42.3632

N10380 X22.1198 Y42.2297

N10382 X22.1361 Y42.0964

N10384 X22.1530 Y41.9636

N10386 X22.1702 Y41.8311

N10388 X22.1879 Y41.6991

N10390 X22.2059 Y41.5676

N10392 X22.2241 Y41.4365

N10394 X22.2427 Y41.3060

N10396 X22.2616 Y41.1759

N10398 X22.2808 Y41.0464

N10400 X22.3003 Y40.9173

N10402 X22.3201 Y40.7888

N10404 X22.3402 Y40.6607

N10406 X22.3607 Y40.5331

N10408 X22.3815 Y40.4061

N10410 X22.4026 Y40.2795

N10412 X22.4241 Y40.1534

N10414 X22.4459 Y40.0279

N10416 X22.4681 Y39.9028

N10418 X22.4906 Y39.7782

N10420 X22.5135 Y39.6541

N10422 X22.5368 Y39.5305

N10424 X22.5604 Y39.4074

N10426 X22.5844 Y39.2848

N10428 X22.6088 Y39.1626

N10430 X22.6336 Y39.0410

N10432 X22.6588 Y38.9198

N10434 X22.6844 Y38.7992

N10436 X22.7104 Y38.6790

N10438 X22.7368 Y38.5593

N10440 X22.7636 Y38.4401

N10442 X22.7908 Y38.3214

N10444 X22.8184 Y38.2032

N10446 X22.8465 Y38.0854

N10448 X22.8750 Y37.9682

N10450 X22.9040 Y37.8514

N10452 X22.9334 Y37.7351

N10454 X22.9632 Y37.6193

N10456 X22.9935 Y37.5040

N10458 X23.0242 Y37.3891

N10460 X23.0555 Y37.2747

N10462 X23.0872 Y37.1609

N10464 X23.1193 Y37.0474

N10466 X23.1520 Y36.9345

N10468 X23.1851 Y36.8221

N10470 X23.2187 Y36.7101

N10472 X23.2528 Y36.5986

N10474 X23.2874 Y36.4876

N10476 X23.3225 Y36.3771

N10478 X23.3581 Y36.2670

N10480 X23.3943 Y36.1574

N10482 X23.4309 Y36.0483

N10484 X23.4681 Y35.9396

N10486 X23.5058 Y35.8315

N10488 X23.5441 Y35.7238

N10490 X23.5829 Y35.6165

N10492 X23.6222 Y35.5098

N10494 X23.6621 Y35.4035

N10496 X23.7025 Y35.2977

N10498 X23.7435 Y35.1923

N10500 X23.7850 Y35.0875

N10502 X23.8272 Y34.9831

N10504 X23.8699 Y34.8791

N10506 X23.9132 Y34.7756

N10508 X23.9570 Y34.6726

N10510 X24.0015 Y34.5701

N10512 X24.0466 Y34.4680

N10514 X24.0922 Y34.3664

N10516 X24.1385 Y34.2653

N10518 X24.1854 Y34.1646

N10520 X24.2328 Y34.0643

N10522 X24.2810 Y33.9646

N10524 X24.3297 Y33.8653

N10526 X24.3791 Y33.7665

N10528 X24.4291 Y33.6681

N10530 X24.4797 Y33.5702

N10532 X24.5309 Y33.4729

N10534 X24.5828 Y33.3760

N10536 X24.6353 Y33.2797

N10538 X24.6885 Y33.1839

N10540 X24.7422 Y33.0887

N10542 X24.7967 Y32.9940

N10544 X24.8517 Y32.8999

N10546 X24.9073 Y32.8064

N10548 X24.9636 Y32.7135

N10550 X25.0205 Y32.6212

N10552 X25.0781 Y32.5296

N10554 X25.1363 Y32.4386

N10556 X25.1951 Y32.3482

N10558 X25.2545 Y32.2585

N10560 X25.3146 Y32.1695

N10562 X25.3753 Y32.0812

N10564 X25.4366 Y31.9935

N10566 X25.4985 Y31.9066

N10568 X25.5611 Y31.8205

N10570 X25.6243 Y31.7350

N10572 X25.6882 Y31.6503

N10574 X25.7526 Y31.5664

N10576 X25.8177 Y31.4833

N10578 X25.8834 Y31.4009

N10580 X25.9498 Y31.3194

N10582 X26.0168 Y31.2386

N10584 X26.0844 Y31.1587

N10586 X26.1526 Y31.0797

N10588 X26.2215 Y31.0015

N10590 X26.2910 Y30.9241

N10592 X26.3611 Y30.8477

N10594 X26.4319 Y30.7721

N10596 X26.5032 Y30.6975

N10598 X26.5752 Y30.6237

N10600 X26.6479 Y30.5509

N10602 X26.7212 Y30.4790

N10604 X26.7950 Y30.4081

N10606 X26.8696 Y30.3382

N10608 X26.9447 Y30.2692

N10610 X27.0205 Y30.2012

N10612 X27.0969 Y30.1342

N10614 X27.1739 Y30.0683

N10616 X27.2516 Y30.0034

N10618 X27.3299 Y29.9395

N10620 X27.4088 Y29.8767

N10622 X27.4883 Y29.8149

N10624 X27.5685 Y29.7542

N10626 X27.6493 Y29.6946

N10628 X27.7308 Y29.6361

N10630 X27.8128 Y29.5788

N10632 X27.8955 Y29.5226

N10634 X27.9788 Y29.4675

N10636 X28.0627 Y29.4135

N10638 X28.1473 Y29.3608

N10640 X28.2325 Y29.3092

N10642 X28.3183 Y29.2588

N10644 X28.4048 Y29.2096

N10646 X28.4919 Y29.1617

N10648 X28.5796 Y29.1149

N10650 X28.6679 Y29.0694

N10652 X28.7569 Y29.0252

N10654 X28.8465 Y28.9822

N10656 X28.9367 Y28.9406

N10658 X29.0275 Y28.9002

N10660 X29.1190 Y28.8611

N10662 X29.2110 Y28.8232

N10664 X29.3035 Y28.7864

N10666 X29.3965 Y28.7508

N10668 X29.4899 Y28.7162

N10670 X29.5837 Y28.6827

N10672 X29.6778 Y28.6500

N10674 X29.7722 Y28.6183

N10676 X29.8669 Y28.5875

N10678 X29.9618 Y28.5574

N10680 X30.0568 Y28.5281

N10682 X30.1520 Y28.4994

N10684 X30.2472 Y28.4714

N10686 X30.3425 Y28.4440

N10688 X30.4378 Y28.4171

N10690 X30.5330 Y28.3907

N10692 X30.6281 Y28.3647

N10694 X30.7230 Y28.3390

N10696 X30.8178 Y28.3137

N10698 X30.9123 Y28.2887

N10700 X31.0066 Y28.2639

N10702 X31.1005 Y28.2392

N10704 X31.1941 Y28.2147

N10706 X31.2872 Y28.1902

N10708 X31.3800 Y28.1657

N10710 X31.4722 Y28.1411

N10712 X31.5638 Y28.1165

N10714 X31.6549 Y28.0916

N10716 X31.7454 Y28.0666

N10718 X31.8352 Y28.0413

N10720 X31.9243 Y28.0158

N10722 X32.0126 Y27.9898

N10724 X32.1001 Y27.9634

N10726 X32.1867 Y27.9365

N10728 X32.2725 Y27.9092

N10730 X32.3574 Y27.8812

N10732 X32.4412 Y27.8526

N10734 X32.5240 Y27.8233

N10736 X32.6058 Y27.7933

N10738 X32.6865 Y27.7625

N10740 X32.7660 Y27.7309

N10742 X32.8443 Y27.6983

N10744 X32.9213 Y27.6648

N10746 X32.9971 Y27.6303

N10748 X33.0715 Y27.5948

N10750 X33.1446 Y27.5581

N10752 X33.2162 Y27.5203

N10754 X33.2864 Y27.4813

N10756 X33.3551 Y27.4410

N10758 X33.4222 Y27.3994

N10760 X33.4877 Y27.3564

N10762 X33.5516 Y27.3120

N10764 X33.6138 Y27.2661

N10766 X33.6743 Y27.2187

N10768 X33.7330 Y27.1697

N10770 X33.7899 Y27.1191

N10772 Y27.0668

N10774 X33.8981 Y27.0127

N10776 X33.9493 Y26.9569

N10778 X33.9985 Y26.8992

N10780 X34.0456 Y26.8396

N10782 X34.0907 Y26.7780

N10784 X34.1337 Y26.7145

N10786 X34.1745 Y26.6489

N10788 X34.2131 Y26.5812

N10790 X34.2494 Y26.5113

N10792 X34.2835 Y26.4392

N10794 X34.3152 Y26.3650

N10796 X34.3447 Y26.2887

N10798 X34.3720 Y26.2103

N10800 X34.3970 Y26.1301

N10802 X34.4198 Y26.0480

N10804 X34.4405 Y25.9642

N10806 X34.4590 Y25.8786

N10808 X34.4753 Y25.7915

N10810 X34.4896 Y25.7028

N10812 X34.5017 Y25.6126

N10814 X34.5118 Y25.5211

N10816 X34.5198 Y25.4282

N10818 X34.5257 Y25.3341

N10820 X34.5297 Y25.2389

N10822 X34.5316 Y25.1425

N10824 X34.5316 Y25.0452

N10826 X34.5297 Y24.9469

N10828 X34.5258 Y24.8478

N10830 X34.5199 Y24.7479

N10832 X34.5122 Y24.6474

N10834 X34.5026 Y24.5462

N10836 X34.4912 Y24.4444

N10838 X34.4780 Y24.3422

N10840 X34.4629 Y24.2396

N10842 X34.4460 Y24.1367

N10844 X34.4274 Y24.0335

N10846 X34.4070 Y23.9302

N10848 X34.3849 Y23.8268

N10850 X34.3611 Y23.7234

N10852 X34.3357 Y23.6201

N10854 X34.3085 Y23.5169

N10856 X34.2797 Y23.4140

N10858 X34.2493 Y23.3113

N10860 X34.2173 Y23.2090

N10862 X34.1837 Y23.1072

N10864 X34.1485 Y23.0059

N10866 X34.1118 Y22.9052

N10868 X34.0736 Y22.8052

N10870 X34.0339 Y22.7059

N10872 X33.9927 Y22.6075

N10874 X33.9500 Y22.5100

N10876 X33.9059 Y22.4135

N10878 X33.8604 Y22.3181

N10880 X33.8135 Y22.2238

N10882 X33.7653 Y22.1307

N10884 X33.7156 Y22.0389

N10886 X33.6647 Y21.9485

N10888 X33.6124 Y21.8595

N10890 X33.5588 Y21.7721

N10892 X33.5040 Y21.6863

N10894 X33.4479 Y21.6021

N10896 X33.3906 Y21.5197

N10898 X33.3320 Y21.4392

N10900 X33.2723 Y21.3605

N10902 X33.2114 Y21.2839

N10904 X33.1494 Y21.2093

N10906 X33.0862 Y21.1368

N10908 X33.0220 Y21.0666

N10910 X32.9566 Y20.9986

N10912 X32.8902 Y20.9330

N10914 X32.8227 Y20.8699

N10916 X32.7543 Y20.8093

N10918 X32.6848 Y20.7513

N10920 X32.6143 Y20.6960

N10922 X32.5429 Y20.6434

N10924 X32.4705 Y20.5936

N10926 X32.3972 Y20.5468

N10928 X32.3230 Y20.5027

N10930 X32.2479 Y20.4615

N10932 X32.1720 Y20.4231

N10934 X32.0952 Y20.3873

N10936 X32.0177 Y20.3543

N10938 X31.9393 Y20.3238

N10940 X31.8601 Y20.2960

N10942 X31.7802 Y20.2708

N10944 X31.6995 Y20.2480

N10946 X31.6181 Y20.2278

N10948 X31.5360 Y20.2100

N10950 X31.4533 Y20.1946

N10952 X31.3699 Y20.1815

N10954 X31.2858 Y20.1708

N10956 X31.2011 Y20.1623

N10958 X31.1159 Y20.1561

N10960 X31.0300 Y20.1521

N10962 X30.9436 Y20.1503

N10964 X30.8567 Y20.1505

N10966 X30.7692 Y20.1529

N10968 X30.6812 Y20.1572

N10970 X30.5928 Y20.1636

N10972 X30.5039 Y20.1720

N10974 X30.4146 Y20.1822

N10976 X30.3249 Y20.1943

N10978 X30.2347 Y20.2083

N10980 X30.1442 Y20.2241

N10982 X30.0533 Y20.2416

N10984 X29.9621 Y20.2608

N10986 X29.8706 Y20.2818

N10988 X29.7788 Y20.3043

N10990 X29.6867 Y20.3285

N10992 X29.5944 Y20.3542

N10994 X29.5018 Y20.3814

N10996 X29.4090 Y20.4101

N10998 X29.3160 Y20.4402

N11000 X29.2228 Y20.4717

N11002 X29.1295 Y20.5046

N11004 X29.0361 Y20.5388

N11006 X28.9425 Y20.5742

N11008 X28.8489 Y20.6109

N11010 X28.7551 Y20.6488

N11012 X28.6614 Y20.6878

N11014 X28.5675	Y20.7280
N11016 X28.4737	Y20.7692
N11018 X28.3799	Y20.8114
N11020 X28.2861	Y20.8547
N11022 X28.1923	Y20.8989
N11024 X28.0987	Y20.9440
N11026 X28.0051	Y20.9900
N11028 X27.9116	Y21.0368
N11030 X27.8182	Y21.0844
N11032 X27.7250	Y21.1327
N11034 X27.6320	Y21.1818
N11036 X27.5392	Y21.2315
N11038 X27.4465	Y21.2818
N11040 X27.3541	Y21.3328
N11042 X27.2619	Y21.3843
N11044 X27.1701	Y21.4363
N11046 X27.0785	Y21.4888
N11048 X26.9872	Y21.5417
N11050 X26.8962	Y21.5949
N11052 X26.8056	
N11054 X26.7154	
N11056 X26.6255	Y21.7567
N11058 X26.5361	Y21.8111
N11060 X26.4470	Y21.8658
N11062 X26.3584	Y21.9208
N11064 X26.2701	Y21.9761
N11066 X26.1822	Y22.0319
N11068 X26.0946	Y22.0881
N11070 X26.0072	Y22.1448

N11072 X25.9202 Y22.2022

N11074 X25.8334 Y22.2602

N11076 X25.7469 Y22.3189

N11078 X25.6605 Y22.3784

N11080 X25.5744 Y22.4388

N11082 X25.4884 Y22.5000

N11084 X25.4026 Y22.5622

N11086 X25.3168 Y22.6254

N11088 X25.2312 Y22.6896

N11090 X25.1457 Y22.7550

N11092 X25.0602 Y22.8216

N11094 X24.9748 Y22.8895

N11096 X24.8894 Y22.9587

N11098 X24.8039 Y23.0292

N11100 X24.7185 Y23.1012

N11102 X24.6330 Y23.1746

N11104 X24.5474 Y23.2496

N11106 X24.4617 Y23.3262

N11108 X24.3759 Y23.4045

N11110 X24.2899 Y23.4846

N11112 X24.2038 Y23.5664

N11114 X24.1175 Y23.6501

N11116 X24.0310 Y23.7356

N11118 X23.9443 Y23.8232

N11120 X23.8573 Y23.9127

N11122 X23.7701 Y24.0044

N11124 X23.6825 Y24.0982

N11126 X23.5947 Y24.1943

N11128 X23.5065 Y24.2925

N11130 X23.4179 Y24.3932

N11132 X23.3290 Y24.4962

N11134 X23.2396 Y24.6016

N11136 X23.1499 Y24.7096

N11138 X23.0596 Y24.8201

N11140 X22.9689 Y24.9333

N11142 X22.8778 Y25.0491

N11144 X22.7861 Y25.1677

N11146 X22.6938 Y25.2891

N11148 X22.6011 Y25.4134

N11150 X22.5077 Y25.5406

N11152 X22.4137 Y25.6708

N11154 X22.3191 Y25.8040

N11156 X22.2239 Y25.9403

N11158 X22.1280 Y26.0798

N11160 X22.0314 Y26.2225

N11162 X21.9341 Y26.3685

N11164 X21.8360 Y26.5179

N11166 X21.7372 Y26.6706

N11168 X21.6376 Y26.8268

N11170 X21.5373 Y26.9866

N11172 X21.4361 Y27.1499

N11174 X21.3340 Y27.3169

N11176 X21.2311 Y27.4875

N11178 X21.1273 Y27.6619

N11180 X21.0226 Y27.8402

N11182 X20.9170 Y28.0223

N11184 X20.8104 Y28.2084

N11186 X20.7028 Y28.3985

N11188 X20.5942 Y28.5926

N11190 X20.4847 Y28.7909

N11192 X20.3740 Y28.9933

N11194 X20.2624 Y29.1998

N11196 X20.1498 Y29.4103

N11198 X20.0363 Y29.6247

N11200 X19.9219 Y29.8429

N11202 X19.8067 Y30.0647

N11204 X19.6908 Y30.2900

N11206 X19.5740 Y30.5188

N11208 X19.4566 Y30.7510

N11210 X19.3386 Y30.9863

N11212 X19.2200 Y31.2248

N11214 X19.1008 Y31.4662

N11216 X18.9811 Y31.7105

N11218 X18.8609 Y31.9576

N11220 X18.7404 Y32.2074

N11222 X18.6194 Y32.4597

N11224 X18.4982 Y32.7145

N11226 X18.3766 Y32.9715

N11228 X18.2549 Y33.2308

N11230 X18.1329 Y33.4923

N11232 X18.0109 Y33.7557

N11234 X17.8887 Y34.0210

N11236 X17.7665 Y34.2881

N11238 X17.6443 Y34.5568

N11240 X17.5221 Y34.8271

N11242 X17.4000 Y35.0989

N11244 X17.2781 Y35.3720

N11246 X17.1563 Y35.6463

N11248 X17.0348 Y35.9217

N11250 X16.9135 Y36.1981

N11252 X16.7926 Y36.4755

N11254 X16.6720 Y36.7536

N11256 X16.5518 Y37.0323

N11258 X16.4321 Y37.3117

N11260 X16.3129 Y37.5915

N11262 X16.1942 Y37.8716

N11264 X16.0762 Y38.1520

N11266 X15.9588 Y38.4325

N11268 X15.8420 Y38.7129

N11270 X15.7260 Y38.9933

N11272 X15.6108 Y39.2735

N11274 X15.4964 Y39.5534

N11276 X15.3829 Y39.8328

N11278 X15.2703 Y40.1116

N11280 X15.1586 Y40.3899

N11282 X15.0480 Y40.6673

N11284 X14.9384 Y40.9439

N11286 X14.8299 Y41.2195

N11288 X14.7225 Y41.4940

N11290 X14.6163 Y41.7673

N11292 X14.5114 Y42.0392

N11294 X14.4078 Y42.3098

N11296 X14.3055 Y42.5788

N11298 X14.2045 Y42.8461

N11300 X14.1050 Y43.1117

N11302 X14.0069 Y43.3754

N11304 X13.9104 Y43.6371

N11306 X13.8154 Y43.8968

N11308 X13.7220 Y44.1542

N11310 X13.6302 Y44.4093

N11312 X13.5402 Y44.6620

N11314 X13.4519 Y44.9122

N11316 X13.3653 Y45.1597

N11318 X13.2806 Y45.4045

N11320 X13.1978 Y45.6464

N11322 X13.1169 Y45.8853

N11324 X13.0380 Y46.1211

N11326 X12.9611 Y46.3538

N11328 X12.8862 Y46.5833

N11330 X12.8135 Y46.8098

N11332 X12.7429 Y47.0333

N11334 X12.6745 Y47.2539

N11336 X12.6084 Y47.4715

N11338 X12.5446 Y47.6864

N11340 X12.4832 Y47.8985

N11342 X12.4242 Y48.1078

N11344 X12.3677 Y48.3145

N11346 X12.3136 Y48.5187

N11348 X12.2621 Y48.7203

N11350 X12.2133 Y48.9194

N11352 X12.1671 Y49.1161

N11354 X12.1236 Y49.3104

N11356 X12.0828 Y49.5025

N11358 X12.0449 Y49.6923

N11360 X12.0099 Y49.8799

N11362 X11.9777 Y50.0654

N11364 X11.9485 Y50.2488

N11366 X11.9223 Y50.4302

N11368 X11.8992 Y50.6097

N11370 X11.8792 Y50.7873

N11372 X11.8623 Y50.9630

N11374 X11.8486 Y51.1370

N11376 X11.8382 Y51.3092

N11378 X11.8311 Y51.4798

N11380 X11.8274 Y51.6488

N11382 X11.8271 Y51.8163

N11384 X11.8302 Y51.9823

N11386 X11.8368 Y52.1468

N11388 X11.8470 Y52.3100

N11390 X11.8608 Y52.4719

N11392 X11.8782 Y52.6326

N11394 X11.8994 Y52.7920

N11396 X11.9243 Y52.9503

N11398 X11.9530 Y53.1076

N11400 X11.9855 Y53.2638

N11402 X12.0220 Y53.4191

N11404 X12.0624 Y53.5735

N11406 X12.1068 Y53.7270

N11408 X12.1553 Y53.8798

N11410 X12.2079 Y54.0318

N11412 X12.2646 Y54.1832

N11414 X12.3255 Y54.3339

N11416 X12.3907 Y54.4842

N11418 X12.4602 Y54.6339

N11420 X12.5340 Y54.7832

N11422 X12.6122 Y54.9321

N11424 X12.6949 Y55.0807

N11426 X12.7820 Y55.2291

N11428 X12.8738 Y55.3773

N11430 X12.9701 Y55.5253

N11432 X13.0711 Y55.6733

N11434 X13.1767 Y55.8212

N11436 X13.2871 Y55.9692

N11438 X13.4024 Y56.1172

N11440 X13.5224 Y56.2654

N11442 X13.6474 Y56.4139

N11444 X13.7773 Y56.5626

N11446 X13.9122 Y56.7116

N11448 X14.0521 Y56.8610

N11450 X14.1972 Y57.0109

N11452 X14.3474 Y57.1613

N11454 X14.5027 Y57.3122

N11456 X14.6634 Y57.4637

N11458 X14.8293 Y57.6160

N11460 X15.0005 Y57.7689

N11462 X15.1767 Y57.9226

N11464 X15.3579 Y58.0769

N11466 X15.5438 Y58.2319

N11468 X15.7344 Y58.3876

N11470 X15.9294 Y58.5439

N11472 X16.1287 Y58.7008

N11474 X16.3321 Y58.8584

N11476 X16.5395 Y59.0167

N11478 X16.7507 Y59.1755

N11480 X16.9655 Y59.3349

N11482 X17.1838 Y59.4950

N11484 X17.4054 Y59.6556

N11486 X17.6302 Y59.8167

N11488 X17.8580 Y59.9785

N11490 X18.0886 Y60.1407

N11492 X18.3219 Y60.3036

N11494 X18.5578 Y60.4669

N11496 X18.7959 Y60.6308

N11498 X19.0363 Y60.7951

N11500 X19.2787 Y60.9600

N11502 X19.5230 Y61.1253

N11504 X19.7689 Y61.2911

N11506 X20.0165 Y61.4574

N11508 X20.2654 Y61.6241

N11510 X20.5156 Y61.7913

N11512 X20.7668 Y61.9589

N11514 X21.0189 Y62.1269

N11516 X21.2718 Y62.2954

N11518 X21.5253 Y62.4642

N11520 X21.7791 Y62.6334

N11522 X22.0333 Y62.8030

N11524 X22.2876 Y62.9729

N11526 X22.5418 Y63.1433

N11528 X22.7957 Y63.3139

N11530 X23.0493 Y63.4849

N11532 X23.3024 Y63.6562

N11534 X23.5548 Y63.8278

N11536 X23.8063 Y63.9998

N11538 X24.0567 Y64.1720

N11540 Y64.3445

N11542 X24.5540 Y64.5173

N11544 X24.8004 Y64.6903

N11546 X25.0452 Y64.8636

N11548 X25.2881 Y65.0371

N11550 X25.5291 Y65.2108

N11552 X25.7679 Y65.3848

N11554 X26.0044 Y65.5589

N11556 X26.2384 Y65.7333

N11558 X26.4698 Y65.9078

N11560 X26.6984 Y66.0825

N11562 X26.9241 Y66.2574

N11564 X27.1466 Y66.4325

N11566 X27.3659 Y66.6076

N11568 X27.5817 Y66.7829

N11570 X27.7940 Y66.9584

N11572 X28.0024 Y67.1339

N11574 X28.2070 Y67.3095

N11576 X28.4075 Y67.4852

N11578 X28.6037 Y67.6610

N11580 X28.7956 Y67.8369

N11582 X28.9828 Y68.0128

N11584 X29.1654 Y68.1888

N11586 X29.3431 Y68.3648

N11588 X29.5157 Y68.5408

N11590 X29.6831 Y68.7169

N11592 X29.8452 Y68.8929

N11594 X30.0019 Y69.0689

N11596 X30.1534 Y69.2450

N11598 X30.2996 Y69.4210

N11600 X30.4408 Y69.5971

N11602 X30.5769 Y69.7731

N11604 X30.7081 Y69.9492

N11606 X30.8344 Y70.1252

N11608 X30.9558 Y70.3013

N11610 X31.0726 Y70.4774

N11612 X31.1847 Y70.6534

N11614 X31.2923 Y70.8295

N11616 X31.3953 Y71.0056

N11618 X31.4940 Y71.1817

N11620 X31.5883 Y71.3578

N11622 X31.6783 Y71.5339

N11624 X31.7642 Y71.7100

N11626 X31.8460 Y71.8862

N11628 X31.9237 Y72.0623

N11630 X31.9976 Y72.2385

N11632 X32.0675 Y72.4146

N11634 X32.1337 Y72.5908

N11636 X32.1961 Y72.7670

N11638 X32.2549 Y72.9432

N11640 X32.3102 Y73.1194

N11642 X32.3620 Y73.2957

N11644 X32.4104 Y73.4719

N11646 X32.4554 Y73.6482

N11648 X32.4972 Y73.8245

N11650 X32.5359 Y74.0008

N11652 X32.5715 Y74.1771

N11654 X32.6040 Y74.3534

N11656 X32.6337 Y74.5298

N11658 X32.6604 Y74.7061

N11660 X32.6844 Y74.8825

N11662 X32.7057 Y75.0590

N11664 X32.7244 Y75.2354

N11666 X32.7406 Y75.4119

N11668 X32.7543 Y75.5884

N11670 X32.7656 Y75.7649

N11672 X32.7745 Y75.9414

N11674 X32.7813 Y76.1180

N11676 X32.7859 Y76.2945

N11678 X32.7884 Y76.4711

N11680 X32.7890 Y76.6478

N11682 X32.7876 Y76.8244

N11684 X32.7844 Y77.0011

N11686 X32.7794 Y77.1779

N11688 X32.7727 Y77.3546

N11690 X32.7644 Y77.5314

N11692 X32.7546 Y77.7082

N11694 X32.7434 Y77.8850

N11696 X32.7307 Y78.0619

N11698 X32.7168 Y78.2388

N11700 X32.7017 Y78.4157

N11702 X32.6854 Y78.5927

N11704 X32.6681 Y78.7697

N11706 X32.6497 Y78.9467

N11708 X32.6305 Y79.1238

N11710 X32.6104 Y79.3009

N11712 X32.5896 Y79.4781

N11714 X32.5682 Y79.6552

N11716 X32.5461 Y79.8325

N11718 X32.5235 Y80.0097

N11720 X32.5005 Y80.1870

N11722 X32.4771 Y80.3643

N11724 X32.4534 Y80.5417

N11726 X32.4295 Y80.7191

N11728 X32.4054 Y80.8966

N11730 X32.3811 Y81.0741

N11732 X32.3566 Y81.2517

N11734 X32.3319 Y81.4294

N11736 X32.3069 Y81.6071

N11738 X32.2817 Y81.7850

N11740 X32.2563 Y81.9629

N11742 X32.2306 Y82.1410

N11744 X32.2047 Y82.3192

N11746 X32.1786 Y82.4975

N11748 X32.1523 Y82.6759

N11750 X32.1257 Y82.8545

N11752 X32.0988 Y83.0333

N11754 X32.0718 Y83.2122

N11756 X32.0444 Y83.3913

N11758 X32.0168 Y83.5706

N11760 X31.9890 Y83.7500

N11762 X31.9609 Y83.9297

N11764 X31.9326 Y84.1096

N11766 X31.9040 Y84.2897

N11768 X31.8751 Y84.4700

N11770 X31.8460 Y84.6505

N11772 X31.8166 Y84.8313

N11774 X31.7869 Y85.0124

N11776 X31.7570 Y85.1937

N11778 X31.7268 Y85.3753

N11780 X31.6963 Y85.5571

N11782 X31.6655 Y85.7393

N11784 X31.6345 Y85.9217

N11786 X31.6031 Y86.1045

N11788 X31.5715 Y86.2876

N11790 X31.5396 Y86.4710

N11792 X31.5074 Y86.6547

N11794 X31.4748 Y86.8388

N11796 X31.4420 Y87.0232

N11798 X31.4089 Y87.2080

N11800 X31.3755 Y87.3931

N11802 X31.3418 Y87.5786

N11804 X31.3078 Y87.7645

N11806 X31.2735 Y87.9508

N11808 X31.2388 Y88.1375

N11810 X31.2039 Y88.3246

N11812 X31.1686 Y88.5122

N11814 X31.1330 Y88.7002

N11816 X31.0971 Y88.8886

N11818 X31.0608 Y89.0774

N11820 X31.0243 Y89.2667

N11822 X30.9873 Y89.4565

N11824 X30.9501 Y89.6467

N11826 X30.9125 Y89.8375

N11828 X30.8746 Y90.0287

N11830 X30.8364 Y90.2204

N11832 X30.7978 Y90.4127

N11834 X30.7588 Y90.6054

N11836 X30.7195 Y90.7987

N11838 X30.6799 Y90.9925

N11840 X30.6399 Y91.1869

N11842 X30.5996 Y91.3818

N11844 X30.5588 Y91.5772

N11846 X30.5178 Y91.7733

N11848 X30.4763 Y91.9699

N11850 X30.4345 Y92.1671

N11852 X30.3924 Y92.3649

N11854 X30.3498 Y92.5633

N11856 X30.3069 Y92.7624

N11858 X30.2636 Y92.9620

N11860 X30.2200 Y93.1623

N11862 X30.1760 Y93.3631

N11864 X30.1316 Y93.5643

N11866 X30.0869 Y93.7660

N11868 X30.0419 Y93.9680

N11870 X29.9966 Y94.1702

N11872 X29.9510 Y94.3726

N11874 X29.9051 Y94.5751

N11876 X29.8589 Y94.7777

N11878 X29.8125 Y94.9802

N11880 X29.7659 Y95.1827

N11882 X29.7191 Y95.3850

N11884 X29.6720 Y95.5870

N11886 X29.6248 Y95.7888

N11888 X29.5774 Y95.9902

N11890 X29.5298 Y96.1911

N11892 X29.4820 Y96.3915

N11894 X29.4342 Y96.5913

N11896 X29.3862 Y96.7905

N11898 X29.3381 Y96.9890

N11900 X29.2899 Y97.1867

N11902 X29.2416 Y97.3835

N11904 X29.1932 Y97.5794

N11906 X29.1448 Y97.7742

N11908 X29.0964 Y97.9680

N11910 X29.0479 Y98.1607

N11912 X28.9994 Y98.3522

N11914 X28.9509 Y98.5423

N11916 X28.9025 Y98.7312

N11918 X28.8540 Y98.9186

N11920 X28.8056 Y99.1045

N11922 X28.7573 Y99.2888

N11924 X28.7090 Y99.4716

N11926 X28.6609 Y99.6526

N11928 X28.6128 Y99.8319

N11930 X28.5648 Y100.0093

N11932 X28.5170 Y100.1848

N11934 X28.4693 Y100.3583

N11936 X28.4217 Y100.5298

N11938 X28.3743 Y100.6992

N11940 X28.3271 Y100.8664

N11942 X28.2801 Y101.0313

N11944 X28.2333 Y101.1939

N11946 X28.1868 Y101.3541

N11948 X28.1404 Y101.5118

N11950 X28.0944 Y101.6671

N11952 X28.0485 Y101.8196

N11954 X28.0030 Y101.9696

N11956 X27.9578 Y102.1167

N11958 X27.9128 Y102.2611

N11960 X27.8682 Y102.4026

N11962 X27.8239 Y102.5411

N11964 X27.7800 Y102.6766

N11966 X27.7364 Y102.8090

N11968 X27.6932 Y102.9382

N11970 X27.6504 Y103.0643

N11972 X27.6080 Y103.1870

N11974 X27.5660 Y103.3063

N11976 X27.5244 Y103.4222

N11978 X27.4833 Y103.5346

N11980 X27.4427 Y103.6433

N11982 X27.4025 Y103.7485

N11984 X27.3628 Y103.8499

N11986 X27.3236 Y103.9475

N11988 X27.2849 Y104.0413

N11990 X27.2468 Y104.1311

N11992 X27.2092 Y104.2170

N11994 X27.1721 Y104.2988

N11996 X27.1356 Y104.3768

N11998 X27.0995 Y104.4509

N12000 X27.0640 Y104.5212

N12002 X27.0289 Y104.5878

N12004 X26.9943 Y104.6507

N12006 X26.9602 Y104.7101

N12008 X26.9265 Y104.7659

N12010 X26.8933 Y104.8182

N12012 X26.8604 Y104.8672

N12014 X26.8280 Y104.9128

N12016 X26.7960 Y104.9551

N12018 X26.7643 Y104.9942

N12020 X26.7330 Y105.0302

N12022 X26.7021 Y105.0631

N12024 X26.6715 Y105.0930

N12026 X26.6412 Y105.1199

N12028 X26.6112 Y105.1439

N12030 X26.5816 Y105.1652

N12032 X26.5522 Y105.1836

N12034 X26.5231 Y105.1994

N12036 X26.4942 Y105.2125

N12038 X26.4656 Y105.2230

N12040 X26.4372 Y105.2311

N12042 X26.4091 Y105.2367

N12044 X26.3811 Y105.2399

N12046 X26.3534 Y105.2409

N12048 X26.3258 Y105.2395

N12050 X26.2984 Y105.2360

N12052 X26.2711 Y105.2304

N12054 X26.2440 Y105.2227

N12056 X26.2170 Y105.2130

N12058 X26.1901 Y105.2014

N12060 X26.1634 Y105.1880

N12062 X26.1367 Y105.1727

N12064 X26.1101 Y105.1557

N12066 X26.0835 Y105.1370

N12068 X26.0570 Y105.1167

N12070 X26.0305 Y105.0949

N12072 X26.0041 Y105.0716

N12074 X25.9776 Y105.0469

N12076 X25.9512 Y105.0208

N12078 X25.9247 Y104.9935

N12080 X25.8982 Y104.9649

N12082 X25.8716 Y104.9352

N12084 X25.8450 Y104.9043

N12086 X25.8183 Y104.8725

N12088 X25.7916 Y104.8397

N12090 X25.7647 Y104.8059

N12092 X25.7377 Y104.7714

N12094 X25.7106 Y104.7360

N12096 X25.6834 Y104.7000

N12098 X25.6560 Y104.6633

N12100 X25.6284 Y104.6260

N12102 X25.6007 Y104.5882

N12104 X25.5728 Y104.5500

N12106 X25.5446 Y104.5113

N12108 X25.5163 Y104.4724

N12110 X25.4877 Y104.4332

N12112 X25.4589 Y104.3937

N12114 X25.4298 Y104.3542

N12116 X25.4004 Y104.3146

N12118 X25.3708 Y104.2749

N12120 X25.3408 Y104.2354

N12122 X25.3106 Y104.1959

N12124 X25.2800 Y104.1567

N12126 X25.2491 Y104.1177

N12128 X25.2179 Y104.0789

N12130 X25.1863 Y104.0403

N12132 X25.1545 Y104.0019

N12134 X25.1224 Y103.9637

N12136 X25.0899 Y103.9256

N12138 X25.0573 Y103.8876

N12140 X25.0243 Y103.8497

N12142 X24.9912 Y103.8119

N12144 X24.9578 Y103.7741

N12146 X24.9241 Y103.7364

N12148 X24.8903 Y103.6987

N12150 X24.8563 Y103.6609

N12152 X24.8221 Y103.6231

N12154 X24.7877 Y103.5852

N12156 X24.7532 Y103.5473

N12158 X24.7185 Y103.5092

N12160 X24.6837 Y103.4710

N12162 X24.6488 Y103.4327

N12164 X24.6138 Y103.3942

N12166 X24.5786 Y103.3554

N12168 X24.5434 Y103.3165

N12170 X24.5081 Y103.2773

N12172 X24.4728 Y103.2378

N12174 X24.4374 Y103.1981

N12176 X24.4020 Y103.1580

N12178 X24.3665 Y103.1176

N12180 X24.3310 Y103.0768

N12182 X24.2955 Y103.0357

N12184 X24.2601 Y102.9941

N12186 X24.2246 Y102.9521

N12188 X24.1892 Y102.9097

N12190 X24.1539 Y102.8668

N12192 X24.1186 Y102.8234

N12194 X24.0833 Y102.7795

N12196 X24.0482 Y102.7350

N12198 X24.0131 Y102.6900

N12200 X23.9782 Y102.6444

N12202 X23.9433 Y102.5982

N12204 X23.9086 Y102.5514

N12206 X23.8741 Y102.5039

N12208 X23.8397 Y102.4557

N12210 X23.8055 Y102.4068

N12212 X23.7714 Y102.3573

N12214 X23.7375 Y102.3069

N12216 X23.7039 Y102.2558

N12218 X23.6704 Y102.2039

N12220 X23.6372 Y102.1512

N12222 X23.6042 Y102.0977

N12224 X23.5715 Y102.0433

N12226 X23.5390 Y101.9881

N12228 X23.5068 Y101.9319

N12230 X23.4749 Y101.8748

N12232 X23.4433 Y101.8167

N12234 X23.4120 Y101.7577

N12236 X23.3811 Y101.6977

N12238 X23.3504 Y101.6367

N12240 X23.3201 Y101.5747

N12242 X23.2902 Y101.5115

N12244 X23.2606 Y101.4473

N12246 X23.2315 Y101.3820

N12248 X23.2027 Y101.3156

N12250 X23.1743 Y101.2480

N12252 X23.1464 Y101.1793

N12254 X23.1188 Y101.1093

N12256 X23.0917 Y101.0381

N12258 X23.0651 Y100.9657

N12260 X23.0389 Y100.8921

N12262 X23.0132 Y100.8172

N12264 X22.9880 Y100.7411

N12266 X22.9632 Y100.6639

N12268 X22.9388 Y100.5855

N12270 X22.9148 Y100.5060

N12272 X22.8913 Y100.4254

N12274 X22.8682 Y100.3438

N12276 X22.8456 Y100.2611

N12278 X22.8233 Y100.1774

N12280 X22.8015 Y100.0927

N12282 X22.7800 Y100.0071

N12284 X22.7589 Y99.9206

N12286 X22.7383 Y99.8332

N12288 X22.7180 Y99.7449

N12290 X22.6981 Y99.6557

N12292 X22.6786 Y99.5658

N12294 X22.6594 Y99.4750

N12296 X22.6406 Y99.3835

N12298 X22.6222 Y99.2913

N12300 X22.6041 Y99.1983

N12302 X22.5864 Y99.1047

N12304 X22.5690 Y99.0104

N12306 X22.5519 Y98.9155

N12308 X22.5352 Y98.8200

N12310 X22.5188 Y98.7239

N12312 X22.5027 Y98.6273

N12314 X22.4869 Y98.5301

N12316 X22.4714 Y98.4325

N12318 X22.4563 Y98.3344

N12320 X22.4414 Y98.2358

N12322 X22.4268 Y98.1369

N12324 X22.4126 Y98.0376

N12326 X22.3986 Y97.9379

N12328 X22.3848 Y97.8378

N12330 X22.3714 Y97.7375

N12332 X22.3582 Y97.6369

N12334 X22.3453 Y97.5361

N12336 X22.3326 Y97.4350

N12338 X22.3201 Y97.3337

N12340 X22.3080 Y97.2323

N12342 X22.2960 Y97.1308

N12344 X22.2843 Y97.0291

N12346 X22.2728 Y96.9273

N12348 X22.2615 Y96.8255

N12350 X22.2505 Y96.7236

N12352 X22.2396 Y96.6218

N12354 X22.2290 Y96.5199

N12356 X22.2186 Y96.4181

N12358 X22.2083 Y96.3164

N12360 X22.1983 Y96.2148

N12362 X22.1884 Y96.1133

N12364 X22.1787 Y96.0120

N12366 X22.1692 Y95.9109

N12368 X22.1598 Y95.8100

N12370 X22.1506 Y95.7093

N12372 X22.1416 Y95.6089

N12374 X22.1327 Y95.5087

N12376 X22.1239 Y95.4089

N12378 X22.1153 Y95.3095

N12380 X22.1068 Y95.2104

N12382 X22.0985 Y95.1117

N12384 X22.0903 Y95.0134

N12386 X22.0822 Y94.9156

N12388 X22.0742 Y94.8183

N12390 X22.0663 Y94.7215

N12392 X22.0585

N12394 X22.0508

N12396 X22.0432 Y94.4343

N12398 X22.0356 Y94.3397

N12400 X22.0281 Y94.2457

N12402 X22.0206 Y94.1523

N12404 X22.0131 Y94.0594

N12406 X22.0057 Y93.9672

N12408 X21.9982 Y93.8756

N12410 X21.9906 Y93.7845

N12412 X21.9831 Y93.6941

N12414 X21.9754 Y93.6044

N12416 X21.9677 Y93.5152

N12418 X21.9599 Y93.4268

N12420 X21.9520 Y93.3390

N12422 X21.9440 Y93.2518

N12424 X21.9358 Y93.1653

N12426 X21.9275 Y93.0795

N12428 X21.9190 Y92.9944

N12430 X21.9103 Y92.9100

N12432 X21.9014 Y92.8263

N12434 X21.8923 Y92.7433

N12436 X21.8830 Y92.6611

N12438 X21.8734 Y92.5795

N12440 X21.8636 Y92.4987

N12442 X21.8535 Y92.4187

N12444 X21.8431 Y92.3394

N12446 X21.8324 Y92.2608

N12448 X21.8214 Y92.1831

N12450 X21.8100 Y92.1061

N12452 X21.7983 Y92.0299

N12454 X21.7863 Y91.9545

N12456 X21.7738 Y91.8799

N12458 X21.7610 Y91.8061

N12460 X21.7477 Y91.7331

N12462 X21.7340 Y91.6610

N12464 X21.7199 Y91.5897

N12466 X21.7053 Y91.5192

N12468 X21.6903 Y91.4496

N12470 X21.6748 Y91.3809

N12472 X21.6587 Y91.3130

N12474 X21.6422 Y91.2460

N12476 X21.6251 Y91.1799

N12478 X21.6074 Y91.1146

N12480 X21.5892 Y91.0503

N12482 X21.5705 Y90.9869

N12484 X21.5511 Y90.9244

N12486 X21.5311 Y90.8628

N12488 X21.5105 Y90.8022

N12490 X21.4892 Y90.7425

N12492 X21.4674 Y90.6837

N12494 X21.4448 Y90.6259

N12496 X21.4215 Y90.5691

N12498 X21.3976 Y90.5132

N12500 X21.3729 Y90.4584

N12502 X21.3475 Y90.4045

N12504 X21.3214 Y90.3516

N12506 X21.2945 Y90.2997

N12508 X21.2668 Y90.2489

N12510 X21.2383 Y90.1990

N12512 X21.2091 Y90.1502

N12514 X21.1790 Y90.1025

N12516 X21.1481 Y90.0557

N12518 X21.1163 Y90.0101

N12520 X21.0836 Y89.9655

N12522 X21.0501 Y89.9219

N12524 X21.0157 Y89.8795

N12526 X20.9804 Y89.8381

N12528 X20.9441 Y89.7978

N12530 X20.9070 Y89.7585

N12532 X20.8691 Y89.7203

N12534 X20.8302 Y89.6830

N12536 X20.7906 Y89.6467

N12538 X20.7502 Y89.6114

N12540 X20.7089 Y89.5769

N12542 X20.6669 Y89.5433

N12544 X20.6242 Y89.5106

N12546 X20.5807 Y89.4787

N12548 X20.5364 Y89.4476

N12550 X20.4915 Y89.4173

N12552 X20.4459 Y89.3878

N12554 X20.3997 Y89.3590

N12556 X20.3527 Y89.3309

N12558 X20.3052 Y89.3034

N12560 X20.2570 Y89.2767

N12562 X20.2083 Y89.2505

N12564 X20.1589 Y89.2250

N12566 X20.1090 Y89.2000

N12568 X20.0586 Y89.1755

N12570 X20.0076 Y89.1516

N12572 X19.9562 Y89.1282

N12574 X19.9042 Y89.1053

N12576 X19.8518 Y89.0828

N12578 X19.7989 Y89.0607

N12580 X19.7456 Y89.0391

N12582 X19.6918 Y89.0178

N12584 X19.6377 Y88.9968

N12586 X19.5832 Y88.9761

N12588 X19.5283 Y88.9558

N12590 X19.4731 Y88.9357

N12592 X19.4175 Y88.9159

N12594 X19.3616 Y88.8962

N12596 X19.3054 Y88.8768

N12598 X19.2490 Y88.8575

N12600 X19.1923 Y88.8383

N12602 X19.1353 Y88.8193

N12604 X19.0782 Y88.8004

N12606 X19.0208 Y88.7815

N12608 X18.9632 Y88.7626

N12610 X18.9055 Y88.7438

N12612 X18.8476 Y88.7249

N12614 X18.7896 Y88.7060

N12616 X18.7314 Y88.6870

N12618 X18.6732 Y88.6679

N12620 X18.6148 Y88.6487

N12622 X18.5564 Y88.6294

N12624 X18.4980 Y88.6099

N12626 X18.4395 Y88.5902

N12628 X18.3811 Y88.5702

N12630 X18.3226 Y88.5500

N12632 X18.2641 Y88.5296

N12634 X18.2057 Y88.5088

N12636 X18.1474 Y88.4877

N12638 X18.0891	Y88.4663
N12640 X18.0310	Y88.4445
N12642 X17.9729	Y88.4223
N12644 X17.9150	Y88.3996
N12646 X17.8572	Y88.3765
N12648 X17.7996	Y88.3529
N12650 X17.7421	Y88.3288
N12652 X17.6849	Y88.3042
N12654 X17.6279	Y88.2790
N12656 X17.5711	Y88.2532
N12658 X17.5146	Y88.2268
N12660 X17.4583	Y88.1998
N12662 X17.4023	Y88.1722
N12664 X17.3466	Y88.1441
N12666 X17.2911	Y88.1153
N12668 X17.2359	Y88.0861
N12670 X17.1809	Y88.0564
N12672 X17.1262	Y88.0261
N12674 X17.0717	Y87.9955
N12676 X17.0175	Y87.9644
N12678 X16.9635	Y87.9329
N12680 X16.9097	Y87.9010
N12682 X16.8562	Y87.8688
N12684 X16.8029	Y87.8362
N12686 X16.7499	Y87.8034
N12688 X16.6970	Y87.7703
N12690 X16.6444	Y87.7369
N12692 X16.5920	Y87.7033
N12694 X16.5398	Y87.6695

N12696 X16.4878 Y87.6355

N12698 X16.4361 Y87.6014

N12700 X16.3845 Y87.5671

N12702 X16.3331 Y87.5328

N12704 X16.2820 Y87.4983

N12706 X16.2310 Y87.4638

N12708 X16.1802 Y87.4293

N12710 X16.1297 Y87.3948

N12712 X16.0793 Y87.3603

N12714 X16.0291 Y87.3258

N12716 X15.9790 Y87.2915

N12718 X15.9292 Y87.2572

N12720 X15.8795 Y87.2230

N12722 X15.8300 Y87.1890

N12724 X15.7807 Y87.1552

N12726 X15.7315 Y87.1215

N12728 X15.6825 Y87.0881

N12730 X15.6337 Y87.0549

N12732 X15.5850 Y87.0220

N12734 X15.5365 Y86.9894

N12736 X15.4881 Y86.9571

N12738 X15.4399 Y86.9251

N12740 X15.3918 Y86.8936

N12742 X15.3438 Y86.8624

N12744 X15.2960 Y86.8316

N12746 X15.2483 Y86.8013

N12748 X15.2008 Y86.7714

N12750 X15.1534 Y86.7421

N12752 X15.1061 Y86.7132

N12754 X15.0590 Y86.6849

N12756 X15.0119 Y86.6572

N12758 X14.9650 Y86.6301

N12760 X14.9182 Y86.6036

N12762 X14.8715 Y86.5777

N12764 X14.8249 Y86.5525

N12766 X14.7784 Y86.5280

N12768 X14.7320 Y86.5042

N12770 X14.6858 Y86.4811

N12772 X14.6396 Y86.4588

N12774 X14.5935 Y86.4373

N12776 X14.5475 Y86.4167

N12778 X14.5016 Y86.3968

N12780 X14.4557 Y86.3778

N12782 X14.4100 Y86.3598

N12784 X14.3643 Y86.3426

N12786 X14.3187 Y86.3264

N12788 X14.2732 Y86.3111

N12790 X14.2277 Y86.2969

N12792 X14.1823 Y86.2836

N12794 X14.1370 Y86.2714

N12796 X14.0917 Y86.2602

N12798 X14.0465 Y86.2501

N12800 X14.0014 Y86.2410

N12802 X13.9563 Y86.2330

N12804 X13.9113 Y86.2261

N12806 X13.8664 Y86.2203

N12808 X13.8216 Y86.2156

N12810 X13.7768 Y86.2120

N12812 X13.7321 Y86.2095

N12814 X13.6874 Y86.2081

N12816 X13.6429 Y86.2079

N12818 X13.5984 Y86.2088

N12820 X13.5540 Y86.2109

N12822 X13.5096 Y86.2142

N12824 X13.4654 Y86.2186

N12826 X13.4212 Y86.2243

N12828 X13.3771 Y86.2311

N12830 X13.3330 Y86.2391

N12832 X13.2891 Y86.2484

N12834 X13.2452 Y86.2589

N12836 X13.2014 Y86.2706

N12838 X13.1577 Y86.2836

N12840 X13.1141 Y86.2978

N12842 X13.0706 Y86.3134

N12844 X13.0271 Y86.3301

N12846 X12.9838 Y86.3482

N12848 X12.9405 Y86.3676

N12850 X12.8973 Y86.3883

N12852 X12.8542 Y86.4103

N12854 X12.8112 Y86.4336

N12856 X12.7683 Y86.4583

N12858 X12.7254 Y86.4843

N12860 X12.6827 Y86.5116

N12862 X12.6400 Y86.5404

N12864 X12.5975 Y86.5705

N12866 X12.5550 Y86.6020

N12868 X12.5127 Y86.6349

N12870 X12.4704 Y86.6692

N12872 X12.4282 Y86.7049

N12874 X12.3861 Y86.7421

N12876 X12.3442 Y86.7806

N12878 X12.3023 Y86.8207

N12880 X12.2605 Y86.8622

N12882 X12.2188 Y86.9051

N12884 X12.1772 Y86.9495

N12886 X12.1358 Y86.9955

N12888 X12.0944 Y87.0429

N12890 X12.0531 Y87.0918

N12892 X12.0119 Y87.1422

N12894 X11.9709 Y87.1942

N12896 X11.9299 Y87.2477

N12898 X11.8891 Y87.3027

N12900 X11.8483 Y87.3593

N12902 X11.8077 Y87.4175

N12904 X11.7672 Y87.4772

N12906 X11.7268 Y87.5385

N12908 X11.6865 Y87.6014

N12910 X11.6463 Y87.6659

N12912 X11.6062 Y87.7321

N12914 X11.5663 Y87.7998

N12916 X11.5264 Y87.8692

N12918 X11.4867 Y87.9402

N12920 X11.4471 Y88.0129

N12922 X11.4076 Y88.0873

N12924 X11.3682 Y88.1633

N12926 X11.3289 Y88.2410

N12928 X11.2898	Y88.3204
N12930 X11.2508	Y88.4014
N12932 X11.2119	Y88.4841
N12934 X11.1731	Y88.5684
N12936 X11.1344	Y88.6543
N12938 X11.0958	Y88.7419
N12940 X11.0573	Y88.8310
N12942 X11.0190	Y88.9218
N12944 X10.9807	Y89.0141
N12946 X10.9425	Y89.1080
N12948 X10.9045	Y89.2034
N12950 X10.8665	Y89.3003
N12952 X10.8287	Y89.3988
N12954 X10.7910	Y89.4988
N12956 X10.7533	Y89.6003
N12958 X10.7157	
N12960 X10.6783	Y89.8077
N12962 X10.6409	Y89.9136
N12964 X10.6036	Y90.0209
N12966 X10.5665	Y90.1297
N12968 X10.5294	Y90.2399
N12970 X10.4923	Y90.3515
N12972 X10.4554	Y90.4645
N12974 X10.4186	Y90.5789
N12976 X10.3818	Y90.6946
N12978 X10.3451	Y90.8117
N12980 X10.3086	Y90.9302
N12982 X10.2720	Y91.0500
N12984 X10.2356	Y91.1711

N12986 X10.1992 Y91.2935

N12988 X10.1629 Y91.4172

N12990 X10.1267 Y91.5422

N12992 X10.0906 Y91.6685

N12994 X10.0545 Y91.7960

N12996 X10.0185 Y91.9248

N12998 X9.9826 Y92.0548

N13000 X9.9467 Y92.1860

N13002 X9.9109 Y92.3184

N13004 X9.8751 Y92.4520

N13006 X9.8394 Y92.5868

N13008 X9.8038 Y92.7228

N13010 X9.7682 Y92.8599

N13012 X9.7327 Y92.9982

N13014 X9.6973 Y93.1376

N13016 X9.6619 Y93.2781

N13018 X9.6265 Y93.4198

N13020 X9.5912 Y93.5625

N13022 X9.5560 Y93.7063

N13024 X9.5208 Y93.8511

N13026 X9.4857 Y93.9971

N13028 X9.4505 Y94.1440

N13030 X9.4155 Y94.2920

N13032 X9.3805 Y94.4410

N13034 X9.3455 Y94.5911

N13036 X9.3106 Y94.7421

N13038 X9.2757 Y94.8941

N13040 X9.2408 Y95.0470

N13042 X9.2060 Y95.2009

N13044 X9.1712 Y95.3558

N13046 X9.1364 Y95.5116

N13048 X9.1017 Y95.6682

N13050 X9.0670 Y95.8259

N13052 X9.0323 Y95.9843

N13054 X8.9977 Y96.1437

N13056 X8.9631 Y96.3040

N13058 X8.9285 Y96.4650

N13060 X8.8939 Y96.6270

N13062 X8.8594 Y96.7897

N13064 X8.8248 Y96.9533

N13066 X8.7903 Y97.1177

N13068 X8.7558 Y97.2829

N13070 X8.7214 Y97.4488

N13072 X8.6869 Y97.6155

N13074 X8.6525 Y97.7830

N13076 X8.6180 Y97.9512

N13078 X8.5836 Y98.1201

N13080 X8.5492 Y98.2897

N13082 X8.5148 Y98.4601

N13084 X8.4803 Y98.6311

N13086 X8.4459 Y98.8028

N13088 X8.4115 Y98.9752

N13090 X8.3771 Y99.1482

N13092 X8.3427 Y99.3219

N13094 X8.3083 Y99.4962

N13096 X8.2739 Y99.6711

N13098 X8.2395 Y99.8466

N13100 X8.2051 Y100.0227

N13102 X8.1707 Y100.1994

N13104 X8.1362 Y100.3766

N13106 X8.1018 Y100.5544

N13108 X8.0673 Y100.7327

N13110 X8.0329 Y100.9116

N13112 X7.9984 Y101.0909

N13114 X7.9639 Y101.2708

N13116 X7.9294 Y101.4512

N13118 X7.8949 Y101.6320

N13120 X7.8603 Y101.8133

N13122 X7.8257 Y101.9950

N13124 X7.7911 Y102.1772

N13126 X7.7565 Y102.3598

N13128 X7.7218 Y102.5428

N13130 X7.6872 Y102.7262

N13132 X7.6525 Y102.9100

N13134 X7.6177 Y103.0942

N13136 X7.5830 Y103.2787

N13138 X7.5481 Y103.4636

N13140 X7.5133 Y103.6488

N13142 X7.4784 Y103.8343

N13144 X7.4435 Y104.0202

N13146 X7.4086 Y104.2063

N13148 X7.3736 Y104.3927

N13150 X7.3386 Y104.5795

N13152 X7.3035 Y104.7664

N13154 X7.2684 Y104.9536

N13156 X7.2332 Y105.1411

N13158 X7.1980 Y105.3287

N13160 X7.1627 Y105.5166

N13162 X7.1274 Y105.7047

N13164 X7.0920 Y105.8930

N13166 X7.0566 Y106.0814

N13168 X7.0211 Y106.2700

N13170 X6.9856 Y106.4587

N13172 X6.9500 Y106.6476

N13174 X6.9144 Y106.8366

N13176 X6.8787 Y107.0257

N13178 X6.8429 Y107.2149

N13180 X6.8071 Y107.4042

N13182 X6.7712 Y107.5936

N13184 X6.7352 Y107.7830

N13186 X6.6992 Y107.9724

N13188 X6.6631 Y108.1619

N13190 X6.6269 Y108.3514

N13192 X6.5907 Y108.5410

N13194 X6.5543 Y108.7305

N13196 X6.5180 Y108.9200

N13198 X6.4815 Y109.1095

N13200 X6.4449 Y109.2989

N13202 X6.4083 Y109.4883

N13204 X6.3716 Y109.6776

N13206 X6.3348 Y109.8668

N13208 X6.2980 Y110.0559

N13210 X6.2610 Y110.2449

N13212 X6.2240 Y110.4339

N13214 X6.1868 Y110.6226

N13216 X6.1496 Y110.8113

N13218 X6.1123 Y110.9997

N13220 X6.0749 Y111.1881

N13222 X6.0374 Y111.3762

N13224 X5.9998 Y111.5641

N13226 X5.9621 Y111.7518

N13228 X5.9243 Y111.9393

N13230 X5.8865 Y112.1266

N13232 X5.8485 Y112.3137

N13234 X5.8104 Y112.5004

N13236 X5.7722 Y112.6869

N13238 X5.7339 Y112.8732

N13240 X5.6955 Y113.0591

N13242 X5.6570 Y113.2447

N13244 X5.6184 Y113.4300

N13246 X5.5797 Y113.6150

N13248 X5.5408 Y113.7996

N13250 X5.5019 Y113.9839

N13252 X5.4628 Y114.1678

N13254 X5.4236 Y114.3513

N13256 X5.3843 Y114.5344

N13258 X5.3449 Y114.7171

N13260 X5.3053 Y114.8994

N13262 X5.2657 Y115.0812

N13264 X5.2259 Y115.2626

N13266 X5.1860 Y115.4436

N13268 X5.1459 Y115.6240

N13270 X5.1057 Y115.8040

N13272 X5.0654 Y115.9835

N13274 X5.0250 Y116.1625

N13276 X4.9845 Y116.3410

N13278 X4.9438 Y116.5189

N13280 X4.9029 Y116.6963

N13282 X4.8620 Y116.8731

N13284 X4.8209 Y117.0494

N13286 X4.7796 Y117.2250

N13288 X4.7382 Y117.4001

N13290 X4.6967 Y117.5745

N13292 X4.6550 Y117.7484

N13294 X4.6132 Y117.9216

N13296 X4.5712 Y118.0941

N13298 X4.5291 Y118.2660

N13300 X4.4869 Y118.4372

N13302 X4.4445 Y118.6077

N13304 X4.4019 Y118.7776

N13306 X4.3592 Y118.9467

N13308 X4.3163 Y119.1151

N13310 X4.2733 Y119.2827

N13312 X4.2301 Y119.4497

N13314 X4.1867 Y119.6158

N13316 X4.1432 Y119.7812

N13318 X4.0996 Y119.9458

N13320 X4.0557 Y120.1095

N13322 X4.0117 Y120.2725

N13324 X3.9676 Y120.4347

N13326 X3.9232 Y120.5960

N13328 X3.8787 Y120.7564

N13330 X3.8340 Y120.9160

N13332 X3.7892 Y121.0748

N13334 X3.7442 Y121.2326

N13336 X3.6990 Y121.3895

N13338 X3.6536 Y121.5456

N13340 X3.6081 Y121.7007

N13342 X3.5623 Y121.8548

N13344 X3.5164 Y122.0080

N13346 X3.4703 Y122.1603

N13348 X3.4240 Y122.3115

N13350 X3.3776 Y122.4618

N13352 X3.3309 Y122.6111

N13354 X3.2841 Y122.7593

N13356 X3.2371 Y122.9066

N13358 X3.1898 Y123.0528

N13360 X3.1424 Y123.1979

N13362 X3.0948 Y123.3420

N13364 X3.0470 Y123.4850

N13366 X2.9990 Y123.6269

N13368 X2.9508 Y123.7677

N13370 X2.9024 Y123.9074

N13372 X2.8538 Y124.0460

N13374 X2.8050 Y124.1834

N13376 X2.7560 Y124.3197

N13378 X2.7068 Y124.4548

N13380 X2.6574 Y124.5887

N13382 X2.6078 Y124.7215

N13384 X2.5579 Y124.8530

N13386 X2.5079 Y124.9833

N13388 X2.4576 Y125.1124

N13390 X2.4072 Y125.2403

N13392 X2.3565 Y125.3669

N13394 X2.3056 Y125.4922

N13396 X2.2544 Y125.6162

N13398 X2.2031 Y125.7390

N13400 X2.1515 Y125.8605

N13402 X2.0998 Y125.9806

N13404 X2.0477 Y126.0994

N13406 X1.9955 Y126.2169

N13408 X1.9430 Y126.3330

N13410 X1.8904 Y126.4477

N13412 X1.8374 Y126.5611

N13414 X1.7843 Y126.6731

N13416 X1.7309 Y126.7836

N13418 X1.6773 Y126.8928

N13420 X1.6234 Y127.0005

N13422 X1.5693 Y127.1068

N13424 X1.5150 Y127.2116

N13426 X1.4604 Y127.3149

N13428 X1.4056 Y127.4168

N13430 X1.3506 Y127.5172

N13432 X1.2953 Y127.6160

N13434 X1.2397 Y127.7134

N13436 X1.1839 Y127.8092

N13438 X1.1279 Y127.9035

N13440 X1.0716 Y127.9962

N13442 X1.0151 Y128.0874

N13444 X0.9583 Y128.1769

N13446 X0.9012 Y128.2649

N13448 X0.8439 Y128.3513

N13450 X0.7864 Y128.4360

N13452 X0.7285 Y128.5191

N13454 X0.6705 Y128.6006

N13456 X0.5600 Y128.7500
